# Gene expression profiles of the small intestinal mucosa of dogs repeatedly infected with the cestode *Echinococcus multilocularis*

**DOI:** 10.1016/j.dib.2018.01.004

**Published:** 2018-01-06

**Authors:** Hirokazu Kouguchi, Takao Irie, Jun Matsumoto, Hidefumi Furuoka, Kenji Ishiwata, Ryo Nakao, Kinpei Yagi

**Affiliations:** a Department of Infectious Diseases, Hokkaido Institute of Public Health, N19 W12, Kita-Ku, Sapporo, Hokkaido 060-0819, Japan; b Laboratory of Medical Zoology, College of Bioresource Sciences, Nihon University, Fujisawa, Kanagawa 252-0880, Japan; c Department of Basic Veterinary Medicine, Obihiro University of Agriculture and Veterinary Medicine, Inada-cho, Obihiro, Hokkaido 080-8555, Japan; d Department of Tropical Medicine, The Jikei University School of Medicine, 3-25-8, Nishi-Shimbashi, Minato-ku, Tokyo 105-8461, Japan; e Laboratory of Parasitology, Department of Disease Control, Graduate School of Veterinary Medicine, Hokkaido University, Sapporo, Hokkaido 001-0020, Japan

**Keywords:** *E. multilocularis*, Microarray, Dog, Echinococcosis, Vaccine

## Abstract

*T*he data set presented in this article is related to a previous research article entitled “ The timing of worm exclusion in dogs repeatedly infected with the cestode *Echinococcus multilocularis*” (Kouguchi et al., 2016) [1]. This article describes the genes >2-fold up- or down-regulated in the first- and repeated-infection groups compared to the healthy controls group. The gene expression profiles were generated using the Agilent-021193 Canine (V2) Gene Expression Microarray (GPL15379). The raw and normalized microarray data have been deposited with the Gene Expression Omnibus (GEO) database under accession number GSE105098.

**Specifications Table**

**Table:** 

Subject area	*Biology*
More specific subject area	*Gene expression study*
Type of data	*Table*
How data was acquired	*Agilent-021193 Canine (V2) Gene Expression Microarray (GPL15379)*
Data format	*Raw, normalized, and analyzed*
Experimental factors	*Gene expression profiles were compared between healthy controls, first-infection, and repeated-infection groups.*
Experimental features	*The relationship between the response at the small intestinal mucosa and worm exclusion in dogs repeatedly infected with E. multilocularis.*
Data source location	*Hokkaido Institute of Public Health, Sapporo, Japan, 43 °04'58.804''N; 141 °19'59.769''E.*
Data accessibility	*The raw and normalized microarray data are available from the Gene Expression Omnibus (GEO) database under accession number GSE105098.*https://www.ncbi.nlm.nih.gov/geo/query/acc.cgi?acc=GSE105098

Value of the data

• The data present the differential gene expression profiles of the small intestinal mucosa of healthy dogs and dogs infected with *Echinococcus multilocularis*.
• The data can be used to identify genes that might be involved in excluding the parasite from the small intestine of dogs.
• By comparing the first and repeated infection groups, the data could aid in the development of a mucosal vaccine.
• The data could contribute to clarifying the exclusion mechanism(s) of various canine pathogens.

## 1 Data

The present study was performed to elucidate the mechanisms underlying worm exclusion in dogs repeatedly infected with *E. multilocularis*. The small intestinal mucosa gene expression profiles of three groups (healthy, first-infection, and repeated-infection) were compared. Total RNA was isolated from the small intestinal mucosa, analyzed for quality and quantity, and used in microarray analysis with the Agilent-021193 Canine (V2) Gene Expression Microarray (GPL15379; Agilent Technologies, Santa Clara, CA, USA). Compared to the control group, 1916 and 2950 genes were >2-fold up-regulated in the first-infection and repeated-infection groups, respectively, and 2418 and 3485 genes were >2-fold down-regulated, respectively (Supplements 1 and 2).

## 2 Experimental design, materials and methods

### 2.1 Experimental design

This study was performed in strict accordance with the National Institutes of Health guide for the care and use of Laboratory animals, and the ethics committee of the Hokkaido Institute of Public Health approved the protocol for the animal experiments (permit number: K25-2). All surgeries were performed under sodium pentobarbital anesthesia, and every effort was made to minimize suffering.

Twelve dogs were divided into three groups (*n*=4): the control group (healthy dogs), the first-infection group (dogs infected with *E. multilocularis* for the first time) and the repeated-infection group (dogs repeatedly infected with the parasite 4 times). The mucosal gene expression profiles between the three groups were compared by microarray analysis.

### 2.2 Materials

*E. multilocularis* (Nemuro strain) was obtained from a dog-cotton rat life cycle routinely maintained at the Hokkaido Institute of Public Health (Sapporo, Japan). Protoscoleces (psc) were collected from cysts that developed in cotton rats 5 –14 months following oral administration of 200 parasite eggs. The Beagle dogs used in this study were purchased from KITAYAMA LABES CO., LTD. (Ina, Nagano, Japan).

### 2.3 Sample preparation

For the control group, healthy dogs were euthanized and necropsies were performed. The small intestine was removed and the central portion (approximately 15 cm) was washed with cold saline and then quickly treated with RNAlater Stabilization Solution (Thermo Fisher Scientific Inc., MA, USA) at 4 °C according to the manufacturer's instructions. These samples were preserved at –70 °C until use. For the first-infection group, 100,000 psc were orally administered to infect the dogs with *E. multilocularis*. In this group, infection continued for 6 days. For the repeated-infection group (*n*=4), 500,000 psc were administered orally for the first, second, and third infections. To terminate infection, 100 mg of praziquantel (two tablets of Droncit, Bayer-Animal Health, Leverkusen, Germany) was administered 35 days post infection (dpi), as previously described [1]. Following deworming, the dogs were re-infected by administering psc at 8–14 day intervals (Table 1). For the final infection, 100,000 psc were orally administered. After 6 days of infection, the central section of the small intestine of dogs infected with *E. multilocularis* was removed and the tissue samples were treated using the method described above.

**Table 1:** Infection regimes for the three groups of dogs.

Groups	Total number of infections	Number of dogs	Age at final infection	Infection period (days)	Days between infections
Healthy control	0	4	9		
First-infection	1	4	8	6	
Reinfection	4	4	8	30, 34, 35, 6	14, 8, 14

Tissue samples from dogs were carefully thawed in RNAlater at 25 °C. The small intestinal mucosa was scraped with a microspatula onto plastic culture dishes, and total RNA was extracted from the tissue samples using an RNeasy Mini Kit (Qiagen GmbH, Hilden, Germany) according to the manufacturer's instructions. In each group, equal amounts of purified total RNA from the four dogs were pooled. The quantity and quality of total RNA samples were determined using a Nanodrop 1000 spectrophotometer. All samples had OD260/280 ratios of 1.97–2.05 with concentrations of 187.7–258.69 ng/µL. Agilent Bioanalyzer analysis demonstrated that all samples had RNA integrity numbers of 7.3–9.3 (Table 2). The samples were hybridized to the Agilent Canine (V2) array according to the manufacturer's instructions.

**Table 2:** Spectrophotometric data and RNA integrity numbers (RINs) of the RNA samples.

Pooled sample	OD260/280	OD260/230	Quantity (ng/µL)	RIN
Healthy control	1.97	1.87	187.76	7.3
First-infection	2.02	1.96	258.69	8.7
Reinfection	2.05	1.75	249.25	9.3

### 2.4 Microarray analyses

The microarray was conducted with a Canine (V2) array (Agilent) according to the manufacturer's protocol. Scanned images were analyzed with Feature Extraction Software 11.5.1.1 (Agilent; protocol: GE1_1105_Oct12 and Grid: 021193_D_F_20141110) using default parameters, to obtain background subtracted and spatially detrended processed signal intensities. Features flagged as feature non-uniform outliers were excluded. The microarray data were normalized using GeneSpring Ver.13.1.1 software (Agilent; per chip: normalization to 75 percentile shift). Fold change (FC) analysis was performed using GeneSpring software with a threshold FC ≥ 2.0. The microarray gene list can be found on the Agilent Technologies web site (http://www.chem.agilent.com/cag/bsp/gene_lists.asp?arrayType=gene).
