# Supplementary material for: Gene expression profiles of the small intestinal mucosa of dogs repeatedly infected with the cestode Echinococcus multilocularis
Source: Data Brief. 2018 Jan 6;17:180–3. doi: 10.1016/j.dib.2018.01.004 (PMC5988226; doi:10.1016/j.dib.2018.01.004)
Supplement: Supplementary file 1 — Supplementary material [file mmc3.doc]

Conflict of Interest

　None
