# Supplementary material for: Gene expression profiles of the small intestinal mucosa of dogs repeatedly infected with the cestode Echinococcus multilocularis
Source: Data Brief. 2018 Jan 6;17:180–3. doi: 10.1016/j.dib.2018.01.004 (PMC5988226; doi:10.1016/j.dib.2018.01.004)
Supplement: Supplementary file 2 — Supplementary material [file mmc1.docx]

Supplement 1. List of differentially regulated genes between control and first-infection group.

| Upregulation | | | Downregulation | | |
| --- | --- | --- | --- | --- | --- |
| Probe name | Gene name | Fold change | Probe name | Gene name | Fold change |
| A_11_P196708 |  | 26.64 | A_11_P066011 |  | -116.01 |
| A_11_P0000025428 | regakine-1-like | 24.53 | A_11_P0000020075 | apolipoprotein C-III | -24.49 |
| A_11_P055286 | tryptase | 20.29 | A_11_P053076 | apolipoprotein C-III | -22.70 |
| A_11_P204503 | clusterin | 20.10 | A_11_P0000041134 |  | -22.70 |
| A_11_P0000024492 | tektin 3 | 19.56 | A_11_P0000020044 | cytochrome P450 2C41 | -18.80 |
| A_11_P0000029567 | carboxypeptidase A3 (mast cell) | 18.57 | A_11_P178948 |  | -16.96 |
| A_11_P0000020103 | chemokine (C-C motif) ligand 1 | 16.61 | A_11_P0000015832 |  | -15.93 |
| A_11_P212903 | clusterin | 16.20 | A_11_P191308 |  | -15.38 |
| A_11_P0000015212 |  | 15.89 | A_11_P0000019772 | uncoupling protein 3 (mitochondrial, proton carrier) | -14.77 |
| A_11_P198223 | periostin, osteoblast specific factor | 15.06 | A_11_P114966 | glycerophosphodiester phosphodiesterase domain containing 2 | -14.25 |
| A_11_P050611 | cubilin (intrinsic factor-cobalamin receptor) | 14.63 | A_11_P0000025698 | glycerophosphodiester phosphodiesterase domain containing 2 | -13.97 |
| A_11_P212743 | neuronal membrane glycoprotein M6-a-like | 14.61 | A_11_P0000041943 |  | -13.10 |
| A_11_P209683 | heat shock protein 70 | 14.12 | A_11_P086766 |  | -12.84 |
| A_11_P0000019867 | cubilin (intrinsic factor-cobalamin receptor) | 13.30 | A_11_P0000020956 | paraoxonase 3 | -12.70 |
| A_11_P055026 | heat shock protein 70 | 12.97 | A_11_P159348 | interferon-related developmental regulator 1 | -12.40 |
| A_11_P050821 | membrane-spanning 4-domains, subfamily A, member 2 | 12.81 | A_11_P0000024491 | peripheral myelin protein 22 | -12.22 |
| A_11_P0000019776 | chemokine (C-C motif) ligand 17 | 12.79 | A_11_P0000019170 |  | -11.83 |
| A_11_P055186 | heat shock protein 70 | 12.02 | A_11_P053491 | uncoupling protein 3 (mitochondrial, proton carrier) | -11.08 |
| A_11_P055911 | Fc fragment of IgE, high affinity I, receptor for; alpha polypeptide | 11.86 | A_11_P0000028581 | cytochrome P450 2G1-like | -10.84 |
| A_11_P0000031140 | histidine decarboxylase | 11.70 | A_11_P079681 | interferon-related developmental regulator 1 | -10.60 |
| A_11_P141050 |  | 11.39 | A_11_P0000035007 | UBX domain protein 8 | -10.53 |
| A_11_P053591 |  | 11.33 | A_11_P0000039861 |  | -10.11 |
| A_11_P053366 | hemoglobin subunit beta-like | 11.31 | A_11_P0000041368 | delta(4)-desaturase, sphingolipid 1 | -9.77 |
| A_11_P218668 |  | 11.21 | A_11_P0000024626 | HYDIN, axonemal central pair apparatus protein | -9.68 |
| A_11_P190298 | hemoglobin subunit beta-like | 11.01 | A_11_P0000020593 | catenin (cadherin-associated protein), alpha-like 1 | -9.03 |
| A_11_P150433 | clusterin | 10.76 | A_11_P095571 | catenin (cadherin-associated protein), alpha-like 1 | -8.77 |
| A_11_P069776 | transcobalamin I (vitamin B12 binding protein, R binder family) | 10.57 | A_11_P126066 | keratin 5 | -8.58 |
| A_11_P0000015698 | myotubularin related protein 10 | 10.49 | A_11_P113041 | exocyst complex component 3-like 2 | -8.47 |
| A_11_P054976 | clusterin | 10.33 | A_11_P00000945 | N-acetylglucosamine-1-phosphodiester alpha-N-acetylglucosaminidase | -8.36 |
| A_11_P0000020076 | clusterin | 10.33 | A_11_P099836 | S100 calcium binding protein A14 | -8.32 |
| A_11_P087926 | 3-hydroxy-3-methylglutaryl-CoA synthase 2 (mitochondrial) | 10.09 | A_11_P0000030967 | stratifin | -8.24 |
| A_11_P069866 | periostin, osteoblast specific factor | 9.94 | A_11_P0000031250 | transmembrane protease, serine 15 | -8.09 |
| A_11_P0000019890 | membrane-spanning 4-domains, subfamily A, member 2 | 9.90 | A_11_P052386 | angiotensin-converting enzyme-like | -8.07 |
| A_11_P089526 | neutrophil cytosolic factor 4, 40kDa | 9.74 | A_11_P0000035533 | activating transcription factor 3 | -7.88 |
| A_11_P0000022671 | periostin, osteoblast specific factor | 9.59 | A_11_P067681 | WNT1 inducible signaling pathway protein 2 | -7.85 |
| A_11_P060846 |  | 9.56 | A_11_P0000024761 |  | -7.84 |
| A_11_P0000020107 | chemokine (C-C motif) ligand 19 | 9.43 | A_11_P154838 |  | -7.79 |
| A_11_P190123 |  | 8.99 | A_11_P0000018515 |  | -7.72 |
| A_11_P136716 | hemoglobin, delta-like | 8.88 | A_11_P000001765 | phosphoenolpyruvate carboxykinase 1 (soluble) | -7.64 |
| A_11_P192543 | apolipoprotein E | 8.72 | A_11_P0000025072 | laminin, alpha 3 | -7.62 |
| A_11_P0000020112 | chemokine (C-C motif) receptor 3 | 8.68 | A_11_P108186 | HYDIN, axonemal central pair apparatus protein | -7.58 |
| A_11_P0000022171 | egf-like module containing, mucin-like, hormone receptor-like 2 | 8.52 | A_11_P0000039879 |  | -7.56 |
| A_11_P193688 | CD1e molecule | 8.51 | A_11_P0000024553 | ISG15 ubiquitin-like modifier | -7.44 |
| A_11_P194308 | major histocompatibility complex, class II, DM beta | 8.50 | A_11_P096991 |  | -7.36 |
| A_11_P055021 | heat shock protein 70 | 8.46 | A_11_P066061 |  | -7.23 |
| A_11_P183038 | glycine amidinotransferase (L-arginine:glycine amidinotransferase) | 8.05 | A_11_P0000029776 | phosphoenolpyruvate carboxykinase 1 (soluble) | -7.17 |
| A_11_P0000020124 | gastric intrinsic factor (vitamin B synthesis) | 7.79 | A_11_P0000041660 |  | -7.06 |
| A_11_P160268 |  | 7.77 | A_11_P062401 |  | -7.00 |
| A_11_P0000021083 | ribonuclease pancreatic-like | 7.72 | A_11_P0000040074 |  | -6.99 |
| A_11_P0000022617 | peptidase inhibitor 3, skin-derived | 7.62 | A_11_P0000032314 | sterile alpha motif domain containing 8 | -6.98 |
| A_11_P062926 | WDFY family member 4 | 7.51 | A_11_P063791 | inositol polyphosphate-5-phosphatase F | -6.96 |
| A_11_P158998 |  | 7.48 | A_11_P0000018359 |  | -6.92 |
| A_11_P206293 |  | 7.35 | A_11_P092121 | solute carrier family 16 (aromatic amino acid transporter), member 10 | -6.88 |
| A_11_P0000015040 | solute carrier family 16, member 9 | 7.28 | A_11_P111641 | CD274 molecule | -6.84 |
| A_11_P0000020227 | MHC class II DLA DRB1 beta chain | 7.20 | A_11_P0000037183 |  | -6.69 |
| A_11_P0000027851 | spleen focus forming virus (SFFV) proviral integration oncogene | 6.98 | A_11_P053546 | serum/glucocorticoid regulated kinase 1 | -6.68 |
| A_11_P0000027413 |  | 6.95 | A_11_P089851 | ring finger protein 149 | -6.65 |
| A_11_P0000015499 |  | 6.93 | A_11_P0000014359 |  | -6.60 |
| A_11_P0000020109 | chemokine (C-C motif) ligand 21 | 6.92 | A_11_P0000015549 |  | -6.54 |
| A_11_P052666 | major histocompatibility complex, class II, DM beta | 6.75 | A_11_P0000021311 | apolipoprotein B (including Ag(x) antigen) | -6.53 |
| A_11_P0000019980 | calcitonin-related polypeptide alpha | 6.62 | A_11_P0000012160 |  | -6.49 |
| A_11_P060556 | immunoglobulin iota chain-like | 6.58 | A_11_P0000028141 | one cut homeobox 2 | -6.35 |
| A_11_P109326 | chromosome 3 open reading frame, human C15orf40 | 6.41 | A_11_P118436 | solute carrier family 4, sodium bicarbonate cotransporter, member 8 | -6.30 |
| A_11_P0000020185 | desmin | 6.34 | A_11_P0000014525 |  | -6.27 |
| A_11_P054901 | major histocompatibility complex, class II, DQ beta 1 | 6.34 | A_11_P105281 |  | -6.23 |
| A_11_P0000019899 | v-kit Hardy-Zuckerman 4 feline sarcoma viral oncogene homolog | 6.34 | A_11_P0000038801 | phosphoenolpyruvate carboxykinase 1 (soluble) | -6.21 |
| A_11_P105036 | CD74 molecule, major histocompatibility complex, class II invariant chain | 6.30 | A_11_P0000024368 | retinoic acid induced 14 | -6.18 |
| A_11_P0000024341 |  | 6.26 | A_11_P051501 | 2'-5'-oligoadenylate synthetase-like | -6.14 |
| A_11_P0000015098 | collagen, type VI, alpha 1 | 6.26 | A_11_P153298 |  | -6.14 |
| A_11_P0000018243 | CD74 molecule, major histocompatibility complex, class II invariant chain | 6.23 | A_11_P209168 |  | -6.12 |
| A_11_P167858 |  | 6.23 | A_11_P149463 | lectin, galactoside-binding, soluble, 3 | -6.12 |
| A_11_P192703 |  | 6.20 | A_11_P052741 | lectin, galactoside-binding, soluble, 3 | -6.10 |
| A_11_P136836 |  | 6.19 | A_11_P000002434 | shroom family member 1 | -6.04 |
| A_11_P176483 |  | 6.18 | A_11_P000003141 |  | -6.04 |
| A_11_P123421 | lysozyme | 6.17 | A_11_P177398 | retinoic acid induced 14 | -6.00 |
| A_11_P054681 |  | 6.15 | A_11_P0000039817 |  | -5.98 |
| A_11_P179718 |  | 6.11 | A_11_P185738 |  | -5.96 |
| A_11_P00000884 | annexin A6 | 6.02 | A_11_P057061 | dehydrogenase/reductase (SDR family) member 9 | -5.95 |
| A_11_P0000022690 | heat shock 105kDa/110kDa protein 1 | 5.97 | A_11_P0000040309 |  | -5.95 |
| A_11_P087831 | tetraspanin 2 | 5.91 | A_11_P055476 | family with sequence similarity 72, member A | -5.94 |
| A_11_P079441 | neuropeptide Y | 5.87 | A_11_P000007662 |  | -5.92 |
| A_11_P188463 |  | 5.86 | A_11_P066186 | G protein-coupled receptor, family C, group 5, member A | -5.92 |
| A_11_P0000015954 |  | 5.86 | A_11_P162258 | angiotensin-converting enzyme-like | -5.92 |
| A_11_P069986 | heat shock 105kDa/110kDa protein 1 | 5.84 | A_11_P0000026944 |  | -5.81 |
| A_11_P0000038736 | Thy-1 cell surface antigen | 5.84 | A_11_P087161 | atlastin GTPase 2 | -5.80 |
| A_11_P052671 |  | 5.83 | A_11_P0000015804 |  | -5.77 |
| A_11_P0000039612 | phospholipase C-like 1 | 5.82 | A_11_P070621 | transmembrane 4 L six family member 20 | -5.76 |
| A_11_P051431 | fibronectin 1 | 5.74 | A_11_P0000015647 |  | -5.76 |
| A_11_P057266 | FK506 binding protein 7 | 5.72 | A_11_P0000019733 | chymotrypsin-like elastase family, member 1 | -5.73 |
| A_11_P126011 | chromosome 3 open reading frame, human C15orf40 | 5.72 | A_11_P213008 |  | -5.72 |
| A_11_P141818 |  | 5.71 | A_11_P0000020059 | laminin, gamma 2 | -5.71 |
| A_11_P185333 | stathmin-like 2 | 5.70 | A_11_P063786 | inositol polyphosphate-5-phosphatase F | -5.66 |
| A_11_P0000020301 | lysozyme | 5.69 | A_11_P0000013414 |  | -5.65 |
| A_11_P052649 | major histocompatibility complex, class II, DQ beta 1 | 5.68 | A_11_P123656 |  | -5.60 |
| A_11_P198578 | transcription factor 21 | 5.68 | A_11_P054966 | chymotrypsin-like elastase family, member 1 | -5.60 |
| A_11_P0000017045 | stannin | 5.61 | A_11_P0000012684 |  | -5.60 |
| A_11_P133791 | neuronal membrane glycoprotein M6-a-like | 5.61 | A_11_P000005396 |  | -5.58 |
| A_11_P0000014930 |  | 5.61 | A_11_P177553 |  | -5.57 |
| A_11_P138566 | glycine amidinotransferase (L-arginine:glycine amidinotransferase) | 5.60 | A_11_P0000010397 |  | -5.56 |
| A_11_P174683 | complement component 3 | 5.60 | A_11_P159593 |  | -5.54 |
| A_11_P053801 | TIMP metallopeptidase inhibitor 1 | 5.59 | A_11_P062486 |  | -5.52 |
| A_11_P157453 |  | 5.59 | A_11_P050686 | cytochrome P450 2B11 | -5.50 |
| A_11_P0000015308 | stathmin-like 2 | 5.57 | A_11_P0000028731 | cell death-inducing DFFA-like effector c | -5.47 |
| A_11_P119561 | MHC class II DR alpha chain | 5.56 | A_11_P0000022512 | membrane metallo-endopeptidase | -5.45 |
| A_11_P0000027724 |  | 5.56 | A_11_P167208 |  | -5.43 |
| A_11_P151618 |  | 5.56 | A_11_P0000025394 | monocyte to macrophage differentiation-associated | -5.41 |
| A_11_P0000020121 | hydroxysteroid (11-beta) dehydrogenase 1 | 5.55 | A_11_P0000017785 |  | -5.40 |
| A_11_P054896 | major histocompatibility complex, class II, DQ beta 1 | 5.51 | A_11_P052536 | nephronophthisis 1 (juvenile) | -5.37 |
| A_11_P156263 |  | 5.51 | A_11_P0000024251 | HECT and RLD domain containing E3 ubiquitin protein ligase 4 | -5.36 |
| A_11_P198273 | heat shock 105kDa/110kDa protein 1 | 5.50 | A_11_P214443 |  | -5.35 |
| A_11_P150838 | complement component 1, s subcomponent | 5.49 | A_11_P131751 | cytidine monophosphate (UMP-CMP) kinase 2, mitochondrial | -5.33 |
| A_11_P055056 | tryptase | 5.48 | A_11_P0000026289 | tumor necrosis factor (ligand) superfamily, member 15 | -5.29 |
| A_11_P0000025577 |  | 5.47 | A_11_P075111 | cell death-inducing DFFA-like effector c | -5.28 |
| A_11_P0000029111 | complement factor D (adipsin) | 5.44 | A_11_P098691 | plexin A2 | -5.27 |
| A_11_P0000026327 | major histocompatibility complex, class II, DM alpha | 5.43 | A_11_P157613 |  | -5.25 |
| A_11_P103166 | thymocyte selection associated family member 2 | 5.41 | A_11_P206953 |  | -5.25 |
| A_11_P099176 | selectin L | 5.40 | A_11_P148063 | cytochrome P450 2C41 | -5.22 |
| A_11_P0000024446 | matrix metallopeptidase 12 (macrophage elastase) | 5.40 | A_11_P190598 | membrane metallo-endopeptidase | -5.20 |
| A_11_P0000015620 | PDZ and LIM domain 3 | 5.35 | A_11_P0000010975 |  | -5.17 |
| A_11_P218553 | CD83 molecule | 5.35 | A_11_P000006423 |  | -5.16 |
| A_11_P130296 |  | 5.34 | A_11_P0000030842 | granzyme A (granzyme 1, cytotoxic T-lymphocyte-associated serine esterase 3) | -5.15 |
| A_11_P113656 | TYRO protein tyrosine kinase binding protein | 5.34 | A_11_P051261 | phospholipase A2, group VII (platelet-activating factor acetylhydrolase, plasma) | -5.13 |
| A_11_P0000028093 | chemokine (C-X-C motif) receptor 4 | 5.33 | A_11_P0000033294 | lysophosphatidic acid receptor 3 | -5.12 |
| A_11_P0000025278 | adenylate kinase 7 | 5.33 | A_11_P107266 | jun proto-oncogene | -5.11 |
| A_11_P051116 | CD40 molecule, TNF receptor superfamily member 5 | 5.32 | A_11_P0000033903 | angiotensin-converting enzyme-like | -5.11 |
| A_11_P0000024047 | Fc fragment of IgE, high affinity I, receptor for; alpha polypeptide | 5.25 | A_11_P0000033713 | guanine nucleotide binding protein (G protein), gamma 2 | -5.10 |
| A_11_P109436 |  | 5.24 | A_11_P085286 | glutamic pyruvate transaminase (alanine aminotransferase) 2 | -5.09 |
| A_11_P089781 | chromosome 10 open reading frame, human C2orf40 | 5.21 | A_11_P0000038034 |  | -5.04 |
| A_11_P104019 |  | 5.17 | A_11_P0000024415 | apolipoprotein A-I | -5.04 |
| A_11_P179953 |  | 5.13 | A_11_P0000016134 |  | -5.03 |
| A_11_P0000021957 | apolipoprotein E | 5.12 | A_11_P0000017895 |  | -5.01 |
| A_11_P121641 | neurofilament, medium polypeptide | 5.05 | A_11_P055481 | SLIT-ROBO Rho GTPase activating protein 2 | -4.99 |
| A_11_P167993 | platelet derived growth factor D | 5.05 | A_11_P103661 |  | -4.97 |
| A_11_P0000032983 | procollagen C-endopeptidase enhancer | 5.04 | A_11_P0000029777 |  | -4.97 |
| A_11_P133126 |  | 5.04 | A_11_P0000010570 |  | -4.96 |
| A_11_P0000020202 | chymase 1, mast cell | 5.02 | A_11_P0000038773 |  | -4.90 |
| A_11_P051466 | secreted phosphoprotein 1 | 5.01 | A_11_P087061 | calpain 13 | -4.89 |
| A_11_P0000019048 |  | 5.00 | A_11_P119366 | S100 calcium binding protein A2 | -4.86 |
| A_11_P000005639 |  | 4.99 | A_11_P000005655 |  | -4.83 |
| A_11_P0000024961 | phospholipase A2, group IVA (cytosolic, calcium-dependent) | 4.97 | A_11_P0000019083 |  | -4.82 |
| A_11_P0000020084 | activation-induced cytidine deaminase | 4.96 | A_11_P0000040107 |  | -4.81 |
| A_11_P0000020370 | apolipoprotein L, 5 | 4.96 | A_11_P0000040627 |  | -4.80 |
| A_11_P123561 |  | 4.95 | A_11_P0000031534 | solute carrier family 6 (neutral amino acid transporter), member 19 | -4.80 |
| A_11_P0000023290 | glutathione peroxidase 8 (putative) | 4.94 | A_11_P151413 | bone marrow stromal cell antigen 1 | -4.78 |
| A_11_P051436 | fibronectin 1 | 4.94 | A_11_P0000022786 | unc-51 like autophagy activating kinase 1 | -4.75 |
| A_11_P076931 | complement component 3 | 4.93 | A_11_P193963 |  | -4.73 |
| A_11_P051056 | egf-like module containing, mucin-like, hormone receptor-like 3 | 4.92 | A_11_P190368 |  | -4.72 |
| A_11_P066376 | complement component 1, s subcomponent | 4.91 | A_11_P0000021769 | serum/glucocorticoid regulated kinase 1 | -4.71 |
| A_11_P000004603 |  | 4.90 | A_11_P173603 |  | -4.69 |
| A_11_P095311 | tropomyosin 2 (beta) | 4.88 | A_11_P0000015806 |  | -4.67 |
| A_11_P111551 | phosphoglucomutase 5 | 4.87 | A_11_P072951 | CD36 molecule (thrombospondin receptor) | -4.67 |
| A_11_P0000025285 | heat shock protein 90kDa alpha (cytosolic), class A member 1 | 4.82 | A_11_P126116 |  | -4.66 |
| A_11_P0000025314 | mast cell immunoglobulin-like receptor 1 | 4.81 | A_11_P0000036968 |  | -4.66 |
| A_11_P050521 | carcinoembryonic antigen-related cell adhesion molecule 30 | 4.80 | A_11_P0000015759 |  | -4.64 |
| A_11_P0000020111 | mastin | 4.78 | A_11_P0000025943 | caspase recruitment domain family, member 10 | -4.63 |
| A_11_P0000036880 |  | 4.75 | A_11_P0000019808 | carboxylesterase 1 | -4.62 |
| A_11_P162343 | fibrinogen-like 1 | 4.74 | A_11_P096086 | acetylcholinesterase | -4.61 |
| A_11_P055246 | MHC class II DLA DRB1 beta chain | 4.74 | A_11_P118496 | monocyte to macrophage differentiation-associated | -4.61 |
| A_11_P138311 |  | 4.73 | A_11_P000008274 |  | -4.60 |
| A_11_P174938 |  | 4.72 | A_11_P098876 | laminin, gamma 2 | -4.59 |
| A_11_P0000019946 | decorin | 4.71 | A_11_P0000030776 | N-myristoyltransferase 2 | -4.55 |
| A_11_P0000031208 |  | 4.71 | A_11_P097046 | transmembrane channel-like 7 | -4.55 |
| A_11_P167963 |  | 4.71 | A_11_P0000010012 |  | -4.51 |
| A_11_P0000019527 |  | 4.70 | A_11_P0000032558 | GRAM domain containing 1B | -4.50 |
| A_11_P148083 | tissue factor pathway inhibitor (lipoprotein-associated coagulation inhibitor) | 4.69 | A_11_P122586 | myopalladin | -4.49 |
| A_11_P107761 | retinol binding protein 7, cellular | 4.69 | A_11_P126736 | cathepsin G | -4.48 |
| A_11_P084686 | v-kit Hardy-Zuckerman 4 feline sarcoma viral oncogene homolog | 4.68 | A_11_P0000019487 |  | -4.48 |
| A_11_P058656 | ELL associated factor 2 | 4.68 | A_11_P0000019916 | phospholipase A2, group VII (platelet-activating factor acetylhydrolase, plasma) | -4.47 |
| A_11_P124036 | cholesterol 25-hydroxylase | 4.67 | A_11_P0000040261 |  | -4.46 |
| A_11_P124686 | spleen tyrosine kinase | 4.65 | A_11_P176468 |  | -4.42 |
| A_11_P063016 | arachidonate 5-lipoxygenase | 4.64 | A_11_P0000020339 | phosphomannomutase 1 | -4.41 |
| A_11_P00000760 |  | 4.64 | A_11_P0000011374 |  | -4.39 |
| A_11_P127181 |  | 4.64 | A_11_P050691 | 2'-5'-oligoadenylate synthetase 1, 40/46kDa | -4.38 |
| A_11_P0000040999 | heat shock 70kDa protein 4-like | 4.63 | A_11_P0000012254 |  | -4.37 |
| A_11_P187538 |  | 4.63 | A_11_P059231 | HECT and RLD domain containing E3 ubiquitin protein ligase family member 6 | -4.37 |
| A_11_P058286 | filamin A interacting protein 1-like | 4.62 | A_11_P0000011690 |  | -4.36 |
| A_11_P0000034950 | chloride intracellular channel 2 | 4.61 | A_11_P0000036612 |  | -4.36 |
| A_11_P217238 |  | 4.60 | A_11_P074856 | DnaJ (Hsp40) homolog, subfamily B, member 8 | -4.35 |
| A_11_P0000029115 | protease, serine, 57 | 4.60 | A_11_P000003578 |  | -4.33 |
| A_11_P203353 |  | 4.58 | A_11_P098176 | chloride channel accessory 4 | -4.32 |
| A_11_P0000016347 | SH2 domain containing 1A | 4.58 | A_11_P000004156 |  | -4.30 |
| A_11_P0000039724 |  | 4.55 | A_11_P216803 |  | -4.27 |
| A_11_P167428 |  | 4.54 | A_11_P0000022873 | leukemia inhibitory factor (cholinergic differentiation factor) | -4.26 |
| A_11_P0000016456 |  | 4.52 | A_11_P0000010987 |  | -4.25 |
| A_11_P060601 |  | 4.50 | A_11_P075861 | transmembrane protein 89 | -4.23 |
| A_11_P0000015556 |  | 4.49 | A_11_P0000041969 |  | -4.22 |
| A_11_P084736 | insulin-like growth factor binding protein 7 | 4.44 | A_11_P074266 | cystatin E/M | -4.21 |
| A_11_P0000034003 | ADP-ribosylation factor-like protein 5C-like | 4.43 | A_11_P0000030985 | grainyhead-like 3 (Drosophila) | -4.20 |
| A_11_P203823 |  | 4.43 | A_11_P000006225 |  | -4.19 |
| A_11_P0000019723 | solute carrier family 46, member 2 | 4.43 | A_11_P0000024094 | abhydrolase domain containing 2 | -4.19 |
| A_11_P0000031801 | FK506 binding protein 7 | 4.43 | A_11_P0000017313 |  | -4.18 |
| A_11_P094304 | fms-related tyrosine kinase 4 | 4.43 | A_11_P054431 | CD14 molecule | -4.18 |
| A_11_P0000015886 |  | 4.41 | A_11_P0000022436 | tetratricopeptide repeat and ankyrin repeat containing 1 | -4.17 |
| A_11_P0000020226 | major histocompatibility complex, class II, DQ beta 1 | 4.41 | A_11_P0000022385 | LIM domain 7 | -4.16 |
| A_11_P090661 | allograft inflammatory factor 1 | 4.41 | A_11_P0000034525 | VENT homeobox | -4.15 |
| A_11_P0000021917 |  | 4.41 | A_11_P0000019828 | Niemann-Pick disease, type C1 | -4.15 |
| A_11_P139356 | homeobox B8 | 4.40 | A_11_P0000040658 | myopalladin | -4.13 |
| A_11_P0000020007 | heat shock 27kDa protein 1 | 4.39 | A_11_P152253 | diacylglycerol O-acyltransferase 2 | -4.13 |
| A_11_P155663 |  | 4.38 | A_11_P0000014831 |  | -4.13 |
| A_11_P052656 | major histocompatibility complex, class II, DQ alpha 1 | 4.38 | A_11_P180118 |  | -4.13 |
| A_11_P0000016445 | protein kinase C and casein kinase substrate in neurons 1 | 4.38 | A_11_P078041 | insulin induced gene 1 | -4.11 |
| A_11_P217073 |  | 4.36 | A_11_P0000032779 | hairy and enhancer of split 4 (Drosophila) | -4.11 |
| A_11_P0000028442 | CD33 molecule | 4.36 | A_11_P0000030484 | ubiquitin specific peptidase 18 | -4.11 |
| A_11_P0000014068 | SWAP switching B-cell complex 70kDa subunit | 4.35 | A_11_P115771 | G protein-coupled receptor 119 | -4.10 |
| A_11_P147103 |  | 4.35 | A_11_P109186 | abhydrolase domain containing 2 | -4.09 |
| A_11_P059506 |  | 4.34 | A_11_P129361 | chromosome 8 open reading frame, human C14orf37 | -4.07 |
| A_11_P058996 | chemokine (C-X-C motif) ligand 13 | 4.33 | A_11_P206083 |  | -4.07 |
| A_11_P063781 | regulator of G-protein signaling 10 | 4.33 | A_11_P0000014155 |  | -4.06 |
| A_11_P066351 | CD163 molecule | 4.32 | A_11_P124566 | EF-hand calcium binding domain 3 | -4.03 |
| A_11_P0000026779 | frizzled family receptor 1 | 4.32 | A_11_P00000496 |  | -4.03 |
| A_11_P0000037919 | cytochrome b-245, beta polypeptide (chronic granulomatous disease) | 4.32 | A_11_P205498 | abhydrolase domain containing 2 | -4.02 |
| A_11_P0000029155 | malic enzyme 3, NADP(+)-dependent, mitochondrial | 4.31 | A_11_P0000021924 | family with sequence similarity 71, member E1 | -4.02 |
| A_11_P000003239 |  | 4.31 | A_11_P141768 |  | -4.01 |
| A_11_P216443 | peptidyl arginine deiminase, type IV | 4.29 | A_11_P0000039986 |  | -4.00 |
| A_11_P0000026757 | interferon regulatory factor 5 | 4.28 | A_11_P0000021212 | insulin induced gene 1 | -4.00 |
| A_11_P147118 | collagen, type I, alpha 2 | 4.27 | A_11_P114366 |  | -4.00 |
| A_11_P0000040294 | heparanase 2 | 4.25 | A_11_P194963 |  | -3.99 |
| A_11_P053541 | bactericidal/permeability-increasing protein | 4.24 | A_11_P0000039193 |  | -3.99 |
| A_11_P073356 |  | 4.24 | A_11_P0000028560 | pleckstrin homology-like domain, family B, member 3 | -3.98 |
| A_11_P193718 |  | 4.24 | A_11_P211903 |  | -3.98 |
| A_11_P0000018663 |  | 4.24 | A_11_P191148 | ectonucleotide pyrophosphatase/phosphodiesterase 2 | -3.98 |
| A_11_P0000024578 | retinol binding protein 7, cellular | 4.23 | A_11_P200968 |  | -3.98 |
| A_11_P0000016302 |  | 4.23 | A_11_P0000017066 |  | -3.97 |
| A_11_P139516 | T-cell immunoglobulin and mucin domain containing 4 | 4.22 | A_11_P180383 | inositol polyphosphate-5-phosphatase F | -3.96 |
| A_11_P192433 | tropomyosin 2 (beta) | 4.22 | A_11_P216593 |  | -3.96 |
| A_11_P0000017700 | protein kinase, cGMP-dependent, type I | 4.21 | A_11_P118361 |  | -3.96 |
| A_11_P058311 | transmembrane protein 45A | 4.19 | A_11_P0000016906 |  | -3.95 |
| A_11_P152618 | insulin-like growth factor 2 (somatomedin A) | 4.18 | A_11_P165373 |  | -3.94 |
| A_11_P203468 |  | 4.18 | A_11_P126356 |  | -3.94 |
| A_11_P0000021417 | actin, gamma 2, smooth muscle, enteric | 4.17 | A_11_P0000011381 |  | -3.93 |
| A_11_P0000022995 | C-type lectin domain family 12, member A | 4.17 | A_11_P212703 |  | -3.92 |
| A_11_P144638 | four and a half LIM domains 3 | 4.16 | A_11_P000001061 |  | -3.92 |
| A_11_P0000016527 |  | 4.16 | A_11_P0000031594 | ADP-ribosylation factor-like 14 | -3.92 |
| A_11_P0000031372 | multimerin 1 | 4.15 | A_11_P217143 |  | -3.91 |
| A_11_P0000028013 |  | 4.15 | A_11_P191753 | tripartite motif containing 36 | -3.91 |
| A_11_P0000041754 | WDFY family member 4 | 4.15 | A_11_P150843 | delta(4)-desaturase, sphingolipid 1 | -3.91 |
| A_11_P201553 | Down syndrome cell adhesion molecule like 1 | 4.14 | A_11_P068801 | folate receptor 1 (adult) | -3.91 |
| A_11_P0000023641 |  | 4.14 | A_11_P0000040081 |  | -3.89 |
| A_11_P0000034467 | insulin-like growth factor binding protein 3 | 4.13 | A_11_P197743 |  | -3.89 |
| A_11_P0000015558 | chemokine (C-X-C motif) receptor 4 | 4.10 | A_11_P050511 | neurofilament, heavy polypeptide | -3.88 |
| A_11_P0000021095 | muscle LIM protein Mlp84B-like | 4.10 | A_11_P0000033833 |  | -3.88 |
| A_11_P106336 | interstitial collagenase-like | 4.09 | A_11_P096083 |  | -3.88 |
| A_11_P0000015092 |  | 4.08 | A_11_P170323 |  | -3.88 |
| A_11_P087786 | olfactomedin-like 3 | 4.07 | A_11_P175823 |  | -3.86 |
| A_11_P136986 | cadherin 11, type 2, OB-cadherin (osteoblast) | 4.07 | A_11_P148643 |  | -3.86 |
| A_11_P120751 | phosphoserine aminotransferase 1 | 4.06 | A_11_P070521 | bone morphogenetic protein 1 | -3.85 |
| A_11_P155233 |  | 4.06 | A_11_P0000014896 | dual specificity phosphatase 6 | -3.84 |
| A_11_P0000014793 |  | 4.05 | A_11_P0000028169 | tumor necrosis factor, alpha-induced protein 3 | -3.83 |
| A_11_P078196 | secreted frizzled-related protein 1 | 4.05 | A_11_P052821 | cytochrome P-450 3A12 | -3.83 |
| A_11_P0000023828 | lymphocyte antigen 86 | 4.03 | A_11_P0000029121 |  | -3.83 |
| A_11_P0000027330 | potassium voltage-gated channel, delayed-rectifier, subfamily S, member 3 | 4.02 | A_11_P0000010433 |  | -3.83 |
| A_11_P050041 | prostaglandin E synthase | 4.00 | A_11_P084101 | transmembrane protein 65 | -3.83 |
| A_11_P136741 |  | 3.99 | A_11_P0000012544 |  | -3.81 |
| A_11_P163398 | interstitial collagenase-like | 3.99 | A_11_P150518 |  | -3.81 |
| A_11_P089701 | aldehyde dehydrogenase 1 family, member L2 | 3.99 | A_11_P205298 | dual specificity phosphatase 6 | -3.81 |
| A_11_P0000018686 | collagen, type VI, alpha 1 | 3.99 | A_11_P061056 | 3'-phosphoadenosine 5'-phosphosulfate synthase 2 | -3.81 |
| A_11_P0000039311 | glutathione peroxidase 3 (plasma) | 3.98 | A_11_P0000038356 |  | -3.81 |
| A_11_P184623 | moesin | 3.98 | A_11_P0000041688 |  | -3.80 |
| A_11_P0000017921 |  | 3.98 | A_11_P149008 |  | -3.79 |
| A_11_P213338 | transglutaminase 2 | 3.98 | A_11_P0000039659 |  | -3.79 |
| A_11_P0000021854 | annexin A1 | 3.97 | A_11_P197093 |  | -3.79 |
| A_11_P151068 | folate receptor 2 (fetal) | 3.97 | A_11_P0000027404 | solute carrier family 20 (phosphate transporter), member 1 | -3.79 |
| A_11_P0000025517 | prostaglandin E synthase | 3.97 | A_11_P173368 |  | -3.78 |
| A_11_P177303 | MHC class II DLA DRB1 beta chain | 3.97 | A_11_P0000013956 |  | -3.76 |
| A_11_P0000037780 |  | 3.96 | A_11_P213728 |  | -3.76 |
| A_11_P187783 | cysteine and glycine-rich protein 1-like | 3.95 | A_11_P109191 | abhydrolase domain containing 2 | -3.76 |
| A_11_P170183 | latrophilin 2 | 3.95 | A_11_P0000032128 | bone marrow stromal cell antigen 1 | -3.75 |
| A_11_P200643 |  | 3.95 | A_11_P189538 |  | -3.75 |
| A_11_P0000022320 | spondin 1, extracellular matrix protein | 3.94 | A_11_P0000014244 |  | -3.74 |
| A_11_P139471 | class II, major histocompatibility complex, transactivator | 3.93 | A_11_P0000016883 |  | -3.74 |
| A_11_P051471 | secreted phosphoprotein 1 | 3.93 | A_11_P075731 | uncharacterized LOC476620 | -3.74 |
| A_11_P113621 |  | 3.92 | A_11_P000008146 |  | -3.74 |
| A_11_P0000018425 |  | 3.92 | A_11_P219313 | keratin 12 | -3.73 |
| A_11_P095286 | CD72 molecule | 3.92 | A_11_P147558 |  | -3.73 |
| A_11_P168463 |  | 3.91 | A_11_P109176 | interferon stimulated exonuclease gene 20kDa | -3.72 |
| A_11_P078451 | fibrinogen-like 1 | 3.91 | A_11_P202233 |  | -3.72 |
| A_11_P0000041342 |  | 3.90 | A_11_P148678 | tripartite motif containing 29 | -3.72 |
| A_11_P053796 | matrix metallopeptidase 9 (gelatinase B, 92kDa gelatinase, 92kDa type IV collagenase) | 3.90 | A_11_P098266 |  | -3.72 |
| A_11_P182298 | junctional adhesion molecule 2 | 3.89 | A_11_P0000039973 |  | -3.71 |
| A_11_P0000035241 |  | 3.89 | A_11_P051251 | keratin 12 | -3.71 |
| A_11_P0000026449 | opioid growth factor receptor-like 1 | 3.88 | A_11_P075701 | hyaluronoglucosaminidase 1 | -3.70 |
| A_11_P171253 | chloride intracellular channel 2 | 3.87 | A_11_P000006714 |  | -3.70 |
| A_11_P163508 |  | 3.86 | A_11_P114196 |  | -3.70 |
| A_11_P188048 |  | 3.86 | A_11_P109641 | bone marrow stromal cell antigen 1 | -3.69 |
| A_11_P168523 |  | 3.85 | A_11_P084021 | ectonucleotide pyrophosphatase/phosphodiesterase 2 | -3.69 |
| A_11_P054676 |  | 3.85 | A_11_P0000028812 | sema domain, immunoglobulin domain (Ig), short basic domain, secreted, (semaphorin) 3B | -3.69 |
| A_11_P0000024685 | serpin peptidase inhibitor, clade E (nexin, plasminogen activator inhibitor type 1), member 1 | 3.85 | A_11_P183738 |  | -3.68 |
| A_11_P193453 | complement component 1, q subcomponent, C chain | 3.84 | A_11_P0000041077 |  | -3.68 |
| A_11_P091586 | bone morphogenetic protein 5 | 3.84 | A_11_P0000035217 |  | -3.68 |
| A_11_P117196 |  | 3.83 | A_11_P146398 |  | -3.67 |
| A_11_P0000032340 | docking protein 3 | 3.82 | A_11_P0000020590 |  | -3.66 |
| A_11_P121116 | coagulation factor II (thrombin) receptor-like 2 | 3.81 | A_11_P0000012359 |  | -3.66 |
| A_11_P203419 |  | 3.81 | A_11_P00000988 | family with sequence similarity 129, member B | -3.66 |
| A_11_P050776 | dystrophin | 3.81 | A_11_P0000015182 |  | -3.66 |
| A_11_P074966 | FYVE, RhoGEF and PH domain containing 5 | 3.80 | A_11_P0000020597 | lysophosphatidic acid receptor 1 | -3.66 |
| A_11_P0000019827 | cytotoxic T-lymphocyte-associated protein 4 | 3.80 | A_11_P202478 | arginine vasopressin receptor 2 | -3.66 |
| A_11_P115586 | plastin 3 | 3.79 | A_11_P095951 | claudin 4 | -3.65 |
| A_11_P00000707 | DnaJ (Hsp40) homolog, subfamily A, member 4 | 3.79 | A_11_P0000032854 |  | -3.64 |
| A_11_P0000023004 | complement component 1, r subcomponent | 3.78 | A_11_P184798 | solute carrier family 20 (phosphate transporter), member 1 | -3.64 |
| A_11_P106172 | crystallin, alpha B | 3.77 | A_11_P195408 | O-linked N-acetylglucosamine (GlcNAc) transferase | -3.64 |
| A_11_P00000269 | vestigial like 4 (Drosophila) | 3.77 | A_11_P0000041761 |  | -3.64 |
| A_11_P00000879 | secreted protein, acidic, cysteine-rich (osteonectin) | 3.76 | A_11_P155253 |  | -3.63 |
| A_11_P0000023806 | schwannomin interacting protein 1 | 3.76 | A_11_P059251 | family with sequence similarity 13, member A | -3.63 |
| A_11_P078161 | plasminogen activator, tissue | 3.76 | A_11_P189168 |  | -3.62 |
| A_11_P153013 |  | 3.76 | A_11_P104141 | HECT and RLD domain containing E3 ubiquitin protein ligase 4 | -3.62 |
| A_11_P153398 | cysteine-rich secretory protein LCCL domain containing 2 | 3.75 | A_11_P050261 | NPC1 (Niemann-Pick disease, type C1, gene)-like 1 | -3.62 |
| A_11_P154853 | low affinity immunoglobulin gamma Fc region receptor III-like | 3.75 | A_11_P170543 |  | -3.62 |
| A_11_P000005569 |  | 3.75 | A_11_P192238 | solute carrier family 20 (phosphate transporter), member 1 | -3.61 |
| A_11_P0000040496 |  | 3.75 | A_11_P120536 | dual specificity phosphatase 6 | -3.61 |
| A_11_P090856 | major histocompatibility complex, class II, DO beta | 3.75 | A_11_P204178 |  | -3.61 |
| A_11_P149558 | chloride intracellular channel 2 | 3.75 | A_11_P0000023821 |  | -3.60 |
| A_11_P050656 | transcription factor 4 | 3.75 | A_11_P0000045 | ectonucleotide pyrophosphatase/phosphodiesterase 2 | -3.60 |
| A_11_P175183 |  | 3.75 | A_11_P0000033481 | interferon stimulated exonuclease gene 20kDa-like 2 | -3.60 |
| A_11_P0000015957 |  | 3.74 | A_11_P0000014592 |  | -3.58 |
| A_11_P102441 | ankyrin repeat domain 55 | 3.74 | A_11_P085986 | dual specificity phosphatase 6 | -3.58 |
| A_11_P0000019937 | matrix metallopeptidase 9 (gelatinase B, 92kDa gelatinase, 92kDa type IV collagenase) | 3.73 | A_11_P149138 | chromodomain protein, Y-like | -3.57 |
| A_11_P087821 | bile acid receptor-like | 3.72 | A_11_P067886 | solute carrier family 9, subfamily A (NHE8, cation proton antiporter 8), member 8 | -3.57 |
| A_11_P138726 |  | 3.72 | A_11_P091431 | phospholipase A2, group VII (platelet-activating factor acetylhydrolase, plasma) | -3.57 |
| A_11_P115296 | armadillo repeat containing, X-linked 2 | 3.72 | A_11_P051951 | alkaline phosphatase, placental | -3.57 |
| A_11_P147108 |  | 3.71 | A_11_P000003876 |  | -3.57 |
| A_11_P0000026210 | tropomyosin 2 (beta) | 3.70 | A_11_P0000039781 |  | -3.57 |
| A_11_P0000016814 | 5'-nucleotidase, ecto (CD73) | 3.69 | A_11_P0000018382 |  | -3.56 |
| A_11_P077796 | pleiotrophin | 3.69 | A_11_P0000017007 |  | -3.55 |
| A_11_P051576 | retinol binding protein 4, plasma | 3.69 | A_11_P0000025200 | chromosome 8 open reading frame, human C14orf37 | -3.55 |
| A_11_P0000027228 | plasminogen activator, tissue | 3.69 | A_11_P160308 |  | -3.54 |
| A_11_P051016 | interleukin 13 receptor, alpha 2 | 3.69 | A_11_P0000017551 |  | -3.54 |
| A_11_P0000020008 | caveolin 1, caveolae protein, 22kDa | 3.69 | A_11_P0000019847 | retinitis pigmentosa GTPase regulator | -3.54 |
| A_11_P054721 |  | 3.68 | A_11_P0000033971 |  | -3.54 |
| A_11_P0000021194 | retinoic acid receptor responder (tazarotene induced) 2 | 3.68 | A_11_P0000027609 | otopetrin 3 | -3.54 |
| A_11_P052891 | selectin P (granule membrane protein 140kDa, antigen CD62) | 3.68 | A_11_P0000026505 | PR domain containing 1, with ZNF domain | -3.54 |
| A_11_P0000028503 | epithelial membrane protein 3 | 3.68 | A_11_P168128 |  | -3.54 |
| A_11_P173983 |  | 3.67 | A_11_P0000016168 |  | -3.53 |
| A_11_P105086 | granzyme K (granzyme 3; tryptase II) | 3.67 | A_11_P157733 |  | -3.53 |
| A_11_P090746 |  | 3.67 | A_11_P067366 | tumor protein p53 inducible nuclear protein 2 | -3.52 |
| A_11_P218848 | ST6 (alpha-N-acetyl-neuraminyl-2,3-beta-galactosyl-1,3)-N-acetylgalactosaminide alpha-2,6-sialyltransferase 1 | 3.66 | A_11_P172343 |  | -3.52 |
| A_11_P199848 | fascin homolog 1, actin-bundling protein (Strongylocentrotus purpuratus) | 3.66 | A_11_P0000044 | ectonucleotide pyrophosphatase/phosphodiesterase 2 | -3.52 |
| A_11_P090656 | allograft inflammatory factor 1 | 3.66 | A_11_P0000033528 | S100 calcium binding protein A2 | -3.51 |
| A_11_P052641 | major histocompatibility complex, class II, DQ beta 1 | 3.66 | A_11_P158448 | kinesin family member 5A | -3.51 |
| A_11_P128186 | lymphoid enhancer-binding factor 1 | 3.66 | A_11_P117821 | MAX interactor 1, dimerization protein | -3.51 |
| A_11_P0000021926 | napsin A aspartic peptidase | 3.65 | A_11_P0000019738 | troponin T type 2 (cardiac) | -3.51 |
| A_11_P0000031005 | cytidine deaminase | 3.65 | A_11_P123816 |  | -3.50 |
| A_11_P0000020177 | MHC class II DR alpha chain | 3.65 | A_11_P222535 | inositol polyphosphate-5-phosphatase F | -3.49 |
| A_11_P173248 | FK506 binding protein 4, 59kDa | 3.64 | A_11_P0000017358 |  | -3.49 |
| A_11_P0000031117 | elongation factor RNA polymerase II-like 3 | 3.64 | A_11_P000006759 |  | -3.48 |
| A_11_P0000030995 | complement component 1, q subcomponent, B chain | 3.63 | A_11_P0000034169 | ectonucleoside triphosphate diphosphohydrolase 8 | -3.48 |
| A_11_P0000033141 | class II, major histocompatibility complex, transactivator | 3.63 | A_11_P00000295 | ring finger protein 213 | -3.47 |
| A_11_P060584 |  | 3.62 | A_11_P133851 |  | -3.47 |
| A_11_P079716 | transcription factor EC | 3.61 | A_11_P0000036845 |  | -3.46 |
| A_11_P078496 | family with sequence similarity 149, member A | 3.60 | A_11_P193878 |  | -3.46 |
| A_11_P185148 | complement component 1, r subcomponent | 3.60 | A_11_P082621 | solute carrier family 46 (folate transporter), member 1 | -3.45 |
| A_11_P065706 |  | 3.60 | A_11_P111651 | CD274 molecule | -3.45 |
| A_11_P0000015557 |  | 3.60 | A_11_P0000019491 |  | -3.45 |
| A_11_P0000013757 |  | 3.60 | A_11_P00000499 | interferon-induced protein with tetratricopeptide repeats 1 | -3.44 |
| A_11_P179728 |  | 3.59 | A_11_P0000033468 | delta(4)-desaturase, sphingolipid 1 | -3.44 |
| A_11_P0000020242 | MHC class Ib | 3.59 | A_11_P202638 | chymotrypsin-like | -3.44 |
| A_11_P190128 |  | 3.59 | A_11_P087551 |  | -3.44 |
| A_11_P146158 | decorin | 3.59 | A_11_P136856 | ankyrin repeat domain-containing protein 26-like | -3.43 |
| A_11_P0000031845 | membrane protein, palmitoylated 4 (MAGUK p55 subfamily member 4) | 3.59 | A_11_P000008057 |  | -3.43 |
| A_11_P176953 |  | 3.58 | A_11_P0000019238 | interleukin 15 | -3.43 |
| A_11_P0000019200 |  | 3.58 | A_11_P0000033402 | proteoglycan 4 | -3.43 |
| A_11_P105631 | transmembrane protein 218 | 3.58 | A_11_P0000032369 | dual specificity phosphatase 1 | -3.42 |
| A_11_P215008 | inducible T-cell co-stimulator | 3.58 | A_11_P128681 | P450 (cytochrome) oxidoreductase | -3.42 |
| A_11_P058156 | serpin peptidase inhibitor, clade E (nexin, plasminogen activator inhibitor type 1), member 2 | 3.58 | A_11_P000003839 |  | -3.42 |
| A_11_P053706 | toll-like receptor 2 | 3.58 | A_11_P00000441 | amyloid beta (A4) precursor protein | -3.42 |
| A_11_P0000024332 | annexin A6 | 3.58 | A_11_P0000018102 |  | -3.40 |
| A_11_P104401 | peptidylprolyl isomerase F | 3.57 | A_11_P0000039503 |  | -3.39 |
| A_11_P0000029420 | UDP-glucose glycoprotein glucosyltransferase 2 | 3.57 | A_11_P172238 |  | -3.38 |
| A_11_P071101 |  | 3.56 | A_11_P0000028797 | poly (ADP-ribose) polymerase family, member 3 | -3.37 |
| A_11_P190893 |  | 3.56 | A_11_P0000024903 | interferon-induced protein 44-like | -3.37 |
| A_11_P0000039283 | serpin peptidase inhibitor, clade H (heat shock protein 47), member 1, (collagen binding protein 1) | 3.56 | A_11_P189253 |  | -3.36 |
| A_11_P050696 | glucagon | 3.56 | A_11_P159858 |  | -3.36 |
| A_11_P054686 |  | 3.55 | A_11_P0000025366 | ORM1-like 3 (S. cerevisiae) | -3.36 |
| A_11_P198188 |  | 3.55 | A_11_P132636 |  | -3.36 |
| A_11_P0000011966 |  | 3.55 | A_11_P198288 | ras homolog family member V | -3.36 |
| A_11_P0000017318 | retinoic acid induced 2 | 3.55 | A_11_P0000034284 | DAB2 interacting protein | -3.36 |
| A_11_P0000039279 | phytanoyl-CoA hydroxylase-like | 3.54 | A_11_P135171 |  | -3.36 |
| A_11_P0000040655 |  | 3.54 | A_11_P0000029956 | endothelin converting enzyme-like 1 | -3.36 |
| A_11_P163888 | neutrophil cytosolic factor 1 | 3.54 | A_11_P0000014147 |  | -3.36 |
| A_11_P110236 | collagen and calcium binding EGF domains 1 | 3.53 | A_11_P0000014469 | angiopoietin-like 4 | -3.36 |
| A_11_P152863 |  | 3.53 | A_11_P0000020498 | solute carrier family 22 (organic cation/carnitine transporter), member 5 | -3.35 |
| A_11_P0000025419 | carbonic anhydrase IV | 3.53 | A_11_P207353 | solute carrier family 40 (iron-regulated transporter), member 1 | -3.35 |
| A_11_P053836 | IgA heavy chain constant region | 3.52 | A_11_P187853 |  | -3.34 |
| A_11_P062741 |  | 3.51 | A_11_P212348 | uncharacterized LOC100688518 | -3.34 |
| A_11_P0000015569 |  | 3.51 | A_11_P102981 | ring finger protein 19B | -3.34 |
| A_11_P0000014877 |  | 3.51 | A_11_P159668 | v-maf avian musculoaponeurotic fibrosarcoma oncogene homolog F | -3.34 |
| A_11_P110951 |  | 3.51 | A_11_P0000032977 |  | -3.34 |
| A_11_P000005379 |  | 3.51 | A_11_P0000033517 | nucleoporin 210kDa-like | -3.34 |
| A_11_P069406 | suppression of tumorigenicity 5 | 3.51 | A_11_P188953 |  | -3.34 |
| A_11_P065801 | NEL-like 2 (chicken) | 3.50 | A_11_P0000021163 | DEAD (Asp-Glu-Ala-Asp) box polypeptide 60 | -3.33 |
| A_11_P0000014606 |  | 3.50 | A_11_P0000039945 |  | -3.33 |
| A_11_P000005367 |  | 3.49 | A_11_P0000038692 |  | -3.33 |
| A_11_P0000027880 | tetraspanin 4 | 3.49 | A_11_P0000016607 | proprotein convertase subtilisin/kexin type 4 | -3.33 |
| A_11_P0000019247 |  | 3.49 | A_11_P0000029057 | SH3-domain GRB2-like 1 | -3.33 |
| A_11_P201543 | collagen, type I, alpha 2 | 3.47 | A_11_P085481 |  | -3.33 |
| A_11_P057356 | calcitonin receptor-like | 3.47 | A_11_P0000014013 |  | -3.32 |
| A_11_P0000038968 | transferrin | 3.46 | A_11_P169433 |  | -3.32 |
| A_11_P0000023235 | vimentin | 3.45 | A_11_P175388 |  | -3.32 |
| A_11_P051191 | serum amyloid A1 | 3.45 | A_11_P0000018530 |  | -3.31 |
| A_11_P138721 |  | 3.45 | A_11_P0000041702 |  | -3.31 |
| A_11_P000009080 |  | 3.45 | A_11_P0000025045 | S100 calcium binding protein A6 | -3.30 |
| A_11_P0000025562 | serum amyloid A1 | 3.44 | A_11_P0000026834 | oxysterol binding protein-like 3 | -3.30 |
| A_11_P0000025622 |  | 3.44 | A_11_P0000020471 | cysteine dioxygenase type 1 | -3.30 |
| A_11_P0000018648 |  | 3.43 | A_11_P0000018524 | ras homolog family member V | -3.29 |
| A_11_P055261 | serum amyloid A1 | 3.43 | A_11_P169273 |  | -3.29 |
| A_11_P0000030996 | complement component 1, q subcomponent, C chain | 3.42 | A_11_P0000028402 | transmembrane protein 150B | -3.28 |
| A_11_P060231 |  | 3.41 | A_11_P188943 |  | -3.28 |
| A_11_P0000016986 | filamin A interacting protein 1-like | 3.41 | A_11_P0000011179 | chemokine (C-C motif) ligand 4 | -3.28 |
| A_11_P0000039617 |  | 3.40 | A_11_P0000019893 | vascular endothelial growth factor A | -3.28 |
| A_11_P052991 | matrix metallopeptidase 2 (gelatinase A, 72kDa gelatinase, 72kDa type IV collagenase) | 3.40 | A_11_P0000015146 |  | -3.28 |
| A_11_P186593 |  | 3.40 | A_11_P100446 | CBP80/20-dependent translation initiation factor | -3.28 |
| A_11_P0000016394 | 4-hydroxy-2-oxoglutarate aldolase 1 | 3.40 | A_11_P0000030809 | heparin-binding EGF-like growth factor | -3.27 |
| A_11_P068481 | protease, serine, 23 | 3.39 | A_11_P094586 | protein Hook homolog 1-like | -3.27 |
| A_11_P060626 |  | 3.39 | A_11_P0000027958 | EH domain binding protein 1-like 1 | -3.27 |
| A_11_P051236 | collagen, type I, alpha 2 | 3.39 | A_11_P199098 | solute carrier family 39 (zinc transporter), member 4 | -3.27 |
| A_11_P178658 |  | 3.39 | A_11_P148298 | regulatory factor X, 2 (influences HLA class II expression) | -3.27 |
| A_11_P0000015602 | methionine sulfoxide reductase B3 | 3.39 | A_11_P000009009 |  | -3.27 |
| A_11_P194428 | neuron-derived neurotrophic factor | 3.38 | A_11_P163433 | ubiquitin specific peptidase 2 | -3.27 |
| A_11_P221143 | CD72 molecule | 3.38 | A_11_P055486 |  | -3.26 |
| A_11_P0000034768 | Bruton agammaglobulinemia tyrosine kinase | 3.38 | A_11_P0000039871 |  | -3.26 |
| A_11_P0000016837 |  | 3.38 | A_11_P0000026512 | forkhead box O3 | -3.26 |
| A_11_P060716 |  | 3.38 | A_11_P051636 |  | -3.26 |
| A_11_P157933 |  | 3.37 | A_11_P0000013012 |  | -3.26 |
| A_11_P146998 | GPN-loop GTPase 1 | 3.37 | A_11_P211713 |  | -3.25 |
| A_11_P0000016348 |  | 3.37 | A_11_P171663 |  | -3.25 |
| A_11_P0000034118 | unc-119 homolog (C. elegans) | 3.36 | A_11_P170388 |  | -3.25 |
| A_11_P107456 | tumor necrosis factor receptor superfamily, member 18 | 3.36 | A_11_P0000034820 | LON peptidase N-terminal domain and ring finger 3 | -3.25 |
| A_11_P185448 | SWI/SNF related, matrix associated, actin dependent regulator of chromatin, subfamily d, member 3 | 3.36 | A_11_P0000041375 |  | -3.25 |
| A_11_P115311 | brain expressed, X-linked 5 | 3.35 | A_11_P0000019726 | beta-defensin 122 | -3.24 |
| A_11_P117051 | connective tissue growth factor-like | 3.35 | A_11_P0000033299 |  | -3.24 |
| A_11_P0000024439 | guanylate cyclase 1, soluble, alpha 2 | 3.35 | A_11_P0000018147 |  | -3.24 |
| A_11_P0000034020 |  | 3.34 | A_11_P0000028869 | elongation factor RNA polymerase II | -3.24 |
| A_11_P054711 |  | 3.34 | A_11_P000001056 |  | -3.24 |
| A_11_P0000016727 |  | 3.34 | A_11_P0000028335 | bicaudal D homolog 2 (Drosophila) | -3.24 |
| A_11_P0000014535 | Bruton agammaglobulinemia tyrosine kinase | 3.33 | A_11_P0000038989 | v-src sarcoma (Schmidt-Ruppin A-2) viral oncogene homolog (avian) | -3.23 |
| A_11_P088446 | myosin, light chain 6B, alkali, smooth muscle and non-muscle | 3.33 | A_11_P190728 | spectrin repeat containing, nuclear envelope 1 | -3.23 |
| A_11_P109866 |  | 3.33 | A_11_P0000010409 |  | -3.23 |
| A_11_P0000039510 | D-amino-acid oxidase | 3.33 | A_11_P0000017308 | agrin | -3.23 |
| A_11_P0000020877 | platelet-derived growth factor receptor, alpha polypeptide | 3.33 | A_11_P084641 | FRY-like | -3.23 |
| A_11_P138911 |  | 3.32 | A_11_P124896 | myelin associated glycoprotein | -3.22 |
| A_11_P202643 | phosphatidic acid phosphatase type 2B | 3.32 | A_11_P000004586 |  | -3.22 |
| A_11_P090741 | heat shock 70kDa protein 1-like | 3.31 | A_11_P0000025901 |  | -3.22 |
| A_11_P0000015340 |  | 3.31 | A_11_P053601 | ATP-binding cassette, sub-family C (CFTR/MRP), member 2 | -3.22 |
| A_11_P222388 | CAP, adenylate cyclase-associated protein, 2 (yeast) | 3.31 | A_11_P151488 |  | -3.22 |
| A_11_P0000039672 | bone morphogenetic protein 5 | 3.31 | A_11_P148998 |  | -3.22 |
| A_11_P066126 | phosphodiesterase 6H, cGMP-specific, cone, gamma | 3.31 | A_11_P087766 |  | -3.22 |
| A_11_P0000017134 | MHC class Ib | 3.31 | A_11_P075421 | family with sequence similarity 116, member A | -3.21 |
| A_11_P0000019368 |  | 3.30 | A_11_P000007636 |  | -3.21 |
| A_11_P0000017301 |  | 3.30 | A_11_P150708 | integrin, alpha 6 | -3.21 |
| A_11_P0000018223 |  | 3.30 | A_11_P152648 |  | -3.21 |
| A_11_P0000022501 | transmembrane 4 L six family member 18 | 3.30 | A_11_P056146 | chromodomain protein, Y-like | -3.20 |
| A_11_P080311 |  | 3.30 | A_11_P0000034446 | platelet-derived growth factor alpha polypeptide | -3.20 |
| A_11_P114341 | dystrophin | 3.29 | A_11_P160423 |  | -3.20 |
| A_11_P0000040801 |  | 3.29 | A_11_P0000041591 | glycerophosphocholine phosphodiesterase GDE1 homolog (S. cerevisiae) | -3.20 |
| A_11_P0000039091 |  | 3.29 | A_11_P000001055 |  | -3.20 |
| A_11_P076591 | acid phosphatase 5, tartrate resistant | 3.29 | A_11_P0000032965 | P450 (cytochrome) oxidoreductase | -3.20 |
| A_11_P0000023046 | chromosome 28 open reading frame, human C10orf10 | 3.29 | A_11_P193753 |  | -3.20 |
| A_11_P0000033963 | FK506 binding protein 10, 65 kDa | 3.28 | A_11_P214273 |  | -3.20 |
| A_11_P209343 | 5'-nucleotidase, ecto (CD73) | 3.28 | A_11_P080756 |  | -3.20 |
| A_11_P0000019702 | Fc fragment of IgG, high affinity Ia, receptor (CD64) | 3.28 | A_11_P062791 | complexin 3 | -3.20 |
| A_11_P0000022280 | folate receptor 2 (fetal) | 3.27 | A_11_P136496 |  | -3.20 |
| A_11_P000002695 | heat shock 22kDa protein 8 | 3.27 | A_11_P218248 |  | -3.20 |
| A_11_P0000034566 | toll-like receptor 8 | 3.27 | A_11_P186968 |  | -3.19 |
| A_11_P121281 |  | 3.27 | A_11_P051846 | endothelin 3 | -3.19 |
| A_11_P201848 |  | 3.26 | A_11_P000001059 |  | -3.18 |
| A_11_P171983 | protocadherin 17 | 3.26 | A_11_P0000026624 | solute carrier family 39 (zinc transporter), member 4 | -3.18 |
| A_11_P051061 | glutathione peroxidase 1 | 3.26 | A_11_P0000011177 |  | -3.18 |
| A_11_P121731 | apolipoprotein L, 5 | 3.26 | A_11_P0000018039 |  | -3.18 |
| A_11_P0000023877 | growth factor receptor-bound protein 14 | 3.26 | A_11_P174033 | ring finger protein 103 | -3.18 |
| A_11_P051121 | toll-like receptor 4 | 3.26 | A_11_P136506 |  | -3.18 |
| A_11_P151793 |  | 3.26 | A_11_P160298 |  | -3.18 |
| A_11_P00000157 |  | 3.25 | A_11_P212243 | taxilin beta | -3.17 |
| A_11_P0000019896 | adenosine A3 receptor | 3.25 | A_11_P0000017478 |  | -3.17 |
| A_11_P0000026485 | LYR motif containing 2 | 3.24 | A_11_P000009722 |  | -3.17 |
| A_11_P0000022001 | FXYD domain containing ion transport regulator 1 | 3.24 | A_11_P0000039704 |  | -3.17 |
| A_11_P121481 |  | 3.24 | A_11_P105541 | suppression of tumorigenicity 14 (colon carcinoma) | -3.17 |
| A_11_P0000015291 |  | 3.24 | A_11_P0000041598 |  | -3.17 |
| A_11_P0000039506 |  | 3.24 | A_11_P0000018584 |  | -3.17 |
| A_11_P0000039325 | asporin | 3.24 | A_11_P0000021657 | interleukin 15 | -3.17 |
| A_11_P171853 |  | 3.24 | A_11_P0000037804 | uncharacterized LOC100688518 | -3.16 |
| A_11_P000003247 | bicaudal C homolog 1 (Drosophila) | 3.23 | A_11_P110251 | complexin 4 | -3.16 |
| A_11_P0000019960 | tissue factor pathway inhibitor (lipoprotein-associated coagulation inhibitor) | 3.23 | A_11_P205363 |  | -3.16 |
| A_11_P0000019900 | TIMP metallopeptidase inhibitor 1 | 3.23 | A_11_P130381 |  | -3.16 |
| A_11_P175683 | laminin, alpha 4 | 3.23 | A_11_P179323 |  | -3.16 |
| A_11_P054732 |  | 3.22 | A_11_P000006338 |  | -3.16 |
| A_11_P0000021930 | protein arginine methyltransferase 1 | 3.22 | A_11_P0000033560 | desmoglein 2 | -3.15 |
| A_11_P202564 | transgelin | 3.21 | A_11_P0000014432 |  | -3.15 |
| A_11_P119506 |  | 3.21 | A_11_P079446 | deafness, autosomal dominant 5 | -3.15 |
| A_11_P203808 | fibroblast growth factor 1 (acidic) | 3.21 | A_11_P173383 |  | -3.15 |
| A_11_P0000019905 | collagen, type I, alpha 2 | 3.21 | A_11_P0000010626 |  | -3.15 |
| A_11_P0000041359 | solute carrier family 12, member 8 | 3.20 | A_11_P0000032732 | 5',3'-nucleotidase, mitochondrial | -3.15 |
| A_11_P0000032127 | fibroblast growth factor-binding protein 1-like | 3.20 | A_11_P000005903 |  | -3.14 |
| A_11_P0000039557 | protease, serine, 23 | 3.20 | A_11_P185373 |  | -3.14 |
| A_11_P0000020638 |  | 3.20 | A_11_P0000028408 | EPS8-like 1 | -3.14 |
| A_11_P0000016638 |  | 3.19 | A_11_P128031 | fatty acid desaturase 2 | -3.14 |
| A_11_P157563 | arginase 1 | 3.19 | A_11_P000007547 |  | -3.14 |
| A_11_P0000016647 | claudin 2 | 3.19 | A_11_P144353 | insulin induced gene 1 | -3.14 |
| A_11_P0000035026 |  | 3.19 | A_11_P057976 |  | -3.14 |
| A_11_P077906 | retinoic acid receptor responder (tazarotene induced) 2 | 3.18 | A_11_P0000040926 |  | -3.14 |
| A_11_P0000026535 | odd-skipped related 2 (Drosophila) | 3.18 | A_11_P0000017995 |  | -3.14 |
| A_11_P000005012 | dedicator of cytokinesis 4 | 3.18 | A_11_P0000022108 |  | -3.14 |
| A_11_P0000033622 | NLR family, pyrin domain containing 3 | 3.18 | A_11_P195178 | prostate transmembrane protein, androgen induced 1 | -3.13 |
| A_11_P076046 |  | 3.18 | A_11_P000008602 |  | -3.13 |
| A_11_P162233 | argininosuccinate synthase 1 | 3.17 | A_11_P0000032010 | gamma-aminobutyric acid (GABA) A receptor, gamma 3 | -3.13 |
| A_11_P060611 |  | 3.17 | A_11_P215508 |  | -3.12 |
| A_11_P149363 |  | 3.17 | A_11_P00000609 | solute carrier family 26 (anion exchanger), member 6 | -3.12 |
| A_11_P00000129 | asporin | 3.16 | A_11_P193033 |  | -3.12 |
| A_11_P066576 | wingless-type MMTV integration site family, member 5B | 3.16 | A_11_P0000029469 | phospholipase C, delta 1 | -3.12 |
| A_11_P0000038858 | heat shock 22kDa protein 8 | 3.16 | A_11_P196413 |  | -3.12 |
| A_11_P000001202 | dual oxidase 2 | 3.16 | A_11_P066786 | kinesin family member 16B | -3.12 |
| A_11_P085876 | nucleosome assembly protein 1-like 1 | 3.16 | A_11_P0000016006 |  | -3.11 |
| A_11_P175888 | nucleosome assembly protein 1-like 1 | 3.16 | A_11_P147853 | endothelin 2 | -3.11 |
| A_11_P104976 |  | 3.16 | A_11_P137206 | protein tyrosine phosphatase, receptor type, R | -3.11 |
| A_11_P0000016223 | delta/notch-like EGF repeat containing | 3.15 | A_11_P086316 | nuclear receptor subfamily 3, group C, member 2 | -3.11 |
| A_11_P0000039652 |  | 3.15 | A_11_P0000027564 |  | -3.10 |
| A_11_P189328 |  | 3.15 | A_11_P086761 | apolipoprotein B (including Ag(x) antigen) | -3.10 |
| A_11_P0000016859 |  | 3.15 | A_11_P0000031313 | phosphodiesterase 9A | -3.10 |
| A_11_P0000030706 | carbonic anhydrase XIII | 3.15 | A_11_P206658 |  | -3.10 |
| A_11_P0000023937 | collagen, type V, alpha 2 | 3.14 | A_11_P216923 |  | -3.09 |
| A_11_P162018 | toll-like receptor 7 | 3.14 | A_11_P060006 | 2'-5'-oligoadenylate synthetase 1, 40/46kDa | -3.09 |
| A_11_P0000015751 |  | 3.14 | A_11_P080686 | ring finger protein 213 | -3.09 |
| A_11_P0000030143 | POZ (BTB) and AT hook containing zinc finger 1 | 3.14 | A_11_P000009403 |  | -3.09 |
| A_11_P0000017014 |  | 3.14 | A_11_P120431 |  | -3.09 |
| A_11_P0000034685 | melanoma antigen family H, 1 | 3.14 | A_11_P202423 |  | -3.09 |
| A_11_P00000927 | RAB34, member RAS oncogene family | 3.14 | A_11_P156143 |  | -3.09 |
| A_11_P170223 |  | 3.13 | A_11_P077581 | prolactin-induced protein | -3.08 |
| A_11_P105251 | Dab, mitogen-responsive phosphoprotein, homolog 2 (Drosophila) | 3.13 | A_11_P0000027216 | extended synaptotagmin-like protein 2 | -3.08 |
| A_11_P084236 | KH domain containing, RNA binding, signal transduction associated 3 | 3.13 | A_11_P124316 | prostate transmembrane protein, androgen induced 1 | -3.08 |
| A_11_P051196 | CD163 molecule | 3.13 | A_11_P0000040725 |  | -3.08 |
| A_11_P083521 | adenylate kinase 1 | 3.13 | A_11_P075886 | shisa family member 5 | -3.08 |
| A_11_P166948 | solute carrier family 12, member 8 | 3.12 | A_11_P211553 |  | -3.08 |
| A_11_P000002961 |  | 3.11 | A_11_P0000023859 |  | -3.08 |
| A_11_P059381 | **endomucin** | 3.11 | A_11_P200993 |  | -3.07 |
| A_11_P0000038911 |  | 3.10 | A_11_P063716 | pancreatic lipase-related protein 2 | -3.07 |
| A_11_P162768 | D-amino-acid oxidase | 3.10 | A_11_P0000040336 |  | -3.07 |
| A_11_P221303 | glutathione peroxidase 3 (plasma) | 3.10 | A_11_P0000030685 | junctophilin 1 | -3.07 |
| A_11_P166003 |  | 3.10 | A_11_P062431 | myosin IE | -3.07 |
| A_11_P0000014988 |  | 3.10 | A_11_P0000034917 |  | -3.06 |
| A_11_P0000023453 |  | 3.10 | A_11_P000002795 |  | -3.06 |
| A_11_P0000018887 | myosin, light chain 9, regulatory | 3.10 | A_11_P191123 | 5'-nucleotidase, cytosolic IB | -3.06 |
| A_11_P059921 |  | 3.10 | A_11_P189398 |  | -3.05 |
| A_11_P058891 | apolipoprotein D | 3.10 | A_11_P129271 |  | -3.05 |
| A_11_P109331 | transmembrane 6 superfamily member 1 | 3.09 | A_11_P000005705 |  | -3.05 |
| A_11_P0000015282 |  | 3.09 | A_11_P214008 |  | -3.04 |
| A_11_P0000024894 | cysteine-rich, angiogenic inducer, 61 | 3.09 | A_11_P175488 |  | -3.04 |
| A_11_P0000021447 | Lix1 homolog (mouse)-like | 3.09 | A_11_P050581 | endothelin 2 | -3.04 |
| A_11_P104386 |  | 3.09 | A_11_P0000029757 | family with sequence similarity 65, member C | -3.04 |
| A_11_P186343 |  | 3.08 | A_11_P077006 | regulatory factor X, 2 (influences HLA class II expression) | -3.04 |
| A_11_P0000015580 | caveolin 1, caveolae protein, 22kDa | 3.08 | A_11_P0000012153 |  | -3.04 |
| A_11_P050831 | ST6 (alpha-N-acetyl-neuraminyl-2,3-beta-galactosyl-1,3)-N-acetylgalactosaminide alpha-2,6-sialyltransferase 1 | 3.08 | A_11_P130536 |  | -3.04 |
| A_11_P0000028944 | RAD23 homolog A (S. cerevisiae) | 3.07 | A_11_P181698 | prostate transmembrane protein, androgen induced 1 | -3.04 |
| A_11_P126571 | glycoprotein (transmembrane) nmb | 3.07 | A_11_P0000030622 | H6 family homeobox 2 | -3.04 |
| A_11_P053821 | ceruloplasmin (ferroxidase) | 3.07 | A_11_P0000016039 |  | -3.03 |
| A_11_P161938 | LIM and cysteine-rich domains 1 | 3.07 | A_11_P137173 |  | -3.03 |
| A_11_P050631 | collagen, type I, alpha 1 | 3.07 | A_11_P0000014792 |  | -3.03 |
| A_11_P0000015134 |  | 3.07 | A_11_P214988 |  | -3.03 |
| A_11_P150858 | deoxyribonuclease II, lysosomal | 3.07 | A_11_P0000015364 |  | -3.03 |
| A_11_P0000021454 |  | 3.07 | A_11_P210874 | HECT and RLD domain containing E3 ubiquitin protein ligase 5 | -3.02 |
| A_11_P058031 |  | 3.07 | A_11_P0000040151 |  | -3.02 |
| A_11_P094186 |  | 3.06 | A_11_P051106 | BCL2-like 1 | -3.02 |
| A_11_P195723 |  | 3.06 | A_11_P0000041646 | FBJ murine osteosarcoma viral oncogene homolog | -3.01 |
| A_11_P0000021252 | deleted in liver cancer 1 | 3.06 | A_11_P000008363 |  | -3.01 |
| A_11_P0000019685 | prostaglandin E receptor 3 (subtype EP3) | 3.06 | A_11_P0000024029 |  | -3.01 |
| A_11_P000002202 | chemokine (C-X-C motif) ligand 12 | 3.05 | A_11_P053811 | laminin, beta 3 | -3.01 |
| A_11_P148543 | heat shock protein 90kDa alpha (cytosolic), class A member 1 | 3.05 | A_11_P0000032497 | suppression of tumorigenicity 14 (colon carcinoma) | -3.01 |
| A_11_P0000033287 | chloride channel accessory 1 | 3.05 | A_11_P139666 |  | -3.01 |
| A_11_P117106 | plastin 3 | 3.05 | A_11_P0000039979 |  | -3.01 |
| A_11_P107946 | WAP four-disulfide core domain 1 | 3.04 | A_11_P0000015221 |  | -3.01 |
| A_11_P054646 |  | 3.04 | A_11_P0000022655 | histamine receptor H3 | -3.01 |
| A_11_P00000209 | potassium voltage-gated channel, delayed-rectifier, subfamily S, member 3 | 3.04 | A_11_P150673 |  | -3.01 |
| A_11_P0000014276 | guanine nucleotide binding protein (G protein), gamma 11 | 3.04 | A_11_P167778 |  | -3.00 |
| A_11_P082456 | chemokine (C-C motif) ligand 14 | 3.04 | A_11_P210423 |  | -3.00 |
| A_11_P180518 |  | 3.04 | A_11_P0000012309 |  | -3.00 |
| A_11_P082361 | chromosome 9 open reading frame, human C17orf64 | 3.04 | A_11_P194493 |  | -3.00 |
| A_11_P0000030635 | snail homolog 2 (Drosophila) | 3.03 | A_11_P117516 | glycerophosphocholine phosphodiesterase GDE1 homolog (S. cerevisiae) | -2.99 |
| A_11_P191393 | transmembrane protein 176A | 3.03 | A_11_P058706 | poly (ADP-ribose) polymerase family, member 14 | -2.99 |
| A_11_P155208 | AHA1, activator of heat shock 90kDa protein ATPase homolog 1 (yeast) | 3.03 | A_11_P0000030255 | nuclear factor, erythroid 2 | -2.99 |
| A_11_P0000020815 |  | 3.03 | A_11_P000006830 |  | -2.99 |
| A_11_P0000040989 |  | 3.02 | A_11_P185673 |  | -2.99 |
| A_11_P178938 |  | 3.02 | A_11_P169713 |  | -2.99 |
| A_11_P0000029606 | retinoblastoma binding protein 9 | 3.02 | A_11_P065221 | solute carrier family 2 (facilitated glucose transporter), member 2 | -2.99 |
| A_11_P067541 | transglutaminase 2 | 3.02 | A_11_P0000025926 | chromobox homolog 6 | -2.99 |
| A_11_P0000037713 |  | 3.01 | A_11_P0000010418 |  | -2.98 |
| A_11_P175023 | apolipoprotein L, 5 | 3.01 | A_11_P000004426 |  | -2.98 |
| A_11_P059501 | coiled-coil domain containing 109B | 3.01 | A_11_P156188 |  | -2.97 |
| A_11_P051281 | sulfatase 1 | 3.01 | A_11_P0000022829 | TRAF-type zinc finger domain containing 1 | -2.97 |
| A_11_P218663 | bactericidal/permeability-increasing protein | 3.01 | A_11_P000009144 |  | -2.97 |
| A_11_P0000015535 | kallikrein 1 | 3.01 | A_11_P0000031937 |  | -2.97 |
| A_11_P177748 |  | 3.01 | A_11_P000008181 |  | -2.97 |
| A_11_P118256 |  | 3.00 | A_11_P0000029713 | lipin 3 | -2.97 |
| A_11_P0000016343 | angiotensin II receptor, type 1 | 3.00 | A_11_P107426 | agrin | -2.97 |
| A_11_P078206 | chromosome 16 open reading frame, human C8orf4 | 3.00 | A_11_P185073 |  | -2.97 |
| A_11_P0000021675 | annexin A5 | 3.00 | A_11_P204243 |  | -2.97 |
| A_11_P162178 |  | 2.99 | A_11_P0000017250 |  | -2.97 |
| A_11_P0000041940 |  | 2.99 | A_11_P159003 |  | -2.96 |
| A_11_P219278 | FAT atypical cadherin 4 | 2.99 | A_11_P062916 | mitogen-activated protein kinase 8 | -2.96 |
| A_11_P192643 | vimentin | 2.99 | A_11_P0000028726 | TatD DNase domain containing 2 | -2.96 |
| A_11_P094241 |  | 2.99 | A_11_P156033 | deltex 3-like (Drosophila) | -2.95 |
| A_11_P0000021057 |  | 2.99 | A_11_P178433 |  | -2.95 |
| A_11_P187798 |  | 2.98 | A_11_P168083 |  | -2.95 |
| A_11_P0000019677 | toll-like receptor 4 | 2.98 | A_11_P094421 | tripartite motif containing 36 | -2.94 |
| A_11_P109871 | protocadherin 7 | 2.98 | A_11_P0000030427 | apolipoprotein B mRNA editing enzyme, catalytic polypeptide 1 | -2.94 |
| A_11_P109421 | DnaJ (Hsp40) homolog, subfamily A, member 4 | 2.98 | A_11_P211563 |  | -2.94 |
| A_11_P093461 |  | 2.98 | A_11_P0000017151 |  | -2.94 |
| A_11_P204798 | double C2-like domains, beta | 2.98 | A_11_P061446 | tetratricopeptide repeat domain 3 | -2.94 |
| A_11_P054956 | heat shock 22kDa protein 8 | 2.98 | A_11_P0000027089 | Rap guanine nucleotide exchange factor (GEF) 2 | -2.93 |
| A_11_P0000032103 | carboxypeptidase Z | 2.98 | A_11_P0000020657 | chromosome 12 open reading frame, human C6orf106 | -2.93 |
| A_11_P056996 |  | 2.97 | A_11_P099736 | pre-B-cell leukemia homeobox interacting protein 1 | -2.93 |
| A_11_P0000040458 | WAP four-disulfide core domain 1 | 2.97 | A_11_P0000033786 | ELM2 and Myb/SANT-like domain containing 1 | -2.93 |
| A_11_P0000020047 | microphthalmia-associated transcription factor | 2.97 | A_11_P126511 |  | -2.93 |
| A_11_P193263 | PR domain containing 6 | 2.96 | A_11_P0000012457 |  | -2.93 |
| A_11_P0000041043 |  | 2.96 | A_11_P0000015077 |  | -2.93 |
| A_11_P086446 | family with sequence similarity 198, member B | 2.95 | A_11_P0000014262 |  | -2.93 |
| A_11_P200708 |  | 2.95 | A_11_P103371 | interferon, lambda receptor 1 | -2.92 |
| A_11_P0000040517 | colony stimulating factor 1 receptor | 2.95 | A_11_P0000033557 | meprin A, beta | -2.92 |
| A_11_P115726 |  | 2.95 | A_11_P0000011064 |  | -2.92 |
| A_11_P0000031531 | lysophosphatidylcholine acyltransferase 1 | 2.95 | A_11_P0000012883 |  | -2.92 |
| A_11_P000003275 | kelch-like family member 5 | 2.95 | A_11_P0000039792 |  | -2.92 |
| A_11_P166633 |  | 2.95 | A_11_P225563 | tripartite motif containing 29 | -2.92 |
| A_11_P0000025581 | THO complex 3 | 2.95 | A_11_P197408 | dual specificity phosphatase 16 | -2.92 |
| A_11_P0000023903 | histone acetyltransferase 1 | 2.95 | A_11_P000004108 |  | -2.92 |
| A_11_P0000034698 | V-set and immunoglobulin domain containing 4 | 2.95 | A_11_P058466 |  | -2.92 |
| A_11_P075166 | LIM and cysteine-rich domains 1 | 2.94 | A_11_P061886 | Bcl2 modifying factor | -2.92 |
| A_11_P179813 |  | 2.94 | A_11_P0000013969 |  | -2.91 |
| A_11_P0000040623 | retinoic acid receptor responder (tazarotene induced) 1 | 2.94 | A_11_P171053 |  | -2.91 |
| A_11_P094216 |  | 2.93 | A_11_P000005905 |  | -2.91 |
| A_11_P189848 |  | 2.93 | A_11_P000009978 |  | -2.91 |
| A_11_P205483 |  | 2.93 | A_11_P080691 | ring finger protein 213 | -2.91 |
| A_11_P00000299 | ClpB caseinolytic peptidase B homolog (E. coli) | 2.93 | A_11_P185028 | transforming, acidic coiled-coil containing protein 2 | -2.91 |
| A_11_P055551 | regulator of G-protein signaling 18 | 2.92 | A_11_P0000079 | fructose-1,6-bisphosphatase 1 | -2.90 |
| A_11_P0000022949 | NEL-like 2 (chicken) | 2.92 | A_11_P0000017606 |  | -2.90 |
| A_11_P0000032449 | insulin gene enhancer protein ISL-1-like | 2.91 | A_11_P181238 | lectin, galactoside-binding, soluble, 8 | -2.90 |
| A_11_P102216 |  | 2.91 | A_11_P0000034159 | olfactory receptor family 1 subfamily E | -2.90 |
| A_11_P0000040439 | protein disulfide isomerase family A, member 6 | 2.91 | A_11_P0000033941 | Rho family GTPase 2 | -2.90 |
| A_11_P080981 | CD300c molecule | 2.91 | A_11_P0000029557 | RAS p21 protein activator 2 | -2.90 |
| A_11_P166378 |  | 2.91 | A_11_P052871 | laminin, beta 3 | -2.90 |
| A_11_P0000025789 | filamin A, alpha | 2.91 | A_11_P0000020861 |  | -2.90 |
| A_11_P198778 |  | 2.91 | A_11_P0000025358 | junction plakoglobin | -2.90 |
| A_11_P0000019529 |  | 2.91 | A_11_P0000026042 | lectin, galactoside-binding-like | -2.90 |
| A_11_P085781 | ribonuclease, RNase A family, 4 | 2.91 | A_11_P070851 |  | -2.90 |
| A_11_P121626 | L-3-hydroxyproline dehydratase (trans-) | 2.91 | A_11_P0000015050 |  | -2.89 |
| A_11_P0000019790 | caveolin 2 | 2.90 | A_11_P191358 |  | -2.89 |
| A_11_P060751 |  | 2.90 | A_11_P0000031515 | catenin (cadherin-associated protein), delta 2 | -2.89 |
| A_11_P172868 | FK506 binding protein 4, 59kDa | 2.90 | A_11_P128901 |  | -2.89 |
| A_11_P143073 | apolipoprotein C-I | 2.90 | A_11_P0000032305 | phospholipase A2, group XIIB | -2.89 |
| A_11_P097076 | myosin, heavy chain 11, smooth muscle | 2.90 | A_11_P150258 |  | -2.89 |
| A_11_P0000022878 |  | 2.90 | A_11_P086426 | lecithin retinol acyltransferase (phosphatidylcholine--retinol O-acyltransferase) | -2.89 |
| A_11_P212218 |  | 2.90 | A_11_P0000017753 |  | -2.89 |
| A_11_P00000442 | Meis homeobox 2 | 2.90 | A_11_P156163 | synaptopodin 2-like | -2.89 |
| A_11_P119276 |  | 2.89 | A_11_P105836 | tripartite motif containing 29 | -2.88 |
| A_11_P066301 | alpha-2-macroglobulin | 2.89 | A_11_P0000013925 |  | -2.88 |
| A_11_P0000013279 |  | 2.89 | A_11_P000009759 |  | -2.88 |
| A_11_P118471 | WAP four-disulfide core domain 2 | 2.88 | A_11_P0000033290 | B-cell CLL/lymphoma 10 | -2.88 |
| A_11_P180308 | junctional adhesion molecule 2 | 2.88 | A_11_P000003081 |  | -2.88 |
| A_11_P081061 |  | 2.88 | A_11_P125761 | diacylglycerol O-acyltransferase 2 | -2.88 |
| A_11_P060571 | uncharacterized LOC612122 | 2.87 | A_11_P0000031615 | SKI-like oncogene | -2.88 |
| A_11_P0000023062 | retinol binding protein 4, plasma | 2.87 | A_11_P0000027097 | probetacellulin-like | -2.88 |
| A_11_P0000033532 | S100 calcium binding protein A9 | 2.87 | A_11_P000006588 |  | -2.88 |
| A_11_P0000021058 | spermatogenesis associated 6 | 2.87 | A_11_P0000025859 | protein tyrosine phosphatase, receptor type, R | -2.88 |
| A_11_P174363 |  | 2.86 | A_11_P068461 | folate hydrolase (prostate-specific membrane antigen) 1 | -2.87 |
| A_11_P133746 | protease, serine, 23 | 2.86 | A_11_P217583 | ER membrane protein complex subunit 1 | -2.87 |
| A_11_P172033 |  | 2.86 | A_11_P0000041156 |  | -2.87 |
| A_11_P0000039236 | protocadherin alpha 5 | 2.86 | A_11_P154498 |  | -2.87 |
| A_11_P140156 |  | 2.86 | A_11_P0000022938 | coiled-coil domain containing 65 | -2.87 |
| A_11_P0000039433 | integrin, alpha M (complement component 3 receptor 3 subunit) | 2.86 | A_11_P0000040946 |  | -2.86 |
| A_11_P0000021862 | phosphoglucomutase 5 | 2.86 | A_11_P0000013305 |  | -2.86 |
| A_11_P0000028805 | mesencephalic astrocyte-derived neurotrophic factor | 2.85 | A_11_P0000013522 |  | -2.86 |
| A_11_P122326 |  | 2.85 | A_11_P0000041853 |  | -2.86 |
| A_11_P094231 |  | 2.85 | A_11_P0000040068 |  | -2.86 |
| A_11_P054606 | Ig lambda chain V-I region BL2-like | 2.85 | A_11_P0000023646 | HECT and RLD domain containing E3 ubiquitin protein ligase 3 | -2.85 |
| A_11_P000002562 |  | 2.84 | A_11_P052194 |  | -2.85 |
| A_11_P0000020717 | alcohol dehydrogenase 5 (class III), chi polypeptide | 2.84 | A_11_P0000027390 | potassium voltage-gated channel, subfamily G, member 3 | -2.85 |
| A_11_P0000032429 | colony stimulating factor 1 receptor | 2.84 | A_11_P0000012462 |  | -2.85 |
| A_11_P054282 |  | 2.84 | A_11_P083426 |  | -2.85 |
| A_11_P0000016233 | FK506 binding protein 1B, 12.6 kDa | 2.83 | A_11_P133131 | choline/ethanolamine phosphotransferase 1 | -2.85 |
| A_11_P0000025474 | serpin peptidase inhibitor, clade F (alpha-2 antiplasmin, pigment epithelium derived factor), member 1 | 2.83 | A_11_P0000012034 |  | -2.85 |
| A_11_P099191 | coiled-coil domain containing 181 | 2.83 | A_11_P0000030827 | protocadherin 1 | -2.85 |
| A_11_P000005819 | GULP, engulfment adaptor PTB domain containing 1 | 2.83 | A_11_P000008858 |  | -2.85 |
| A_11_P0000028474 | reticulocalbin 3, EF-hand calcium binding domain | 2.83 | A_11_P211498 | laminin, beta 3 | -2.85 |
| A_11_P184718 |  | 2.82 | A_11_P0000041733 |  | -2.84 |
| A_11_P109351 | SH3-domain GRB2-like 3 | 2.82 | A_11_P207788 | spermatogenesis associated, serine-rich 2-like | -2.84 |
| A_11_P181168 |  | 2.82 | A_11_P092381 |  | -2.84 |
| A_11_P180523 |  | 2.82 | A_11_P208293 |  | -2.84 |
| A_11_P148528 | non-specific cytotoxic cell receptor protein 1 homolog (zebrafish) | 2.82 | A_11_P00000740 | endothelin converting enzyme 1 | -2.84 |
| A_11_P0000029830 | SMAD family member 9 | 2.82 | A_11_P080721 | ectonucleotide pyrophosphatase/phosphodiesterase 7 | -2.84 |
| A_11_P00000984 | adenylate kinase 1 | 2.82 | A_11_P0000025427 | chemokine (C-C motif) ligand 3 | -2.84 |
| A_11_P148283 |  | 2.82 | A_11_P168503 |  | -2.84 |
| A_11_P186393 |  | 2.81 | A_11_P152048 |  | -2.84 |
| A_11_P054596 |  | 2.81 | A_11_P110321 | coiled-coil domain containing 68 | -2.84 |
| A_11_P0000022913 |  | 2.81 | A_11_P189813 |  | -2.84 |
| A_11_P0000023184 | stathmin-like 2 | 2.81 | A_11_P204133 | RNA binding motif protein 4 | -2.84 |
| A_11_P0000015005 | protein tyrosine phosphatase, receptor type, O | 2.81 | A_11_P0000011744 |  | -2.83 |
| A_11_P0000021670 | heat shock 70kDa protein 4-like | 2.81 | A_11_P0000025096 |  | -2.83 |
| A_11_P000001494 | collagen, type VI, alpha 1 | 2.80 | A_11_P00000304 | UBA domain containing 2 | -2.83 |
| A_11_P0000030654 | carbonic anhydrase VIII | 2.80 | A_11_P093296 | KIAA0247 ortholog | -2.83 |
| A_11_P175098 |  | 2.80 | A_11_P084936 | epiregulin | -2.83 |
| A_11_P0000016210 | collagen, type VI, alpha 3 | 2.80 | A_11_P205743 | solute carrier family 32 (GABA vesicular transporter), member 1 | -2.83 |
| A_11_P171653 |  | 2.80 | A_11_P170263 | suppressor of Ty 5 homolog (S. cerevisiae) | -2.83 |
| A_11_P137316 | transgelin | 2.79 | A_11_P000006360 |  | -2.83 |
| A_11_P0000031247 | protein BTG3-like | 2.79 | A_11_P061951 | delta-like 4 (Drosophila) | -2.83 |
| A_11_P080086 |  | 2.79 | A_11_P134721 |  | -2.83 |
| A_11_P061216 | junctional adhesion molecule 2 | 2.79 | A_11_P199633 |  | -2.83 |
| A_11_P0000029151 | RAB38, member RAS oncogene family | 2.79 | A_11_P0000019657 |  | -2.83 |
| A_11_P077101 | Epstein-Barr virus induced 3 | 2.79 | A_11_P000001138 |  | -2.83 |
| A_11_P123411 | cysteine and glycine-rich protein 1-like | 2.79 | A_11_P000006770 |  | -2.83 |
| A_11_P0000030940 | lysosomal protein transmembrane 5 | 2.79 | A_11_P190943 |  | -2.82 |
| A_11_P0000034704 | acyl-CoA wax alcohol acyltransferase 2 | 2.79 | A_11_P124811 | transmembrane protein 38B | -2.82 |
| A_11_P0000023774 | mitochondrial ribosomal protein L47 | 2.79 | A_11_P0000023938 | solute carrier family 40 (iron-regulated transporter), member 1 | -2.82 |
| A_11_P116486 | KH domain containing, RNA binding, signal transduction associated 3 | 2.78 | A_11_P077326 | proprotein convertase subtilisin/kexin type 4 | -2.82 |
| A_11_P0000041507 | cysteine and glycine-rich protein 1-like | 2.78 | A_11_P165468 |  | -2.82 |
| A_11_P0000041630 | regulator of G-protein signaling 16 | 2.78 | A_11_P199573 |  | -2.82 |
| A_11_P0000020541 | TEK tyrosine kinase, endothelial | 2.78 | A_11_P205453 |  | -2.82 |
| A_11_P0000026898 | leucine proline-enriched proteoglycan (leprecan) 1 | 2.78 | A_11_P000001058 | TCR gamma alternate reading frame protein | -2.82 |
| A_11_P0000021007 |  | 2.78 | A_11_P0000030527 | tolloid-like 2 | -2.82 |
| A_11_P101211 |  | 2.78 | A_11_P0000039359 |  | -2.81 |
| A_11_P0000020311 | GLI pathogenesis-related 1 | 2.78 | A_11_P0000010943 |  | -2.81 |
| A_11_P0000022319 |  | 2.78 | A_11_P0000011110 |  | -2.81 |
| A_11_P0000039104 |  | 2.78 | A_11_P0000017578 |  | -2.81 |
| A_11_P0000020009 | chemokine (C-C motif) ligand 2 | 2.77 | A_11_P138356 |  | -2.81 |
| A_11_P077926 | transmembrane protein 176B | 2.77 | A_11_P000004928 |  | -2.80 |
| A_11_P0000019805 | TIMP metallopeptidase inhibitor 2 | 2.77 | A_11_P000008402 |  | -2.80 |
| A_11_P140346 |  | 2.77 | A_11_P077596 | transient receptor potential cation channel, subfamily V, member 6 | -2.80 |
| A_11_P095261 | DnaJ (Hsp40) homolog, subfamily B, member 5 | 2.76 | A_11_P000003799 |  | -2.80 |
| A_11_P193023 | protein kinase, cGMP-dependent, type I | 2.76 | A_11_P0000034459 |  | -2.80 |
| A_11_P120791 | reelin | 2.76 | A_11_P0000022687 | furry homolog (Drosophila) | -2.80 |
| A_11_P184733 | complement component 1, q subcomponent, C chain | 2.76 | A_11_P185528 |  | -2.80 |
| A_11_P0000019292 | dihydropyrimidinase-like 3 | 2.76 | A_11_P000005407 |  | -2.79 |
| A_11_P125721 |  | 2.76 | A_11_P099851 |  | -2.79 |
| A_11_P0000041288 | latrophilin 2 | 2.76 | A_11_P0000032062 | furin (paired basic amino acid cleaving enzyme) | -2.79 |
| A_11_P117811 | multiple PDZ domain protein | 2.76 | A_11_P0000032445 | ADP-ribosylation factor-like 15 | -2.79 |
| A_11_P0000027453 | dysferlin, limb girdle muscular dystrophy 2B (autosomal recessive) | 2.76 | A_11_P0000022042 |  | -2.79 |
| A_11_P0000017498 |  | 2.75 | A_11_P160753 | fructose-1,6-bisphosphatase 1 | -2.79 |
| A_11_P178353 | immunoglobulin J polypeptide, linker protein for immunoglobulin alpha and mu polypeptides | 2.75 | A_11_P214858 |  | -2.78 |
| A_11_P189818 | GLI pathogenesis-related 1 | 2.75 | A_11_P200418 |  | -2.78 |
| A_11_P107296 | phosphatidic acid phosphatase type 2B | 2.74 | A_11_P193238 |  | -2.78 |
| A_11_P0000010954 |  | 2.74 | A_11_P0000016601 |  | -2.78 |
| A_11_P0000040546 |  | 2.74 | A_11_P090541 | ectonucleotide pyrophosphatase/phosphodiesterase 3 | -2.78 |
| A_11_P194118 | lysophosphatidylcholine acyltransferase 1 | 2.74 | A_11_P051621 | ATP-binding cassette, sub-family C (CFTR/MRP), member 5 | -2.78 |
| A_11_P0000022406 |  | 2.74 | A_11_P063666 | transcription factor 7-like 2 (T-cell specific, HMG-box) | -2.78 |
| A_11_P0000025508 | adenylate kinase 8 | 2.74 | A_11_P169958 |  | -2.78 |
| A_11_P0000029966 | ADP-ribosylation factor-like 4C | 2.74 | A_11_P000006529 |  | -2.78 |
| A_11_P062681 | progestin and adipoQ receptor family member V | 2.74 | A_11_P065457 | keratin 5 | -2.78 |
| A_11_P079241 | asparagine synthetase [glutamine-hydrolyzing]-like | 2.74 | A_11_P183073 | tripartite motif containing 36 | -2.77 |
| A_11_P078561 | MLF1 interacting protein | 2.73 | A_11_P0000026372 | potassium channel, subfamily K, member 5 | -2.77 |
| A_11_P119541 | kelch-like family member 26 | 2.73 | A_11_P102656 | FCH domain only 2 | -2.77 |
| A_11_P072016 | calmegin | 2.73 | A_11_P095621 | lysophosphatidic acid receptor 1 | -2.77 |
| A_11_P0000030339 | adhesion molecule with Ig-like domain 2 | 2.73 | A_11_P0000019736 | chemokine (C-C motif) ligand 5 | -2.77 |
| A_11_P171573 | calponin 3, acidic | 2.73 | A_11_P164018 | dual specificity phosphatase 16 | -2.77 |
| A_11_P0000016896 | TBC1 domain family, member 4 | 2.73 | A_11_P089976 | ankyrin repeat domain 23 | -2.77 |
| A_11_P091766 | interleukin-1 receptor-associated kinase 1 binding protein 1 | 2.72 | A_11_P188283 |  | -2.77 |
| A_11_P0000025530 | adenylate kinase 1 | 2.72 | A_11_P0000038699 | kinesin family member 16B | -2.77 |
| A_11_P0000038585 |  | 2.72 | A_11_P175353 | P450 (cytochrome) oxidoreductase | -2.77 |
| A_11_P0000019708 | CD40 molecule, TNF receptor superfamily member 5 | 2.72 | A_11_P0000041129 |  | -2.77 |
| A_11_P117766 | FAT atypical cadherin 4 | 2.72 | A_11_P0000015509 | patatin-like phospholipase domain containing 2 | -2.77 |
| A_11_P0000040223 | STEAP family member 2, metalloreductase | 2.72 | A_11_P0000034051 | ATP-binding cassette, sub-family C (CFTR/MRP), member 3 | -2.77 |
| A_11_P057566 | phospholipase C-like 1 | 2.72 | A_11_P084646 |  | -2.77 |
| A_11_P0000025044 |  | 2.72 | A_11_P000006259 |  | -2.76 |
| A_11_P060656 |  | 2.72 | A_11_P000009301 |  | -2.76 |
| A_11_P0000041980 | zinc finger, CCHC domain containing 12 | 2.72 | A_11_P0000027421 |  | -2.76 |
| A_11_P0000026810 | anterior gradient 2 | 2.72 | A_11_P0000040595 | component of oligomeric golgi complex 1 | -2.76 |
| A_11_P169253 | A kinase (PRKA) anchor protein 12 | 2.72 | A_11_P103301 | aurora kinase A and ninein interacting protein | -2.76 |
| A_11_P0000021337 |  | 2.72 | A_11_P0000026771 | ATP-binding cassette, sub-family B (MDR/TAP), member 4 | -2.76 |
| A_11_P0000039318 | lectin, galactoside-binding, soluble, 1 | 2.72 | A_11_P194778 |  | -2.76 |
| A_11_P123001 | FK506 binding protein 4, 59kDa | 2.71 | A_11_P094646 | solute carrier family 22 (organic cation/zwitterion transporter), member 4 | -2.76 |
| A_11_P104971 | secreted protein, acidic, cysteine-rich (osteonectin) | 2.71 | A_11_P217988 |  | -2.76 |
| A_11_P190043 |  | 2.71 | A_11_P160983 |  | -2.76 |
| A_11_P172533 |  | 2.71 | A_11_P188468 | tubulin tyrosine ligase-like family, member 4 | -2.76 |
| A_11_P067811 | phospholipid transfer protein | 2.70 | A_11_P052921 | desmocollin 2 | -2.75 |
| A_11_P116396 | collagen, type IV, alpha 1 | 2.70 | A_11_P0000025004 | kynurenine 3-monooxygenase (kynurenine 3-hydroxylase) | -2.75 |
| A_11_P138916 |  | 2.70 | A_11_P136061 |  | -2.75 |
| A_11_P073766 | damage-specific DNA binding protein 2, 48kDa | 2.70 | A_11_P0000028299 | family with sequence similarity 108, member B1 | -2.75 |
| A_11_P182663 | retinol binding protein 4, plasma | 2.70 | A_11_P075316 | kelch repeat and BTB (POZ) domain containing 8 | -2.75 |
| A_11_P070466 | ADAM-like, decysin 1 | 2.70 | A_11_P0000040255 |  | -2.75 |
| A_11_P0000023442 |  | 2.69 | A_11_P177373 |  | -2.75 |
| A_11_P123486 | signal peptide, CUB domain, EGF-like 3 | 2.69 | A_11_P0000030226 | N-acylsphingosine amidohydrolase (non-lysosomal ceramidase) 2B | -2.75 |
| A_11_P062356 |  | 2.69 | A_11_P000003 | DnaJ (Hsp40) homolog, subfamily C, member 14 | -2.75 |
| A_11_P0000021669 | heat shock 70kDa protein 4-like | 2.69 | A_11_P0000031544 |  | -2.75 |
| A_11_P000003692 |  | 2.69 | A_11_P0000016557 | 3'-phosphoadenosine 5'-phosphosulfate synthase 2 | -2.75 |
| A_11_P0000015383 |  | 2.69 | A_11_P0000025066 | desmocollin 2 | -2.75 |
| A_11_P063466 | ADP-ribosylation factor-like 3 | 2.69 | A_11_P0000024507 | sterol regulatory element binding transcription factor 1 | -2.75 |
| A_11_P098996 |  | 2.69 | A_11_P081676 | keratin 20 | -2.75 |
| A_11_P0000015006 |  | 2.69 | A_11_P091031 | chromosome 12 open reading frame, human C6orf222 | -2.74 |
| A_11_P0000032720 | microfibrillar-associated protein 4 | 2.69 | A_11_P0000011418 |  | -2.74 |
| A_11_P0000027151 |  | 2.68 | A_11_P0000013256 |  | -2.74 |
| A_11_P176163 |  | 2.68 | A_11_P053761 | junction plakoglobin | -2.74 |
| A_11_P0000020188 | tachykinin receptor 2 | 2.68 | A_11_P0000020147 | reticulon 1 | -2.74 |
| A_11_P0000025231 | SPARC related modular calcium binding 1 | 2.68 | A_11_P187858 |  | -2.74 |
| A_11_P176903 |  | 2.68 | A_11_P198343 |  | -2.74 |
| A_11_P067041 | signal-regulatory protein alpha | 2.68 | A_11_P171993 | spermatogenesis associated, serine-rich 2-like | -2.74 |
| A_11_P051981 | ceruloplasmin (ferroxidase) | 2.68 | A_11_P192748 |  | -2.74 |
| A_11_P0000024112 | transmembrane 6 superfamily member 1 | 2.68 | A_11_P052281 | v-raf murine sarcoma viral oncogene homolog B1 | -2.74 |
| A_11_P098286 | nexilin (F actin binding protein) | 2.68 | A_11_P000008161 |  | -2.74 |
| A_11_P190383 | complement component 1, q subcomponent binding protein | 2.68 | A_11_P000008848 |  | -2.74 |
| A_11_P0000015228 |  | 2.68 | A_11_P000002303 |  | -2.74 |
| A_11_P0000034628 | cytochrome b-245, beta polypeptide (chronic granulomatous disease) | 2.68 | A_11_P170673 | junction plakoglobin | -2.74 |
| A_11_P076551 | deoxyribonuclease II, lysosomal | 2.68 | A_11_P066206 |  | -2.73 |
| A_11_P052676 | HLA class II histocompatibility antigen, DO alpha chain-like | 2.68 | A_11_P178253 |  | -2.73 |
| A_11_P0000017722 | caldesmon 1 | 2.68 | A_11_P113206 | pleckstrin homology-like domain, family B, member 3 | -2.73 |
| A_11_P0000022912 | actin, alpha 2, smooth muscle, aorta | 2.67 | A_11_P000004226 |  | -2.73 |
| A_11_P0000040170 | matrilin 2 | 2.67 | A_11_P0000031879 | cytochrome P450, family 27, subfamily A, polypeptide 1 | -2.73 |
| A_11_P212098 | transmembrane protein 47 | 2.67 | A_11_P000008377 |  | -2.73 |
| A_11_P116361 | transforming growth factor, beta receptor II (70/80kDa) | 2.67 | A_11_P0000027069 | doublecortin-like kinase 2 | -2.73 |
| A_11_P198953 | A kinase (PRKA) anchor protein 2 | 2.67 | A_11_P0000015118 |  | -2.73 |
| A_11_P0000033335 | neuron navigator 1 | 2.67 | A_11_P0000011343 |  | -2.73 |
| A_11_P207478 |  | 2.67 | A_11_P079451 | chromosome 14 open reading frame, human C7orf31 | -2.72 |
| A_11_P179153 | regulator of G-protein signaling 4 | 2.67 | A_11_P069696 | growth arrest-specific 2 | -2.72 |
| A_11_P082791 | nucleoredoxin | 2.67 | A_11_P189788 | mindbomb E3 ubiquitin protein ligase 1 | -2.72 |
| A_11_P0000030955 | replication protein A2, 32kDa | 2.67 | A_11_P074561 | phospholipase A2, group XVI-like | -2.72 |
| A_11_P0000033887 |  | 2.66 | A_11_P0000021390 | ring finger protein 103 | -2.72 |
| A_11_P0000017755 |  | 2.66 | A_11_P0000023904 | integrin, alpha 6 | -2.72 |
| A_11_P0000025334 | HIG1 hypoxia inducible domain family, member 1B | 2.66 | A_11_P0000030413 | dual specificity phosphatase 16 | -2.72 |
| A_11_P0000032632 | chromosome 5 open reading frame, human C11orf70 | 2.66 | A_11_P000003237 |  | -2.72 |
| A_11_P0000034081 | chromosome 9 open reading frame, human C17orf64 | 2.66 | A_11_P111281 | fructose-1,6-bisphosphatase 1 | -2.72 |
| A_11_P225203 | tumor necrosis factor (ligand) superfamily, member 9 | 2.66 | A_11_P000003112 |  | -2.72 |
| A_11_P196068 |  | 2.66 | A_11_P057581 | spermatogenesis associated, serine-rich 2-like | -2.72 |
| A_11_P0000020753 | serine/arginine-rich splicing factor 12 | 2.66 | A_11_P000007494 |  | -2.71 |
| A_11_P168688 |  | 2.65 | A_11_P0000017505 | paired related homeobox 1 | -2.71 |
| A_11_P093391 | acyl-CoA thioesterase 6 | 2.65 | A_11_P0000028021 | von Willebrand factor C and EGF domains | -2.71 |
| A_11_P0000017392 |  | 2.65 | A_11_P0000017299 |  | -2.71 |
| A_11_P0000020841 | KH domain containing, RNA binding, signal transduction associated 3 | 2.65 | A_11_P0000010199 |  | -2.71 |
| A_11_P087721 | sepiapterin reductase (7,8-dihydrobiopterin:NADP+ oxidoreductase) | 2.65 | A_11_P097361 | chromosome 6 open reading frame, human C16orf5 | -2.71 |
| A_11_P0000033648 | CCAAT/enhancer binding protein (C/EBP), epsilon | 2.65 | A_11_P090336 | lectin, galactoside-binding-like | -2.71 |
| A_11_P0000040818 |  | 2.65 | A_11_P0000021739 | suppressor of cytokine signaling 6-like | -2.70 |
| A_11_P0000021203 | SWI/SNF related, matrix associated, actin dependent regulator of chromatin, subfamily d, member 3 | 2.65 | A_11_P059236 | HECT and RLD domain containing E3 ubiquitin protein ligase 5 | -2.70 |
| A_11_P197298 | family with sequence similarity 149, member A | 2.65 | A_11_P0000010537 |  | -2.70 |
| A_11_P0000039528 | phosphoserine phosphatase | 2.65 | A_11_P139506 | sperm antigen with calponin homology and coiled-coil domains 1 | -2.70 |
| A_11_P095036 | protein tyrosine phosphatase-like A domain containing 2 | 2.65 | A_11_P182553 |  | -2.70 |
| A_11_P0000022755 | phosphotyrosine interaction domain containing 1 | 2.65 | A_11_P193338 |  | -2.70 |
| A_11_P0000022972 | Ras association (RalGDS/AF-6) domain family (N-terminal) member 8 | 2.64 | A_11_P108436 | F-box and leucine-rich repeat protein 8 | -2.70 |
| A_11_P091191 |  | 2.64 | A_11_P0000013633 |  | -2.70 |
| A_11_P000001761 | immunoglobulin J polypeptide, linker protein for immunoglobulin alpha and mu polypeptides | 2.64 | A_11_P155538 |  | -2.70 |
| A_11_P153108 | apolipoprotein E | 2.64 | A_11_P099921 | solute carrier family 14 (urea transporter), member 2 | -2.70 |
| A_11_P153523 |  | 2.63 | A_11_P171793 |  | -2.70 |
| A_11_P000003976 |  | 2.63 | A_11_P122181 |  | -2.70 |
| A_11_P0000020650 | HLA class II histocompatibility antigen, DO alpha chain-like | 2.63 | A_11_P054771 |  | -2.70 |
| A_11_P110811 | t-complex 1 | 2.63 | A_11_P0000026065 | aspartic peptidase, retroviral-like 1 | -2.69 |
| A_11_P0000016326 |  | 2.63 | A_11_P0000022838 | mediator complex subunit 13-like | -2.69 |
| A_11_P100296 |  | 2.63 | A_11_P0000019773 | uncoupling protein 2 (mitochondrial, proton carrier) | -2.69 |
| A_11_P000006300 | caldesmon 1 | 2.63 | A_11_P0000018780 |  | -2.69 |
| A_11_P0000030457 | neurotrophin 3 | 2.62 | A_11_P0000010396 |  | -2.69 |
| A_11_P0000035500 |  | 2.62 | A_11_P183778 |  | -2.69 |
| A_11_P088621 | methyltransferase like 1 | 2.62 | A_11_P186453 | mitogen-activated protein kinase 6 | -2.69 |
| A_11_P0000029629 | CDP-diacylglycerol synthase (phosphatidate cytidylyltransferase) 2 | 2.62 | A_11_P080226 |  | -2.69 |
| A_11_P153703 |  | 2.62 | A_11_P214623 | chymotrypsin-like | -2.69 |
| A_11_P096031 | ORAI calcium release-activated calcium modulator 2 | 2.62 | A_11_P0000015651 |  | -2.69 |
| A_11_P154123 |  | 2.62 | A_11_P0000011475 |  | -2.69 |
| A_11_P0000016551 | solute carrier family 15 (oligopeptide transporter), member 2 | 2.62 | A_11_P0000040841 | solute carrier family 35 (UDP-GlcNAc/UDP-glucose transporter), member D2 | -2.68 |
| A_11_P0000036943 | CAP, adenylate cyclase-associated protein, 2 (yeast) | 2.62 | A_11_P0000015863 | extended synaptotagmin-like protein 2 | -2.68 |
| A_11_P078301 | neuregulin 1 | 2.62 | A_11_P134056 |  | -2.68 |
| A_11_P130921 | phosphatidic acid phosphatase type 2B | 2.61 | A_11_P157703 | solute carrier family 6 (neutral amino acid transporter), member 19 | -2.68 |
| A_11_P0000020446 | chaperonin containing TCP1, subunit 4 (delta) | 2.61 | A_11_P0000041670 |  | -2.68 |
| A_11_P058961 | chemokine (C-X-C motif) ligand 10 | 2.61 | A_11_P176423 |  | -2.68 |
| A_11_P115341 | G protein-coupled receptor associated sorting protein 2 | 2.60 | A_11_P000001139 |  | -2.68 |
| A_11_P198258 |  | 2.60 | A_11_P186323 | integrin, alpha 6 | -2.68 |
| A_11_P066286 |  | 2.60 | A_11_P0000040239 |  | -2.68 |
| A_11_P0000036403 | platelet derived growth factor D | 2.60 | A_11_P0000014518 | UDP-glucose ceramide glucosyltransferase | -2.68 |
| A_11_P190118 | poly (ADP-ribose) polymerase 1 | 2.59 | A_11_P0000010619 |  | -2.67 |
| A_11_P0000018413 |  | 2.59 | A_11_P0000036400 |  | -2.67 |
| A_11_P184983 | interleukin 13 receptor, alpha 2 | 2.59 | A_11_P0000013465 |  | -2.67 |
| A_11_P000004074 |  | 2.59 | A_11_P130951 |  | -2.67 |
| A_11_P0000019938 | podoplanin | 2.59 | A_11_P0000022973 | intermediate filament tail domain containing 1 | -2.67 |
| A_11_P0000015913 | syntaxin binding protein 6 (amisyn) | 2.59 | A_11_P000004482 |  | -2.67 |
| A_11_P0000017181 | coiled-coil domain containing 146 | 2.59 | A_11_P000002392 |  | -2.67 |
| A_11_P0000026672 | kinase insert domain receptor (a type III receptor tyrosine kinase) | 2.59 | A_11_P0000039362 | junction plakoglobin | -2.67 |
| A_11_P0000026839 | homeobox A2 | 2.59 | A_11_P0000034181 | ectonucleoside triphosphate diphosphohydrolase 2 | -2.67 |
| A_11_P090651 |  | 2.59 | A_11_P155723 |  | -2.67 |
| A_11_P155268 |  | 2.59 | A_11_P173158 |  | -2.67 |
| A_11_P000005840 | actin filament associated protein 1-like 1 | 2.58 | A_11_P0000040275 |  | -2.67 |
| A_11_P054218 |  | 2.58 | A_11_P149498 |  | -2.67 |
| A_11_P137026 | actin, gamma 2, smooth muscle, enteric | 2.58 | A_11_P0000024853 | vav 3 guanine nucleotide exchange factor | -2.67 |
| A_11_P0000029347 | membrane-spanning 4-domains, subfamily A, member 1 | 2.58 | A_11_P000002462 |  | -2.67 |
| A_11_P0000021335 | glucokinase (hexokinase 4) regulator | 2.58 | A_11_P205843 | tetratricopeptide repeat, ankyrin repeat and coiled-coil containing 1 | -2.67 |
| A_11_P118456 |  | 2.58 | A_11_P083281 | ral guanine nucleotide dissociation stimulator | -2.66 |
| A_11_P0000024950 | neutrophil cytosolic factor 2 | 2.58 | A_11_P054086 | P450 (cytochrome) oxidoreductase | -2.66 |
| A_11_P0000022141 | leucine zipper transcription factor-like 1 | 2.57 | A_11_P160768 | secretory carrier membrane protein 5 | -2.66 |
| A_11_P155388 | kelch-like family member 5 | 2.57 | A_11_P0000022045 | SET domain containing 5 | -2.66 |
| A_11_P198378 |  | 2.57 | A_11_P0000019804 | ATP-binding cassette, sub-family C (CFTR/MRP), member 2 | -2.66 |
| A_11_P098761 | vasohibin 2 | 2.57 | A_11_P079801 |  | -2.66 |
| A_11_P065961 |  | 2.57 | A_11_P000002460 |  | -2.66 |
| A_11_P116616 | collagen, type V, alpha 2 | 2.57 | A_11_P0000012059 |  | -2.66 |
| A_11_P064456 | carbonic anhydrase II | 2.57 | A_11_P0000039710 |  | -2.66 |
| A_11_P181718 | moesin | 2.57 | A_11_P107926 | KIAA0513 ortholog | -2.66 |
| A_11_P143118 |  | 2.57 | A_11_P0000040283 |  | -2.65 |
| A_11_P0000013965 |  | 2.57 | A_11_P202528 |  | -2.65 |
| A_11_P0000033926 | frizzled family receptor 2 | 2.57 | A_11_P076126 | homer homolog 3 (Drosophila) | -2.65 |
| A_11_P155368 | GLI pathogenesis-related 1 | 2.56 | A_11_P0000033597 | twisted gastrulation homolog 1 (Drosophila) | -2.65 |
| A_11_P0000034763 | sushi-repeat containing protein, X-linked 2 | 2.56 | A_11_P0000040094 |  | -2.65 |
| A_11_P163913 |  | 2.56 | A_11_P0000027125 | Rho guanine nucleotide exchange factor (GEF) 5 | -2.65 |
| A_11_P059201 |  | 2.56 | A_11_P0000039739 |  | -2.65 |
| A_11_P164428 | protein kinase (cAMP-dependent, catalytic) inhibitor gamma | 2.56 | A_11_P0000024552 | agrin | -2.65 |
| A_11_P054726 |  | 2.56 | A_11_P054426 |  | -2.64 |
| A_11_P065126 | serine palmitoyltransferase, small subunit B | 2.56 | A_11_P174978 | 1-acylglycerol-3-phosphate O-acyltransferase 9 | -2.64 |
| A_11_P0000040519 | inter-alpha-trypsin inhibitor heavy chain 3 | 2.56 | A_11_P000007265 |  | -2.64 |
| A_11_P0000013747 | WWC family member 3 | 2.55 | A_11_P176203 |  | -2.64 |
| A_11_P152483 | fibrillin-1 | 2.55 | A_11_P201358 |  | -2.64 |
| A_11_P192263 | ceruloplasmin (ferroxidase) | 2.55 | A_11_P069711 | leucine zipper protein 2-like | -2.64 |
| A_11_P0000031048 | procollagen-lysine, 2-oxoglutarate 5-dioxygenase 1 | 2.55 | A_11_P0000029730 | syndecan 4 | -2.64 |
| A_11_P183763 |  | 2.55 | A_11_P053626 | sterol regulatory element binding transcription factor 1 | -2.64 |
| A_11_P0000023367 | eukaryotic translation initiation factor 3, subunit I | 2.55 | A_11_P000008882 |  | -2.64 |
| A_11_P101926 | GATA binding protein 3 | 2.55 | A_11_P050716 | 2'-5'-oligoadenylate synthetase 3, 100kDa | -2.64 |
| A_11_P176623 | protocadherin 18 | 2.55 | A_11_P184533 | MAX interactor 1, dimerization protein | -2.64 |
| A_11_P149893 | transforming growth factor, beta-induced, 68kDa | 2.55 | A_11_P0000023775 | ubiquitin specific peptidase 13 (isopeptidase T-3) | -2.64 |
| A_11_P000003753 |  | 2.55 | A_11_P0000027769 |  | -2.64 |
| A_11_P094171 |  | 2.55 | A_11_P000008360 |  | -2.63 |
| A_11_P056006 | CD1e molecule | 2.55 | A_11_P000004087 |  | -2.63 |
| A_11_P0000024868 | calponin 3, acidic | 2.55 | A_11_P056131 | PX domain containing 1 | -2.63 |
| A_11_P0000037213 |  | 2.55 | A_11_P000009027 |  | -2.63 |
| A_11_P0000031812 | calcitonin receptor-like | 2.54 | A_11_P000002437 |  | -2.63 |
| A_11_P154763 |  | 2.54 | A_11_P0000012133 | beta-defensin 140 | -2.63 |
| A_11_P207763 |  | 2.54 | A_11_P0000040245 |  | -2.63 |
| A_11_P151553 |  | 2.54 | A_11_P062746 | promyelocytic leukemia | -2.63 |
| A_11_P119706 | zinc finger protein 70 | 2.54 | A_11_P213423 | spermatogenesis associated, serine-rich 2-like | -2.63 |
| A_11_P00000250 | translocase of outer mitochondrial membrane 40 homolog (yeast) | 2.54 | A_11_P000007445 |  | -2.63 |
| A_11_P088376 |  | 2.54 | A_11_P150198 | selenoprotein P, plasma, 1 | -2.63 |
| A_11_P0000033531 | S100 calcium binding protein A12 | 2.54 | A_11_P112526 | carcinoembryonic antigen-related cell adhesion molecule 18-like | -2.63 |
| A_11_P098676 |  | 2.54 | A_11_P113386 | sterile alpha motif domain containing 4B | -2.62 |
| A_11_P079216 | ACN9 homolog (S. cerevisiae) | 2.54 | A_11_P207358 | serpin peptidase inhibitor, clade C (antithrombin), member 1 | -2.62 |
| A_11_P00000625 | protein kinase D1 | 2.54 | A_11_P0000017880 |  | -2.62 |
| A_11_P0000027898 | oxysterol binding protein-like 5 | 2.54 | A_11_P202158 |  | -2.62 |
| A_11_P195413 |  | 2.54 | A_11_P0000012062 |  | -2.62 |
| A_11_P052296 | beta-defensin 108B-like | 2.54 | A_11_P0000038318 |  | -2.62 |
| A_11_P0000014836 |  | 2.54 | A_11_P0000026270 | UDP-glucose ceramide glucosyltransferase | -2.62 |
| A_11_P0000025255 | AHA1, activator of heat shock 90kDa protein ATPase homolog 1 (yeast) | 2.54 | A_11_P175298 |  | -2.62 |
| A_11_P0000029610 | beaded filament structural protein 1, filensin | 2.54 | A_11_P060496 | phosphatidylserine decarboxylase | -2.62 |
| A_11_P0000041538 | chromosome 4 open reading frame, human C1orf198 | 2.54 | A_11_P0000015435 |  | -2.62 |
| A_11_P054766 | CD1b molecule | 2.53 | A_11_P225193 | Meis homeobox 1 | -2.62 |
| A_11_P000002691 |  | 2.53 | A_11_P0000013908 |  | -2.62 |
| A_11_P0000033430 | dermatopontin | 2.53 | A_11_P0000032220 | coagulation factor II (thrombin) receptor-like 1 | -2.62 |
| A_11_P119826 | abl-interactor 2 | 2.53 | A_11_P000001622 |  | -2.61 |
| A_11_P000003843 |  | 2.53 | A_11_P0000021248 | tankyrase, TRF1-interacting ankyrin-related ADP-ribose polymerase | -2.61 |
| A_11_P0000032082 | BCL2-related protein A1 | 2.53 | A_11_P218993 |  | -2.61 |
| A_11_P0000031821 | serum deprivation response | 2.53 | A_11_P121121 |  | -2.61 |
| A_11_P0000033920 | dephospho-CoA kinase domain containing | 2.53 | A_11_P00000828 |  | -2.61 |
| A_11_P170423 |  | 2.53 | A_11_P221783 | selenoprotein P, plasma, 1 | -2.61 |
| A_11_P133951 | four and a half LIM domains 1 | 2.53 | A_11_P181373 |  | -2.61 |
| A_11_P0000017797 |  | 2.53 | A_11_P0000039390 |  | -2.61 |
| A_11_P0000039357 |  | 2.52 | A_11_P103326 |  | -2.61 |
| A_11_P220428 | vascular endothelial growth factor B | 2.52 | A_11_P092476 | interleukin 25 | -2.61 |
| A_11_P110966 | dermatan sulfate epimerase | 2.52 | A_11_P0000041232 |  | -2.61 |
| A_11_P134161 | synaptopodin 2 | 2.52 | A_11_P173388 |  | -2.61 |
| A_11_P0000030398 | Rho GDP dissociation inhibitor (GDI) beta | 2.52 | A_11_P164233 |  | -2.61 |
| A_11_P000005324 |  | 2.52 | A_11_P0000021014 |  | -2.61 |
| A_11_P0000029513 | leucine rich repeat containing 3B | 2.52 | A_11_P128496 |  | -2.60 |
| A_11_P126991 | cellular repressor of E1A-stimulated genes 1 | 2.52 | A_11_P061409 | carbonyl reductase [NADPH] 1-like | -2.60 |
| A_11_P0000025512 | argininosuccinate synthase 1 | 2.52 | A_11_P0000025584 | carbonyl reductase [NADPH] 1-like | -2.60 |
| A_11_P162053 |  | 2.52 | A_11_P197773 | attractin | -2.60 |
| A_11_P0000041473 | calcium/calmodulin-dependent protein kinase ID | 2.51 | A_11_P00000849 | O-linked N-acetylglucosamine (GlcNAc) transferase | -2.60 |
| A_11_P075131 | calcium/calmodulin-dependent protein kinase I | 2.51 | A_11_P0000039701 |  | -2.60 |
| A_11_P182203 | FXYD domain containing ion transport regulator 6 | 2.51 | A_11_P0000033796 | apoptosis resistant E3 ubiquitin protein ligase 1 | -2.60 |
| A_11_P196123 | regulatory associated protein of MTOR, complex 1 | 2.51 | A_11_P0000033818 |  | -2.60 |
| A_11_P0000028454 | C-type lectin domain family 11, member A | 2.51 | A_11_P161193 |  | -2.60 |
| A_11_P0000041971 | cell death-inducing DFFA-like effector a | 2.51 | A_11_P059241 | HECT and RLD domain containing E3 ubiquitin protein ligase 3 | -2.60 |
| A_11_P0000020898 | immunoglobulin J polypeptide, linker protein for immunoglobulin alpha and mu polypeptides | 2.51 | A_11_P199088 |  | -2.60 |
| A_11_P0000022391 |  | 2.51 | A_11_P157328 |  | -2.60 |
| A_11_P114646 | FtsJ RNA methyltransferase homolog 1 (E. coli) | 2.51 | A_11_P057901 |  | -2.60 |
| A_11_P105971 | adhesion molecule, interacts with CXADR antigen 1 | 2.51 | A_11_P0000014114 |  | -2.59 |
| A_11_P210923 | crystallin, alpha B | 2.51 | A_11_P0000017415 |  | -2.59 |
| A_11_P0000040788 | guanylate cyclase 1, soluble, beta 3 | 2.51 | A_11_P184328 |  | -2.59 |
| A_11_P054741 |  | 2.51 | A_11_P133716 | amphiphysin | -2.59 |
| A_11_P0000029365 | KIAA0226-like ortholog | 2.51 | A_11_P068331 | pannexin 1 | -2.59 |
| A_11_P174948 | family with sequence similarity 129, member A | 2.50 | A_11_P0000017789 |  | -2.59 |
| A_11_P090271 | transmembrane protein 17 | 2.50 | A_11_P147963 |  | -2.59 |
| A_11_P153633 | glutathione peroxidase 1 | 2.50 | A_11_P154873 |  | -2.59 |
| A_11_P098731 | transmembrane protein 206 | 2.50 | A_11_P0000033666 |  | -2.58 |
| A_11_P0000017078 | LFNG O-fucosylpeptide 3-beta-N-acetylglucosaminyltransferase | 2.50 | A_11_P0000040272 |  | -2.58 |
| A_11_P050096 | transmembrane protein 47 | 2.50 | A_11_P190308 |  | -2.58 |
| A_11_P201873 | platelet/endothelial cell adhesion molecule 1 | 2.50 | A_11_P0000012015 |  | -2.58 |
| A_11_P092961 |  | 2.50 | A_11_P152423 |  | -2.58 |
| A_11_P214308 | protein disulfide isomerase family A, member 6 | 2.50 | A_11_P210533 |  | -2.58 |
| A_11_P0000020011 | potassium large conductance calcium-activated channel, subfamily M, beta member 1 | 2.50 | A_11_P165843 | transmembrane protein 38B | -2.58 |
| A_11_P221843 | nuclear factor I/B | 2.50 | A_11_P0000036272 |  | -2.58 |
| A_11_P000006067 |  | 2.50 | A_11_P0000024271 | annexin A7 | -2.58 |
| A_11_P081986 | prohibitin | 2.50 | A_11_P109446 | KIAA0232 ortholog | -2.58 |
| A_11_P058941 |  | 2.49 | A_11_P0000012418 |  | -2.58 |
| A_11_P0000014457 |  | 2.49 | A_11_P053551 | interleukin 18 (interferon-gamma-inducing factor) | -2.58 |
| A_11_P136766 | protein disulfide isomerase family A, member 6 | 2.49 | A_11_P160033 |  | -2.58 |
| A_11_P111081 | sphingomyelin phosphodiesterase, acid-like 3A | 2.49 | A_11_P0000027498 | OTU domain containing 7B | -2.57 |
| A_11_P0000020115 | toll-like receptor 2 | 2.49 | A_11_P000004075 |  | -2.57 |
| A_11_P000003451 |  | 2.49 | A_11_P152398 | O-linked N-acetylglucosamine (GlcNAc) transferase | -2.57 |
| A_11_P00000810 | pre-mRNA processing factor 19 | 2.49 | A_11_P160953 |  | -2.57 |
| A_11_P0000023089 | ADP-ribosylation factor-like 3 | 2.49 | A_11_P00000997 | chymotrypsin-like | -2.57 |
| A_11_P0000020958 | tachykinin, precursor 1 | 2.49 | A_11_P123391 |  | -2.57 |
| A_11_P000005799 | thioesterase superfamily member 4 | 2.49 | A_11_P000004213 |  | -2.57 |
| A_11_P050361 | lymphotoxin beta (TNF superfamily, member 3) | 2.49 | A_11_P171673 | ring finger protein 19B | -2.57 |
| A_11_P0000018880 | hairy/enhancer-of-split related with YRPW motif 1 | 2.49 | A_11_P108351 | chymotrypsin-like | -2.57 |
| A_11_P0000039580 |  | 2.49 | A_11_P0000035070 |  | -2.57 |
| A_11_P0000033526 | S100 calcium binding protein A13 | 2.49 | A_11_P0000025240 | ectonucleoside triphosphate diphosphohydrolase 5 | -2.57 |
| A_11_P0000019727 | dynein, light chain, Tctex-type 3 | 2.49 | A_11_P164078 |  | -2.57 |
| A_11_P0000039303 |  | 2.49 | A_11_P0000018035 |  | -2.56 |
| A_11_P0000029671 | hemopoietic cell kinase | 2.48 | A_11_P151988 | interferon-induced protein 44-like | -2.56 |
| A_11_P161948 |  | 2.48 | A_11_P0000031995 | SH2 domain containing 1B | -2.56 |
| A_11_P117006 | destrin (actin depolymerizing factor) | 2.48 | A_11_P0000023078 | dynamin binding protein | -2.56 |
| A_11_P0000088 | nuclear factor I/B | 2.48 | A_11_P068021 |  | -2.56 |
| A_11_P0000022116 | glutathione peroxidase 1 | 2.48 | A_11_P111216 | cell division cycle 14B | -2.56 |
| A_11_P109131 | family with sequence similarity 174, member B | 2.48 | A_11_P181783 | flightless I homolog (Drosophila) | -2.56 |
| A_11_P203023 | olfactomedin 1 | 2.48 | A_11_P145888 |  | -2.56 |
| A_11_P094786 | transforming growth factor, beta-induced, 68kDa | 2.48 | A_11_P175283 | enoyl CoA hydratase domain containing 2 | -2.56 |
| A_11_P0000015491 |  | 2.48 | A_11_P091686 | small ArfGAP 1 | -2.56 |
| A_11_P058426 | pleckstrin homology-like domain, family B, member 2 | 2.48 | A_11_P106516 | transmembrane 4 L six family member 5 | -2.56 |
| A_11_P00000436 | erythrocyte membrane protein band 4.1-like 1 | 2.48 | A_11_P0000034650 | synapsin I | -2.55 |
| A_11_P0000020174 | caveolin 2 | 2.47 | A_11_P0000021920 | electron-transfer-flavoprotein, beta polypeptide | -2.55 |
| A_11_P115371 | transcription elongation factor A (SII)-like 1 | 2.47 | A_11_P0000034568 | glycine receptor, alpha 2 | -2.55 |
| A_11_P052931 | thymidylate synthetase | 2.47 | A_11_P00000504 | tetratricopeptide repeat domain 3 | -2.55 |
| A_11_P00000690 | serglycin | 2.47 | A_11_P0000016608 |  | -2.55 |
| A_11_P108451 | Ras-related associated with diabetes | 2.47 | A_11_P056911 | tetratricopeptide repeat, ankyrin repeat and coiled-coil containing 1 | -2.55 |
| A_11_P060151 | dynein, light chain, LC8-type 1 | 2.47 | A_11_P0000021327 | abhydrolase domain containing 1 | -2.55 |
| A_11_P0000039218 |  | 2.47 | A_11_P082986 | ATPase, Ca++ transporting, ubiquitous | -2.55 |
| A_11_P138336 | microfibrillar-associated protein 4 | 2.47 | A_11_P000008378 |  | -2.55 |
| A_11_P061036 | protein kinase, cGMP-dependent, type I | 2.47 | A_11_P077456 | mucosal vascular addressin cell adhesion molecule 1 | -2.55 |
| A_11_P060996 | stromal cell-derived factor 2-like 1 | 2.47 | A_11_P0000017663 |  | -2.55 |
| A_11_P106056 | cell adhesion molecule 1 | 2.46 | A_11_P0000020130 | amyloid beta (A4) precursor protein | -2.55 |
| A_11_P162163 | ribosomal protein S6 kinase, 90kDa, polypeptide 2 | 2.46 | A_11_P106026 |  | -2.54 |
| A_11_P0000023402 | complement component 1, q subcomponent, A chain | 2.46 | A_11_P195978 |  | -2.54 |
| A_11_P0000038820 |  | 2.46 | A_11_P0000035538 |  | -2.54 |
| A_11_P132936 | FK506 binding protein 10, 65 kDa | 2.46 | A_11_P000003243 |  | -2.54 |
| A_11_P081161 | CD79b molecule, immunoglobulin-associated beta | 2.46 | A_11_P0000025248 | tubulin tyrosine ligase-like family, member 5 | -2.54 |
| A_11_P0000020349 | solute carrier family 25 (mitochondrial carrier; peroxisomal membrane protein, 34kDa), member 17 | 2.46 | A_11_P120231 | olfactomedin-like 2A | -2.54 |
| A_11_P0000016821 |  | 2.46 | A_11_P000007045 |  | -2.54 |
| A_11_P0000025544 | heat shock 70kDa protein 5 (glucose-regulated protein, 78kDa) | 2.46 | A_11_P068081 | synaptonemal complex protein 2 | -2.54 |
| A_11_P165768 | ets homologous factor | 2.46 | A_11_P060426 | galactose-3-O-sulfotransferase 1 | -2.54 |
| A_11_P086886 | transmembrane protein 214 | 2.46 | A_11_P0000021623 | EH-domain containing 1 | -2.54 |
| A_11_P0000023599 | beta-site APP-cleaving enzyme 2 | 2.46 | A_11_P0000011605 |  | -2.54 |
| A_11_P0000014556 | lysosomal protein transmembrane 5 | 2.45 | A_11_P0000028539 | excision repair cross-complementing rodent repair deficiency, complementation group 2 | -2.54 |
| A_11_P159353 | oligonucleotide/oligosaccharide-binding fold containing 1 | 2.45 | A_11_P0000014213 | 1-acylglycerol-3-phosphate O-acyltransferase 9 | -2.54 |
| A_11_P0000034272 | NIMA-related kinase 6 | 2.45 | A_11_P0000018399 |  | -2.54 |
| A_11_P0000034651 | complement factor properdin | 2.45 | A_11_P066886 | glycerophosphocholine phosphodiesterase GDE1 homolog (S. cerevisiae) | -2.53 |
| A_11_P174703 |  | 2.45 | A_11_P200268 |  | -2.53 |
| A_11_P145753 |  | 2.45 | A_11_P0000040043 |  | -2.53 |
| A_11_P157603 | tumor suppressor candidate 3 | 2.45 | A_11_P214488 |  | -2.53 |
| A_11_P0000027576 |  | 2.45 | A_11_P066961 | attractin | -2.53 |
| A_11_P0000026311 |  | 2.45 | A_11_P146963 |  | -2.53 |
| A_11_P0000040918 |  | 2.45 | A_11_P000003373 |  | -2.53 |
| A_11_P076936 | complement component 3 | 2.45 | A_11_P0000024745 | dynactin 5 (p25) | -2.53 |
| A_11_P188973 | ribosomal protein S5 | 2.45 | A_11_P000003061 |  | -2.53 |
| A_11_P172673 |  | 2.45 | A_11_P0000041936 |  | -2.53 |
| A_11_P000002593 | integrin, alpha 5 (fibronectin receptor, alpha polypeptide) | 2.45 | A_11_P0000017177 |  | -2.53 |
| A_11_P192393 |  | 2.45 | A_11_P0000018787 |  | -2.53 |
| A_11_P179043 |  | 2.44 | A_11_P179548 |  | -2.53 |
| A_11_P095041 | protein tyrosine phosphatase-like A domain containing 2 | 2.44 | A_11_P0000039189 | perilipin 2 | -2.52 |
| A_11_P073206 | glutamine and serine rich 1 | 2.44 | A_11_P0000032121 | UV-stimulated scaffold protein A | -2.52 |
| A_11_P0000017314 | colony stimulating factor 1 (macrophage) | 2.44 | A_11_P170403 | cytochrome P450 2C21 | -2.52 |
| A_11_P0000014530 |  | 2.44 | A_11_P0000028910 | olfactory receptor 7C1-like | -2.52 |
| A_11_P0000024889 | LIM domain only 4 | 2.44 | A_11_P0000016141 |  | -2.52 |
| A_11_P0000021493 | laminin, beta 1 | 2.44 | A_11_P178268 | activating signal cointegrator 1 complex subunit 2 | -2.52 |
| A_11_P0000025028 | chaperonin containing TCP1, subunit 3 (gamma) | 2.44 | A_11_P199868 |  | -2.52 |
| A_11_P0000026024 |  | 2.44 | A_11_P000009036 |  | -2.52 |
| A_11_P0000032647 | complement component 1, q subcomponent binding protein | 2.44 | A_11_P093796 | calmin (calponin-like, transmembrane) | -2.52 |
| A_11_P205273 | transmembrane protein 109 | 2.44 | A_11_P087826 | synaptonemal complex protein 1 | -2.52 |
| A_11_P211568 |  | 2.44 | A_11_P067651 | TOX high mobility group box family member 2 | -2.52 |
| A_11_P092151 |  | 2.44 | A_11_P145583 |  | -2.52 |
| A_11_P125136 | praja ring finger 1, E3 ubiquitin protein ligase | 2.43 | A_11_P000003295 |  | -2.52 |
| A_11_P105606 | checkpoint kinase 1 | 2.43 | A_11_P084356 | plectin | -2.52 |
| A_11_P070313 | PIN2/TERF1 interacting, telomerase inhibitor 1 | 2.43 | A_11_P0000012957 |  | -2.52 |
| A_11_P158073 | ectonucleotide pyrophosphatase/phosphodiesterase 6 | 2.43 | A_11_P000009294 |  | -2.52 |
| A_11_P155363 | WD repeat domain, phosphoinositide interacting 1 | 2.43 | A_11_P171163 |  | -2.52 |
| A_11_P050046 |  | 2.43 | A_11_P0000030849 | mesoderm induction early response 1, family member 3 | -2.52 |
| A_11_P194518 |  | 2.43 | A_11_P0000030806 | neuregulin 2 | -2.51 |
| A_11_P0000025953 | parvalbumin | 2.43 | A_11_P112481 | sialic acid binding Ig-like lectin 10 | -2.51 |
| A_11_P099261 |  | 2.43 | A_11_P0000014509 |  | -2.51 |
| A_11_P073931 | insulin-like growth factor 2 (somatomedin A) | 2.43 | A_11_P0000013536 |  | -2.51 |
| A_11_P196303 |  | 2.43 | A_11_P150733 |  | -2.51 |
| A_11_P210408 |  | 2.43 | A_11_P069354 | cOR10AB2 olfactory receptor family 10 subfamily AB-like | -2.51 |
| A_11_P067926 |  | 2.43 | A_11_P000001292 | carnitine palmitoyltransferase 1A (liver) | -2.51 |
| A_11_P157223 | protein LDOC1-like | 2.43 | A_11_P099081 | SUN domain containing ossification factor | -2.51 |
| A_11_P169978 |  | 2.43 | A_11_P0000017540 |  | -2.51 |
| A_11_P0000032163 |  | 2.42 | A_11_P0000030281 |  | -2.51 |
| A_11_P064831 |  | 2.42 | A_11_P0000030437 | calsyntenin 3 | -2.51 |
| A_11_P0000026054 |  | 2.42 | A_11_P176568 |  | -2.51 |
| A_11_P0000018028 |  | 2.42 | A_11_P106961 | sperm antigen with calponin homology and coiled-coil domains 1 | -2.51 |
| A_11_P056966 | fibroblast activation protein, alpha | 2.42 | A_11_P210778 |  | -2.51 |
| A_11_P0000016656 | FXYD domain containing ion transport regulator 6 | 2.42 | A_11_P208958 | chymotrypsin-like | -2.51 |
| A_11_P0000014916 | neuron specific gene family member 1 | 2.42 | A_11_P100913 | signal-regulatory protein beta-1 isoform 3-like | -2.51 |
| A_11_P100111 | zinc finger protein 521 | 2.42 | A_11_P098436 |  | -2.51 |
| A_11_P0000014954 |  | 2.42 | A_11_P0000041789 |  | -2.51 |
| A_11_P0000017687 |  | 2.42 | A_11_P176773 |  | -2.51 |
| A_11_P061176 | protein BTG3-like | 2.41 | A_11_P198518 |  | -2.51 |
| A_11_P0000030351 | solute carrier family 2 (facilitated glucose transporter), member 13 | 2.41 | A_11_P0000021635 | phospholipase A2, group XVI-like | -2.51 |
| A_11_P172353 |  | 2.41 | A_11_P000006861 |  | -2.50 |
| A_11_P061526 | beta-site APP-cleaving enzyme 2 | 2.41 | A_11_P0000025594 |  | -2.50 |
| A_11_P171293 | TYRO3 protein tyrosine kinase | 2.41 | A_11_P0000019887 | interleukin 18 (interferon-gamma-inducing factor) | -2.50 |
| A_11_P102856 | iroquois homeobox 5 | 2.41 | A_11_P0000018741 |  | -2.50 |
| A_11_P096911 | immunoglobulin superfamily, member 6 | 2.41 | A_11_P059991 | TRAF-type zinc finger domain containing 1 | -2.50 |
| A_11_P079111 | cyclin-dependent kinase 14 | 2.41 | A_11_P062021 | phospholipase A2, group IVF | -2.50 |
| A_11_P051231 | sulfatase 2 | 2.41 | A_11_P201533 |  | -2.50 |
| A_11_P0000021278 | ectonucleotide pyrophosphatase/phosphodiesterase 6 | 2.41 | A_11_P0000041715 |  | -2.50 |
| A_11_P0000040835 | homeobox B7 | 2.41 | A_11_P0000028732 | RNA pseudouridylate synthase domain containing 3 | -2.50 |
| A_11_P0000028332 | ninjurin 1 | 2.40 | A_11_P0000019880 | integrin, beta 3 (platelet glycoprotein IIIa, antigen CD61) | -2.50 |
| A_11_P073018 |  | 2.40 | A_11_P0000020996 |  | -2.50 |
| A_11_P117496 | GM2 ganglioside activator | 2.40 | A_11_P0000011677 |  | -2.49 |
| A_11_P0000041965 | chibby homolog 1 (Drosophila) | 2.40 | A_11_P077836 | transmembrane protein 140 | -2.49 |
| A_11_P186303 | heat shock protein 90kDa beta (Grp94), member 1 | 2.40 | A_11_P0000019480 |  | -2.49 |
| A_11_P082331 | T-box 2 | 2.40 | A_11_P104331 |  | -2.49 |
| A_11_P191688 | interferon-related developmental regulator 2 | 2.40 | A_11_P0000027798 | olfactory receptor 1052-like | -2.49 |
| A_11_P050436 | RAB27A, member RAS oncogene family | 2.40 | A_11_P110791 |  | -2.49 |
| A_11_P0000031945 | T-cell surface glycoprotein CD1a-like | 2.40 | A_11_P153053 |  | -2.49 |
| A_11_P000003656 |  | 2.40 | A_11_P190503 |  | -2.49 |
| A_11_P0000021123 | nucleoporin 37kDa | 2.40 | A_11_P071396 | solute carrier family 4, sodium bicarbonate cotransporter, member 7 | -2.49 |
| A_11_P0000030987 | UDP-galactose-4-epimerase | 2.40 | A_11_P0000029628 | glycerophosphocholine phosphodiesterase GDE1 homolog (S. cerevisiae) | -2.49 |
| A_11_P0000025167 | mirror-image polydactyly 1 | 2.40 | A_11_P141105 |  | -2.49 |
| A_11_P000003377 |  | 2.40 | A_11_P0000040335 |  | -2.49 |
| A_11_P113229 |  | 2.40 | A_11_P0000015699 |  | -2.48 |
| A_11_P0000028158 | transcription factor 21 | 2.40 | A_11_P0000031030 | EPH receptor A2 | -2.48 |
| A_11_P103841 |  | 2.39 | A_11_P063311 | ectonucleoside triphosphate diphosphohydrolase 7 | -2.48 |
| A_11_P180758 | cytochrome P450, family 1, subfamily B, polypeptide 1 | 2.39 | A_11_P0000018936 |  | -2.48 |
| A_11_P187623 | ribosomal protein L35 | 2.39 | A_11_P094761 | paired-like homeodomain 1 | -2.48 |
| A_11_P139706 | endothelin receptor type B | 2.39 | A_11_P205093 | protein inhibitor of activated STAT, 1 | -2.48 |
| A_11_P075356 | protein tyrosine phosphatase, receptor type, G | 2.39 | A_11_P000008703 |  | -2.48 |
| A_11_P052371 | collagen, type IV, alpha 2 | 2.39 | A_11_P0000010718 |  | -2.48 |
| A_11_P0000032559 | CXADR-like membrane protein | 2.39 | A_11_P0000019276 |  | -2.48 |
| A_11_P148593 | deoxyribonuclease II, lysosomal | 2.39 | A_11_P196558 | transmembrane protein 2 | -2.48 |
| A_11_P074506 | vascular endothelial growth factor B | 2.39 | A_11_P0000010194 |  | -2.48 |
| A_11_P121456 | NDRG family member 3 | 2.39 | A_11_P057501 | serine/threonine kinase 17b | -2.48 |
| A_11_P0000031476 | follistatin-like 1 | 2.39 | A_11_P208793 |  | -2.48 |
| A_11_P191833 |  | 2.39 | A_11_P088726 |  | -2.48 |
| A_11_P091851 | chromosome 12 open reading frame, human C6orf165 | 2.39 | A_11_P087201 | mitogen-activated protein kinase kinase kinase kinase 3 | -2.48 |
| A_11_P083831 |  | 2.39 | A_11_P112931 |  | -2.48 |
| A_11_P0000016840 | G protein-coupled receptor associated sorting protein 2 | 2.39 | A_11_P068766 |  | -2.48 |
| A_11_P071261 | ectonucleoside triphosphate diphosphohydrolase 3 | 2.39 | A_11_P0000040689 | G protein-coupled receptor 133 | -2.47 |
| A_11_P163823 |  | 2.38 | A_11_P0000032150 | sel-1 suppressor of lin-12-like 3 (C. elegans) | -2.47 |
| A_11_P0000031154 | testis expressed 9 | 2.38 | A_11_P0000021034 |  | -2.47 |
| A_11_P0000041352 |  | 2.38 | A_11_P0000039696 |  | -2.47 |
| A_11_P0000028469 | prostate tumor overexpressed 1 | 2.38 | A_11_P200603 |  | -2.47 |
| A_11_P168478 |  | 2.38 | A_11_P092411 | solute carrier family 7 (amino acid transporter light chain, y+L system), member 7 | -2.47 |
| A_11_P076736 | mitochondrial ribosomal protein L4 | 2.38 | A_11_P050146 | glucose-6-phosphatase, catalytic subunit | -2.47 |
| A_11_P0000030614 | BCL2-associated athanogene 3 | 2.38 | A_11_P0000028733 |  | -2.47 |
| A_11_P155573 | sorting nexin 10 | 2.38 | A_11_P0000034723 |  | -2.47 |
| A_11_P0000020166 | chemokine (C-X-C motif) ligand 10 | 2.38 | A_11_P000001054 |  | -2.47 |
| A_11_P0000013960 |  | 2.38 | A_11_P215928 |  | -2.47 |
| A_11_P171903 | KIT ligand | 2.38 | A_11_P202163 |  | -2.47 |
| A_11_P157868 |  | 2.38 | A_11_P0000034922 | dual specificity phosphatase 9 | -2.47 |
| A_11_P0000025316 |  | 2.38 | A_11_P111321 | solute carrier family 28 (concentrative nucleoside transporter), member 3 | -2.46 |
| A_11_P0000031748 | UDP-N-acetyl-alpha-D-galactosamine:polypeptide N-acetylgalactosaminyltransferase 5 (GalNAc-T5) | 2.38 | A_11_P0000022939 | Rho family GTPase 1 | -2.46 |
| A_11_P114326 | chromosome X open reading frame, human CXorf21 | 2.38 | A_11_P187283 | MHC class I DLA-12 | -2.46 |
| A_11_P0000023740 | uridine monophosphate synthetase | 2.38 | A_11_P0000014097 |  | -2.46 |
| A_11_P0000037144 | RASD family, member 2 | 2.38 | A_11_P174043 | troponin T type 2 (cardiac) | -2.46 |
| A_11_P063271 | cartilage acidic protein 1 | 2.37 | A_11_P0000033509 | pygopus homolog 2 (Drosophila) | -2.46 |
| A_11_P179868 |  | 2.37 | A_11_P0000024359 | RPTOR independent companion of MTOR, complex 2 | -2.46 |
| A_11_P064671 | chaperonin containing TCP1, subunit 5 (epsilon) | 2.37 | A_11_P105846 | ubiquitin specific peptidase 2 | -2.46 |
| A_11_P086601 | membrane bound O-acyltransferase domain containing 2 | 2.37 | A_11_P105191 | coiled-coil domain containing 152 | -2.46 |
| A_11_P0000034697 | moesin | 2.37 | A_11_P083496 | family with sequence similarity 102, member A | -2.46 |
| A_11_P0000028452 | uncharacterized LOC484356 | 2.37 | A_11_P0000022925 | solute carrier family 4, sodium bicarbonate cotransporter, member 8 | -2.46 |
| A_11_P093721 | ankyrin repeat and SOCS box containing 2 | 2.37 | A_11_P053631 | myosin, heavy chain 8, skeletal muscle, perinatal | -2.46 |
| A_11_P080131 | progesterone immunomodulatory binding factor 1 | 2.37 | A_11_P0000028612 | actinin, alpha 4 | -2.46 |
| A_11_P166458 | RNA-binding protein Nova-1-like | 2.37 | A_11_P128926 |  | -2.46 |
| A_11_P105931 | pleckstrin homology-like domain, family B, member 1 | 2.37 | A_11_P0000010022 |  | -2.46 |
| A_11_P0000017101 | hepatocyte nuclear factor 1-beta-like | 2.37 | A_11_P176593 | ankyrin 3, node of Ranvier (ankyrin G) | -2.46 |
| A_11_P093541 | AHA1, activator of heat shock 90kDa protein ATPase homolog 1 (yeast) | 2.37 | A_11_P167278 |  | -2.46 |
| A_11_P0000021614 | EGF containing fibulin-like extracellular matrix protein 2 | 2.37 | A_11_P183253 |  | -2.46 |
| A_11_P0000023662 | H2A histone family, member Z | 2.37 | A_11_P0000010692 |  | -2.45 |
| A_11_P0000015821 |  | 2.37 | A_11_P0000011515 |  | -2.45 |
| A_11_P052526 | glutaminyl-peptide cyclotransferase | 2.37 | A_11_P0000032426 | N-deacetylase/N-sulfotransferase (heparan glucosaminyl) 1 | -2.45 |
| A_11_P123086 | leukocyte-associated immunoglobulin-like receptor 1 | 2.36 | A_11_P0000017971 |  | -2.45 |
| A_11_P142253 | zinc finger protein 271-like | 2.36 | A_11_P0000014463 |  | -2.45 |
| A_11_P0000017020 |  | 2.36 | A_11_P0000016899 |  | -2.45 |
| A_11_P0000031672 | guanosine monophosphate reductase | 2.36 | A_11_P096546 | alpha hemoglobin stabilizing protein | -2.45 |
| A_11_P080546 |  | 2.36 | A_11_P056341 | jumonji, AT rich interactive domain 2 | -2.45 |
| A_11_P136776 | eukaryotic translation elongation factor 2 | 2.36 | A_11_P0000010833 |  | -2.45 |
| A_11_P0000026200 | sigma non-opioid intracellular receptor 1 | 2.36 | A_11_P221568 | transient receptor potential cation channel, subfamily V, member 6 | -2.45 |
| A_11_P201178 |  | 2.36 | A_11_P0000039968 |  | -2.45 |
| A_11_P0000015652 | MpV17 mitochondrial inner membrane protein | 2.35 | A_11_P00000781 | junction plakoglobin | -2.45 |
| A_11_P117706 | fibroblast growth factor 13-like | 2.35 | A_11_P202553 |  | -2.45 |
| A_11_P0000024972 | calcyclin binding protein | 2.35 | A_11_P000005446 |  | -2.45 |
| A_11_P131926 |  | 2.35 | A_11_P0000019706 | collagen, type VII, alpha 1 | -2.45 |
| A_11_P055571 | spermatogenesis associated 17 | 2.35 | A_11_P199273 | anti-Mullerian hormone receptor, type II | -2.45 |
| A_11_P0000012546 |  | 2.35 | A_11_P0000020535 | perilipin 2 | -2.44 |
| A_11_P156993 | FXYD domain containing ion transport regulator 6 | 2.35 | A_11_P155148 |  | -2.44 |
| A_11_P110701 | regulator of G-protein signaling 17 | 2.35 | A_11_P100936 | signal-regulatory protein beta-1 isoform 3-like | -2.44 |
| A_11_P056351 | CAP, adenylate cyclase-associated protein, 2 (yeast) | 2.35 | A_11_P171278 |  | -2.44 |
| A_11_P0000016364 |  | 2.35 | A_11_P164308 | membrane protein, palmitoylated 6 (MAGUK p55 subfamily member 6) | -2.44 |
| A_11_P0000020 | methionine sulfoxide reductase B3 | 2.35 | A_11_P0000029479 | cysteine-serine-rich nuclear protein 1 | -2.44 |
| A_11_P0000015471 |  | 2.35 | A_11_P0000010138 |  | -2.44 |
| A_11_P0000023909 | chimerin 1 | 2.35 | A_11_P0000014507 |  | -2.44 |
| A_11_P057196 | chimerin 1 | 2.35 | A_11_P070661 |  | -2.44 |
| A_11_P0000039664 |  | 2.35 | A_11_P216373 |  | -2.44 |
| A_11_P0000028099 | glycosyltransferase-like domain containing 1 | 2.35 | A_11_P074686 |  | -2.44 |
| A_11_P173913 | chaperonin containing TCP1, subunit 6A (zeta 1) | 2.35 | A_11_P169213 | WD repeat domain 60 | -2.44 |
| A_11_P0000021816 | dermatan sulfate epimerase | 2.34 | A_11_P066406 | G protein-coupled receptor 162 | -2.44 |
| A_11_P0000027980 | multiple endocrine neoplasia I | 2.34 | A_11_P0000017633 |  | -2.44 |
| A_11_P0000040564 |  | 2.34 | A_11_P0000029753 | UDP-Gal:betaGlcNAc beta 1,4- galactosyltransferase, polypeptide 5 | -2.44 |
| A_11_P194988 | eukaryotic translation initiation factor 3, subunit H | 2.34 | A_11_P066316 |  | -2.43 |
| A_11_P0000040367 |  | 2.34 | A_11_P0000020149 | solute carrier family 5 (sodium/glucose cotransporter), member 1 | -2.43 |
| A_11_P0000040925 |  | 2.34 | A_11_P000003454 |  | -2.43 |
| A_11_P000001229 |  | 2.34 | A_11_P000004635 |  | -2.43 |
| A_11_P0000015024 |  | 2.34 | A_11_P083676 | DENN/MADD domain containing 1A | -2.43 |
| A_11_P074801 | solute carrier family 41, member 3 | 2.34 | A_11_P0000040319 |  | -2.43 |
| A_11_P202988 | NIMA-related kinase 6 | 2.34 | A_11_P083551 | family with sequence similarity 129, member B | -2.43 |
| A_11_P219843 | phosphoglucomutase 1 | 2.34 | A_11_P073866 | mucin-6-like | -2.43 |
| A_11_P0000028289 | glucosaminyl (N-acetyl) transferase 1, core 2 | 2.34 | A_11_P0000041867 |  | -2.43 |
| A_11_P079231 | tachykinin, precursor 1 | 2.34 | A_11_P115991 |  | -2.43 |
| A_11_P0000039386 | ankyrin repeat domain 29 | 2.34 | A_11_P0000040232 |  | -2.43 |
| A_11_P0000010694 |  | 2.34 | A_11_P000008155 |  | -2.43 |
| A_11_P0000027265 | macrophage scavenger receptor 1 | 2.34 | A_11_P100161 | Niemann-Pick disease, type C1 | -2.43 |
| A_11_P0000020246 | 5-hydroxytryptamine (serotonin) receptor 2B, G protein-coupled | 2.34 | A_11_P0000035594 | junction plakoglobin | -2.43 |
| A_11_P088186 |  | 2.34 | A_11_P182943 | lysine (K)-specific demethylase 5B | -2.43 |
| A_11_P0000022785 |  | 2.33 | A_11_P135131 |  | -2.43 |
| A_11_P169463 | DEAD (Asp-Glu-Ala-Asp) box polypeptide 54 | 2.33 | A_11_P081541 | DEXH (Asp-Glu-X-His) box polypeptide 58 | -2.43 |
| A_11_P000003240 |  | 2.33 | A_11_P000008612 |  | -2.43 |
| A_11_P079226 | tachykinin, precursor 1 | 2.33 | A_11_P099381 | consortin, connexin sorting protein | -2.43 |
| A_11_P054262 |  | 2.33 | A_11_P193163 |  | -2.43 |
| A_11_P0000024154 | ribosomal protein L9 | 2.33 | A_11_P198603 | peptidyl arginine deiminase, type II | -2.42 |
| A_11_P000008547 |  | 2.33 | A_11_P185948 |  | -2.42 |
| A_11_P166738 | sphingomyelin phosphodiesterase, acid-like 3A | 2.33 | A_11_P0000016429 | cAMP responsive element binding protein 3-like 3 | -2.42 |
| A_11_P109356 | SH3-domain GRB2-like 3 | 2.33 | A_11_P078231 | tumor necrosis factor receptor superfamily, member 21 | -2.42 |
| A_11_P081461 |  | 2.33 | A_11_P102001 | Kruppel-like factor 6 | -2.42 |
| A_11_P0000025830 | cyclin-dependent kinase 4 | 2.32 | A_11_P075871 | collagen, type VII, alpha 1 | -2.42 |
| A_11_P0000020104 | chemokine (C-C motif) ligand 26 | 2.32 | A_11_P0000011100 | hepcidin antimicrobial peptide | -2.42 |
| A_11_P172258 |  | 2.32 | A_11_P0000032151 | solute carrier family 34 (type II sodium/phosphate contransporter), member 2 | -2.42 |
| A_11_P130911 |  | 2.32 | A_11_P198983 |  | -2.42 |
| A_11_P0000039052 |  | 2.32 | A_11_P0000029435 | ligase IV, DNA, ATP-dependent | -2.42 |
| A_11_P0000052 | death-domain associated protein | 2.32 | A_11_P000002335 |  | -2.42 |
| A_11_P066136 | matrix Gla protein | 2.32 | A_11_P186438 |  | -2.42 |
| A_11_P137861 | roundabout, axon guidance receptor, homolog 1 (Drosophila) | 2.32 | A_11_P0000020589 |  | -2.42 |
| A_11_P097921 | KIAA1324 ortholog | 2.32 | A_11_P000008094 |  | -2.42 |
| A_11_P0000039130 |  | 2.32 | A_11_P0000034821 |  | -2.42 |
| A_11_P195633 | phospholipase A2, group IB (pancreas) | 2.32 | A_11_P177098 |  | -2.42 |
| A_11_P0000041924 | X-prolyl aminopeptidase (aminopeptidase P) 3, putative | 2.32 | A_11_P058751 |  | -2.42 |
| A_11_P170463 | lysophosphatidylcholine acyltransferase 1 | 2.32 | A_11_P0000016329 |  | -2.41 |
| A_11_P113801 | programmed cell death 2-like | 2.32 | A_11_P0000015986 | aldolase B, fructose-bisphosphate | -2.41 |
| A_11_P0000020306 | potassium large conductance calcium-activated channel, subfamily M, beta member 4 | 2.32 | A_11_P0000025643 | glycerol kinase | -2.41 |
| A_11_P0000027122 | protein disulfide isomerase family A, member 4 | 2.31 | A_11_P0000010878 |  | -2.41 |
| A_11_P0000025537 | angiopoietin-like 2 | 2.31 | A_11_P062426 | myosin IE | -2.41 |
| A_11_P122486 |  | 2.31 | A_11_P138206 |  | -2.41 |
| A_11_P122696 |  | 2.31 | A_11_P000008233 |  | -2.41 |
| A_11_P108031 |  | 2.31 | A_11_P0000019500 |  | -2.41 |
| A_11_P0000011980 |  | 2.31 | A_11_P0000029495 | 5-beta-cholestane-3-alpha,7-alpha-diol 12-alpha-hydroxylase-like | -2.41 |
| A_11_P0000040266 | zinc finger protein 175-like | 2.31 | A_11_P069561 | phosphatidylinositol-4-phosphate 3-kinase, catalytic subunit type 2 alpha | -2.41 |
| A_11_P146113 |  | 2.31 | A_11_P0000019670 | endothelin 3 | -2.41 |
| A_11_P188530 | nucleosome assembly protein 1-like 1 | 2.31 | A_11_P164808 |  | -2.41 |
| A_11_P0000020194 | KIT ligand | 2.31 | A_11_P0000019933 | ATP-binding cassette, sub-family B (MDR/TAP), member 1 | -2.41 |
| A_11_P0000022922 |  | 2.31 | A_11_P0000030767 | ADP-ribosylation factor-like 5B | -2.41 |
| A_11_P173393 | EGF containing fibulin-like extracellular matrix protein 1 | 2.31 | A_11_P132976 |  | -2.41 |
| A_11_P147268 | collagen, type I, alpha 2 | 2.31 | A_11_P0000040101 |  | -2.41 |
| A_11_P0000032690 | major facilitator superfamily domain containing 6-like | 2.30 | A_11_P0000013152 |  | -2.41 |
| A_11_P059371 |  | 2.30 | A_11_P0000033090 |  | -2.41 |
| A_11_P0000032918 | family with sequence similarity 65, member A | 2.30 | A_11_P091041 |  | -2.41 |
| A_11_P067301 | syntrophin, alpha 1 | 2.30 | A_11_P0000016662 |  | -2.41 |
| A_11_P0000023190 | carbonic anhydrase II | 2.30 | A_11_P0000041347 |  | -2.41 |
| A_11_P0000040688 |  | 2.30 | A_11_P0000024544 | enoyl CoA hydratase domain containing 2 | -2.41 |
| A_11_P0000029078 | nuclear factor I/C (CCAAT-binding transcription factor) | 2.30 | A_11_P0000032738 | UDP-N-acetylglucosamine/UDP-glucose/GDP-mannose transporter-like | -2.41 |
| A_11_P0000021785 |  | 2.30 | A_11_P000002447 |  | -2.40 |
| A_11_P0000017137 |  | 2.30 | A_11_P0000032810 | ERBB receptor feedback inhibitor 1 | -2.40 |
| A_11_P0000023133 | HtrA serine peptidase 1 | 2.30 | A_11_P179448 |  | -2.40 |
| A_11_P060741 |  | 2.30 | A_11_P00000580 | myotubularin related protein 11 | -2.40 |
| A_11_P00000115 | very low density lipoprotein receptor | 2.29 | A_11_P000001186 |  | -2.40 |
| A_11_P119216 |  | 2.29 | A_11_P053261 | angiotensin I converting enzyme (peptidyl-dipeptidase A) 2 | -2.40 |
| A_11_P0000021071 | ribosomal protein S8 | 2.29 | A_11_P000005395 | chromosome 8 open reading frame, human C14orf37 | -2.40 |
| A_11_P180838 |  | 2.29 | A_11_P194218 |  | -2.40 |
| A_11_P0000029573 | COMM domain containing 2 | 2.29 | A_11_P0000041019 | annexin A7 | -2.40 |
| A_11_P0000017231 | GDNF family receptor alpha 2 | 2.29 | A_11_P0000015376 |  | -2.40 |
| A_11_P108026 | centromere protein N | 2.29 | A_11_P0000033870 | exocyst complex component 3-like 4 | -2.40 |
| A_11_P0000021281 | nei endonuclease VIII-like 3 (E. coli) | 2.29 | A_11_P0000041405 |  | -2.40 |
| A_11_P102846 | lysophosphatidylcholine acyltransferase 2 | 2.29 | A_11_P0000026914 | small ArfGAP2 | -2.40 |
| A_11_P174658 | zinc finger protein 576 | 2.29 | A_11_P0000027208 | ring finger protein 32 | -2.39 |
| A_11_P000009336 |  | 2.29 | A_11_P214023 | leucine rich repeat containing 8 family, member A | -2.39 |
| A_11_P202673 |  | 2.29 | A_11_P152338 | calpastatin | -2.39 |
| A_11_P163223 |  | 2.29 | A_11_P0000010515 |  | -2.39 |
| A_11_P166743 |  | 2.29 | A_11_P0000031012 | ring finger protein 186 | -2.39 |
| A_11_P0000016236 |  | 2.29 | A_11_P0000017390 |  | -2.39 |
| A_11_P125586 |  | 2.28 | A_11_P137881 | APC membrane recruitment protein 1 | -2.39 |
| A_11_P120981 | growth associated protein 43 | 2.28 | A_11_P104326 | annexin A7 | -2.39 |
| A_11_P122191 | family with sequence similarity 129, member A | 2.28 | A_11_P0000033544 | phosphatidylinositol 3-kinase, catalytic subunit type 3 | -2.39 |
| A_11_P201503 | solute carrier family 24 (sodium/potassium/calcium exchanger), member 3 | 2.28 | A_11_P0000028727 |  | -2.39 |
| A_11_P0000023496 | solute carrier family 27 (fatty acid transporter), member 2 | 2.28 | A_11_P059881 |  | -2.39 |
| A_11_P0000023635 | Rho GTPase activating protein 24 | 2.28 | A_11_P072267 | AMMECR1-like | -2.39 |
| A_11_P121201 |  | 2.28 | A_11_P071406 | NIMA-related kinase 10 | -2.39 |
| A_11_P0000023194 | receptor-interacting serine-threonine kinase 2 | 2.28 | A_11_P0000039833 | lectin, galactoside-binding-like | -2.39 |
| A_11_P0000040180 |  | 2.28 | A_11_P000008060 |  | -2.39 |
| A_11_P0000016564 | syndecan 3 | 2.28 | A_11_P0000010845 |  | -2.39 |
| A_11_P090191 | EGF containing fibulin-like extracellular matrix protein 1 | 2.28 | A_11_P0000023422 |  | -2.39 |
| A_11_P0000021253 | tumor suppressor candidate 3 | 2.28 | A_11_P112491 | electron-transfer-flavoprotein, beta polypeptide | -2.39 |
| A_11_P0000020687 | polymerase (RNA) I polypeptide C, 30kDa | 2.28 | A_11_P086466 |  | -2.39 |
| A_11_P0000027580 | reelin | 2.28 | A_11_P071666 | protein phosphatase 2, regulatory subunit B'', alpha | -2.38 |
| A_11_P058181 |  | 2.28 | A_11_P086001 |  | -2.38 |
| A_11_P0000027 | synuclein, alpha interacting protein | 2.27 | A_11_P166698 |  | -2.38 |
| A_11_P176288 | sepiapterin reductase (7,8-dihydrobiopterin:NADP+ oxidoreductase) | 2.27 | A_11_P190833 | ataxin 7 | -2.38 |
| A_11_P053226 | ribosomal protein L13a | 2.27 | A_11_P0000031114 | transglutaminase 7 | -2.38 |
| A_11_P0000030399 | matrix Gla protein | 2.27 | A_11_P00000766 | mitogen-activated protein kinase 6 | -2.38 |
| A_11_P0000039041 |  | 2.27 | A_11_P169098 | 3-oxo-5-beta-steroid 4-dehydrogenase-like | -2.38 |
| A_11_P172503 | dual specificity phosphatase 7 | 2.27 | A_11_P084276 | solute carrier family 45, member 4 | -2.38 |
| A_11_P00000914 | complement component 3 | 2.27 | A_11_P145218 |  | -2.38 |
| A_11_P138396 |  | 2.27 | A_11_P0000010753 |  | -2.38 |
| A_11_P053646 |  | 2.27 | A_11_P0000019308 |  | -2.38 |
| A_11_P0000031402 | ELOVL fatty acid elongase 6 | 2.27 | A_11_P0000011706 |  | -2.38 |
| A_11_P078326 | RNA binding protein with multiple splicing | 2.27 | A_11_P061511 |  | -2.38 |
| A_11_P148153 | leucine zipper transcription factor-like 1 | 2.27 | A_11_P215423 |  | -2.38 |
| A_11_P0000016672 | serglycin | 2.27 | A_11_P196438 | jumonji, AT rich interactive domain 2 | -2.38 |
| A_11_P0000023797 | replication factor C (activator 1) 4, 37kDa | 2.27 | A_11_P000007153 |  | -2.38 |
| A_11_P127081 |  | 2.27 | A_11_P071401 |  | -2.38 |
| A_11_P092416 |  | 2.26 | A_11_P158193 |  | -2.37 |
| A_11_P215868 |  | 2.26 | A_11_P140531 | xin actin-binding repeat containing 1 | -2.37 |
| A_11_P119176 | zinc finger, AN1-type domain 2A | 2.26 | A_11_P0000016936 |  | -2.37 |
| A_11_P059331 |  | 2.26 | A_11_P0000015052 |  | -2.37 |
| A_11_P000005869 |  | 2.26 | A_11_P0000015133 |  | -2.37 |
| A_11_P0000023712 | pleckstrin homology-like domain, family B, member 2 | 2.26 | A_11_P000009308 |  | -2.37 |
| A_11_P00000467 | runt-related transcription factor 1; translocated to, 1 (cyclin D-related) | 2.26 | A_11_P0000020347 | DnaJ (Hsp40) homolog, subfamily B, member 7 | -2.37 |
| A_11_P070481 | lysyl oxidase-like 2 | 2.26 | A_11_P00000588 | coiled-coil domain containing 172 | -2.37 |
| A_11_P000006465 |  | 2.26 | A_11_P051166 | peroxisome proliferator-activated receptor gamma | -2.37 |
| A_11_P171588 |  | 2.26 | A_11_P0000010514 |  | -2.37 |
| A_11_P116226 | deoxyribonuclease I-like 1 | 2.26 | A_11_P000009869 |  | -2.37 |
| A_11_P212538 | zinc finger protein 428 | 2.26 | A_11_P182028 | kinesin heavy chain member 2A | -2.37 |
| A_11_P0000035125 | endothelial cell surface expressed chemotaxis and apoptosis regulator | 2.26 | A_11_P184563 | HERPUD family member 2 | -2.37 |
| A_11_P0000022620 | phosphatidylinositol glycan anchor biosynthesis, class T | 2.26 | A_11_P0000030343 | twinfilin actin-binding protein 1 | -2.37 |
| A_11_P0000017141 | uronyl-2-sulfotransferase | 2.26 | A_11_P0000024169 | phosphatidylinositol 4-kinase type 2 beta | -2.37 |
| A_11_P050621 | non-metastatic cells 1, protein (NM23A) expressed in | 2.26 | A_11_P0000033507 | zinc finger and BTB domain containing 7B | -2.37 |
| A_11_P118131 |  | 2.26 | A_11_P071311 | SEC22 vesicle trafficking protein homolog C (S. cerevisiae) | -2.37 |
| A_11_P0000023706 | intraflagellar transport 57 homolog (Chlamydomonas) | 2.25 | A_11_P0000016970 |  | -2.37 |
| A_11_P0000022178 | growth arrest and DNA-damage-inducible, gamma interacting protein 1 | 2.25 | A_11_P0000039584 |  | -2.36 |
| A_11_P0000039106 | transmembrane protein 132A | 2.25 | A_11_P050966 | ATP-binding cassette, sub-family C (CFTR/MRP), member 5 | -2.36 |
| A_11_P076561 | peroxiredoxin 2 | 2.25 | A_11_P0000028562 | carcinoembryonic antigen-related cell adhesion molecule 1 | -2.36 |
| A_11_P152478 | heat shock 70kDa protein 5 (glucose-regulated protein, 78kDa) | 2.25 | A_11_P0000039481 |  | -2.36 |
| A_11_P204653 | prostaglandin E synthase 2 | 2.25 | A_11_P189163 |  | -2.36 |
| A_11_P0000016904 |  | 2.25 | A_11_P0000033516 | ATPase, aminophospholipid transporter, class I, type 8B, member 2 | -2.36 |
| A_11_P218983 |  | 2.25 | A_11_P139711 |  | -2.36 |
| A_11_P094101 |  | 2.25 | A_11_P000009183 |  | -2.36 |
| A_11_P0000014508 | ring finger protein 125, E3 ubiquitin protein ligase | 2.25 | A_11_P000009952 |  | -2.36 |
| A_11_P0000041280 | myosin IC | 2.25 | A_11_P0000029896 | prepronociceptin | -2.36 |
| A_11_P000005837 |  | 2.25 | A_11_P140731 |  | -2.36 |
| A_11_P0000039909 |  | 2.24 | A_11_P0000026259 | transmembrane protein 38B | -2.36 |
| A_11_P152468 | protein arginine methyltransferase 5 | 2.24 | A_11_P0000016279 |  | -2.36 |
| A_11_P050891 | flavin containing monooxygenase 1 | 2.24 | A_11_P0000030456 | anoctamin 2 | -2.36 |
| A_11_P0000015897 | peptidylprolyl isomerase F | 2.24 | A_11_P127456 |  | -2.36 |
| A_11_P0000033539 | solute carrier family 14 (urea transporter), member 1 | 2.24 | A_11_P0000019570 |  | -2.36 |
| A_11_P0000022533 | destrin (actin depolymerizing factor) | 2.24 | A_11_P0000013530 |  | -2.36 |
| A_11_P0000018732 | dephospho-CoA kinase domain containing | 2.24 | A_11_P000008028 |  | -2.36 |
| A_11_P0000019852 | prostaglandin D2 synthase 21kDa (brain) | 2.24 | A_11_P0000033256 | solute carrier family 25 (mitochondrial carrier; phosphate carrier), member 24 | -2.36 |
| A_11_P0000030200 | kelch-like family member 22 | 2.24 | A_11_P0000019364 |  | -2.35 |
| A_11_P118826 | dickkopf WNT signaling pathway inhibitor 3 | 2.24 | A_11_P0000030970 | uncharacterized LOC612166 | -2.35 |
| A_11_P0000024687 | thyroid hormone receptor interactor 6 | 2.24 | A_11_P0000022837 |  | -2.35 |
| A_11_P0000014532 |  | 2.24 | A_11_P0000017796 |  | -2.35 |
| A_11_P0000021031 | mitochondrial ribosomal protein S15 | 2.24 | A_11_P136606 |  | -2.35 |
| A_11_P0000028216 | t-complex 1 | 2.24 | A_11_P165498 |  | -2.35 |
| A_11_P143643 | sepiapterin reductase (7,8-dihydrobiopterin:NADP+ oxidoreductase) | 2.24 | A_11_P060011 | 2'-5'-oligoadenylate synthetase 3, 100kDa | -2.35 |
| A_11_P115591 | plastin 3 | 2.24 | A_11_P206183 |  | -2.35 |
| A_11_P149998 |  | 2.24 | A_11_P164673 | cytochrome P450, family 27, subfamily A, polypeptide 1 | -2.35 |
| A_11_P0000022407 | collagen, type IV, alpha 2 | 2.24 | A_11_P075881 | 6-phosphofructo-2-kinase/fructose-2,6-biphosphatase 4 | -2.35 |
| A_11_P054698 |  | 2.24 | A_11_P0000015170 | lipin 2 | -2.35 |
| A_11_P0000040500 | aldehyde dehydrogenase 2 family (mitochondrial) | 2.24 | A_11_P000008536 |  | -2.35 |
| A_11_P060061 | RNA binding motif protein 19 | 2.24 | A_11_P0000030015 | G protein-coupled receptor 133 | -2.35 |
| A_11_P0000015737 | transmembrane protein 109 | 2.24 | A_11_P190088 |  | -2.35 |
| A_11_P111721 | CDC28 protein kinase regulatory subunit 2 | 2.23 | A_11_P000009801 |  | -2.35 |
| A_11_P000003922 | synaptotagmin binding, cytoplasmic RNA interacting protein | 2.23 | A_11_P0000014001 |  | -2.35 |
| A_11_P067486 | SAM domain and HD domain 1 | 2.23 | A_11_P063936 | clarin 3 | -2.35 |
| A_11_P207656 | guanine nucleotide binding protein (G protein), beta polypeptide 2-like 1 | 2.23 | A_11_P0000038402 |  | -2.35 |
| A_11_P0000016767 | transmembrane protein 206 | 2.23 | A_11_P052146 | olfactory receptor-like protein OLF4-like | -2.35 |
| A_11_P092841 | FK506 binding protein 3, 25kDa | 2.23 | A_11_P187688 | spectrin, alpha, non-erythrocytic 1 | -2.35 |
| A_11_P078401 | tumor suppressor candidate 3 | 2.23 | A_11_P181943 |  | -2.35 |
| A_11_P050916 | angiopoietin 2 | 2.23 | A_11_P201858 |  | -2.35 |
| A_11_P143758 | serum amyloid A1 | 2.23 | A_11_P0000041963 |  | -2.34 |
| A_11_P060016 | RAS protein activator like 1 (GAP1 like) | 2.23 | A_11_P0000031922 | cathepsin E | -2.34 |
| A_11_P192818 | annexin A1 | 2.23 | A_11_P0000039893 |  | -2.34 |
| A_11_P0000024914 | cystathionase (cystathionine gamma-lyase) | 2.23 | A_11_P0000016706 |  | -2.34 |
| A_11_P000004561 | protein kinase D3 | 2.23 | A_11_P0000028872 | pyroglutamyl-peptidase I | -2.34 |
| A_11_P214788 | DEAD (Asp-Glu-Ala-Asp) box polypeptide 10 | 2.23 | A_11_P0000032239 | mixed lineage kinase 4 | -2.34 |
| A_11_P0000017195 | StAR-related lipid transfer (START) domain containing 8 | 2.23 | A_11_P0000040413 |  | -2.34 |
| A_11_P157193 | uncharacterized LOC475115 | 2.23 | A_11_P0000028597 | zinc finger protein 36, C3H type, homolog (mouse) | -2.34 |
| A_11_P221423 | transcription elongation factor A (SII)-like 1 | 2.23 | A_11_P203213 | tudor domain containing 7 | -2.34 |
| A_11_P0000027439 | methylenetetrahydrofolate dehydrogenase (NADP+ dependent) 2, methenyltetrahydrofolate cyclohydrolase | 2.23 | A_11_P215153 | mitochondrial fission factor | -2.34 |
| A_11_P0000041830 | teneurin transmembrane protein 4 | 2.23 | A_11_P160628 | catsper channel auxiliary subunit gamma | -2.34 |
| A_11_P158593 |  | 2.22 | A_11_P0000026168 |  | -2.34 |
| A_11_P157498 |  | 2.22 | A_11_P214143 | ankyrin repeat domain 17 | -2.34 |
| A_11_P195013 |  | 2.22 | A_11_P0000026996 |  | -2.34 |
| A_11_P064091 | mitochondrial ribosomal protein L15 | 2.22 | A_11_P181378 |  | -2.34 |
| A_11_P101696 | plexin domain containing 2 | 2.22 | A_11_P090481 | MAX dimerization protein 1 | -2.34 |
| A_11_P0000030334 | histone deacetylase 7 | 2.22 | A_11_P0000012969 |  | -2.34 |
| A_11_P118916 | melanoma cell adhesion molecule | 2.22 | A_11_P142353 |  | -2.33 |
| A_11_P0000018740 | collagen beta(1-O)galactosyltransferase 1 | 2.22 | A_11_P180153 |  | -2.33 |
| A_11_P184428 |  | 2.22 | A_11_P170688 |  | -2.33 |
| A_11_P0000022558 | NOP56 ribonucleoprotein | 2.22 | A_11_P168498 |  | -2.33 |
| A_11_P072226 | neuron-derived neurotrophic factor | 2.22 | A_11_P098746 | basic leucine zipper transcription factor, ATF-like 3 | -2.33 |
| A_11_P153898 | heat shock 60kDa protein 1 (chaperonin) | 2.22 | A_11_P117426 |  | -2.33 |
| A_11_P142448 | 5-hydroxytryptamine (serotonin) receptor 7, adenylate cyclase-coupled | 2.22 | A_11_P000008090 |  | -2.33 |
| A_11_P152998 | eukaryotic translation initiation factor 2, subunit 1 alpha, 35kDa | 2.22 | A_11_P213843 | family with sequence similarity 71, member D | -2.33 |
| A_11_P128278 | eukaryotic translation elongation factor 1 alpha 1 | 2.22 | A_11_P109941 | slit homolog 2 (Drosophila) | -2.33 |
| A_11_P185793 |  | 2.22 | A_11_P0000010367 |  | -2.33 |
| A_11_P165488 |  | 2.22 | A_11_P172253 |  | -2.33 |
| A_11_P074271 | barrier to autointegration factor 1 | 2.22 | A_11_P0000012003 |  | -2.33 |
| A_11_P0000037506 |  | 2.22 | A_11_P144033 |  | -2.33 |
| A_11_P139571 |  | 2.22 | A_11_P000007559 |  | -2.33 |
| A_11_P0000014231 | hemoglobin subunit alpha-like | 2.22 | A_11_P000009145 |  | -2.33 |
| A_11_P137741 | nucleoporin 88kDa | 2.22 | A_11_P168703 |  | -2.33 |
| A_11_P178108 | N-terminal EF-hand calcium binding protein 1 | 2.22 | A_11_P120136 | mesoderm induction early response 1, family member 3 | -2.33 |
| A_11_P173053 |  | 2.21 | A_11_P000006807 |  | -2.33 |
| A_11_P0000040194 |  | 2.21 | A_11_P000001329 |  | -2.33 |
| A_11_P118991 |  | 2.21 | A_11_P0000011806 |  | -2.33 |
| A_11_P183398 | ribosomal protein L8 | 2.21 | A_11_P0000031851 |  | -2.33 |
| A_11_P0000014456 |  | 2.21 | A_11_P000007788 |  | -2.33 |
| A_11_P119566 | lipopolysaccharide-induced TNF factor | 2.21 | A_11_P0000041393 |  | -2.33 |
| A_11_P0000010198 |  | 2.21 | A_11_P0000040214 |  | -2.33 |
| A_11_P0000038981 | platelet derived growth factor D | 2.21 | A_11_P0000023572 | ubiquitin specific peptidase 25 | -2.33 |
| A_11_P109221 | isocitrate dehydrogenase 2 (NADP+), mitochondrial | 2.21 | A_11_P183633 |  | -2.33 |
| A_11_P0000019995 | chemokine (C-X-C motif) receptor 7 | 2.21 | A_11_P0000028249 | minichromosome maintenance complex component 9 | -2.33 |
| A_11_P00000389 | RNA binding motif protein, X-linked | 2.21 | A_11_P091386 | runt-related transcription factor 2 | -2.33 |
| A_11_P0000038846 |  | 2.21 | A_11_P0000041762 |  | -2.33 |
| A_11_P0000015239 |  | 2.21 | A_11_P0000012410 |  | -2.32 |
| A_11_P119461 |  | 2.21 | A_11_P204438 | potassium channel, subfamily K, member 5 | -2.32 |
| A_11_P118441 | malic enzyme 1, NADP(+)-dependent, cytosolic | 2.21 | A_11_P158598 |  | -2.32 |
| A_11_P168828 |  | 2.21 | A_11_P0000024159 | family with sequence similarity 114, member A1 | -2.32 |
| A_11_P0000040664 |  | 2.21 | A_11_P0000025367 |  | -2.32 |
| A_11_P126441 | fibronectin leucine rich transmembrane protein 3 | 2.21 | A_11_P0000031663 | neural precursor cell expressed, developmentally down-regulated 9 | -2.32 |
| A_11_P0000021081 | poly (ADP-ribose) polymerase 2 | 2.21 | A_11_P0000025415 |  | -2.32 |
| A_11_P0000028813 | sema domain, immunoglobulin domain (Ig), short basic domain, secreted, (semaphorin) 3F | 2.21 | A_11_P180208 | neighbor of BRCA1 gene 1 | -2.32 |
| A_11_P131231 | DnaJ (Hsp40) homolog, subfamily A, member 3 | 2.20 | A_11_P0000032805 |  | -2.32 |
| A_11_P053661 | ribosomal protein L19 | 2.20 | A_11_P181303 | olfactory receptor-like | -2.32 |
| A_11_P166048 | Dab, mitogen-responsive phosphoprotein, homolog 2 (Drosophila) | 2.20 | A_11_P0000029847 | GS homeobox 1 | -2.32 |
| A_11_P060756 |  | 2.20 | A_11_P0000016505 | mitogen-activated protein kinase kinase 3 | -2.32 |
| A_11_P107191 | EF-hand calcium binding domain 7 | 2.20 | A_11_P0000025687 | HECT, UBA and WWE domain containing 1, E3 ubiquitin protein ligase | -2.32 |
| A_11_P0000022895 | RAN binding protein 1 | 2.20 | A_11_P0000026887 | solute carrier family 13 (sodium/sulfate symporter), member 1 | -2.32 |
| A_11_P223123 | CDC42 effector protein (Rho GTPase binding) 3 | 2.20 | A_11_P0000040222 |  | -2.32 |
| A_11_P0000034923 | pregnancy up-regulated non-ubiquitously expressed CaM kinase | 2.20 | A_11_P0000040110 |  | -2.32 |
| A_11_P195190 |  | 2.20 | A_11_P000004885 |  | -2.32 |
| A_11_P113391 |  | 2.20 | A_11_P209178 | karyopherin alpha 4 (importin alpha 3) | -2.32 |
| A_11_P132586 | phosphoribosyl transferase domain containing 1 | 2.20 | A_11_P214673 |  | -2.32 |
| A_11_P150178 | MAP/microtubule affinity-regulating kinase 1 | 2.20 | A_11_P183613 | zinc finger protein 513 | -2.32 |
| A_11_P0000014995 |  | 2.20 | A_11_P082721 | abhydrolase domain containing 15 | -2.32 |
| A_11_P000006353 | selenoprotein W, 1 | 2.20 | A_11_P057251 |  | -2.32 |
| A_11_P0000021437 |  | 2.20 | A_11_P0000015257 |  | -2.31 |
| A_11_P0000015406 | microtubule-associated protein 2 | 2.20 | A_11_P0000033002 |  | -2.31 |
| A_11_P0000017974 |  | 2.20 | A_11_P0000025709 | choroideremia (Rab escort protein 1) | -2.31 |
| A_11_P0000041150 |  | 2.20 | A_11_P200543 |  | -2.31 |
| A_11_P168798 | membrane-associated ring finger (C3HC4) 9 | 2.20 | A_11_P217593 |  | -2.31 |
| A_11_P095476 |  | 2.20 | A_11_P0000011530 |  | -2.31 |
| A_11_P156183 | peptidylprolyl isomerase B (cyclophilin B) | 2.20 | A_11_P108616 | elongation factor, RNA polymerase II, 2 | -2.31 |
| A_11_P215848 | A kinase (PRKA) anchor protein 2 | 2.19 | A_11_P0000015564 |  | -2.31 |
| A_11_P088381 |  | 2.19 | A_11_P155513 | 1-acylglycerol-3-phosphate O-acyltransferase 9 | -2.31 |
| A_11_P0000017174 | solute carrier family 1 (glutamate/neutral amino acid transporter), member 4 | 2.19 | A_11_P0000032881 | cap methyltransferase 2 | -2.31 |
| A_11_P213428 | guanine nucleotide binding protein (G protein), beta polypeptide 2-like 1 | 2.19 | A_11_P000008045 |  | -2.31 |
| A_11_P0000025666 | FtsJ RNA methyltransferase homolog 1 (E. coli) | 2.19 | A_11_P181338 |  | -2.31 |
| A_11_P127788 | chaperonin containing TCP1, subunit 6A (zeta 1) | 2.19 | A_11_P0000029836 |  | -2.31 |
| A_11_P160123 | reticulon 4 interacting protein 1 | 2.19 | A_11_P0000019009 |  | -2.31 |
| A_11_P0000014206 |  | 2.19 | A_11_P0000031342 | sosondowah ankyrin repeat domain family member B | -2.31 |
| A_11_P050076 | dynein, light chain, Tctex-type 3 | 2.19 | A_11_P066906 | adrenergic, alpha-1D-, receptor | -2.31 |
| A_11_P108976 | myotubularin related protein 10 | 2.19 | A_11_P000004943 |  | -2.31 |
| A_11_P193933 |  | 2.19 | A_11_P195798 |  | -2.31 |
| A_11_P137961 | 3'-phosphoadenosine 5'-phosphosulfate synthase 1 | 2.19 | A_11_P127176 |  | -2.31 |
| A_11_P125436 | enolase 2 (gamma, neuronal) | 2.19 | A_11_P0000010040 |  | -2.31 |
| A_11_P0000020209 | vitamin K-dependent protein C-like | 2.19 | A_11_P0000030548 | stearoyl-CoA desaturase (delta-9-desaturase) | -2.31 |
| A_11_P00000281 | EBNA1 binding protein 2 | 2.19 | A_11_P087636 | tetratricopeptide repeat domain 31 | -2.31 |
| A_11_P066046 | fatty acid binding protein 5 (psoriasis-associated) | 2.19 | A_11_P201223 | aquaporin 8 | -2.30 |
| A_11_P203718 | ubiquitin-conjugating enzyme E2S | 2.19 | A_11_P0000032858 | gigaxonin | -2.30 |
| A_11_P155028 | moesin | 2.18 | A_11_P181458 | chymotrypsin-like elastase family, member 3B | -2.30 |
| A_11_P188138 |  | 2.18 | A_11_P0000020232 | cOR13F4 olfactory receptor family 13 subfamily F-like | -2.30 |
| A_11_P0000023189 |  | 2.18 | A_11_P0000021827 | tRNA methyltransferase 11 homolog (S. cerevisiae) | -2.30 |
| A_11_P111596 | very low density lipoprotein receptor | 2.18 | A_11_P00000302 |  | -2.30 |
| A_11_P115926 | four and a half LIM domains 1 | 2.18 | A_11_P156528 | membrane-associated ring finger (C3HC4) 7, E3 ubiquitin protein ligase | -2.30 |
| A_11_P096581 | vitamin K epoxide reductase complex, subunit 1 | 2.18 | A_11_P0000022068 | chromosome 20 open reading frame, human C3orf67 | -2.30 |
| A_11_P0000013744 |  | 2.18 | A_11_P0000040133 | SIN3 transcription regulator family member B | -2.30 |
| A_11_P064531 |  | 2.18 | A_11_P0000015184 |  | -2.30 |
| A_11_P111041 | anti-silencing function 1A histone chaperone | 2.18 | A_11_P0000025545 | golgin A1 | -2.30 |
| A_11_P0000014881 | phosphoribosyl transferase domain containing 1 | 2.18 | A_11_P139741 |  | -2.30 |
| A_11_P113301 | exosome component 5 | 2.18 | A_11_P061441 |  | -2.30 |
| A_11_P0000040637 | androgen-induced 1 | 2.18 | A_11_P0000022306 | transmembrane protein 41B | -2.30 |
| A_11_P106456 | complement component 1, q subcomponent binding protein | 2.18 | A_11_P092676 | sec1 family domain containing 1 | -2.30 |
| A_11_P0000029408 | SLIT and NTRK-like family, member 6 | 2.18 | A_11_P064721 | PAP associated domain containing 7 | -2.30 |
| A_11_P098371 | cystathionase (cystathionine gamma-lyase) | 2.18 | A_11_P0000013820 |  | -2.30 |
| A_11_P0000025529 | prostaglandin E synthase 2 | 2.18 | A_11_P0000030168 | ureidopropionase, beta | -2.30 |
| A_11_P0000022691 | arachidonate 5-lipoxygenase-activating protein | 2.18 | A_11_P0000023165 | UBX domain protein 2B | -2.30 |
| A_11_P0000029722 |  | 2.18 | A_11_P0000041911 |  | -2.30 |
| A_11_P221838 | coiled-coil-helix-coiled-coil-helix domain containing 6 | 2.18 | A_11_P146418 |  | -2.30 |
| A_11_P0000024955 | ring finger protein 2 | 2.18 | A_11_P0000025824 | SH3 and cysteine rich domain 3 | -2.30 |
| A_11_P00000694 |  | 2.18 | A_11_P0000039469 |  | -2.30 |
| A_11_P0000022021 | monoglyceride lipase | 2.17 | A_11_P126351 |  | -2.30 |
| A_11_P0000027524 | repetin | 2.17 | A_11_P123456 | elongation factor, RNA polymerase II, 2 | -2.30 |
| A_11_P0000039621 |  | 2.17 | A_11_P133941 |  | -2.30 |
| A_11_P0000016757 | betaine--homocysteine S-methyltransferase 2 | 2.17 | A_11_P200883 |  | -2.30 |
| A_11_P083741 |  | 2.17 | A_11_P051366 | NADH dehydrogenase subunit 6 | -2.30 |
| A_11_P000007746 |  | 2.17 | A_11_P0000040618 |  | -2.30 |
| A_11_P0000032594 | RNA exonuclease 2 | 2.17 | A_11_P0000025692 |  | -2.30 |
| A_11_P122506 | BCL2-associated athanogene 3 | 2.17 | A_11_P0000032781 | pleckstrin homology domain containing, family N member 1 | -2.29 |
| A_11_P131461 | flotillin 2 | 2.17 | A_11_P159778 | homer homolog 3 (Drosophila) | -2.29 |
| A_11_P162488 |  | 2.17 | A_11_P0000020371 | target of myb1 (chicken) | -2.29 |
| A_11_P0000038813 | collagen, type IV, alpha 1 | 2.17 | A_11_P087506 |  | -2.29 |
| A_11_P0000033269 | transmembrane protein 56 | 2.17 | A_11_P0000028842 | chemokine (C-C motif) receptor 9 | -2.29 |
| A_11_P190493 | platelet/endothelial cell adhesion molecule 1 | 2.16 | A_11_P0000041229 |  | -2.29 |
| A_11_P0000039874 |  | 2.16 | A_11_P188738 |  | -2.29 |
| A_11_P0000038948 |  | 2.16 | A_11_P0000019030 |  | -2.29 |
| A_11_P086806 | tumor protein p53 inducible protein 3 | 2.16 | A_11_P153933 | ERBB receptor feedback inhibitor 1 | -2.29 |
| A_11_P063241 | ankyrin repeat domain 2 (stretch responsive muscle) | 2.16 | A_11_P0000027473 | mannosidase, alpha, class 1A, member 2 | -2.29 |
| A_11_P0000040936 |  | 2.16 | A_11_P093302 | serine/arginine-rich splicing factor 5 | -2.29 |
| A_11_P0000031998 | regulator of G-protein signaling 5 | 2.16 | A_11_P0000031094 | ras homolog family member V | -2.29 |
| A_11_P0000032571 | melanoma cell adhesion molecule | 2.16 | A_11_P0000039914 |  | -2.29 |
| A_11_P0000020987 |  | 2.16 | A_11_P000007354 |  | -2.29 |
| A_11_P162708 | IMP (inosine 5'-monophosphate) dehydrogenase 2 | 2.16 | A_11_P0000027041 | transmembrane and coiled-coil domain family 3 | -2.29 |
| A_11_P0000016277 |  | 2.16 | A_11_P0000040845 | cadherin 1, type 1, E-cadherin (epithelial) | -2.29 |
| A_11_P052396 | peptide YY | 2.16 | A_11_P0000031144 | KIAA1370 ortholog | -2.29 |
| A_11_P151813 | coenzyme Q10 homolog A (S. cerevisiae) | 2.16 | A_11_P0000041518 |  | -2.29 |
| A_11_P122951 |  | 2.16 | A_11_P0000019385 |  | -2.29 |
| A_11_P0000027639 | jumonji domain containing 6 | 2.16 | A_11_P0000011863 |  | -2.28 |
| A_11_P119896 | FLYWCH family member 2 | 2.16 | A_11_P0000035725 |  | -2.28 |
| A_11_P0000033986 | insulin-like growth factor binding protein 4 | 2.15 | A_11_P208289 | phosphorylase, glycogen, muscle | -2.28 |
| A_11_P059981 |  | 2.15 | A_11_P0000017913 |  | -2.28 |
| A_11_P0000023202 |  | 2.15 | A_11_P0000041749 |  | -2.28 |
| A_11_P0000034777 | brain expressed, X-linked 4 | 2.15 | A_11_P0000024782 | sorting nexin 29 | -2.28 |
| A_11_P064566 | cyclin E2 | 2.15 | A_11_P151943 |  | -2.28 |
| A_11_P0000014 | cyclin-dependent kinase 4 | 2.15 | A_11_P133086 |  | -2.28 |
| A_11_P0000019865 | CD86 molecule | 2.15 | A_11_P217833 |  | -2.28 |
| A_11_P158290 | TBC1 domain family, member 7 | 2.15 | A_11_P0000018997 | XIAP associated factor 1 | -2.28 |
| A_11_P0000020458 | partner of NOB1 homolog (S. cerevisiae) | 2.15 | A_11_P0000021502 | SRSF protein kinase 2 | -2.28 |
| A_11_P000005695 |  | 2.15 | A_11_P054746 | defensin, beta 124 | -2.28 |
| A_11_P0000026424 | minichromosome maintenance complex component 3 | 2.15 | A_11_P0000019267 | transmembrane protein 231 | -2.28 |
| A_11_P066261 | C-type lectin domain family 9, member A | 2.15 | A_11_P0000022097 | aminoacylase 1 | -2.28 |
| A_11_P0000033590 | TGFB-induced factor homeobox 1 | 2.15 | A_11_P0000026833 | membrane protein, palmitoylated 6 (MAGUK p55 subfamily member 6) | -2.28 |
| A_11_P050306 | ubiquitin A-52 residue ribosomal protein fusion product 1 | 2.15 | A_11_P177543 | HERPUD family member 2 | -2.28 |
| A_11_P0000023382 | interferon, alpha-inducible protein 6 | 2.15 | A_11_P164218 | calpain 5 | -2.28 |
| A_11_P076516 | chromosome 20 open reading frame, human C19orf53 | 2.15 | A_11_P0000016590 | V-set and transmembrane domain containing 2 like | -2.28 |
| A_11_P051206 | myxovirus (influenza virus) resistance 1, interferon-inducible protein p78 (mouse) | 2.15 | A_11_P122051 |  | -2.28 |
| A_11_P173688 | receptor tyrosine kinase-like orphan receptor 2 | 2.15 | A_11_P101606 | G protein-coupled receptor 158 | -2.28 |
| A_11_P0000018623 |  | 2.15 | A_11_P000008798 |  | -2.28 |
| A_11_P0000018145 | anterior gradient 2 | 2.15 | A_11_P0000016857 | complement component 1, r subcomponent-like | -2.28 |
| A_11_P0000016537 |  | 2.15 | A_11_P152443 |  | -2.28 |
| A_11_P0000021000 | transcription factor EC | 2.15 | A_11_P000005656 |  | -2.28 |
| A_11_P0000021902 | isochorismatase domain containing 2 | 2.15 | A_11_P081926 |  | -2.28 |
| A_11_P053966 | beta-defensin 108B-like | 2.14 | A_11_P0000017574 |  | -2.28 |
| A_11_P0000031692 | histone H1.1-like | 2.14 | A_11_P196858 | adaptor-related protein complex 2, alpha 1 subunit | -2.28 |
| A_11_P126146 | RAB38, member RAS oncogene family | 2.14 | A_11_P0000030938 | serine incorporator 2 | -2.28 |
| A_11_P0000062 | enoyl CoA hydratase domain containing 1 | 2.14 | A_11_P0000026675 | KIAA1211 ortholog | -2.28 |
| A_11_P110051 | ribosome binding factor A (putative) | 2.14 | A_11_P0000015872 |  | -2.28 |
| A_11_P095226 | sigma non-opioid intracellular receptor 1 | 2.14 | A_11_P140881 | angiopoietin-like 7 | -2.28 |
| A_11_P0000016817 | RAB3A interacting protein (rabin3)-like 1 | 2.14 | A_11_P0000041488 |  | -2.27 |
| A_11_P125541 |  | 2.14 | A_11_P0000015403 |  | -2.27 |
| A_11_P109196 |  | 2.14 | A_11_P0000023380 |  | -2.27 |
| A_11_P182398 | protease-associated domain containing 1 | 2.14 | A_11_P0000027071 | SH3 domain containing 19 | -2.27 |
| A_11_P133191 |  | 2.14 | A_11_P0000039967 | lysine (K)-specific demethylase 4C | -2.27 |
| A_11_P055926 |  | 2.14 | A_11_P0000012766 |  | -2.27 |
| A_11_P152313 | chromosome 26 open reading frame, human C22orf39 | 2.14 | A_11_P0000035152 |  | -2.27 |
| A_11_P0000020395 | interleukin 1 receptor, type II | 2.14 | A_11_P164043 | heterogeneous nuclear ribonucleoprotein A2/B1 | -2.27 |
| A_11_P087466 | CD8b molecule | 2.14 | A_11_P097891 |  | -2.27 |
| A_11_P0000026946 | origin recognition complex, subunit 6 | 2.14 | A_11_P218078 |  | -2.27 |
| A_11_P193588 |  | 2.14 | A_11_P176998 |  | -2.27 |
| A_11_P0000023670 | 3'-phosphoadenosine 5'-phosphosulfate synthase 1 | 2.14 | A_11_P0000038329 |  | -2.27 |
| A_11_P094541 | FK506 binding protein 3, 25kDa | 2.14 | A_11_P103176 |  | -2.27 |
| A_11_P0000041879 |  | 2.14 | A_11_P114091 | glycine receptor, alpha 2 | -2.27 |
| A_11_P154911 | mitochondrial ribosomal protein L45 | 2.14 | A_11_P000007548 |  | -2.27 |
| A_11_P108341 |  | 2.14 | A_11_P0000014038 | target of myb1 (chicken) | -2.27 |
| A_11_P0000033301 | nexilin (F actin binding protein) | 2.14 | A_11_P0000021975 |  | -2.27 |
| A_11_P0000016854 | Rho GTPase activating protein 42 | 2.14 | A_11_P053386 | ATP-binding cassette, sub-family B (MDR/TAP), member 1 | -2.27 |
| A_11_P107076 |  | 2.14 | A_11_P000009647 |  | -2.27 |
| A_11_P085081 | peptidylprolyl isomerase E (cyclophilin E) | 2.14 | A_11_P0000019272 |  | -2.27 |
| A_11_P060456 | selenoprotein M | 2.14 | A_11_P0000041054 | hyaluronan and proteoglycan link protein 4 | -2.27 |
| A_11_P169963 | hydroxysteroid dehydrogenase like 2 | 2.14 | A_11_P134551 |  | -2.27 |
| A_11_P0000023157 | mitochondrial ribosomal protein L15 | 2.14 | A_11_P0000015934 | enoyl CoA hydratase domain containing 2 | -2.27 |
| A_11_P076586 |  | 2.14 | A_11_P196883 |  | -2.26 |
| A_11_P093661 | tyrosyl-DNA phosphodiesterase 1 | 2.13 | A_11_P066466 | pleckstrin homology domain containing, family G (with RhoGef domain) member 6 | -2.26 |
| A_11_P0000016604 | nephronectin | 2.13 | A_11_P0000030490 | chromosome 28 open reading frame, human C10orf71 | -2.26 |
| A_11_P151018 |  | 2.13 | A_11_P0000032362 | sideroflexin 1 | -2.26 |
| A_11_P158878 |  | 2.13 | A_11_P0000029951 | integral membrane protein 2C | -2.26 |
| A_11_P098401 | DEP domain containing 1 | 2.13 | A_11_P0000014965 |  | -2.26 |
| A_11_P057141 | solute carrier family 25 (aspartate/glutamate carrier), member 12 | 2.13 | A_11_P000009237 |  | -2.26 |
| A_11_P0000029516 | thyroid hormone receptor, beta | 2.13 | A_11_P055111 | serine peptidase inhibitor, Kazal type 5 | -2.26 |
| A_11_P000006244 |  | 2.13 | A_11_P138656 | T-box 3 | -2.26 |
| A_11_P0000033089 | coronin, actin binding protein, 1A | 2.13 | A_11_P188233 | transducer of ERBB2, 1 | -2.26 |
| A_11_P105091 | endothelial cell-specific molecule 1 | 2.13 | A_11_P065206 | claudin 11 | -2.26 |
| A_11_P0000021556 | eukaryotic translation initiation factor 3, subunit M | 2.13 | A_11_P0000029880 | hydroxyprostaglandin dehydrogenase 15-(NAD) | -2.26 |
| A_11_P082141 | transmembrane protein 100-like | 2.13 | A_11_P000007859 |  | -2.26 |
| A_11_P153813 | exophilin 5 | 2.13 | A_11_P0000018401 | cadherin 1, type 1, E-cadherin (epithelial) | -2.26 |
| A_11_P0000040917 |  | 2.13 | A_11_P194038 |  | -2.26 |
| A_11_P0000025188 | nidogen 2 (osteonidogen) | 2.13 | A_11_P0000030990 | E2F transcription factor 2 | -2.26 |
| A_11_P172738 | CAP, adenylate cyclase-associated protein, 2 (yeast) | 2.13 | A_11_P075416 |  | -2.26 |
| A_11_P051996 | thymidylate synthetase | 2.13 | A_11_P181663 |  | -2.26 |
| A_11_P0000041800 |  | 2.13 | A_11_P0000023096 | collagen, type XVII, alpha 1 | -2.26 |
| A_11_P103296 | stathmin 1 | 2.13 | A_11_P139766 | regulator of G-protein signaling 3 | -2.26 |
| A_11_P161778 | ST3 beta-galactoside alpha-2,3-sialyltransferase 6 | 2.13 | A_11_P0000040548 |  | -2.26 |
| A_11_P154333 | heat shock 70kDa protein 5 (glucose-regulated protein, 78kDa) | 2.13 | A_11_P122292 | serine/arginine-rich splicing factor 5 | -2.25 |
| A_11_P216048 |  | 2.12 | A_11_P059126 | heparanase | -2.25 |
| A_11_P0000040739 |  | 2.12 | A_11_P0000010350 |  | -2.25 |
| A_11_P0000021616 | ribonuclease H2, subunit C | 2.12 | A_11_P216423 |  | -2.25 |
| A_11_P120121 |  | 2.12 | A_11_P175578 |  | -2.25 |
| A_11_P079096 | STEAP family member 2, metalloreductase | 2.12 | A_11_P173758 |  | -2.25 |
| A_11_P0000017362 | pleckstrin homology domain containing, family A (phosphoinositide binding specific) member 2 | 2.12 | A_11_P000006180 |  | -2.25 |
| A_11_P0000020442 | vaccinia related kinase 2 | 2.12 | A_11_P0000023702 | nuclear factor of kappa light polypeptide gene enhancer in B-cells inhibitor, zeta | -2.25 |
| A_11_P0000022344 | mitochondrial ribosomal protein L16 | 2.12 | A_11_P169568 | synaptotagmin IX | -2.25 |
| A_11_P158093 | solute carrier family 25 (mitochondrial carrier; peroxisomal membrane protein, 34kDa), member 17 | 2.12 | A_11_P128256 | WD repeat domain 44 | -2.25 |
| A_11_P0000034095 | notchless homolog 1 (Drosophila) | 2.12 | A_11_P0000020308 | zinc finger, C3H1-type containing | -2.25 |
| A_11_P196163 |  | 2.12 | A_11_P0000030992 | leucine zipper protein 1 | -2.25 |
| A_11_P0000016964 |  | 2.12 | A_11_P058026 | tubulin tyrosine ligase-like family, member 4 | -2.25 |
| A_11_P158508 | calcitonin receptor-like | 2.12 | A_11_P0000040061 |  | -2.25 |
| A_11_P0000026018 | mutS homolog 2, colon cancer, nonpolyposis type 1 (E. coli) | 2.12 | A_11_P185933 |  | -2.25 |
| A_11_P0000021654 | mitotic spindle assembly checkpoint protein MAD2A-like | 2.12 | A_11_P0000010234 |  | -2.25 |
| A_11_P0000021444 | ankyrin repeat domain 35 | 2.12 | A_11_P090266 | UDP-GlcNAc:betaGal beta-1,3-N-acetylglucosaminyltransferase 2 | -2.25 |
| A_11_P168423 |  | 2.12 | A_11_P058741 | kalirin, RhoGEF kinase | -2.25 |
| A_11_P165788 |  | 2.12 | A_11_P000007941 |  | -2.25 |
| A_11_P171643 | transmembrane protein 198-like | 2.12 | A_11_P0000041796 |  | -2.25 |
| A_11_P0000016927 |  | 2.12 | A_11_P059041 | RasGEF domain family, member 1B | -2.25 |
| A_11_P0000025104 |  | 2.12 | A_11_P0000040197 |  | -2.24 |
| A_11_P0000033799 |  | 2.12 | A_11_P209163 | tudor domain containing 7 | -2.24 |
| A_11_P162613 | ectonucleotide pyrophosphatase/phosphodiesterase 6 | 2.12 | A_11_P0000010120 |  | -2.24 |
| A_11_P0000038947 |  | 2.12 | A_11_P150703 | cyclin-dependent kinase inhibitor 1A (p21, Cip1) | -2.24 |
| A_11_P092226 | germinal center-associated, signaling and motility-like | 2.12 | A_11_P183938 |  | -2.24 |
| A_11_P076526 | nuclear factor I/X (CCAAT-binding transcription factor) | 2.12 | A_11_P065421 |  | -2.24 |
| A_11_P0000039832 |  | 2.11 | A_11_P087241 |  | -2.24 |
| A_11_P155013 |  | 2.11 | A_11_P0000019975 | RAB5C, member RAS oncogene family | -2.24 |
| A_11_P0000024120 | low density lipoprotein receptor-related protein associated protein 1 | 2.11 | A_11_P067281 |  | -2.24 |
| A_11_P061481 | proteasome assembly chaperone 1-like | 2.11 | A_11_P169058 |  | -2.24 |
| A_11_P000003984 | heat shock protein 70kDa family, member 13 | 2.11 | A_11_P0000033830 | potassium channel, subfamily K, member 10 | -2.24 |
| A_11_P0000027693 |  | 2.11 | A_11_P207738 |  | -2.24 |
| A_11_P0000018808 | atlastin GTPase 3 | 2.11 | A_11_P158108 |  | -2.24 |
| A_11_P0000016459 | tyrosine kinase with immunoglobulin-like and EGF-like domains 1 | 2.11 | A_11_P0000010581 |  | -2.24 |
| A_11_P00000853 | early B-cell factor 1 | 2.11 | A_11_P184398 |  | -2.24 |
| A_11_P078461 | N-acylsphingosine amidohydrolase (acid ceramidase) 1 | 2.11 | A_11_P0000016806 | FERM domain containing 8 | -2.24 |
| A_11_P121586 | hydroxysteroid dehydrogenase like 2 | 2.11 | A_11_P136596 |  | -2.24 |
| A_11_P125046 | fibrillin 2 | 2.11 | A_11_P0000018362 | desmocollin 2 | -2.24 |
| A_11_P140316 |  | 2.11 | A_11_P000004510 |  | -2.24 |
| A_11_P093111 | tRNA methyltransferase 5 | 2.11 | A_11_P186763 |  | -2.24 |
| A_11_P000005848 |  | 2.11 | A_11_P069431 | transmembrane protein 41B | -2.24 |
| A_11_P165153 |  | 2.11 | A_11_P093961 |  | -2.24 |
| A_11_P222913 | mitochondrial ribosomal protein L28 | 2.11 | A_11_P0000012331 |  | -2.24 |
| A_11_P193468 |  | 2.11 | A_11_P0000014314 |  | -2.24 |
| A_11_P064381 |  | 2.11 | A_11_P0000019074 |  | -2.24 |
| A_11_P152018 | proteasome (prosome, macropain) 26S subunit, non-ATPase, 3 | 2.11 | A_11_P103226 | zinc finger, DHHC-type containing 18 | -2.23 |
| A_11_P000005676 | sideroflexin 2 | 2.11 | A_11_P127521 |  | -2.23 |
| A_11_P050736 | prohibitin | 2.11 | A_11_P191488 |  | -2.23 |
| A_11_P0000022139 | FYVE and coiled-coil domain containing 1 | 2.10 | A_11_P168633 | family with sequence similarity 40, member A | -2.23 |
| A_11_P224678 |  | 2.10 | A_11_P101921 | proline and serine-rich protein 2 | -2.23 |
| A_11_P121106 |  | 2.10 | A_11_P171568 |  | -2.23 |
| A_11_P0000018603 |  | 2.10 | A_11_P000004455 | lysophosphatidylglycerol acyltransferase 1 | -2.23 |
| A_11_P0000021880 | F-box/WD repeat-containing protein 12-like | 2.10 | A_11_P0000027008 | solute carrier family 39 (zinc transporter), member 2 | -2.23 |
| A_11_P0000024768 | coenzyme Q7 homolog, ubiquinone (yeast) | 2.10 | A_11_P077111 | cAMP responsive element binding protein 3-like 3 | -2.23 |
| A_11_P0000026678 | ADP-ribosylation factor-like 9 | 2.10 | A_11_P090021 | protein phosphatase, Mg2+/Mn2+ dependent, 1B | -2.23 |
| A_11_P064501 | N-terminal EF-hand calcium binding protein 1 | 2.10 | A_11_P071891 | arylacetamide deacetylase | -2.23 |
| A_11_P0000029975 | TNF receptor-associated factor 3 interacting protein 1 | 2.10 | A_11_P203048 | tuftelin interacting protein 11 | -2.23 |
| A_11_P115116 |  | 2.10 | A_11_P083606 | zinc finger and BTB domain containing 43 | -2.23 |
| A_11_P165628 | TGFB-induced factor homeobox 2 | 2.10 | A_11_P052606 | spectrin, beta, non-erythrocytic 1 | -2.23 |
| A_11_P0000024356 | Dab, mitogen-responsive phosphoprotein, homolog 2 (Drosophila) | 2.10 | A_11_P0000014339 | stearoyl-CoA desaturase (delta-9-desaturase) | -2.23 |
| A_11_P065996 | lymphoid-restricted membrane protein | 2.10 | A_11_P107436 | ring finger protein 223 | -2.23 |
| A_11_P147533 | fatty acid binding protein 5 (psoriasis-associated) | 2.10 | A_11_P147159 | TLC domain containing 1 | -2.23 |
| A_11_P058291 | cms1 ribosomal small subunit homolog (yeast) | 2.10 | A_11_P000009306 |  | -2.23 |
| A_11_P177283 |  | 2.10 | A_11_P000009481 |  | -2.23 |
| A_11_P053266 | dystrophin | 2.10 | A_11_P089501 | ras-related C3 botulinum toxin substrate 2 (rho family, small GTP binding protein Rac2) | -2.23 |
| A_11_P0000025405 |  | 2.10 | A_11_P209153 |  | -2.22 |
| A_11_P0000017004 | hippocalcin-like 1 | 2.10 | A_11_P200403 |  | -2.22 |
| A_11_P079976 | nucleophosmin (nucleolar phosphoprotein B23, numatrin) | 2.10 | A_11_P0000020217 | melanocortin 1 receptor (alpha melanocyte stimulating hormone receptor) | -2.22 |
| A_11_P205323 | regulator of G-protein signaling 1 | 2.10 | A_11_P0000026004 | sema domain, immunoglobulin domain (Ig), transmembrane domain (TM) and short cytoplasmic domain, (semaphorin) 4C | -2.22 |
| A_11_P0000027938 | CD248 molecule, endosialin | 2.10 | A_11_P199213 |  | -2.22 |
| A_11_P0000032916 | glucose-fructose oxidoreductase domain containing 2 | 2.10 | A_11_P0000020102 | chemokine (C-C motif) ligand 3 | -2.22 |
| A_11_P0000039290 |  | 2.10 | A_11_P0000040135 | family with sequence similarity 98, member C | -2.22 |
| A_11_P000001019 |  | 2.10 | A_11_P0000034757 | poly(A) binding protein, cytoplasmic 5 | -2.22 |
| A_11_P102426 | superkiller viralicidic activity 2-like 2 (S. cerevisiae) | 2.10 | A_11_P225273 |  | -2.22 |
| A_11_P053726 | lipoprotein lipase | 2.09 | A_11_P181863 | cysteine-serine-rich nuclear protein 1 | -2.22 |
| A_11_P0000032183 | ST8 alpha-N-acetyl-neuraminide alpha-2,8-sialyltransferase 4 | 2.09 | A_11_P0000022709 | intraflagellar transport 88 homolog (Chlamydomonas) | -2.22 |
| A_11_P0000024905 | DnaJ (Hsp40) homolog, subfamily B, member 4 | 2.09 | A_11_P063081 | fibroblast growth factor binding protein 3 | -2.22 |
| A_11_P0000030742 | cleavage stimulation factor, 3' pre-RNA, subunit 2, 64kDa | 2.09 | A_11_P0000027168 | poly (ADP-ribose) polymerase family, member 12 | -2.22 |
| A_11_P0000027257 | protein phosphatase 1, regulatory subunit 3B | 2.09 | A_11_P0000022321 | related RAS viral (r-ras) oncogene homolog 2 | -2.22 |
| A_11_P0000033084 | INO80 complex subunit E | 2.09 | A_11_P00000149 | spectrin, beta, non-erythrocytic 1 | -2.22 |
| A_11_P051631 | butyrylcholinesterase | 2.09 | A_11_P0000014688 |  | -2.22 |
| A_11_P0000012808 |  | 2.09 | A_11_P0000031576 | lipase, member H | -2.22 |
| A_11_P115081 |  | 2.09 | A_11_P000009953 |  | -2.22 |
| A_11_P155563 | UDP-Gal:betaGlcNAc beta 1,4- galactosyltransferase, polypeptide 2 | 2.09 | A_11_P0000013116 |  | -2.22 |
| A_11_P0000028100 | zinc finger E-box binding homeobox 2 | 2.09 | A_11_P0000033627 |  | -2.22 |
| A_11_P0000016571 | peroxiredoxin 4 | 2.09 | A_11_P070756 | S-antigen; retina and pineal gland (arrestin) | -2.22 |
| A_11_P097611 | nth endonuclease III-like 1 (E. coli) | 2.09 | A_11_P0000022918 |  | -2.22 |
| A_11_P160463 |  | 2.09 | A_11_P0000039601 |  | -2.22 |
| A_11_P0000014283 |  | 2.09 | A_11_P072911 | gamma-secretase activating protein | -2.22 |
| A_11_P148193 |  | 2.09 | A_11_P0000033919 | phospholipase C, delta 3 | -2.22 |
| A_11_P0000020582 | Myb/SANT-like DNA-binding domain containing 3 | 2.09 | A_11_P0000033722 | GTP cyclohydrolase 1 | -2.21 |
| A_11_P130976 | ubiquitin family domain containing 1 | 2.09 | A_11_P000009902 |  | -2.21 |
| A_11_P0000013881 |  | 2.09 | A_11_P058361 | nuclear factor of kappa light polypeptide gene enhancer in B-cells inhibitor, zeta | -2.21 |
| A_11_P083746 | matrilin 2 | 2.09 | A_11_P107271 | Myb-like, SWIRM and MPN domains 1 | -2.21 |
| A_11_P185778 | COP9 signalosome subunit 6 | 2.09 | A_11_P0000022129 | plexin B1 | -2.21 |
| A_11_P0000015226 |  | 2.09 | A_11_P0000040654 |  | -2.21 |
| A_11_P0000031272 | MIS18 kinetochore protein homolog A (S. pombe) | 2.09 | A_11_P170803 | TRAF-type zinc finger domain containing 1 | -2.21 |
| A_11_P074321 |  | 2.08 | A_11_P090501 | adducin 2 (beta) | -2.21 |
| A_11_P0000030554 | leucine zipper, putative tumor suppressor 2 | 2.08 | A_11_P099756 | aquaporin 10 | -2.21 |
| A_11_P203943 |  | 2.08 | A_11_P074436 | phosphorylase, glycogen, muscle | -2.21 |
| A_11_P095818 |  | 2.08 | A_11_P000003995 |  | -2.21 |
| A_11_P087096 | nucleophosmin (nucleolar phosphoprotein B23, numatrin) | 2.08 | A_11_P168343 |  | -2.21 |
| A_11_P161513 | ATP-binding cassette, sub-family F (GCN20), member 1 | 2.08 | A_11_P0000034440 |  | -2.21 |
| A_11_P0000028054 | cyclin A2 | 2.08 | A_11_P000006005 |  | -2.21 |
| A_11_P139556 |  | 2.08 | A_11_P081726 | WAS/WASL interacting protein family, member 2 | -2.21 |
| A_11_P080121 | dachshund homolog 1 (Drosophila) | 2.08 | A_11_P158023 |  | -2.21 |
| A_11_P0000022796 | Rab interacting lysosomal protein-like 1 | 2.08 | A_11_P0000018268 |  | -2.21 |
| A_11_P0000016818 | F-box and leucine-rich repeat protein 16 | 2.08 | A_11_P132896 | transmembrane protein 2 | -2.21 |
| A_11_P178028 |  | 2.08 | A_11_P055201 | myosin, heavy chain 3, skeletal muscle, embryonic | -2.21 |
| A_11_P0000016707 |  | 2.08 | A_11_P088246 | thioesterase superfamily member 5 | -2.21 |
| A_11_P00000734 | leucine rich repeat containing 25 | 2.08 | A_11_P0000010008 |  | -2.21 |
| A_11_P081496 | N-acetylglucosaminidase, alpha | 2.08 | A_11_P084416 | kinesin family member C2 | -2.21 |
| A_11_P0000025974 | carbohydrate (chondroitin 4) sulfotransferase 11 | 2.08 | A_11_P080626 | protein phosphatase 1, regulatory subunit 27 | -2.21 |
| A_11_P116671 |  | 2.08 | A_11_P0000028594 | dual-specificity tyrosine-(Y)-phosphorylation regulated kinase 1B | -2.21 |
| A_11_P0000033719 | bone morphogenetic protein 4 | 2.08 | A_11_P122016 | cyclin-dependent kinase inhibitor 1A (p21, Cip1) | -2.21 |
| A_11_P0000017246 |  | 2.08 | A_11_P0000027965 | tigger transposable element derived 3 | -2.21 |
| A_11_P0000015137 |  | 2.08 | A_11_P151273 |  | -2.21 |
| A_11_P098066 | mitochondrial ribosomal protein L45 | 2.08 | A_11_P081531 | heat shock protein, alpha-crystallin-related, B9 | -2.21 |
| A_11_P0000041504 |  | 2.08 | A_11_P053126 | cadherin 1, type 1, E-cadherin (epithelial) | -2.21 |
| A_11_P169323 |  | 2.08 | A_11_P0000040064 |  | -2.21 |
| A_11_P161973 | hydroxysteroid (17-beta) dehydrogenase 7 | 2.08 | A_11_P0000024976 | serpin peptidase inhibitor, clade C (antithrombin), member 1 | -2.21 |
| A_11_P0000031507 | 3-hydroxybutyrate dehydrogenase, type 1 | 2.08 | A_11_P207018 |  | -2.21 |
| A_11_P071426 | retinoic acid receptor, beta | 2.08 | A_11_P078106 | SH2 domain containing 4A | -2.21 |
| A_11_P095426 | N-acetylneuraminic acid synthase | 2.08 | A_11_P170283 | plectin | -2.21 |
| A_11_P0000031831 | chromosome 37 open reading frame, human C2orf69 | 2.08 | A_11_P0000021885 | extracellular matrix protein 2, female organ and adipocyte specific | -2.21 |
| A_11_P204038 |  | 2.08 | A_11_P109451 |  | -2.20 |
| A_11_P050681 | phosphatidylethanolamine binding protein 1 | 2.08 | A_11_P0000015995 |  | -2.20 |
| A_11_P164928 | junctional adhesion molecule 3 | 2.08 | A_11_P151433 | ubiquitin-like modifier activating enzyme 7 | -2.20 |
| A_11_P212583 |  | 2.08 | A_11_P0000017443 |  | -2.20 |
| A_11_P106206 |  | 2.08 | A_11_P0000022976 | ST8 alpha-N-acetyl-neuraminide alpha-2,8-sialyltransferase 1 | -2.20 |
| A_11_P0000024406 | intraflagellar transport 46 homolog (Chlamydomonas) | 2.07 | A_11_P105066 | solute carrier family 26 (anion exchanger), member 2 | -2.20 |
| A_11_P0000024606 |  | 2.07 | A_11_P0000017424 |  | -2.20 |
| A_11_P194363 | serpin peptidase inhibitor, clade A (alpha-1 antiproteinase, antitrypsin), member 1 | 2.07 | A_11_P0000024022 | Mdm4 p53 binding protein homolog (mouse) | -2.20 |
| A_11_P177043 |  | 2.07 | A_11_P163628 |  | -2.20 |
| A_11_P061801 |  | 2.07 | A_11_P000003938 |  | -2.20 |
| A_11_P0000031405 |  | 2.07 | A_11_P061581 | solute carrier family 37 (glucose-6-phosphate transporter), member 1 | -2.20 |
| A_11_P104106 |  | 2.07 | A_11_P202313 | Rho family GTPase 2 | -2.20 |
| A_11_P137196 | calcium channel, voltage-dependent, beta 4 subunit | 2.07 | A_11_P0000040391 |  | -2.20 |
| A_11_P0000032692 | glucagon-like peptide 2 receptor | 2.07 | A_11_P168898 |  | -2.20 |
| A_11_P0000017293 |  | 2.07 | A_11_P173578 | family with sequence similarity 190, member B | -2.20 |
| A_11_P114246 | peroxiredoxin 4 | 2.07 | A_11_P0000030539 | R3H domain and coiled-coil containing 1-like | -2.20 |
| A_11_P0000020178 | major histocompatibility complex, class II, DQ alpha 1 | 2.07 | A_11_P068531 |  | -2.20 |
| A_11_P0000021424 | polyadenylate-binding protein-interacting protein 2B-like | 2.07 | A_11_P114556 | chromosome X open reading frame, human CXorf36 | -2.20 |
| A_11_P163788 |  | 2.07 | A_11_P205223 |  | -2.20 |
| A_11_P191218 | kinase insert domain receptor (a type III receptor tyrosine kinase) | 2.07 | A_11_P074596 |  | -2.20 |
| A_11_P148478 | ribosome production factor 1 homolog (S. cerevisiae) | 2.07 | A_11_P0000025993 |  | -2.20 |
| A_11_P0000038924 |  | 2.07 | A_11_P0000033767 | KIAA0247 ortholog | -2.20 |
| A_11_P123181 | selenoprotein H | 2.07 | A_11_P0000039198 | FK506 binding protein 5 | -2.20 |
| A_11_P120341 |  | 2.07 | A_11_P194923 |  | -2.20 |
| A_11_P0000039847 | integrin, alpha 4 (antigen CD49D, alpha 4 subunit of VLA-4 receptor) | 2.07 | A_11_P184048 |  | -2.20 |
| A_11_P185138 |  | 2.07 | A_11_P0000026406 | GTP binding protein 2 | -2.20 |
| A_11_P126726 |  | 2.07 | A_11_P085496 | leucine rich repeat containing 41 | -2.20 |
| A_11_P124401 | DENN/MADD domain containing 5A | 2.07 | A_11_P168573 | Rho guanine nucleotide exchange factor (GEF) 3 | -2.20 |
| A_11_P0000026840 | homeobox A5 | 2.07 | A_11_P0000010630 |  | -2.19 |
| A_11_P0000041445 |  | 2.07 | A_11_P0000031045 | tumor necrosis factor receptor superfamily, member 1B | -2.19 |
| A_11_P088481 | coenzyme Q10 homolog A (S. cerevisiae) | 2.07 | A_11_P122011 |  | -2.19 |
| A_11_P0000027326 |  | 2.06 | A_11_P110696 | F-box protein 5 | -2.19 |
| A_11_P0000033444 | exonuclease 1 | 2.06 | A_11_P0000039841 | ankyrin repeat and SOCS box containing 14 | -2.19 |
| A_11_P137461 |  | 2.06 | A_11_P212308 | SMAD family member 2 | -2.19 |
| A_11_P0000023243 | phytanoyl-CoA hydroxylase-like | 2.06 | A_11_P0000028300 | transmembrane protein 2 | -2.19 |
| A_11_P148943 |  | 2.06 | A_11_P0000031261 | keratin associated protein 24-1 | -2.19 |
| A_11_P118841 |  | 2.06 | A_11_P0000016175 | 1-acylglycerol-3-phosphate O-acyltransferase 3 | -2.19 |
| A_11_P061891 | BUB1 mitotic checkpoint serine/threonine kinase B | 2.06 | A_11_P0000026702 | Ras association (RalGDS/AF-6) domain family member 6 | -2.19 |
| A_11_P054116 | ribosomal protein L18 | 2.06 | A_11_P0000029778 | prostate transmembrane protein, androgen induced 1 | -2.19 |
| A_11_P0000026837 | src kinase associated phosphoprotein 2 | 2.06 | A_11_P0000024363 | alanine--glyoxylate aminotransferase 2 | -2.19 |
| A_11_P088441 |  | 2.06 | A_11_P183608 |  | -2.19 |
| A_11_P0000020588 | protein NipSnap homolog 3A-like | 2.06 | A_11_P0000022578 |  | -2.19 |
| A_11_P0000021625 | tRNA methyltransferase 11-2 homolog (S. cerevisiae) | 2.06 | A_11_P184648 |  | -2.19 |
| A_11_P179498 | secretogranin II | 2.06 | A_11_P212968 | glutathione reductase | -2.19 |
| A_11_P0000015166 | interferon, gamma-inducible protein 16 | 2.06 | A_11_P165173 | cell death-inducing DFFA-like effector a | -2.19 |
| A_11_P0000018725 | hepatic leukemia factor | 2.06 | A_11_P000004927 |  | -2.19 |
| A_11_P0000024536 | phosphatidic acid phosphatase type 2B | 2.06 | A_11_P084426 | protein phosphatase 1, regulatory subunit 16A | -2.19 |
| A_11_P00000255 | mitochondrial ribosomal protein S7 | 2.06 | A_11_P181628 |  | -2.19 |
| A_11_P0000022305 | DENN/MADD domain containing 5A | 2.06 | A_11_P084901 | albumin | -2.19 |
| A_11_P0000024525 | phosphoglucomutase 1 | 2.06 | A_11_P0000026625 | cysteine/histidine-rich 1 | -2.19 |
| A_11_P000006266 |  | 2.06 | A_11_P172968 |  | -2.19 |
| A_11_P117041 | adenylate cyclase 3 | 2.06 | A_11_P190078 |  | -2.19 |
| A_11_P055681 | retinoid X receptor, gamma | 2.06 | A_11_P0000040291 |  | -2.19 |
| A_11_P0000016689 |  | 2.06 | A_11_P0000032416 | solute carrier family 36 (proton/amino acid symporter), member 1 | -2.18 |
| A_11_P062516 | KIAA0101 ortholog | 2.06 | A_11_P0000025811 | signal transducer and activator of transcription 2, 113kDa | -2.18 |
| A_11_P121461 |  | 2.06 | A_11_P0000027173 | zinc finger CCCH-type, antiviral 1 | -2.18 |
| A_11_P000006111 |  | 2.06 | A_11_P148383 | sterile alpha motif domain containing 4A | -2.18 |
| A_11_P057806 | frizzled family receptor 5 | 2.06 | A_11_P0000023959 | spermatogenesis associated, serine-rich 2-like | -2.18 |
| A_11_P140216 | zinc finger and BTB domain containing 10 | 2.05 | A_11_P0000018368 | shisa family member 5 | -2.18 |
| A_11_P0000029165 | teneurin transmembrane protein 4 | 2.05 | A_11_P115466 |  | -2.18 |
| A_11_P0000016691 |  | 2.05 | A_11_P170838 |  | -2.18 |
| A_11_P0000017202 |  | 2.05 | A_11_P193973 |  | -2.18 |
| A_11_P061966 | Opa interacting protein 5 | 2.05 | A_11_P0000019948 | S-antigen; retina and pineal gland (arrestin) | -2.18 |
| A_11_P0000022365 | TSC22 domain family, member 1 | 2.05 | A_11_P145998 |  | -2.18 |
| A_11_P098126 |  | 2.05 | A_11_P00000555 | serine/threonine kinase 32C | -2.18 |
| A_11_P0000031772 | SPC25, NDC80 kinetochore complex component | 2.05 | A_11_P0000040811 |  | -2.18 |
| A_11_P000005878 | phosphotyrosine interaction domain containing 1 | 2.05 | A_11_P174248 | RPTOR independent companion of MTOR, complex 2 | -2.18 |
| A_11_P111881 | ribosomal protein S5 | 2.05 | A_11_P204903 | IQ motif containing GTPase activating protein 2 | -2.18 |
| A_11_P083221 | surfeit 6 | 2.05 | A_11_P119016 | phospholipase A2, group XVI-like | -2.18 |
| A_11_P00000715 | G protein-coupled receptor 34 | 2.05 | A_11_P0000033616 | SMAD family member 7 | -2.18 |
| A_11_P0000034843 | UTP14, U3 small nucleolar ribonucleoprotein, homolog A (yeast) | 2.05 | A_11_P0000020245 | peroxisome proliferator-activated receptor gamma | -2.18 |
| A_11_P0000022937 | peripherin | 2.05 | A_11_P078761 | tripartite motif containing 58 | -2.18 |
| A_11_P223093 | reticulon 4 interacting protein 1 | 2.05 | A_11_P192073 | Bardet-Biedl syndrome 2 | -2.18 |
| A_11_P0000020899 | UTP3, small subunit (SSU) processome component, homolog (S. cerevisiae) | 2.05 | A_11_P179423 |  | -2.18 |
| A_11_P0000018946 |  | 2.05 | A_11_P171908 |  | -2.18 |
| A_11_P104056 | bicaudal C homolog 1 (Drosophila) | 2.05 | A_11_P0000012084 |  | -2.18 |
| A_11_P0000041525 |  | 2.05 | A_11_P0000016004 |  | -2.18 |
| A_11_P0000038930 |  | 2.05 | A_11_P0000033702 | kelch domain containing 1 | -2.18 |
| A_11_P185613 | gem (nuclear organelle) associated protein 6 | 2.05 | A_11_P0000011932 |  | -2.18 |
| A_11_P149568 |  | 2.05 | A_11_P0000033577 | mindbomb E3 ubiquitin protein ligase 1 | -2.18 |
| A_11_P000004336 |  | 2.05 | A_11_P218263 |  | -2.18 |
| A_11_P0000019901 | adrenomedullin | 2.05 | A_11_P00000167 | proline-rich nuclear receptor coactivator 1 | -2.18 |
| A_11_P088366 | adenosine A3 receptor | 2.05 | A_11_P069191 | cOR56B6 olfactory receptor family 56 subfamily B-like | -2.18 |
| A_11_P183563 |  | 2.05 | A_11_P113416 | uncharacterized LOC612543 | -2.17 |
| A_11_P074501 | protein phosphatase 1, regulatory (inhibitor) subunit 14B | 2.05 | A_11_P101916 | UPF2 regulator of nonsense transcripts homolog (yeast) | -2.17 |
| A_11_P000005199 |  | 2.05 | A_11_P061353 |  | -2.17 |
| A_11_P0000022123 | IMP (inosine 5'-monophosphate) dehydrogenase 2 | 2.04 | A_11_P215123 |  | -2.17 |
| A_11_P165933 | cytochrome P450, family 26, subfamily B, polypeptide 1 | 2.04 | A_11_P0000025392 | sperm associated antigen 9 | -2.17 |
| A_11_P0000021129 | hedgehog interacting protein | 2.04 | A_11_P0000012795 |  | -2.17 |
| A_11_P0000014338 |  | 2.04 | A_11_P092906 | mitogen-activated protein kinase kinase kinase kinase 5 | -2.17 |
| A_11_P0000035459 | solute carrier family 39 (zinc transporter), member 6 | 2.04 | A_11_P220978 | mitofusin 2 | -2.17 |
| A_11_P0000024165 | TBC1 domain family, member 19 | 2.04 | A_11_P0000018609 |  | -2.17 |
| A_11_P0000029668 | cytochrome c oxidase subunit IV isoform 2 (lung) | 2.04 | A_11_P183843 | family with sequence similarity 190, member B | -2.17 |
| A_11_P198758 |  | 2.04 | A_11_P0000015594 | protein phosphatase 1, regulatory subunit 27 | -2.17 |
| A_11_P0000041122 | zinc finger protein 323 | 2.04 | A_11_P0000028762 | ataxin 7 | -2.17 |
| A_11_P0000025204 |  | 2.04 | A_11_P069001 |  | -2.17 |
| A_11_P186938 | family with sequence similarity 129, member A | 2.04 | A_11_P155328 | family with sequence similarity 190, member B | -2.17 |
| A_11_P0000023935 | GULP, engulfment adaptor PTB domain containing 1 | 2.04 | A_11_P0000032895 | nuclear factor of activated T-cells 5, tonicity-responsive | -2.17 |
| A_11_P0000034639 |  | 2.04 | A_11_P0000015619 | lectin, galactoside-binding-like | -2.17 |
| A_11_P0000033682 | KIAA0391 ortholog | 2.04 | A_11_P0000025081 | enolase superfamily member 1 | -2.17 |
| A_11_P0000039088 |  | 2.04 | A_11_P0000034290 | cAMP responsive element binding protein 3-like 2-like | -2.17 |
| A_11_P167348 |  | 2.04 | A_11_P099926 | SET binding protein 1 | -2.17 |
| A_11_P116181 | signal sequence receptor, delta | 2.04 | A_11_P0000025521 | spectrin, alpha, non-erythrocytic 1 | -2.17 |
| A_11_P190853 |  | 2.04 | A_11_P127746 |  | -2.17 |
| A_11_P0000025501 | ribosomal protein L7a | 2.04 | A_11_P201853 |  | -2.17 |
| A_11_P050401 | serpin peptidase inhibitor, clade A (alpha-1 antiproteinase, antitrypsin), member 1 | 2.04 | A_11_P182178 |  | -2.17 |
| A_11_P0000024924 | importin 9 | 2.04 | A_11_P077571 | transmembrane protein 139 | -2.17 |
| A_11_P196078 | glycine C-acetyltransferase | 2.04 | A_11_P169203 |  | -2.17 |
| A_11_P0000020762 |  | 2.04 | A_11_P191708 | aminoacylase 1 | -2.17 |
| A_11_P0000020297 | Mdm1 nuclear protein homolog (mouse) | 2.04 | A_11_P0000039575 |  | -2.17 |
| A_11_P183883 | cerebral endothelial cell adhesion molecule | 2.04 | A_11_P0000028939 | immediate early response 2 | -2.17 |
| A_11_P166978 |  | 2.04 | A_11_P155103 | ring finger protein 128, E3 ubiquitin protein ligase | -2.17 |
| A_11_P134076 |  | 2.04 | A_11_P125656 |  | -2.17 |
| A_11_P0000021906 | HSPA (heat shock 70kDa) binding protein, cytoplasmic cochaperone 1 | 2.04 | A_11_P0000020767 | HECT domain and ankyrin repeat containing E3 ubiquitin protein ligase 1 | -2.17 |
| A_11_P0000038833 |  | 2.04 | A_11_P00000802 | zinc finger and BTB domain containing 4 | -2.17 |
| A_11_P0000039219 | sarcoglycan, alpha (50kDa dystrophin-associated glycoprotein) | 2.03 | A_11_P0000039221 |  | -2.17 |
| A_11_P105621 | fasciculation and elongation protein zeta 1 (zygin I) | 2.03 | A_11_P148608 | membrane protein, palmitoylated 6 (MAGUK p55 subfamily member 6) | -2.17 |
| A_11_P218558 | GULP, engulfment adaptor PTB domain containing 1 | 2.03 | A_11_P0000030906 | adenylate cyclase 7 | -2.16 |
| A_11_P0000040470 | myosin IC | 2.03 | A_11_P104231 | SAR1 homolog A (S. cerevisiae) | -2.16 |
| A_11_P0000015575 |  | 2.03 | A_11_P000004443 | crystallin, beta B2 | -2.16 |
| A_11_P197573 |  | 2.03 | A_11_P0000026434 | family with sequence similarity 83, member B | -2.16 |
| A_11_P0000038796 |  | 2.03 | A_11_P000001493 | phospholipid hydroperoxide glutathione peroxidase | -2.16 |
| A_11_P0000020824 | eukaryotic translation initiation factor 3, subunit H | 2.03 | A_11_P189013 | elongation factor, RNA polymerase II, 2 | -2.16 |
| A_11_P190433 |  | 2.03 | A_11_P0000040118 | chromosome 11 open reading frame, human C5orf45 | -2.16 |
| A_11_P054666 |  | 2.03 | A_11_P00000172 | HERPUD family member 2 | -2.16 |
| A_11_P205533 |  | 2.03 | A_11_P0000012822 |  | -2.16 |
| A_11_P209458 | syntaxin 2 | 2.03 | A_11_P0000030979 | progestin and adipoQ receptor family member VII | -2.16 |
| A_11_P105996 | FXYD domain containing ion transport regulator 6 | 2.03 | A_11_P0000037172 |  | -2.16 |
| A_11_P121916 | CDC42 effector protein (Rho GTPase binding) 3 | 2.03 | A_11_P0000016749 |  | -2.16 |
| A_11_P0000029683 | translocase of inner mitochondrial membrane 8 homolog A (yeast) | 2.03 | A_11_P116591 |  | -2.16 |
| A_11_P0000015023 |  | 2.03 | A_11_P151178 |  | -2.16 |
| A_11_P076266 | mitochondrial ribosomal protein L34 | 2.03 | A_11_P0000010969 |  | -2.16 |
| A_11_P055826 | CD48 molecule | 2.03 | A_11_P208068 |  | -2.16 |
| A_11_P0000031341 | starch binding domain 1 | 2.03 | A_11_P000008190 |  | -2.16 |
| A_11_P086646 | hippocalcin-like 1 | 2.03 | A_11_P000005176 |  | -2.16 |
| A_11_P0000024839 |  | 2.03 | A_11_P200928 |  | -2.16 |
| A_11_P056386 | membrane bound O-acyltransferase domain containing 1 | 2.03 | A_11_P000009606 |  | -2.16 |
| A_11_P0000040677 |  | 2.03 | A_11_P193833 | cryptochrome 2 (photolyase-like) | -2.16 |
| A_11_P082371 | zinc finger, HIT-type containing 3 | 2.03 | A_11_P0000027055 | growth arrest-specific 2 like 3 | -2.16 |
| A_11_P0000030008 | peroxisomal membrane protein 2, 22kDa | 2.03 | A_11_P0000017888 |  | -2.16 |
| A_11_P185703 |  | 2.03 | A_11_P0000021243 | glutathione reductase | -2.16 |
| A_11_P126161 | CDK5 regulatory subunit associated protein 1-like 1 | 2.03 | A_11_P0000031374 | glutamate receptor, ionotropic, delta 2 | -2.16 |
| A_11_P0000032193 | arylsulfatase family, member K | 2.03 | A_11_P176968 | endonuclease/exonuclease/phosphatase family domain containing 1 | -2.16 |
| A_11_P157393 |  | 2.03 | A_11_P133921 |  | -2.16 |
| A_11_P000003548 |  | 2.02 | A_11_P0000025967 | BTB (POZ) domain containing 11 | -2.16 |
| A_11_P0000016317 |  | 2.02 | A_11_P0000014514 |  | -2.15 |
| A_11_P0000024225 | G protein-coupled receptor 137B | 2.02 | A_11_P184788 |  | -2.15 |
| A_11_P0000032270 | catenin (cadherin-associated protein), alpha 3 | 2.02 | A_11_P139056 |  | -2.15 |
| A_11_P051221 | thiopurine S-methyltransferase | 2.02 | A_11_P0000020101 | chemokine (C-C motif) ligand 4 | -2.15 |
| A_11_P089076 | family with sequence similarity 118, member A | 2.02 | A_11_P137331 | pyruvate dehydrogenase kinase, isozyme 2 | -2.15 |
| A_11_P106771 | RAN guanine nucleotide release factor | 2.02 | A_11_P000008297 |  | -2.15 |
| A_11_P0000040520 | adenylate cyclase 2 (brain) | 2.02 | A_11_P0000027703 |  | -2.15 |
| A_11_P179608 |  | 2.02 | A_11_P079316 | alkylglycerol monooxygenase | -2.15 |
| A_11_P0000021977 | fibrillarin | 2.02 | A_11_P0000028155 | trace amine associated receptor 5 | -2.15 |
| A_11_P088976 | TraB domain containing | 2.02 | A_11_P000003805 |  | -2.15 |
| A_11_P0000024748 |  | 2.02 | A_11_P134226 |  | -2.15 |
| A_11_P0000024291 | annexin A8-like 1 | 2.02 | A_11_P00000784 | family with sequence similarity 190, member B | -2.15 |
| A_11_P056313 | TBC1 domain family, member 7 | 2.02 | A_11_P0000026447 | collagen, type XIX, alpha 1 | -2.15 |
| A_11_P0000020419 | phosphatidylinositol glycan anchor biosynthesis, class F | 2.02 | A_11_P104666 | cadherin-related family member 2 | -2.15 |
| A_11_P119426 | dermatopontin | 2.02 | A_11_P0000016125 |  | -2.15 |
| A_11_P177633 |  | 2.02 | A_11_P134751 |  | -2.15 |
| A_11_P056816 | RAN, member RAS oncogene family | 2.02 | A_11_P0000018242 |  | -2.15 |
| A_11_P000004843 |  | 2.02 | A_11_P184173 | glycerophosphodiester phosphodiesterase domain containing 2 | -2.15 |
| A_11_P0000033804 | basic leucine zipper transcription factor, ATF-like | 2.02 | A_11_P116436 | tetratricopeptide repeat domain 19 | -2.15 |
| A_11_P0000014531 | translocase of outer mitochondrial membrane 7 homolog (yeast) | 2.02 | A_11_P0000017228 |  | -2.15 |
| A_11_P200978 |  | 2.02 | A_11_P0000028616 | catsper channel auxiliary subunit gamma | -2.15 |
| A_11_P201968 | topoisomerase (DNA) II alpha 170kDa | 2.02 | A_11_P0000021963 | plasminogen activator, urokinase receptor | -2.15 |
| A_11_P0000022219 | eukaryotic translation elongation factor 2 | 2.02 | A_11_P00000249 |  | -2.15 |
| A_11_P0000022200 | KN motif and ankyrin repeat domains 3 | 2.02 | A_11_P0000022550 | attractin | -2.15 |
| A_11_P097646 | heparan sulfate (glucosamine) 3-O-sulfotransferase 6 | 2.02 | A_11_P152189 |  | -2.15 |
| A_11_P0000039188 |  | 2.02 | A_11_P000008126 |  | -2.15 |
| A_11_P067966 | cytochrome P450, family 24, subfamily A, polypeptide 1 | 2.02 | A_11_P000004217 |  | -2.15 |
| A_11_P0000039246 |  | 2.02 | A_11_P0000019437 |  | -2.15 |
| A_11_P0000013927 |  | 2.02 | A_11_P152728 | calcium binding and coiled-coil domain 2 | -2.15 |
| A_11_P0000027245 | neuregulin 1 | 2.02 | A_11_P0000020965 |  | -2.15 |
| A_11_P102556 | centromere protein K | 2.02 | A_11_P0000034065 | tripartite motif containing 25 | -2.15 |
| A_11_P093976 | kinesin light chain 1 | 2.01 | A_11_P134301 |  | -2.15 |
| A_11_P058886 | 3-hydroxybutyrate dehydrogenase, type 1 | 2.01 | A_11_P206163 |  | -2.15 |
| A_11_P056371 | NHL repeat containing 1 | 2.01 | A_11_P093211 | MYC associated factor X | -2.15 |
| A_11_P0000016404 | receptor accessory protein 2 | 2.01 | A_11_P067671 | fat storage-inducing transmembrane protein 2 | -2.14 |
| A_11_P0000028787 | stabilin 1 | 2.01 | A_11_P174913 |  | -2.14 |
| A_11_P125021 |  | 2.01 | A_11_P0000010153 |  | -2.14 |
| A_11_P0000019785 | flavin containing monooxygenase 1 | 2.01 | A_11_P0000033754 | MYC associated factor X | -2.14 |
| A_11_P0000013962 |  | 2.01 | A_11_P107421 |  | -2.14 |
| A_11_P0000033486 | SMG5 nonsense mediated mRNA decay factor | 2.01 | A_11_P000004859 |  | -2.14 |
| A_11_P0000030442 | prohibitin 2 | 2.01 | A_11_P000003821 |  | -2.14 |
| A_11_P177188 | ribosomal protein L15 | 2.01 | A_11_P000009296 |  | -2.14 |
| A_11_P0000028236 | family with sequence similarity 26, member F | 2.01 | A_11_P0000020726 | family with sequence similarity 135, member A | -2.14 |
| A_11_P000001740 |  | 2.01 | A_11_P174758 | growth differentiation factor 9 | -2.14 |
| A_11_P0000025297 |  | 2.01 | A_11_P0000017363 |  | -2.14 |
| A_11_P111506 | phosphatidylinositol glycan anchor biosynthesis, class N | 2.01 | A_11_P0000023128 | WD repeat domain 11 | -2.14 |
| A_11_P000002068 | beta-spectrin | 2.01 | A_11_P0000022827 | disintegrin and metalloproteinase domain-containing protein 1a-like | -2.14 |
| A_11_P115106 |  | 2.01 | A_11_P186943 | potassium channel, subfamily K, member 5 | -2.14 |
| A_11_P0000020715 | bone morphogenetic protein 5 | 2.01 | A_11_P171378 |  | -2.14 |
| A_11_P00000221 |  | 2.01 | A_11_P128361 | transmembrane protein 41B | -2.14 |
| A_11_P206278 | thioredoxin-like 4A | 2.01 | A_11_P086611 | ArfGAP with SH3 domain, ankyrin repeat and PH domain 2 | -2.14 |
| A_11_P0000018400 | splicing factor 3b, subunit 5, 10kDa | 2.01 | A_11_P0000029104 | midnolin | -2.14 |
| A_11_P102516 | leucine rich repeat containing 70 | 2.01 | A_11_P071616 | acyl-CoA dehydrogenase family, member 11 | -2.14 |
| A_11_P182038 | ribosomal protein S3 | 2.01 | A_11_P214688 |  | -2.14 |
| A_11_P0000028315 | receptor tyrosine kinase-like orphan receptor 2 | 2.01 | A_11_P00000175 | endonuclease/exonuclease/phosphatase family domain containing 1 | -2.14 |
| A_11_P0000018304 | exophilin 5 | 2.01 | A_11_P063921 |  | -2.14 |
| A_11_P164088 | zinc finger protein 496 | 2.01 | A_11_P169808 |  | -2.14 |
| A_11_P099256 | myelin protein zero-like 1 | 2.01 | A_11_P104226 | apoptosis-inducing factor, mitochondrion-associated, 2 | -2.14 |
| A_11_P0000032891 | zinc finger homeobox 3 | 2.01 | A_11_P000003414 |  | -2.14 |
| A_11_P149613 |  | 2.01 | A_11_P086936 | solute carrier family 30 (zinc transporter), member 3 | -2.14 |
| A_11_P082186 |  | 2.01 | A_11_P149428 | atlastin GTPase 2 | -2.14 |
| A_11_P0000040604 | DNA (cytosine-5-)-methyltransferase 1 | 2.01 | A_11_P188513 | serine/threonine kinase 38 like | -2.14 |
| A_11_P163288 | ZFP30 zinc finger protein | 2.01 | A_11_P0000029083 | transducin-like enhancer of split 6 (E(sp1) homolog, Drosophila) | -2.14 |
| A_11_P0000040550 |  | 2.01 | A_11_P124366 | growth factor receptor-bound protein 10 | -2.14 |
| A_11_P093201 | RAB15, member RAS onocogene family | 2.01 | A_11_P094546 | GRAM domain containing 3 | -2.14 |
| A_11_P0000019888 | prostaglandin E receptor 2 (subtype EP2), 53kDa | 2.01 | A_11_P000007361 |  | -2.14 |
| A_11_P0000020037 | Sec61 beta subunit | 2.01 | A_11_P159913 |  | -2.14 |
| A_11_P0000026173 | myeloid/lymphoid or mixed-lineage leukemia (trithorax homolog, Drosophila); translocated to, 3 | 2.01 | A_11_P161268 |  | -2.14 |
| A_11_P0000024830 | mitochondrial ribosomal protein L28 | 2.01 | A_11_P214643 | membrane-associated ring finger (C3HC4) 3, E3 ubiquitin protein ligase | -2.14 |
| A_11_P169563 | atlastin GTPase 3 | 2.01 | A_11_P198198 |  | -2.13 |
| A_11_P0000030111 | tyrosylprotein sulfotransferase 2 | 2.01 | A_11_P069146 |  | -2.13 |
| A_11_P117451 | v-yes-1 Yamaguchi sarcoma viral related oncogene homolog | 2.01 | A_11_P000007889 |  | -2.13 |
| A_11_P190603 |  | 2.01 | A_11_P0000026414 | meprin A, alpha (PABA peptide hydrolase) | -2.13 |
| A_11_P0000040224 |  | 2.00 | A_11_P000001791 | sodium channel, voltage-gated, type II, beta subunit | -2.13 |
| A_11_P0000023872 | RNA binding motif, single stranded interacting protein 1 | 2.00 | A_11_P053771 | advanced glycosylation end product-specific receptor | -2.13 |
| A_11_P150223 | translocase of inner mitochondrial membrane 44 homolog (yeast) | 2.00 | A_11_P0000022571 | BPI fold containing family B, member 1 | -2.13 |
| A_11_P174088 |  | 2.00 | A_11_P058691 | deltex 3-like (Drosophila) | -2.13 |
| A_11_P0000020080 | RAN, member RAS oncogene family | 2.00 | A_11_P213373 |  | -2.13 |
| A_11_P111656 | programmed cell death 1 ligand 2 | 2.00 | A_11_P0000015917 |  | -2.13 |
| A_11_P150853 |  | 2.00 | A_11_P0000039454 |  | -2.13 |
| A_11_P171628 | collagen, type V, alpha 2 | 2.00 | A_11_P0000029147 |  | -2.13 |
| A_11_P0000024775 | myosin, heavy chain 11, smooth muscle | 2.00 | A_11_P067361 |  | -2.13 |
| A_11_P0000020262 | ribosomal protein S26 | 2.00 | A_11_P000007743 |  | -2.13 |
| A_11_P116286 | C-x(9)-C motif containing 4 homolog (S. cerevisiae) | 2.00 | A_11_P0000023684 | SEC24 family, member D (S. cerevisiae) | -2.13 |
| A_11_P098246 | tubulin tyrosine ligase-like family, member 7 | 2.00 | A_11_P0000021359 | atlastin GTPase 2 | -2.13 |
|  |  |  | A_11_P0000038499 |  | -2.13 |
|  |  |  | A_11_P124681 | strawberry notch homolog 1 (Drosophila) | -2.13 |
|  |  |  | A_11_P0000027671 | glucagon receptor | -2.13 |
|  |  |  | A_11_P000009440 |  | -2.13 |
|  |  |  | A_11_P0000040313 |  | -2.13 |
|  |  |  | A_11_P115241 | synaptotagmin-like 4 | -2.13 |
|  |  |  | A_11_P0000023585 | synaptojanin 1 | -2.13 |
|  |  |  | A_11_P072201 | KIAA1109 ortholog | -2.13 |
|  |  |  | A_11_P202993 |  | -2.13 |
|  |  |  | A_11_P0000040355 |  | -2.13 |
|  |  |  | A_11_P0000015122 |  | -2.13 |
|  |  |  | A_11_P0000039543 | ubiquitin-conjugating enzyme E2R 2 | -2.13 |
|  |  |  | A_11_P095016 | perilipin 2 | -2.13 |
|  |  |  | A_11_P0000040394 |  | -2.13 |
|  |  |  | A_11_P0000016349 | solute carrier family 13 (sodium-dependent dicarboxylate transporter), member 2 | -2.13 |
|  |  |  | A_11_P0000040204 |  | -2.13 |
|  |  |  | A_11_P109911 | phosphatidylinositol 4-kinase type 2 beta | -2.13 |
|  |  |  | A_11_P000004519 |  | -2.12 |
|  |  |  | A_11_P141713 |  | -2.12 |
|  |  |  | A_11_P212878 | E74-like factor 2 (ets domain transcription factor) | -2.12 |
|  |  |  | A_11_P0000023026 | adiponectin receptor 2 | -2.12 |
|  |  |  | A_11_P0000016550 | cryptochrome 2 (photolyase-like) | -2.12 |
|  |  |  | A_11_P0000026571 | metastasis suppressor 1 | -2.12 |
|  |  |  | A_11_P0000010895 |  | -2.12 |
|  |  |  | A_11_P193983 | SUN domain containing ossification factor | -2.12 |
|  |  |  | A_11_P180083 |  | -2.12 |
|  |  |  | A_11_P0000017932 |  | -2.12 |
|  |  |  | A_11_P0000040056 |  | -2.12 |
|  |  |  | A_11_P0000019409 |  | -2.12 |
|  |  |  | A_11_P189518 |  | -2.12 |
|  |  |  | A_11_P0000018254 |  | -2.12 |
|  |  |  | A_11_P000006847 |  | -2.12 |
|  |  |  | A_11_P120021 |  | -2.12 |
|  |  |  | A_11_P176528 | serine/threonine kinase 38 like | -2.12 |
|  |  |  | A_11_P175758 | jumonji, AT rich interactive domain 2 | -2.12 |
|  |  |  | A_11_P0000029105 | serine/threonine kinase 11 | -2.12 |
|  |  |  | A_11_P209385 | glycerol kinase | -2.12 |
|  |  |  | A_11_P180878 | autophagy related 2A | -2.12 |
|  |  |  | A_11_P0000032923 | KIAA0895-like ortholog | -2.12 |
|  |  |  | A_11_P0000034753 | premature ovarian failure, 1B | -2.12 |
|  |  |  | A_11_P0000019802 | UDP glucuronosyltransferase 1 family, polypeptide A6 | -2.12 |
|  |  |  | A_11_P0000030890 | chemokine (C-C motif) ligand 22 | -2.12 |
|  |  |  | A_11_P179738 |  | -2.11 |
|  |  |  | A_11_P215788 | neighbor of BRCA1 gene 1 | -2.11 |
|  |  |  | A_11_P0000026261 | Kruppel-like factor 4 (gut) | -2.11 |
|  |  |  | A_11_P184583 | ArfGAP with FG repeats 1 | -2.11 |
|  |  |  | A_11_P197383 |  | -2.11 |
|  |  |  | A_11_P0000012642 |  | -2.11 |
|  |  |  | A_11_P0000027086 | platelet derived growth factor C | -2.11 |
|  |  |  | A_11_P0000040612 |  | -2.11 |
|  |  |  | A_11_P0000030140 | ring finger protein 185 | -2.11 |
|  |  |  | A_11_P136636 |  | -2.11 |
|  |  |  | A_11_P177533 |  | -2.11 |
|  |  |  | A_11_P0000028207 | transmembrane protein 181 | -2.11 |
|  |  |  | A_11_P000007577 |  | -2.11 |
|  |  |  | A_11_P0000012201 |  | -2.11 |
|  |  |  | A_11_P088791 |  | -2.11 |
|  |  |  | A_11_P0000031859 | crystallin, gamma D | -2.11 |
|  |  |  | A_11_P0000019761 | solute carrier family 15 (oligopeptide transporter), member 1 | -2.11 |
|  |  |  | A_11_P0000019380 |  | -2.11 |
|  |  |  | A_11_P082836 | solute carrier family 43 (amino acid system L transporter), member 2 | -2.11 |
|  |  |  | A_11_P130871 | MAX dimerization protein 1 | -2.11 |
|  |  |  | A_11_P0000029640 |  | -2.11 |
|  |  |  | A_11_P0000016112 |  | -2.11 |
|  |  |  | A_11_P0000017073 |  | -2.11 |
|  |  |  | A_11_P0000018128 |  | -2.11 |
|  |  |  | A_11_P0000031620 |  | -2.11 |
|  |  |  | A_11_P177243 |  | -2.11 |
|  |  |  | A_11_P137711 | nuclear fragile X mental retardation protein interacting protein 2 | -2.11 |
|  |  |  | A_11_P0000041685 |  | -2.11 |
|  |  |  | A_11_P0000028208 | synaptotagmin-like 3 | -2.11 |
|  |  |  | A_11_P148458 | PDZ and LIM domain 5 | -2.11 |
|  |  |  | A_11_P0000030185 | armadillo repeat gene deleted in velocardiofacial syndrome | -2.11 |
|  |  |  | A_11_P0000031754 |  | -2.11 |
|  |  |  | A_11_P0000024886 | leucine rich repeat containing 8 family, member B | -2.11 |
|  |  |  | A_11_P0000013394 |  | -2.11 |
|  |  |  | A_11_P0000015183 |  | -2.11 |
|  |  |  | A_11_P0000020713 | ELOVL fatty acid elongase 5 | -2.11 |
|  |  |  | A_11_P218563 | T cell receptor associated transmembrane adaptor 1 | -2.11 |
|  |  |  | A_11_P0000032677 | lysine (K)-specific demethylase 6B | -2.11 |
|  |  |  | A_11_P111046 |  | -2.11 |
|  |  |  | A_11_P110011 | complexin 1 | -2.11 |
|  |  |  | A_11_P000004395 |  | -2.11 |
|  |  |  | A_11_P0000027836 | cOR4C3 olfactory receptor family 4 subfamily X-like | -2.11 |
|  |  |  | A_11_P065536 | UDP-N-acetyl-alpha-D-galactosamine:polypeptide N-acetylgalactosaminyltransferase 6 (GalNAc-T6) | -2.11 |
|  |  |  | A_11_P000009345 |  | -2.11 |
|  |  |  | A_11_P0000029636 | mitochondrial antiviral signaling protein | -2.11 |
|  |  |  | A_11_P069246 | dynein heavy chain domain 1 | -2.10 |
|  |  |  | A_11_P215738 | zinc finger protein 598 | -2.10 |
|  |  |  | A_11_P180843 |  | -2.10 |
|  |  |  | A_11_P0000020224 | MHC class I DLA-64 | -2.10 |
|  |  |  | A_11_P0000016517 |  | -2.10 |
|  |  |  | A_11_P000002452 |  | -2.10 |
|  |  |  | A_11_P101550 |  | -2.10 |
|  |  |  | A_11_P0000031102 | mitogen-activated protein kinase binding protein 1 | -2.10 |
|  |  |  | A_11_P0000032108 | SH3-domain binding protein 2 | -2.10 |
|  |  |  | A_11_P0000015369 | N-myristoyltransferase 2 | -2.10 |
|  |  |  | A_11_P066856 | bone morphogenetic protein 2 | -2.10 |
|  |  |  | A_11_P0000032815 | transmembrane protein 201 | -2.10 |
|  |  |  | A_11_P0000017248 | cholinergic receptor, nicotinic, beta 2 (neuronal) | -2.10 |
|  |  |  | A_11_P000007774 |  | -2.10 |
|  |  |  | A_11_P0000019829 | G protein-coupled receptor 83 | -2.10 |
|  |  |  | A_11_P0000027056 | solute carrier family 5 (sodium/monocarboxylate cotransporter), member 8 | -2.10 |
|  |  |  | A_11_P0000026621 | diacylglycerol O-acyltransferase 1 | -2.10 |
|  |  |  | A_11_P0000041406 |  | -2.10 |
|  |  |  | A_11_P00000358 | phosphatidic acid phosphatase type 2A | -2.10 |
|  |  |  | A_11_P0000012118 |  | -2.10 |
|  |  |  | A_11_P0000032894 |  | -2.10 |
|  |  |  | A_11_P0000024210 | IQ motif containing GTPase activating protein 2 | -2.10 |
|  |  |  | A_11_P0000025335 | ADAM metallopeptidase domain 11 | -2.10 |
|  |  |  | A_11_P0000019843 | dual oxidase 1 | -2.10 |
|  |  |  | A_11_P081791 | titin-cap | -2.10 |
|  |  |  | A_11_P000002246 |  | -2.10 |
|  |  |  | A_11_P190513 | O-linked N-acetylglucosamine (GlcNAc) transferase | -2.10 |
|  |  |  | A_11_P0000025386 |  | -2.10 |
|  |  |  | A_11_P159443 | F-box protein 33 | -2.10 |
|  |  |  | A_11_P000005673 |  | -2.10 |
|  |  |  | A_11_P104236 |  | -2.09 |
|  |  |  | A_11_P082156 | diacylglycerol kinase, epsilon 64kDa | -2.09 |
|  |  |  | A_11_P199428 |  | -2.09 |
|  |  |  | A_11_P191058 |  | -2.09 |
|  |  |  | A_11_P0000039585 | natural killer-tumor recognition sequence | -2.09 |
|  |  |  | A_11_P166363 |  | -2.09 |
|  |  |  | A_11_P217133 |  | -2.09 |
|  |  |  | A_11_P101152 | interferon, alpha 7 | -2.09 |
|  |  |  | A_11_P059256 | tigger transposable element derived 2 | -2.09 |
|  |  |  | A_11_P00000493 | patatin-like phospholipase domain containing 2 | -2.09 |
|  |  |  | A_11_P093051 |  | -2.09 |
|  |  |  | A_11_P0000026922 | rhomboid, veinlet-like 2 (Drosophila) | -2.09 |
|  |  |  | A_11_P069456 | SET binding factor 2 | -2.09 |
|  |  |  | A_11_P000007787 |  | -2.09 |
|  |  |  | A_11_P0000035253 |  | -2.09 |
|  |  |  | A_11_P0000016122 |  | -2.09 |
|  |  |  | A_11_P112156 | synaptotagmin V | -2.09 |
|  |  |  | A_11_P061336 |  | -2.09 |
|  |  |  | A_11_P175763 |  | -2.09 |
|  |  |  | A_11_P00000692 |  | -2.09 |
|  |  |  | A_11_P130616 |  | -2.09 |
|  |  |  | A_11_P0000011907 |  | -2.09 |
|  |  |  | A_11_P204913 | LYR motif containing 1 | -2.09 |
|  |  |  | A_11_P000009681 |  | -2.09 |
|  |  |  | A_11_P0000039402 |  | -2.09 |
|  |  |  | A_11_P173153 |  | -2.09 |
|  |  |  | A_11_P203848 | SH3-domain binding protein 2 | -2.09 |
|  |  |  | A_11_P0000028906 | cytochrome P450, family 4, subfamily F, polypeptide 22 | -2.09 |
|  |  |  | A_11_P072471 | cyclin T2 | -2.09 |
|  |  |  | A_11_P169508 | G protein-coupled receptor 116 | -2.09 |
|  |  |  | A_11_P214983 |  | -2.09 |
|  |  |  | A_11_P102316 | Rho GTPase activating protein 26 | -2.09 |
|  |  |  | A_11_P000002555 |  | -2.09 |
|  |  |  | A_11_P210188 |  | -2.09 |
|  |  |  | A_11_P0000019123 |  | -2.09 |
|  |  |  | A_11_P189233 |  | -2.09 |
|  |  |  | A_11_P000002199 | runt-related transcription factor 2 | -2.08 |
|  |  |  | A_11_P098271 | interferon-induced protein 44-like | -2.08 |
|  |  |  | A_11_P0000018564 |  | -2.08 |
|  |  |  | A_11_P194393 | acyl-CoA synthetase long-chain family member 5 | -2.08 |
|  |  |  | A_11_P105366 | myotubularin related protein 12 | -2.08 |
|  |  |  | A_11_P000007624 |  | -2.08 |
|  |  |  | A_11_P066941 |  | -2.08 |
|  |  |  | A_11_P103216 | nuclear receptor subfamily 0, group B, member 2 | -2.08 |
|  |  |  | A_11_P102401 | serine peptidase inhibitor, Kazal type 5 | -2.08 |
|  |  |  | A_11_P0000029457 | 1-acylglycerol-3-phosphate O-acyltransferase ABHD5-like | -2.08 |
|  |  |  | A_11_P0000013807 |  | -2.08 |
|  |  |  | A_11_P127231 |  | -2.08 |
|  |  |  | A_11_P000008636 |  | -2.08 |
|  |  |  | A_11_P0000014214 |  | -2.08 |
|  |  |  | A_11_P187183 |  | -2.08 |
|  |  |  | A_11_P0000013005 |  | -2.08 |
|  |  |  | A_11_P210458 |  | -2.08 |
|  |  |  | A_11_P0000023732 | karyopherin alpha 1 (importin alpha 5) | -2.08 |
|  |  |  | A_11_P000009273 |  | -2.08 |
|  |  |  | A_11_P0000019672 | adenosine A2b receptor | -2.08 |
|  |  |  | A_11_P167163 |  | -2.08 |
|  |  |  | A_11_P00000712 | phosphorylase, glycogen, muscle | -2.08 |
|  |  |  | A_11_P0000024725 | quinolinate phosphoribosyltransferase | -2.08 |
|  |  |  | A_11_P000008853 |  | -2.08 |
|  |  |  | A_11_P0000019830 | solute carrier family 3 (cystine, dibasic and neutral amino acid transporters, activator of cystine, dibasic and neutral amino acid transport), member 1 | -2.08 |
|  |  |  | A_11_P0000016993 | inositol 1,4,5-trisphosphate receptor, type 3 | -2.08 |
|  |  |  | A_11_P0000035199 |  | -2.08 |
|  |  |  | A_11_P108856 | IQ motif containing GTPase activating protein 2 | -2.08 |
|  |  |  | A_11_P0000023038 |  | -2.08 |
|  |  |  | A_11_P0000023263 | solute carrier family 23 (nucleobase transporters), member 1 | -2.08 |
|  |  |  | A_11_P0000022970 | serine/threonine kinase 38 like | -2.08 |
|  |  |  | A_11_P0000036088 |  | -2.08 |
|  |  |  | A_11_P0000020709 |  | -2.08 |
|  |  |  | A_11_P0000020132 | potassium voltage-gated channel, shaker-related subfamily, member 5 | -2.08 |
|  |  |  | A_11_P0000023925 | sperm specific antigen 2 | -2.08 |
|  |  |  | A_11_P060466 | ring finger protein 185 | -2.08 |
|  |  |  | A_11_P122566 | poly (ADP-ribose) polymerase family, member 12 | -2.08 |
|  |  |  | A_11_P0000039420 | LIM domain 7 | -2.08 |
|  |  |  | A_11_P079616 |  | -2.08 |
|  |  |  | A_11_P0000020569 |  | -2.08 |
|  |  |  | A_11_P189433 | serpin peptidase inhibitor, clade A (alpha-1 antiproteinase, antitrypsin), member 5 | -2.08 |
|  |  |  | A_11_P0000040314 |  | -2.07 |
|  |  |  | A_11_P062166 | sorbitol dehydrogenase | -2.07 |
|  |  |  | A_11_P0000030862 | phosphoinositide-3-kinase, regulatory subunit 1 (alpha) | -2.07 |
|  |  |  | A_11_P202983 |  | -2.07 |
|  |  |  | A_11_P0000013229 |  | -2.07 |
|  |  |  | A_11_P194443 | protein-L-isoaspartate (D-aspartate) O-methyltransferase domain containing 2 | -2.07 |
|  |  |  | A_11_P000009124 |  | -2.07 |
|  |  |  | A_11_P100401 |  | -2.07 |
|  |  |  | A_11_P0000024621 | pyruvate dehydrogenase phosphatase regulatory subunit | -2.07 |
|  |  |  | A_11_P0000036539 |  | -2.07 |
|  |  |  | A_11_P0000038742 |  | -2.07 |
|  |  |  | A_11_P0000012080 |  | -2.07 |
|  |  |  | A_11_P000005108 | peroxisomal biogenesis factor 26 | -2.07 |
|  |  |  | A_11_P0000038229 |  | -2.07 |
|  |  |  | A_11_P172273 |  | -2.07 |
|  |  |  | A_11_P075591 | aminolevulinate, delta-, synthase 1 | -2.07 |
|  |  |  | A_11_P0000019971 | vasodilator-stimulated phosphoprotein | -2.07 |
|  |  |  | A_11_P058836 | transmembrane 4 L six family member 19 | -2.07 |
|  |  |  | A_11_P0000024913 |  | -2.07 |
|  |  |  | A_11_P0000032471 | cadherin 9, type 2 (T1-cadherin) | -2.07 |
|  |  |  | A_11_P172833 |  | -2.07 |
|  |  |  | A_11_P110471 | microtubule-associated protein 7 | -2.07 |
|  |  |  | A_11_P000004175 |  | -2.07 |
|  |  |  | A_11_P000008611 |  | -2.07 |
|  |  |  | A_11_P0000030284 | keratin 84 | -2.07 |
|  |  |  | A_11_P175813 | transmembrane protein 63A | -2.07 |
|  |  |  | A_11_P108611 | calpastatin | -2.07 |
|  |  |  | A_11_P064561 | dpy-19-like 4 (C. elegans) | -2.07 |
|  |  |  | A_11_P000005053 |  | -2.07 |
|  |  |  | A_11_P098831 | syntaxin 6 | -2.07 |
|  |  |  | A_11_P0000023731 |  | -2.07 |
|  |  |  | A_11_P147883 | olfactory receptor, family 2, subfamily A, member 14 | -2.07 |
|  |  |  | A_11_P0000027226 | solute carrier family 20 (phosphate transporter), member 2 | -2.07 |
|  |  |  | A_11_P0000016694 | UBX domain protein 8 | -2.07 |
|  |  |  | A_11_P112766 | izumo sperm-egg fusion 1 | -2.06 |
|  |  |  | A_11_P217508 |  | -2.06 |
|  |  |  | A_11_P0000021818 |  | -2.06 |
|  |  |  | A_11_P192048 | acyl-CoA dehydrogenase family, member 11 | -2.06 |
|  |  |  | A_11_P178373 |  | -2.06 |
|  |  |  | A_11_P184518 |  | -2.06 |
|  |  |  | A_11_P000004178 |  | -2.06 |
|  |  |  | A_11_P0000018007 |  | -2.06 |
|  |  |  | A_11_P062086 | stereocilin | -2.06 |
|  |  |  | A_11_P163908 | phosphatidic acid phosphatase type 2A | -2.06 |
|  |  |  | A_11_P0000038561 |  | -2.06 |
|  |  |  | A_11_P0000018721 |  | -2.06 |
|  |  |  | A_11_P111111 |  | -2.06 |
|  |  |  | A_11_P100231 | enolase superfamily member 1 | -2.06 |
|  |  |  | A_11_P0000016458 |  | -2.06 |
|  |  |  | A_11_P0000017659 |  | -2.06 |
|  |  |  | A_11_P0000010719 |  | -2.06 |
|  |  |  | A_11_P096881 | retinoblastoma binding protein 6 | -2.06 |
|  |  |  | A_11_P092766 | nuclear factor of kappa light polypeptide gene enhancer in B-cells inhibitor, alpha | -2.06 |
|  |  |  | A_11_P087056 | lysocardiolipin acyltransferase 1 | -2.06 |
|  |  |  | A_11_P0000016748 |  | -2.06 |
|  |  |  | A_11_P0000031977 |  | -2.06 |
|  |  |  | A_11_P062271 | adaptor-related protein complex 4, epsilon 1 subunit | -2.06 |
|  |  |  | A_11_P197977 |  | -2.06 |
|  |  |  | A_11_P0000021443 | PDZ domain containing 1 | -2.06 |
|  |  |  | A_11_P156558 |  | -2.06 |
|  |  |  | A_11_P0000040015 |  | -2.06 |
|  |  |  | A_11_P0000031758 | interferon induced with helicase C domain 1 | -2.06 |
|  |  |  | A_11_P00000102 | transmembrane protein 263 | -2.06 |
|  |  |  | A_11_P0000021264 | FAT atypical cadherin 1 | -2.06 |
|  |  |  | A_11_P0000017367 |  | -2.06 |
|  |  |  | A_11_P0000041778 |  | -2.06 |
|  |  |  | A_11_P0000023132 | pleckstrin homology domain containing, family A (phosphoinositide binding specific) member 1 | -2.05 |
|  |  |  | A_11_P104496 | family with sequence similarity 190, member B | -2.05 |
|  |  |  | A_11_P0000013352 |  | -2.05 |
|  |  |  | A_11_P0000019803 | transglutaminase 1 (K polypeptide epidermal type I, protein-glutamine-gamma-glutamyltransferase) | -2.05 |
|  |  |  | A_11_P0000027445 | F-box protein 41 | -2.05 |
|  |  |  | A_11_P178798 |  | -2.05 |
|  |  |  | A_11_P000008697 |  | -2.05 |
|  |  |  | A_11_P135321 |  | -2.05 |
|  |  |  | A_11_P0000022504 | TSC22 domain family, member 2 | -2.05 |
|  |  |  | A_11_P0000021775 | interferon gamma receptor 1 | -2.05 |
|  |  |  | A_11_P0000040731 | perilipin 2 | -2.05 |
|  |  |  | A_11_P133471 |  | -2.05 |
|  |  |  | A_11_P000004406 |  | -2.05 |
|  |  |  | A_11_P0000037485 |  | -2.05 |
|  |  |  | A_11_P093641 | echinoderm microtubule associated protein like 5 | -2.05 |
|  |  |  | A_11_P0000027909 | two pore segment channel 2 | -2.05 |
|  |  |  | A_11_P0000024668 | spindle and kinetochore associated complex subunit 2 | -2.05 |
|  |  |  | A_11_P200658 |  | -2.05 |
|  |  |  | A_11_P075746 | ubiquitin-like modifier activating enzyme 7 | -2.05 |
|  |  |  | A_11_P174638 | atlastin GTPase 2 | -2.05 |
|  |  |  | A_11_P164298 |  | -2.05 |
|  |  |  | A_11_P185898 |  | -2.05 |
|  |  |  | A_11_P000008211 |  | -2.05 |
|  |  |  | A_11_P0000010715 |  | -2.05 |
|  |  |  | A_11_P126156 |  | -2.05 |
|  |  |  | A_11_P0000010167 |  | -2.05 |
|  |  |  | A_11_P0000010531 |  | -2.05 |
|  |  |  | A_11_P0000016020 |  | -2.05 |
|  |  |  | A_11_P178498 |  | -2.05 |
|  |  |  | A_11_P099046 | RAB GTPase activating protein 1-like | -2.05 |
|  |  |  | A_11_P0000033000 | zinc finger and SCAN domain containing 25 | -2.05 |
|  |  |  | A_11_P119811 |  | -2.05 |
|  |  |  | A_11_P0000013000 |  | -2.05 |
|  |  |  | A_11_P198443 |  | -2.05 |
|  |  |  | A_11_P000008681 |  | -2.05 |
|  |  |  | A_11_P0000032585 | CD3g molecule, gamma (CD3-TCR complex) | -2.05 |
|  |  |  | A_11_P000005964 |  | -2.05 |
|  |  |  | A_11_P107731 | solute carrier family 2 (facilitated glucose/fructose transporter), member 5 | -2.05 |
|  |  |  | A_11_P190258 |  | -2.05 |
|  |  |  | A_11_P0000026184 | interferon, kappa | -2.05 |
|  |  |  | A_11_P0000038731 |  | -2.05 |
|  |  |  | A_11_P0000014791 | heterochromatin protein 1, binding protein 3 | -2.05 |
|  |  |  | A_11_P0000034043 | pyruvate dehydrogenase kinase, isozyme 2 | -2.04 |
|  |  |  | A_11_P000006614 |  | -2.04 |
|  |  |  | A_11_P165318 |  | -2.04 |
|  |  |  | A_11_P0000012011 |  | -2.04 |
|  |  |  | A_11_P0000013635 |  | -2.04 |
|  |  |  | A_11_P0000041794 |  | -2.04 |
|  |  |  | A_11_P0000021761 | malic enzyme 2, NAD(+)-dependent, mitochondrial | -2.04 |
|  |  |  | A_11_P0000013387 |  | -2.04 |
|  |  |  | A_11_P108266 | nuclear factor of activated T-cells 5, tonicity-responsive | -2.04 |
|  |  |  | A_11_P163883 |  | -2.04 |
|  |  |  | A_11_P161613 | prostaglandin F2 receptor inhibitor | -2.04 |
|  |  |  | A_11_P217543 |  | -2.04 |
|  |  |  | A_11_P196783 |  | -2.04 |
|  |  |  | A_11_P0000016563 |  | -2.04 |
|  |  |  | A_11_P102496 | kinesin heavy chain member 2A | -2.04 |
|  |  |  | A_11_P000007325 |  | -2.04 |
|  |  |  | A_11_P096006 | uroplakin 3B | -2.04 |
|  |  |  | A_11_P058676 | family with sequence similarity 162, member A | -2.04 |
|  |  |  | A_11_P0000021452 | pleckstrin homology domain containing, family O member 1 | -2.04 |
|  |  |  | A_11_P0000039409 |  | -2.04 |
|  |  |  | A_11_P0000022073 |  | -2.04 |
|  |  |  | A_11_P0000015743 |  | -2.04 |
|  |  |  | A_11_P182333 | target of myb1 (chicken) | -2.04 |
|  |  |  | A_11_P0000016677 |  | -2.04 |
|  |  |  | A_11_P0000018451 | heparin-binding EGF-like growth factor | -2.04 |
|  |  |  | A_11_P0000033426 | methyltransferase like 11B | -2.04 |
|  |  |  | A_11_P094926 | lysine (K)-specific demethylase 4C | -2.04 |
|  |  |  | A_11_P0000010635 |  | -2.04 |
|  |  |  | A_11_P0000037047 |  | -2.04 |
|  |  |  | A_11_P0000041689 |  | -2.04 |
|  |  |  | A_11_P000006204 |  | -2.04 |
|  |  |  | A_11_P0000041487 | laminin, alpha 2 | -2.04 |
|  |  |  | A_11_P0000035621 | SID1 transmembrane family, member 2 | -2.04 |
|  |  |  | A_11_P0000017978 |  | -2.04 |
|  |  |  | A_11_P0000040748 |  | -2.04 |
|  |  |  | A_11_P085421 | PDZK1 interacting protein 1 | -2.04 |
|  |  |  | A_11_P0000033110 | sodium channel, non-voltage-gated 1, gamma subunit | -2.04 |
|  |  |  | A_11_P167148 |  | -2.04 |
|  |  |  | A_11_P0000040164 |  | -2.03 |
|  |  |  | A_11_P207258 | shisa family member 5 | -2.03 |
|  |  |  | A_11_P137701 | SAR1 homolog A (S. cerevisiae) | -2.03 |
|  |  |  | A_11_P052786 | protein tyrosine phosphatase, receptor type, D | -2.03 |
|  |  |  | A_11_P156628 |  | -2.03 |
|  |  |  | A_11_P190568 | TEA domain family member 4 | -2.03 |
|  |  |  | A_11_P0000010146 |  | -2.03 |
|  |  |  | A_11_P0000014397 |  | -2.03 |
|  |  |  | A_11_P114171 | cyclin-dependent kinase-like 5 | -2.03 |
|  |  |  | A_11_P050841 | ATP-binding cassette, sub-family B (MDR/TAP), member 1 | -2.03 |
|  |  |  | A_11_P108981 | myotubularin related protein 10 | -2.03 |
|  |  |  | A_11_P0000015980 |  | -2.03 |
|  |  |  | A_11_P174223 |  | -2.03 |
|  |  |  | A_11_P000007467 |  | -2.03 |
|  |  |  | A_11_P194543 |  | -2.03 |
|  |  |  | A_11_P0000010259 |  | -2.03 |
|  |  |  | A_11_P0000014698 | fatty acid desaturase 6 | -2.03 |
|  |  |  | A_11_P0000026005 | ankyrin repeat domain 23 | -2.03 |
|  |  |  | A_11_P0000040041 |  | -2.03 |
|  |  |  | A_11_P0000011318 |  | -2.03 |
|  |  |  | A_11_P159723 | RAB5C, member RAS oncogene family | -2.03 |
|  |  |  | A_11_P058641 |  | -2.03 |
|  |  |  | A_11_P0000039687 |  | -2.03 |
|  |  |  | A_11_P0000015483 | ADP-ribosylation factor GTPase activating protein 3 | -2.03 |
|  |  |  | A_11_P0000023131 | transforming, acidic coiled-coil containing protein 2 | -2.03 |
|  |  |  | A_11_P0000027739 |  | -2.03 |
|  |  |  | A_11_P140471 |  | -2.03 |
|  |  |  | A_11_P0000028705 | IQ motif and Sec7 domain 1 | -2.03 |
|  |  |  | A_11_P0000021776 | PERP, TP53 apoptosis effector | -2.03 |
|  |  |  | A_11_P0000018266 |  | -2.03 |
|  |  |  | A_11_P192093 | inositol hexakisphosphate kinase 1 | -2.03 |
|  |  |  | A_11_P169788 | protein phosphatase 4, regulatory subunit 1 | -2.03 |
|  |  |  | A_11_P088236 | leucine rich repeat and Ig domain containing 4 | -2.03 |
|  |  |  | A_11_P00000475 |  | -2.03 |
|  |  |  | A_11_P064796 | coiled-coil domain containing 39 | -2.03 |
|  |  |  | A_11_P000005312 |  | -2.03 |
|  |  |  | A_11_P202623 |  | -2.03 |
|  |  |  | A_11_P0000012155 |  | -2.03 |
|  |  |  | A_11_P187073 |  | -2.03 |
|  |  |  | A_11_P051506 | coxsackie virus and adenovirus receptor | -2.03 |
|  |  |  | A_11_P0000023298 | kinesin heavy chain member 2A | -2.03 |
|  |  |  | A_11_P174538 |  | -2.03 |
|  |  |  | A_11_P139801 |  | -2.03 |
|  |  |  | A_11_P052381 | myosin, light chain 4, alkali; atrial, embryonic | -2.02 |
|  |  |  | A_11_P195718 |  | -2.02 |
|  |  |  | A_11_P0000011874 |  | -2.02 |
|  |  |  | A_11_P0000026138 | SEC24 family, member A (S. cerevisiae) | -2.02 |
|  |  |  | A_11_P0000018814 | vascular endothelial growth factor A | -2.02 |
|  |  |  | A_11_P172538 | BTB (POZ) domain containing 11 | -2.02 |
|  |  |  | A_11_P134261 |  | -2.02 |
|  |  |  | A_11_P0000027146 | transient receptor potential cation channel, subfamily V, member 5 | -2.02 |
|  |  |  | A_11_P000009453 |  | -2.02 |
|  |  |  | A_11_P0000019859 | solute carrier family 1 (neuronal/epithelial high affinity glutamate transporter, system Xag), member 1 | -2.02 |
|  |  |  | A_11_P0000013810 |  | -2.02 |
|  |  |  | A_11_P214828 |  | -2.02 |
|  |  |  | A_11_P0000014830 |  | -2.02 |
|  |  |  | A_11_P089081 | uroplakin 3A | -2.02 |
|  |  |  | A_11_P110221 | zinc finger, CCHC domain containing 2 | -2.02 |
|  |  |  | A_11_P0000012355 |  | -2.02 |
|  |  |  | A_11_P153588 | phosphatidylinositol transfer protein, alpha | -2.02 |
|  |  |  | A_11_P0000030925 | histone deacetylase 1 | -2.02 |
|  |  |  | A_11_P211448 |  | -2.02 |
|  |  |  | A_11_P0000023350 | NEDD4 binding protein 1 | -2.02 |
|  |  |  | A_11_P161143 |  | -2.02 |
|  |  |  | A_11_P149088 |  | -2.02 |
|  |  |  | A_11_P0000039744 |  | -2.02 |
|  |  |  | A_11_P122441 |  | -2.02 |
|  |  |  | A_11_P058413 |  | -2.02 |
|  |  |  | A_11_P0000016414 |  | -2.02 |
|  |  |  | A_11_P195143 |  | -2.02 |
|  |  |  | A_11_P091521 | EF-hand domain (C-terminal) containing 1 | -2.02 |
|  |  |  | A_11_P0000019366 |  | -2.02 |
|  |  |  | A_11_P000002484 |  | -2.02 |
|  |  |  | A_11_P118811 | nudix (nucleoside diphosphate linked moiety X)-type motif 3 | -2.02 |
|  |  |  | A_11_P0000012440 |  | -2.02 |
|  |  |  | A_11_P0000010487 |  | -2.02 |
|  |  |  | A_11_P058771 | leishmanolysin-like (metallopeptidase M8 family) | -2.02 |
|  |  |  | A_11_P0000016109 |  | -2.02 |
|  |  |  | A_11_P208808 |  | -2.02 |
|  |  |  | A_11_P139536 |  | -2.02 |
|  |  |  | A_11_P110496 | NHS-like 1 | -2.02 |
|  |  |  | A_11_P0000023431 | vacuolar protein sorting 13 homolog D (S. cerevisiae) | -2.02 |
|  |  |  | A_11_P0000014494 |  | -2.02 |
|  |  |  | A_11_P169608 |  | -2.02 |
|  |  |  | A_11_P081421 | neighbor of BRCA1 gene 1 | -2.02 |
|  |  |  | A_11_P0000033097 | interleukin 4 receptor | -2.02 |
|  |  |  | A_11_P191178 | family with sequence similarity 135, member A | -2.02 |
|  |  |  | A_11_P0000026848 | corticotropin releasing hormone receptor 2 | -2.01 |
|  |  |  | A_11_P052478 | olfactory receptor | -2.01 |
|  |  |  | A_11_P0000041410 |  | -2.01 |
|  |  |  | A_11_P074161 | slingshot protein phosphatase 3 | -2.01 |
|  |  |  | A_11_P0000032292 | sphingosine-1-phosphate lyase 1 | -2.01 |
|  |  |  | A_11_P000006657 |  | -2.01 |
|  |  |  | A_11_P0000029589 | cyclin L1 | -2.01 |
|  |  |  | A_11_P056346 | ataxin 1 | -2.01 |
|  |  |  | A_11_P186478 |  | -2.01 |
|  |  |  | A_11_P000008368 |  | -2.01 |
|  |  |  | A_11_P073821 | cryptochrome 2 (photolyase-like) | -2.01 |
|  |  |  | A_11_P0000025854 | leucine rich repeat containing 10 | -2.01 |
|  |  |  | A_11_P000006171 |  | -2.01 |
|  |  |  | A_11_P104076 | ankyrin 3, node of Ranvier (ankyrin G) | -2.01 |
|  |  |  | A_11_P0000013464 |  | -2.01 |
|  |  |  | A_11_P0000041676 |  | -2.01 |
|  |  |  | A_11_P0000018259 |  | -2.01 |
|  |  |  | A_11_P0000033709 | mitogen-activated protein kinase kinase kinase kinase 5 | -2.01 |
|  |  |  | A_11_P060116 | protein kinase, AMP-activated, beta 1 non-catalytic subunit | -2.01 |
|  |  |  | A_11_P0000015172 |  | -2.01 |
|  |  |  | A_11_P000002402 |  | -2.01 |
|  |  |  | A_11_P189368 |  | -2.01 |
|  |  |  | A_11_P207308 | family with sequence similarity 187, member B | -2.01 |
|  |  |  | A_11_P0000032749 | protein kinase, AMP-activated, alpha 2 catalytic subunit | -2.01 |
|  |  |  | A_11_P083731 | tubulin tyrosine ligase-like family, member 11 | -2.01 |
|  |  |  | A_11_P170193 |  | -2.01 |
|  |  |  | A_11_P0000028746 | basic helix-loop-helix family, member e40 | -2.01 |
|  |  |  | A_11_P0000039364 | solute carrier family 9, subfamily A (NHE1, cation proton antiporter 1), member 1 | -2.01 |
|  |  |  | A_11_P0000025855 | bestrophin 3 | -2.01 |
|  |  |  | A_11_P0000041917 |  | -2.01 |
|  |  |  | A_11_P059446 | Rho guanine nucleotide exchange factor (GEF) 38 | -2.01 |
|  |  |  | A_11_P137211 |  | -2.01 |
|  |  |  | A_11_P186898 |  | -2.01 |
|  |  |  | A_11_P0000011073 |  | -2.01 |
|  |  |  | A_11_P000009102 |  | -2.01 |
|  |  |  | A_11_P0000017179 |  | -2.01 |
|  |  |  | A_11_P171333 |  | -2.01 |
|  |  |  | A_11_P0000011425 |  | -2.01 |
|  |  |  | A_11_P0000028827 | shisa family member 5 | -2.01 |
|  |  |  | A_11_P0000013788 |  | -2.01 |
|  |  |  | A_11_P177683 | arrestin domain containing 1 | -2.01 |
|  |  |  | A_11_P00000447 | Rap guanine nucleotide exchange factor (GEF) 2 | -2.01 |
|  |  |  | A_11_P0000024334 | myozenin 3 | -2.01 |
|  |  |  | A_11_P000005046 |  | -2.01 |
|  |  |  | A_11_P0000081 | catenin (cadherin-associated protein), alpha 1, 102kDa | -2.01 |
|  |  |  | A_11_P00000503 | tetratricopeptide repeat domain 3 | -2.01 |
|  |  |  | A_11_P113121 |  | -2.01 |
|  |  |  | A_11_P0000028037 | CCR4 carbon catabolite repression 4-like (S. cerevisiae) | -2.01 |
|  |  |  | A_11_P055341 | BTG family, member 2 | -2.01 |
|  |  |  | A_11_P108401 | leucine rich repeat containing 36 | -2.00 |
|  |  |  | A_11_P0000027217 | WD repeat domain 60 | -2.00 |
|  |  |  | A_11_P000006034 |  | -2.00 |
|  |  |  | A_11_P0000028298 | guanine deaminase | -2.00 |
|  |  |  | A_11_P063616 | MAX interactor 1, dimerization protein | -2.00 |
|  |  |  | A_11_P0000027806 | olfactory receptor 1052-like | -2.00 |
|  |  |  | A_11_P0000036303 |  | -2.00 |
|  |  |  | A_11_P000003327 |  | -2.00 |
|  |  |  | A_11_P0000030952 | platelet-activating factor receptor | -2.00 |
|  |  |  | A_11_P000007076 |  | -2.00 |
|  |  |  | A_11_P149218 | GTP binding protein 2 | -2.00 |
|  |  |  | A_11_P205863 |  | -2.00 |
|  |  |  | A_11_P0000032133 | otopetrin 1 | -2.00 |
|  |  |  | A_11_P0000022639 | ATPase, class II, type 9A | -2.00 |
|  |  |  | A_11_P0000034111 | RAB11 family interacting protein 4 (class II) | -2.00 |
|  |  |  | A_11_P0000034488 |  | -2.00 |
|  |  |  | A_11_P188518 |  | -2.00 |
|  |  |  | A_11_P126541 | EPH receptor B3 | -2.00 |
|  |  |  | A_11_P078271 | DDHD domain containing 2 | -2.00 |
|  |  |  | A_11_P055651 | dimethylaniline monooxygenase [N-oxide-forming] 5-like | -2.00 |
|  |  |  | A_11_P00000846 | ornithine decarboxylase antizyme 2 | -2.00 |
|  |  |  | A_11_P0000026601 | lymphocyte antigen 6 complex, locus H | -2.00 |
|  |  |  | A_11_P100331 | protein phosphatase 4, regulatory subunit 1 | -2.00 |
|  |  |  | A_11_P0000037211 |  | -2.00 |
|  |  |  | A_11_P188883 |  | -2.00 |
|  |  |  | A_11_P197508 |  | -2.00 |
