# Supplementary material for: Gene expression profiles of the small intestinal mucosa of dogs repeatedly infected with the cestode Echinococcus multilocularis
Source: Data Brief. 2018 Jan 6;17:180–3. doi: 10.1016/j.dib.2018.01.004 (PMC5988226; doi:10.1016/j.dib.2018.01.004)
Supplement: Supplementary file 3 — Supplementary material [file mmc2.docx]

Supplement 2. List of differentially regulated genes between control and reinfection group.

| Upregulation | | | Downregulation | | |
| --- | --- | --- | --- | --- | --- |
| Probe name | Gene name | Fold change | Probe name | Gene name | Fold change |
| A_11_P190123 |  | 51.09 | A_11_P066011 |  | -111.64 |
| A_11_P0000025428 | regakine-1-like | 49.99 | A_11_P0000020956 | paraoxonase 3 | -45.62 |
| A_11_P198223 | periostin, osteoblast specific factor | 48.33 | A_11_P0000015832 |  | -28.56 |
| A_11_P206293 |  | 44.76 | A_11_P0000039861 |  | -23.58 |
| A_11_P160268 |  | 43.15 | A_11_P131751 | cytidine monophosphate (UMP-CMP) kinase 2, mitochondrial | -20.87 |
| A_11_P053591 |  | 43.03 | A_11_P111641 | CD274 molecule | -20.40 |
| A_11_P0000021083 | ribonuclease pancreatic-like | 38.96 | A_11_P000001138 |  | -19.92 |
| A_11_P176483 |  | 34.61 | A_11_P159348 | interferon-related developmental regulator 1 | -17.88 |
| A_11_P188463 |  | 33.91 | A_11_P0000041134 |  | -15.75 |
| A_11_P157453 |  | 33.35 | A_11_P0000039879 |  | -15.32 |
| A_11_P136836 |  | 33.24 | A_11_P114966 | glycerophosphodiester phosphodiesterase domain containing 2 | -14.54 |
| A_11_P151618 |  | 32.96 | A_11_P0000030842 | granzyme A (granzyme 1, cytotoxic T-lymphocyte-associated serine esterase 3) | -14.52 |
| A_11_P179953 |  | 30.32 | A_11_P0000025698 | glycerophosphodiester phosphodiesterase domain containing 2 | -14.29 |
| A_11_P167858 |  | 30.19 | A_11_P0000018515 |  | -13.52 |
| A_11_P156263 |  | 29.21 | A_11_P0000029777 |  | -13.48 |
| A_11_P183038 | glycine amidinotransferase (L-arginine:glycine amidinotransferase) | 28.43 | A_11_P176468 |  | -13.37 |
| A_11_P069866 | periostin, osteoblast specific factor | 28.23 | A_11_P0000038692 |  | -13.32 |
| A_11_P0000029567 | carboxypeptidase A3 (mast cell) | 25.35 | A_11_P000001059 |  | -13.11 |
| A_11_P0000022671 | periostin, osteoblast specific factor | 24.61 | A_11_P000001056 |  | -13.05 |
| A_11_P055286 | tryptase | 24.58 | A_11_P0000019808 | carboxylesterase 1 | -13.05 |
| A_11_P0000020103 | chemokine (C-C motif) ligand 1 | 19.60 | A_11_P053076 | apolipoprotein C-III | -13.02 |
| A_11_P138566 | glycine amidinotransferase (L-arginine:glycine amidinotransferase) | 18.36 | A_11_P0000025427 | chemokine (C-C motif) ligand 3 | -12.69 |
| A_11_P069776 | transcobalamin I (vitamin B12 binding protein, R binder family) | 17.49 | A_11_P062401 |  | -12.61 |
| A_11_P050821 | membrane-spanning 4-domains, subfamily A, member 2 | 16.56 | A_11_P159593 |  | -12.54 |
| A_11_P0000031140 | histidine decarboxylase | 15.36 | A_11_P0000037183 |  | -12.50 |
| A_11_P163398 | interstitial collagenase-like | 13.41 | A_11_P000001055 |  | -12.33 |
| A_11_P00000879 | secreted protein, acidic, cysteine-rich (osteonectin) | 13.30 | A_11_P0000020075 | apolipoprotein C-III | -12.08 |
| A_11_P051436 | fibronectin 1 | 12.94 | A_11_P0000035007 | UBX domain protein 8 | -12.04 |
| A_11_P051431 | fibronectin 1 | 12.85 | A_11_P0000041660 |  | -12.01 |
| A_11_P212743 | neuronal membrane glycoprotein M6-a-like | 12.66 | A_11_P087161 | atlastin GTPase 2 | -11.73 |
| A_11_P060556 | immunoglobulin iota chain-like | 12.62 | A_11_P0000024553 | ISG15 ubiquitin-like modifier | -11.72 |
| A_11_P106336 | interstitial collagenase-like | 11.95 | A_11_P0000024415 | apolipoprotein A-I | -11.47 |
| A_11_P0000015098 | collagen, type VI, alpha 1 | 11.89 | A_11_P000001058 | TCR gamma alternate reading frame protein | -11.47 |
| A_11_P0000019890 | membrane-spanning 4-domains, subfamily A, member 2 | 11.83 | A_11_P0000011690 |  | -11.45 |
| A_11_P0000020112 | chemokine (C-C motif) receptor 3 | 11.66 | A_11_P108186 | HYDIN, axonemal central pair apparatus protein | -11.24 |
| A_11_P193688 | CD1e molecule | 11.52 | A_11_P216593 |  | -11.18 |
| A_11_P114126 | S100 calcium binding protein G | 11.15 | A_11_P000001054 |  | -11.15 |
| A_11_P057266 | FK506 binding protein 7 | 11.01 | A_11_P153298 |  | -10.80 |
| A_11_P141050 |  | 10.93 | A_11_P0000024491 | peripheral myelin protein 22 | -10.71 |
| A_11_P055911 | Fc fragment of IgE, high affinity I, receptor for; alpha polypeptide | 10.67 | A_11_P214443 |  | -10.65 |
| A_11_P054681 |  | 10.41 | A_11_P213008 |  | -10.34 |
| A_11_P0000024685 | serpin peptidase inhibitor, clade E (nexin, plasminogen activator inhibitor type 1), member 1 | 10.31 | A_11_P079681 | interferon-related developmental regulator 1 | -10.31 |
| A_11_P000005639 |  | 10.08 | A_11_P072951 | CD36 molecule (thrombospondin receptor) | -10.19 |
| A_11_P089526 | neutrophil cytosolic factor 4, 40kDa | 9.81 | A_11_P173603 |  | -10.14 |
| A_11_P0000022171 | egf-like module containing, mucin-like, hormone receptor-like 2 | 9.68 | A_11_P0000039817 |  | -9.96 |
| A_11_P110951 |  | 9.66 | A_11_P0000019736 | chemokine (C-C motif) ligand 5 | -9.89 |
| A_11_P200643 |  | 9.66 | A_11_P0000032314 | sterile alpha motif domain containing 8 | -9.86 |
| A_11_P104976 |  | 9.62 | A_11_P0000024626 | HYDIN, axonemal central pair apparatus protein | -9.82 |
| A_11_P117196 |  | 9.54 | A_11_P000006423 |  | -9.80 |
| A_11_P083221 | surfeit 6 | 9.40 | A_11_P0000015549 |  | -9.77 |
| A_11_P0000019776 | chemokine (C-C motif) ligand 17 | 9.25 | A_11_P113041 | exocyst complex component 3-like 2 | -9.73 |
| A_11_P124036 | cholesterol 25-hydroxylase | 9.16 | A_11_P0000014359 |  | -9.71 |
| A_11_P0000018663 |  | 9.06 | A_11_P167208 |  | -9.55 |
| A_11_P068481 | protease, serine, 23 | 9.04 | A_11_P0000014525 |  | -9.53 |
| A_11_P0000039557 | protease, serine, 23 | 9.01 | A_11_P092121 | solute carrier family 16 (aromatic amino acid transporter), member 10 | -9.51 |
| A_11_P147118 | collagen, type I, alpha 2 | 9.01 | A_11_P087826 | synaptonemal complex protein 1 | -9.49 |
| A_11_P053801 | TIMP metallopeptidase inhibitor 1 | 8.74 | A_11_P051251 | keratin 12 | -9.41 |
| A_11_P104019 |  | 8.70 | A_11_P216803 |  | -9.32 |
| A_11_P109436 |  | 8.64 | A_11_P118436 | solute carrier family 4, sodium bicarbonate cotransporter, member 8 | -9.30 |
| A_11_P054581 |  | 8.63 | A_11_P0000019772 | uncoupling protein 3 (mitochondrial, proton carrier) | -9.26 |
| A_11_P054721 |  | 8.56 | A_11_P219313 | keratin 12 | -9.26 |
| A_11_P0000033532 | S100 calcium binding protein A9 | 8.55 | A_11_P191308 |  | -9.18 |
| A_11_P0000025577 |  | 8.52 | A_11_P0000033517 | nucleoporin 210kDa-like | -9.16 |
| A_11_P0000016527 |  | 8.48 | A_11_P000008274 |  | -9.15 |
| A_11_P144638 | four and a half LIM domains 3 | 8.37 | A_11_P0000033713 | guanine nucleotide binding protein (G protein), gamma 2 | -9.05 |
| A_11_P00000884 | annexin A6 | 8.36 | A_11_P0000019663 | CD8a molecule | -9.02 |
| A_11_P0000015620 | PDZ and LIM domain 3 | 8.20 | A_11_P054966 | chymotrypsin-like elastase family, member 1 | -8.96 |
| A_11_P087926 | 3-hydroxy-3-methylglutaryl-CoA synthase 2 (mitochondrial) | 8.20 | A_11_P0000041368 | delta(4)-desaturase, sphingolipid 1 | -8.91 |
| A_11_P0000020008 | caveolin 1, caveolae protein, 22kDa | 8.12 | A_11_P146398 |  | -8.88 |
| A_11_P0000019899 | v-kit Hardy-Zuckerman 4 feline sarcoma viral oncogene homolog | 8.08 | A_11_P126066 | keratin 5 | -8.77 |
| A_11_P0000031208 |  | 8.00 | A_11_P067681 | WNT1 inducible signaling pathway protein 2 | -8.74 |
| A_11_P201848 |  | 7.99 | A_11_P0000040107 |  | -8.63 |
| A_11_P204503 | clusterin | 7.93 | A_11_P190598 | membrane metallo-endopeptidase | -8.61 |
| A_11_P0000040655 |  | 7.90 | A_11_P0000019170 |  | -8.58 |
| A_11_P0000014930 |  | 7.87 | A_11_P0000041943 |  | -8.56 |
| A_11_P111551 | phosphoglucomutase 5 | 7.87 | A_11_P050686 | cytochrome P450 2B11 | -8.53 |
| A_11_P056966 | fibroblast activation protein, alpha | 7.87 | A_11_P0000015182 |  | -8.43 |
| A_11_P060601 |  | 7.85 | A_11_P000007662 |  | -8.41 |
| A_11_P0000015308 | stathmin-like 2 | 7.81 | A_11_P000001061 |  | -8.35 |
| A_11_P179813 |  | 7.74 | A_11_P191753 | tripartite motif containing 36 | -8.27 |
| A_11_P077796 | pleiotrophin | 7.70 | A_11_P0000024251 | HECT and RLD domain containing E3 ubiquitin protein ligase 4 | -8.27 |
| A_11_P0000019527 |  | 7.64 | A_11_P051501 | 2'-5'-oligoadenylate synthetase-like | -8.23 |
| A_11_P0000031801 | FK506 binding protein 7 | 7.62 | A_11_P0000040471 | angiotensinogen (serpin peptidase inhibitor, clade A, member 8) | -8.20 |
| A_11_P133746 | protease, serine, 23 | 7.62 | A_11_P0000019733 | chymotrypsin-like elastase family, member 1 | -8.20 |
| A_11_P104971 | secreted protein, acidic, cysteine-rich (osteonectin) | 7.60 | A_11_P0000040074 |  | -8.13 |
| A_11_P089701 | aldehyde dehydrogenase 1 family, member L2 | 7.56 | A_11_P213728 |  | -8.11 |
| A_11_P0000020185 | desmin | 7.55 | A_11_P0000018359 |  | -8.09 |
| A_11_P190128 |  | 7.50 | A_11_P218563 | T cell receptor associated transmembrane adaptor 1 | -7.96 |
| A_11_P0000033531 | S100 calcium binding protein A12 | 7.38 | A_11_P0000024372 |  | -7.89 |
| A_11_P099176 | selectin L | 7.28 | A_11_P0000015804 |  | -7.86 |
| A_11_P205483 |  | 7.26 | A_11_P0000021311 | apolipoprotein B (including Ag(x) antigen) | -7.81 |
| A_11_P000004603 |  | 7.12 | A_11_P052386 | angiotensin-converting enzyme-like | -7.74 |
| A_11_P0000020111 | mastin | 7.10 | A_11_P0000016134 |  | -7.74 |
| A_11_P084736 | insulin-like growth factor binding protein 7 | 7.05 | A_11_P0000012254 |  | -7.69 |
| A_11_P0000029155 | malic enzyme 3, NADP(+)-dependent, mitochondrial | 6.93 | A_11_P0000017995 |  | -7.65 |
| A_11_P185333 | stathmin-like 2 | 6.90 | A_11_P0000038773 |  | -7.59 |
| A_11_P000002562 |  | 6.88 | A_11_P177398 | retinoic acid induced 14 | -7.51 |
| A_11_P0000032127 | fibroblast growth factor-binding protein 1-like | 6.84 | A_11_P0000041688 |  | -7.45 |
| A_11_P0000023290 | glutathione peroxidase 8 (putative) | 6.83 | A_11_P0000014592 |  | -7.42 |
| A_11_P0000033963 | FK506 binding protein 10, 65 kDa | 6.79 | A_11_P0000018382 |  | -7.35 |
| A_11_P138726 |  | 6.78 | A_11_P206953 |  | -7.32 |
| A_11_P0000015040 | solute carrier family 16, member 9 | 6.69 | A_11_P084936 | epiregulin | -7.30 |
| A_11_P0000025517 | prostaglandin E synthase | 6.66 | A_11_P0000031250 | transmembrane protease, serine 15 | -7.28 |
| A_11_P0000023718 | coiled-coil domain containing 80 | 6.66 | A_11_P00000945 | N-acetylglucosamine-1-phosphodiester alpha-N-acetylglucosaminidase | -7.25 |
| A_11_P050041 | prostaglandin E synthase | 6.63 | A_11_P053491 | uncoupling protein 3 (mitochondrial, proton carrier) | -7.24 |
| A_11_P0000023937 | collagen, type V, alpha 2 | 6.62 | A_11_P0000026289 | tumor necrosis factor (ligand) superfamily, member 15 | -7.23 |
| A_11_P216443 | peptidyl arginine deiminase, type IV | 6.61 | A_11_P00000295 | ring finger protein 213 | -7.15 |
| A_11_P055056 | tryptase | 6.61 | A_11_P098266 |  | -7.06 |
| A_11_P0000040664 |  | 6.56 | A_11_P0000025004 | kynurenine 3-monooxygenase (kynurenine 3-hydroxylase) | -7.06 |
| A_11_P053366 | hemoglobin subunit beta-like | 6.55 | A_11_P0000036968 |  | -6.97 |
| A_11_P087831 | tetraspanin 2 | 6.54 | A_11_P0000022512 | membrane metallo-endopeptidase | -6.96 |
| A_11_P0000032983 | procollagen C-endopeptidase enhancer | 6.48 | A_11_P178948 |  | -6.96 |
| A_11_P0000020109 | chemokine (C-C motif) ligand 21 | 6.42 | A_11_P0000017066 |  | -6.94 |
| A_11_P0000040294 | heparanase 2 | 6.41 | A_11_P0000019491 |  | -6.89 |
| A_11_P167963 |  | 6.40 | A_11_P084641 | FRY-like | -6.88 |
| A_11_P217073 |  | 6.39 | A_11_P123656 |  | -6.88 |
| A_11_P162343 | fibrinogen-like 1 | 6.38 | A_11_P170543 |  | -6.85 |
| A_11_P0000020202 | chymase 1, mast cell | 6.38 | A_11_P0000039503 |  | -6.82 |
| A_11_P218668 |  | 6.38 | A_11_P206083 |  | -6.82 |
| A_11_P190298 | hemoglobin subunit beta-like | 6.38 | A_11_P162258 | angiotensin-converting enzyme-like | -6.80 |
| A_11_P0000018686 | collagen, type VI, alpha 1 | 6.35 | A_11_P0000041969 |  | -6.79 |
| A_11_P079441 | neuropeptide Y | 6.32 | A_11_P180118 |  | -6.78 |
| A_11_P0000021417 | actin, gamma 2, smooth muscle, enteric | 6.31 | A_11_P0000033833 |  | -6.76 |
| A_11_P060741 |  | 6.29 | A_11_P000001139 |  | -6.75 |
| A_11_P052991 | matrix metallopeptidase 2 (gelatinase A, 72kDa gelatinase, 72kDa type IV collagenase) | 6.29 | A_11_P133716 | amphiphysin | -6.70 |
| A_11_P212903 | clusterin | 6.28 | A_11_P066186 | G protein-coupled receptor, family C, group 5, member A | -6.68 |
| A_11_P178658 |  | 6.28 | A_11_P148643 |  | -6.66 |
| A_11_P054666 |  | 6.27 | A_11_P096083 |  | -6.61 |
| A_11_P054676 |  | 6.26 | A_11_P205223 |  | -6.58 |
| A_11_P182298 | junctional adhesion molecule 2 | 6.25 | A_11_P148998 |  | -6.56 |
| A_11_P050631 | collagen, type I, alpha 1 | 6.20 | A_11_P0000041077 |  | -6.56 |
| A_11_P0000027724 |  | 6.16 | A_11_P000001765 | phosphoenolpyruvate carboxykinase 1 (soluble) | -6.54 |
| A_11_P138311 |  | 6.15 | A_11_P0000022436 | tetratricopeptide repeat and ankyrin repeat containing 1 | -6.54 |
| A_11_P060584 |  | 6.15 | A_11_P0000022873 | leukemia inhibitory factor (cholinergic differentiation factor) | -6.53 |
| A_11_P0000026779 | frizzled family receptor 1 | 6.14 | A_11_P0000019570 |  | -6.51 |
| A_11_P0000039218 |  | 6.11 | A_11_P089851 | ring finger protein 149 | -6.51 |
| A_11_P116396 | collagen, type IV, alpha 1 | 6.10 | A_11_P0000024903 | interferon-induced protein 44-like | -6.50 |
| A_11_P0000029513 | leucine rich repeat containing 3B | 6.09 | A_11_P0000040110 |  | -6.48 |
| A_11_P203419 |  | 6.07 | A_11_P0000011730 |  | -6.46 |
| A_11_P0000020121 | hydroxysteroid (11-beta) dehydrogenase 1 | 6.04 | A_11_P0000010409 |  | -6.46 |
| A_11_P218848 | ST6 (alpha-N-acetyl-neuraminyl-2,3-beta-galactosyl-1,3)-N-acetylgalactosaminide alpha-2,6-sialyltransferase 1 | 6.02 | A_11_P000005396 |  | -6.45 |
| A_11_P055916 | Duffy blood group, atypical chemokine receptor | 6.02 | A_11_P150518 |  | -6.43 |
| A_11_P174938 |  | 6.02 | A_11_P0000031534 | solute carrier family 6 (neutral amino acid transporter), member 19 | -6.41 |
| A_11_P054711 |  | 6.00 | A_11_P0000016168 |  | -6.38 |
| A_11_P187538 |  | 5.99 | A_11_P075861 | transmembrane protein 89 | -6.38 |
| A_11_P109866 |  | 5.97 | A_11_P050691 | 2'-5'-oligoadenylate synthetase 1, 40/46kDa | -6.34 |
| A_11_P119506 |  | 5.96 | A_11_P0000012160 |  | -6.32 |
| A_11_P052371 | collagen, type IV, alpha 2 | 5.94 | A_11_P0000040309 |  | -6.32 |
| A_11_P194308 | major histocompatibility complex, class II, DM beta | 5.94 | A_11_P0000024368 | retinoic acid induced 14 | -6.31 |
| A_11_P123561 |  | 5.92 | A_11_P0000016329 |  | -6.31 |
| A_11_P0000023806 | schwannomin interacting protein 1 | 5.89 | A_11_P0000020101 | chemokine (C-C motif) ligand 4 | -6.30 |
| A_11_P141818 |  | 5.88 | A_11_P0000025072 | laminin, alpha 3 | -6.26 |
| A_11_P094186 |  | 5.84 | A_11_P000003876 |  | -6.26 |
| A_11_P0000038736 | Thy-1 cell surface antigen | 5.83 | A_11_P0000022385 | LIM domain 7 | -6.25 |
| A_11_P0000022406 |  | 5.82 | A_11_P092381 |  | -6.22 |
| A_11_P060611 |  | 5.82 | A_11_P0000019083 |  | -6.22 |
| A_11_P095311 | tropomyosin 2 (beta) | 5.82 | A_11_P0000030985 | grainyhead-like 3 (Drosophila) | -6.21 |
| A_11_P133791 | neuronal membrane glycoprotein M6-a-like | 5.79 | A_11_P080626 | protein phosphatase 1, regulatory subunit 27 | -6.20 |
| A_11_P0000024446 | matrix metallopeptidase 12 (macrophage elastase) | 5.79 | A_11_P055476 | family with sequence similarity 72, member A | -6.17 |
| A_11_P199848 | fascin homolog 1, actin-bundling protein (Strongylocentrotus purpuratus) | 5.79 | A_11_P052741 | lectin, galactoside-binding, soluble, 3 | -6.17 |
| A_11_P094216 |  | 5.75 | A_11_P0000022829 | TRAF-type zinc finger domain containing 1 | -6.14 |
| A_11_P0000023235 | vimentin | 5.75 | A_11_P186968 |  | -6.09 |
| A_11_P078451 | fibrinogen-like 1 | 5.75 | A_11_P202233 |  | -6.08 |
| A_11_P0000016456 |  | 5.72 | A_11_P0000020593 | catenin (cadherin-associated protein), alpha-like 1 | -6.05 |
| A_11_P192543 | apolipoprotein E | 5.66 | A_11_P0000015806 |  | -6.03 |
| A_11_P0000024961 | phospholipase A2, group IVA (cytosolic, calcium-dependent) | 5.63 | A_11_P095571 | catenin (cadherin-associated protein), alpha-like 1 | -6.02 |
| A_11_P084686 | v-kit Hardy-Zuckerman 4 feline sarcoma viral oncogene homolog | 5.63 | A_11_P0000030420 | killer cell lectin-like receptor subfamily K, member 1 | -6.02 |
| A_11_P0000020124 | gastric intrinsic factor (vitamin B synthesis) | 5.62 | A_11_P051846 | endothelin 3 | -6.01 |
| A_11_P060751 |  | 5.62 | A_11_P189253 |  | -5.99 |
| A_11_P115586 | plastin 3 | 5.62 | A_11_P098176 | chloride channel accessory 4 | -5.99 |
| A_11_P060756 |  | 5.62 | A_11_P0000041375 |  | -5.99 |
| A_11_P0000027851 | spleen focus forming virus (SFFV) proviral integration oncogene | 5.62 | A_11_P211903 |  | -5.99 |
| A_11_P0000015580 | caveolin 1, caveolae protein, 22kDa | 5.62 | A_11_P070621 | transmembrane 4 L six family member 20 | -5.97 |
| A_11_P134161 | synaptopodin 2 | 5.62 | A_11_P063791 | inositol polyphosphate-5-phosphatase F | -5.96 |
| A_11_P054732 |  | 5.60 | A_11_P156033 | deltex 3-like (Drosophila) | -5.96 |
| A_11_P0000039612 | phospholipase C-like 1 | 5.60 | A_11_P0000039979 |  | -5.95 |
| A_11_P054686 |  | 5.55 | A_11_P193963 |  | -5.94 |
| A_11_P0000017318 | retinoic acid induced 2 | 5.55 | A_11_P0000015759 |  | -5.91 |
| A_11_P0000029111 | complement factor D (adipsin) | 5.53 | A_11_P087551 |  | -5.91 |
| A_11_P201543 | collagen, type I, alpha 2 | 5.52 | A_11_P190728 | spectrin repeat containing, nuclear envelope 1 | -5.90 |
| A_11_P094241 |  | 5.50 | A_11_P0000014147 |  | -5.89 |
| A_11_P0000028503 | epithelial membrane protein 3 | 5.50 | A_11_P100446 | CBP80/20-dependent translation initiation factor | -5.87 |
| A_11_P0000027330 | potassium voltage-gated channel, delayed-rectifier, subfamily S, member 3 | 5.49 | A_11_P058466 |  | -5.84 |
| A_11_P167993 | platelet derived growth factor D | 5.48 | A_11_P175823 |  | -5.84 |
| A_11_P187783 | cysteine and glycine-rich protein 1-like | 5.48 | A_11_P000006338 |  | -5.83 |
| A_11_P0000016647 | claudin 2 | 5.44 | A_11_P063786 | inositol polyphosphate-5-phosphatase F | -5.78 |
| A_11_P054282 |  | 5.43 | A_11_P0000041522 |  | -5.77 |
| A_11_P086446 | family with sequence similarity 198, member B | 5.43 | A_11_P051101 | killer cell lectin-like receptor subfamily D, member 1 | -5.75 |
| A_11_P058286 | filamin A interacting protein 1-like | 5.41 | A_11_P198343 |  | -5.74 |
| A_11_P136986 | cadherin 11, type 2, OB-cadherin (osteoblast) | 5.41 | A_11_P052381 | myosin, light chain 4, alkali; atrial, embryonic | -5.73 |
| A_11_P0000022407 | collagen, type IV, alpha 2 | 5.39 | A_11_P149463 | lectin, galactoside-binding, soluble, 3 | -5.73 |
| A_11_P000003239 |  | 5.39 | A_11_P071406 | NIMA-related kinase 10 | -5.72 |
| A_11_P0000040546 |  | 5.38 | A_11_P122586 | myopalladin | -5.71 |
| A_11_P0000028474 | reticulocalbin 3, EF-hand calcium binding domain | 5.37 | A_11_P189538 |  | -5.70 |
| A_11_P0000024047 | Fc fragment of IgE, high affinity I, receptor for; alpha polypeptide | 5.37 | A_11_P0000010570 |  | -5.70 |
| A_11_P0000020370 | apolipoprotein L, 5 | 5.37 | A_11_P195408 | O-linked N-acetylglucosamine (GlcNAc) transferase | -5.69 |
| A_11_P138721 |  | 5.36 | A_11_P0000019487 |  | -5.69 |
| A_11_P196708 |  | 5.36 | A_11_P107266 | jun proto-oncogene | -5.68 |
| A_11_P094231 |  | 5.35 | A_11_P160628 | catsper channel auxiliary subunit gamma | -5.67 |
| A_11_P051236 | collagen, type I, alpha 2 | 5.35 | A_11_P087991 | CD160 molecule | -5.67 |
| A_11_P050656 | transcription factor 4 | 5.35 | A_11_P059231 | HECT and RLD domain containing E3 ubiquitin protein ligase family member 6 | -5.67 |
| A_11_P094101 |  | 5.35 | A_11_P0000029776 | phosphoenolpyruvate carboxykinase 1 (soluble) | -5.65 |
| A_11_P109326 | chromosome 3 open reading frame, human C15orf40 | 5.35 | A_11_P062486 |  | -5.64 |
| A_11_P109871 | protocadherin 7 | 5.33 | A_11_P0000030484 | ubiquitin specific peptidase 18 | -5.63 |
| A_11_P087786 | olfactomedin-like 3 | 5.33 | A_11_P199633 |  | -5.63 |
| A_11_P115296 | armadillo repeat containing, X-linked 2 | 5.32 | A_11_P0000039986 |  | -5.60 |
| A_11_P00000760 |  | 5.31 | A_11_P176753 |  | -5.59 |
| A_11_P0000021854 | annexin A1 | 5.31 | A_11_P175388 |  | -5.59 |
| A_11_P094304 | fms-related tyrosine kinase 4 | 5.29 | A_11_P133851 |  | -5.57 |
| A_11_P122326 |  | 5.29 | A_11_P134056 |  | -5.57 |
| A_11_P000003247 | bicaudal C homolog 1 (Drosophila) | 5.29 | A_11_P0000017151 |  | -5.57 |
| A_11_P0000021095 | muscle LIM protein Mlp84B-like | 5.29 | A_11_P051951 | alkaline phosphatase, placental | -5.56 |
| A_11_P0000018223 |  | 5.27 | A_11_P105281 |  | -5.55 |
| A_11_P116616 | collagen, type V, alpha 2 | 5.27 | A_11_P0000015594 | protein phosphatase 1, regulatory subunit 27 | -5.54 |
| A_11_P0000020107 | chemokine (C-C motif) ligand 19 | 5.27 | A_11_P0000038034 |  | -5.54 |
| A_11_P198578 | transcription factor 21 | 5.26 | A_11_P0000033903 | angiotensin-converting enzyme-like | -5.53 |
| A_11_P062741 |  | 5.26 | A_11_P0000041702 |  | -5.53 |
| A_11_P0000017700 | protein kinase, cGMP-dependent, type I | 5.24 | A_11_P068081 | synaptonemal complex protein 2 | -5.53 |
| A_11_P0000020011 | potassium large conductance calcium-activated channel, subfamily M, beta member 1 | 5.24 | A_11_P000005655 |  | -5.50 |
| A_11_P198188 |  | 5.23 | A_11_P212348 | uncharacterized LOC100688518 | -5.48 |
| A_11_P0000019946 | decorin | 5.22 | A_11_P058361 | nuclear factor of kappa light polypeptide gene enhancer in B-cells inhibitor, zeta | -5.48 |
| A_11_P050831 | ST6 (alpha-N-acetyl-neuraminyl-2,3-beta-galactosyl-1,3)-N-acetylgalactosaminide alpha-2,6-sialyltransferase 1 | 5.17 | A_11_P000002434 | shroom family member 1 | -5.48 |
| A_11_P101211 |  | 5.16 | A_11_P123391 |  | -5.47 |
| A_11_P0000031875 | insulin-like growth factor binding protein 5 | 5.16 | A_11_P205498 | abhydrolase domain containing 2 | -5.46 |
| A_11_P0000022501 | transmembrane 4 L six family member 18 | 5.15 | A_11_P0000013414 |  | -5.43 |
| A_11_P145753 |  | 5.11 | A_11_P0000038801 | phosphoenolpyruvate carboxykinase 1 (soluble) | -5.42 |
| A_11_P060656 |  | 5.09 | A_11_P060006 | 2'-5'-oligoadenylate synthetase 1, 40/46kDa | -5.38 |
| A_11_P106172 | crystallin, alpha B | 5.09 | A_11_P118361 |  | -5.37 |
| A_11_P126571 | glycoprotein (transmembrane) nmb | 5.08 | A_11_P0000027564 |  | -5.37 |
| A_11_P0000015569 |  | 5.07 | A_11_P0000011744 |  | -5.37 |
| A_11_P073356 |  | 5.06 | A_11_P0000039973 |  | -5.36 |
| A_11_P127996 |  | 5.06 | A_11_P0000039701 |  | -5.35 |
| A_11_P176163 |  | 5.04 | A_11_P165373 |  | -5.35 |
| A_11_P063016 | arachidonate 5-lipoxygenase | 5.03 | A_11_P168128 |  | -5.33 |
| A_11_P053836 | IgA heavy chain constant region | 5.03 | A_11_P000003141 |  | -5.33 |
| A_11_P185448 | SWI/SNF related, matrix associated, actin dependent regulator of chromatin, subfamily d, member 3 | 5.02 | A_11_P177553 |  | -5.32 |
| A_11_P185793 |  | 5.02 | A_11_P176773 |  | -5.32 |
| A_11_P104401 | peptidylprolyl isomerase F | 5.01 | A_11_P0000035533 | activating transcription factor 3 | -5.31 |
| A_11_P000005367 |  | 5.01 | A_11_P159858 |  | -5.30 |
| A_11_P054596 |  | 5.01 | A_11_P0000025248 | tubulin tyrosine ligase-like family, member 5 | -5.30 |
| A_11_P180308 | junctional adhesion molecule 2 | 5.00 | A_11_P0000020347 | DnaJ (Hsp40) homolog, subfamily B, member 7 | -5.30 |
| A_11_P165933 | cytochrome P450, family 26, subfamily B, polypeptide 1 | 5.00 | A_11_P170688 |  | -5.30 |
| A_11_P0000024332 | annexin A6 | 5.00 | A_11_P111651 | CD274 molecule | -5.27 |
| A_11_P061216 | junctional adhesion molecule 2 | 5.00 | A_11_P198518 |  | -5.27 |
| A_11_P054698 |  | 5.00 | A_11_P055481 | SLIT-ROBO Rho GTPase activating protein 2 | -5.27 |
| A_11_P189328 |  | 4.99 | A_11_P117821 | MAX interactor 1, dimerization protein | -5.27 |
| A_11_P192433 | tropomyosin 2 (beta) | 4.99 | A_11_P0000028711 | glutamate receptor interacting protein 2 | -5.24 |
| A_11_P126011 | chromosome 3 open reading frame, human C15orf40 | 4.98 | A_11_P061409 | carbonyl reductase [NADPH] 1-like | -5.24 |
| A_11_P0000018243 | CD74 molecule, major histocompatibility complex, class II invariant chain | 4.98 | A_11_P0000017971 |  | -5.23 |
| A_11_P0000025278 | adenylate kinase 7 | 4.94 | A_11_P0000016141 |  | -5.22 |
| A_11_P0000033887 |  | 4.93 | A_11_P058421 | CD96 molecule | -5.21 |
| A_11_P059506 |  | 4.93 | A_11_P0000018102 |  | -5.20 |
| A_11_P105036 | CD74 molecule, major histocompatibility complex, class II invariant chain | 4.92 | A_11_P127591 | killer cell lectin-like receptor subfamily D, member 1 | -5.20 |
| A_11_P054218 |  | 4.91 | A_11_P0000017478 |  | -5.20 |
| A_11_P069406 | suppression of tumorigenicity 5 | 4.90 | A_11_P102981 | ring finger protein 19B | -5.20 |
| A_11_P0000016814 | 5'-nucleotidase, ecto (CD73) | 4.89 | A_11_P0000018530 |  | -5.17 |
| A_11_P0000018425 |  | 4.88 | A_11_P0000017007 |  | -5.17 |
| A_11_P187798 |  | 4.87 | A_11_P000009759 |  | -5.17 |
| A_11_P170223 |  | 4.87 | A_11_P0000014463 |  | -5.15 |
| A_11_P0000039283 | serpin peptidase inhibitor, clade H (heat shock protein 47), member 1, (collagen binding protein 1) | 4.86 | A_11_P139666 |  | -5.14 |
| A_11_P060571 | uncharacterized LOC612122 | 4.85 | A_11_P0000012883 |  | -5.14 |
| A_11_P202564 | transgelin | 4.85 | A_11_P086316 | nuclear receptor subfamily 3, group C, member 2 | -5.14 |
| A_11_P102441 | ankyrin repeat domain 55 | 4.84 | A_11_P0000017358 |  | -5.13 |
| A_11_P060716 |  | 4.84 | A_11_P115771 | G protein-coupled receptor 119 | -5.13 |
| A_11_P204798 | double C2-like domains, beta | 4.83 | A_11_P062431 | myosin IE | -5.12 |
| A_11_P0000035026 |  | 4.83 | A_11_P206658 |  | -5.12 |
| A_11_P0000021929 | carnitine palmitoyltransferase 1C | 4.83 | A_11_P129271 |  | -5.11 |
| A_11_P138911 |  | 4.83 | A_11_P149498 |  | -5.11 |
| A_11_P0000020226 | major histocompatibility complex, class II, DQ beta 1 | 4.81 | A_11_P086761 | apolipoprotein B (including Ag(x) antigen) | -5.11 |
| A_11_P148083 | tissue factor pathway inhibitor (lipoprotein-associated coagulation inhibitor) | 4.81 | A_11_P0000033299 |  | -5.10 |
| A_11_P060626 |  | 4.80 | A_11_P000007494 |  | -5.10 |
| A_11_P0000041359 | solute carrier family 12, member 8 | 4.80 | A_11_P150708 | integrin, alpha 6 | -5.09 |
| A_11_P0000013757 |  | 4.79 | A_11_P000003578 |  | -5.07 |
| A_11_P0000021194 | retinoic acid receptor responder (tazarotene induced) 2 | 4.78 | A_11_P0000040725 |  | -5.07 |
| A_11_P192703 |  | 4.75 | A_11_P176593 | ankyrin 3, node of Ranvier (ankyrin G) | -5.06 |
| A_11_P000001761 | immunoglobulin J polypeptide, linker protein for immunoglobulin alpha and mu polypeptides | 4.75 | A_11_P0000021924 | family with sequence similarity 71, member E1 | -5.06 |
| A_11_P0000027938 | CD248 molecule, endosialin | 4.74 | A_11_P0000020044 | cytochrome P450 2C41 | -5.06 |
| A_11_P163768 | collagen, type V, alpha 2 | 4.73 | A_11_P202638 | chymotrypsin-like | -5.05 |
| A_11_P0000014916 | neuron specific gene family member 1 | 4.72 | A_11_P109176 | interferon stimulated exonuclease gene 20kDa | -5.04 |
| A_11_P0000031005 | cytidine deaminase | 4.71 | A_11_P0000040658 | myopalladin | -5.04 |
| A_11_P166948 | solute carrier family 12, member 8 | 4.70 | A_11_P0000028141 | one cut homeobox 2 | -5.03 |
| A_11_P136716 | hemoglobin, delta-like | 4.70 | A_11_P0000032585 | CD3g molecule, gamma (CD3-TCR complex) | -5.03 |
| A_11_P0000015957 |  | 4.70 | A_11_P0000040946 |  | -5.02 |
| A_11_P178353 | immunoglobulin J polypeptide, linker protein for immunoglobulin alpha and mu polypeptides | 4.70 | A_11_P0000026834 | oxysterol binding protein-like 3 | -5.00 |
| A_11_P0000014606 |  | 4.69 | A_11_P050331 | CD8a molecule | -4.98 |
| A_11_P0000015751 |  | 4.68 | A_11_P086466 |  | -4.97 |
| A_11_P000002593 | integrin, alpha 5 (fibronectin receptor, alpha polypeptide) | 4.68 | A_11_P208958 | chymotrypsin-like | -4.96 |
| A_11_P0000041342 |  | 4.65 | A_11_P0000028731 | cell death-inducing DFFA-like effector c | -4.94 |
| A_11_P179718 |  | 4.65 | A_11_P00000293 | NIMA-related kinase 10 | -4.94 |
| A_11_P107946 | WAP four-disulfide core domain 1 | 4.65 | A_11_P0000016906 |  | -4.94 |
| A_11_P058891 | apolipoprotein D | 4.65 | A_11_P114196 |  | -4.94 |
| A_11_P094171 |  | 4.63 | A_11_P128901 |  | -4.94 |
| A_11_P0000020227 | MHC class II DLA DRB1 beta chain | 4.60 | A_11_P160308 |  | -4.93 |
| A_11_P170183 | latrophilin 2 | 4.60 | A_11_P181238 | lectin, galactoside-binding, soluble, 8 | -4.92 |
| A_11_P0000016233 | FK506 binding protein 1B, 12.6 kDa | 4.60 | A_11_P0000032558 | GRAM domain containing 1B | -4.92 |
| A_11_P180523 |  | 4.57 | A_11_P151488 |  | -4.92 |
| A_11_P0000019905 | collagen, type I, alpha 2 | 4.57 | A_11_P0000023821 |  | -4.91 |
| A_11_P0000040458 | WAP four-disulfide core domain 1 | 4.57 | A_11_P211553 |  | -4.91 |
| A_11_P0000019247 |  | 4.57 | A_11_P189168 |  | -4.90 |
| A_11_P122486 |  | 4.57 | A_11_P200968 |  | -4.89 |
| A_11_P052666 | major histocompatibility complex, class II, DM beta | 4.56 | A_11_P099836 | S100 calcium binding protein A14 | -4.89 |
| A_11_P0000022913 |  | 4.56 | A_11_P0000041761 |  | -4.89 |
| A_11_P0000023046 | chromosome 28 open reading frame, human C10orf10 | 4.56 | A_11_P104141 | HECT and RLD domain containing E3 ubiquitin protein ligase 4 | -4.88 |
| A_11_P103166 | thymocyte selection associated family member 2 | 4.52 | A_11_P152253 | diacylglycerol O-acyltransferase 2 | -4.87 |
| A_11_P000002695 | heat shock 22kDa protein 8 | 4.52 | A_11_P0000040627 |  | -4.87 |
| A_11_P203823 |  | 4.50 | A_11_P067886 | solute carrier family 9, subfamily A (NHE8, cation proton antiporter 8), member 8 | -4.86 |
| A_11_P0000030635 | snail homolog 2 (Drosophila) | 4.49 | A_11_P185673 |  | -4.85 |
| A_11_P176953 |  | 4.49 | A_11_P216373 |  | -4.85 |
| A_11_P192643 | vimentin | 4.48 | A_11_P0000011177 |  | -4.85 |
| A_11_P0000019702 | Fc fragment of IgG, high affinity Ia, receptor (CD64) | 4.48 | A_11_P129361 | chromosome 8 open reading frame, human C14orf37 | -4.85 |
| A_11_P054741 |  | 4.44 | A_11_P0000029557 | RAS p21 protein activator 2 | -4.85 |
| A_11_P054196 |  | 4.44 | A_11_P0000012684 |  | -4.84 |
| A_11_P0000019900 | TIMP metallopeptidase inhibitor 1 | 4.43 | A_11_P0000020498 | solute carrier family 22 (organic cation/carnitine transporter), member 5 | -4.84 |
| A_11_P0000015006 |  | 4.43 | A_11_P180383 | inositol polyphosphate-5-phosphatase F | -4.83 |
| A_11_P0000033430 | dermatopontin | 4.42 | A_11_P0000021163 | DEAD (Asp-Glu-Ala-Asp) box polypeptide 60 | -4.83 |
| A_11_P147268 | collagen, type I, alpha 2 | 4.42 | A_11_P135171 |  | -4.83 |
| A_11_P0000024439 | guanylate cyclase 1, soluble, alpha 2 | 4.41 | A_11_P0000019893 | vascular endothelial growth factor A | -4.82 |
| A_11_P0000036880 |  | 4.40 | A_11_P0000018997 | XIAP associated factor 1 | -4.81 |
| A_11_P0000034950 | chloride intracellular channel 2 | 4.40 | A_11_P170323 |  | -4.80 |
| A_11_P078561 | MLF1 interacting protein | 4.38 | A_11_P0000041156 |  | -4.78 |
| A_11_P0000023453 |  | 4.37 | A_11_P0000040151 |  | -4.78 |
| A_11_P0000016223 | delta/notch-like EGF repeat containing | 4.37 | A_11_P0000032172 |  | -4.77 |
| A_11_P0000025297 |  | 4.37 | A_11_P109191 | abhydrolase domain containing 2 | -4.76 |
| A_11_P0000026810 | anterior gradient 2 | 4.37 | A_11_P109186 | abhydrolase domain containing 2 | -4.74 |
| A_11_P0000028013 |  | 4.36 | A_11_P075111 | cell death-inducing DFFA-like effector c | -4.73 |
| A_11_P0000026200 | sigma non-opioid intracellular receptor 1 | 4.36 | A_11_P178433 |  | -4.73 |
| A_11_P077906 | retinoic acid receptor responder (tazarotene induced) 2 | 4.36 | A_11_P204178 |  | -4.73 |
| A_11_P0000039318 | lectin, galactoside-binding, soluble, 1 | 4.35 | A_11_P214623 | chymotrypsin-like | -4.73 |
| A_11_P0000021337 |  | 4.34 | A_11_P0000025394 | monocyte to macrophage differentiation-associated | -4.72 |
| A_11_P070481 | lysyl oxidase-like 2 | 4.34 | A_11_P0000014792 |  | -4.71 |
| A_11_P168688 |  | 4.33 | A_11_P066316 |  | -4.71 |
| A_11_P0000026210 | tropomyosin 2 (beta) | 4.33 | A_11_P177098 |  | -4.70 |
| A_11_P0000017045 | stannin | 4.33 | A_11_P205453 |  | -4.69 |
| A_11_P132936 | FK506 binding protein 10, 65 kDa | 4.32 | A_11_P0000020339 | phosphomannomutase 1 | -4.67 |
| A_11_P186593 |  | 4.32 | A_11_P0000015050 |  | -4.66 |
| A_11_P0000041538 | chromosome 4 open reading frame, human C1orf198 | 4.32 | A_11_P0000028842 | chemokine (C-C motif) receptor 9 | -4.66 |
| A_11_P0000015556 |  | 4.31 | A_11_P137206 | protein tyrosine phosphatase, receptor type, R | -4.66 |
| A_11_P0000037780 |  | 4.30 | A_11_P152048 |  | -4.65 |
| A_11_P000005379 |  | 4.28 | A_11_P052536 | nephronophthisis 1 (juvenile) | -4.64 |
| A_11_P000001740 |  | 4.28 | A_11_P120996 | lymphocyte-specific protein tyrosine kinase | -4.64 |
| A_11_P138916 |  | 4.28 | A_11_P204133 | RNA binding motif protein 4 | -4.63 |
| A_11_P146998 | GPN-loop GTPase 1 | 4.28 | A_11_P152423 |  | -4.62 |
| A_11_P0000021058 | spermatogenesis associated 6 | 4.27 | A_11_P0000023096 | collagen, type XVII, alpha 1 | -4.62 |
| A_11_P0000016348 |  | 4.27 | A_11_P0000037804 | uncharacterized LOC100688518 | -4.61 |
| A_11_P00000927 | RAB34, member RAS oncogene family | 4.26 | A_11_P158448 | kinesin family member 5A | -4.61 |
| A_11_P107456 | tumor necrosis factor receptor superfamily, member 18 | 4.26 | A_11_P0000010845 |  | -4.61 |
| A_11_P150838 | complement component 1, s subcomponent | 4.26 | A_11_P0000022918 |  | -4.61 |
| A_11_P201873 | platelet/endothelial cell adhesion molecule 1 | 4.26 | A_11_P0000039810 |  | -4.60 |
| A_11_P052671 |  | 4.25 | A_11_P0000015364 |  | -4.60 |
| A_11_P0000016727 |  | 4.25 | A_11_P183778 |  | -4.60 |
| A_11_P190893 |  | 4.25 | A_11_P0000027609 | otopetrin 3 | -4.59 |
| A_11_P209343 | 5'-nucleotidase, ecto (CD73) | 4.25 | A_11_P0000020224 | MHC class I DLA-64 | -4.59 |
| A_11_P0000021930 | protein arginine methyltransferase 1 | 4.23 | A_11_P0000041917 |  | -4.59 |
| A_11_P0000038968 | transferrin | 4.23 | A_11_P071401 |  | -4.58 |
| A_11_P058031 |  | 4.21 | A_11_P218248 |  | -4.57 |
| A_11_P143073 | apolipoprotein C-I | 4.20 | A_11_P0000028170 | KIAA1244 ortholog | -4.56 |
| A_11_P052891 | selectin P (granule membrane protein 140kDa, antigen CD62) | 4.20 | A_11_P0000017073 |  | -4.56 |
| A_11_P054606 | Ig lambda chain V-I region BL2-like | 4.20 | A_11_P202163 |  | -4.55 |
| A_11_P0000023903 | histone acetyltransferase 1 | 4.19 | A_11_P000009722 |  | -4.55 |
| A_11_P109331 | transmembrane 6 superfamily member 1 | 4.18 | A_11_P200993 |  | -4.55 |
| A_11_P0000032163 |  | 4.16 | A_11_P126736 | cathepsin G | -4.55 |
| A_11_P174683 | complement component 3 | 4.16 | A_11_P053601 | ATP-binding cassette, sub-family C (CFTR/MRP), member 2 | -4.55 |
| A_11_P000001494 | collagen, type VI, alpha 1 | 4.15 | A_11_P0000041405 |  | -4.52 |
| A_11_P0000016343 | angiotensin II receptor, type 1 | 4.15 | A_11_P057061 | dehydrogenase/reductase (SDR family) member 9 | -4.52 |
| A_11_P119561 | MHC class II DR alpha chain | 4.14 | A_11_P059236 | HECT and RLD domain containing E3 ubiquitin protein ligase 5 | -4.51 |
| A_11_P115726 |  | 4.13 | A_11_P0000016883 |  | -4.51 |
| A_11_P085781 | ribonuclease, RNase A family, 4 | 4.13 | A_11_P170658 |  | -4.50 |
| A_11_P155233 |  | 4.12 | A_11_P0000041853 |  | -4.50 |
| A_11_P137026 | actin, gamma 2, smooth muscle, enteric | 4.12 | A_11_P108351 | chymotrypsin-like | -4.49 |
| A_11_P112331 |  | 4.12 | A_11_P204998 | tubulin, beta 4A class IVa | -4.49 |
| A_11_P054901 | major histocompatibility complex, class II, DQ beta 1 | 4.12 | A_11_P155253 |  | -4.49 |
| A_11_P175683 | laminin, alpha 4 | 4.11 | A_11_P054431 | CD14 molecule | -4.49 |
| A_11_P113656 | TYRO protein tyrosine kinase binding protein | 4.11 | A_11_P0000040335 |  | -4.48 |
| A_11_P113621 |  | 4.11 | A_11_P189398 |  | -4.48 |
| A_11_P203353 |  | 4.10 | A_11_P120536 | dual specificity phosphatase 6 | -4.48 |
| A_11_P190493 | platelet/endothelial cell adhesion molecule 1 | 4.10 | A_11_P099081 | SUN domain containing ossification factor | -4.48 |
| A_11_P056996 |  | 4.10 | A_11_P185073 |  | -4.47 |
| A_11_P0000028454 | C-type lectin domain family 11, member A | 4.10 | A_11_P148678 | tripartite motif containing 29 | -4.46 |
| A_11_P0000015383 |  | 4.10 | A_11_P172343 |  | -4.46 |
| A_11_P054896 | major histocompatibility complex, class II, DQ beta 1 | 4.09 | A_11_P00000997 | chymotrypsin-like | -4.45 |
| A_11_P084236 | KH domain containing, RNA binding, signal transduction associated 3 | 4.09 | A_11_P168343 |  | -4.45 |
| A_11_P0000015282 |  | 4.08 | A_11_P0000034525 | VENT homeobox | -4.44 |
| **A_11_P210523** | **interferon regulatory factor 4** | **4.08** | A_11_P0000028560 | pleckstrin homology-like domain, family B, member 3 | -4.44 |
| A_11_P0000028093 | chemokine (C-X-C motif) receptor 4 | 4.08 | A_11_P172448 |  | -4.43 |
| A_11_P0000028442 | CD33 molecule | 4.07 | A_11_P0000040275 |  | -4.43 |
| A_11_P000001019 |  | 4.06 | A_11_P193973 |  | -4.43 |
| A_11_P0000011476 |  | 4.06 | A_11_P000001622 |  | -4.42 |
| A_11_P066351 | CD163 molecule | 4.04 | A_11_P149008 |  | -4.42 |
| A_11_P00000157 |  | 4.04 | A_11_P0000030776 | N-myristoyltransferase 2 | -4.42 |
| A_11_P0000013965 |  | 4.02 | A_11_P000008612 |  | -4.42 |
| A_11_P0000023184 | stathmin-like 2 | 4.01 | A_11_P0000040319 |  | -4.40 |
| A_11_P0000027880 | tetraspanin 4 | 4.01 | A_11_P080686 | ring finger protein 213 | -4.40 |
| A_11_P050891 | flavin containing monooxygenase 1 | 4.00 | A_11_P123816 |  | -4.38 |
| A_11_P0000026898 | leucine proline-enriched proteoglycan (leprecan) 1 | 4.00 | A_11_P000005903 |  | -4.37 |
| A_11_P168523 |  | 4.00 | A_11_P097046 | transmembrane channel-like 7 | -4.36 |
| A_11_P083521 | adenylate kinase 1 | 4.00 | A_11_P0000031594 | ADP-ribosylation factor-like 14 | -4.36 |
| A_11_P0000019938 | podoplanin | 4.00 | A_11_P0000039659 |  | -4.36 |
| A_11_P0000025789 | filamin A, alpha | 3.99 | A_11_P059991 | TRAF-type zinc finger domain containing 1 | -4.35 |
| A_11_P0000027682 | solute carrier family 16 (monocarboxylate transporter), member 3 | 3.98 | A_11_P000002484 |  | -4.34 |
| A_11_P0000035500 |  | 3.98 | A_11_P195718 |  | -4.34 |
| A_11_P213338 | transglutaminase 2 | 3.97 | A_11_P087871 | CD2 molecule | -4.33 |
| A_11_P0000020898 | immunoglobulin J polypeptide, linker protein for immunoglobulin alpha and mu polypeptides | 3.97 | A_11_P058706 | poly (ADP-ribose) polymerase family, member 14 | -4.33 |
| A_11_P130296 |  | 3.96 | A_11_P000002460 |  | -4.32 |
| A_11_P0000039104 |  | 3.95 | A_11_P077911 | GTPase, IMAP family member 8 | -4.32 |
| A_11_P121481 |  | 3.95 | A_11_P192238 | solute carrier family 20 (phosphate transporter), member 1 | -4.31 |
| A_11_P072776 | secreted frizzled-related protein 4 | 3.94 | A_11_P0000019847 | retinitis pigmentosa GTPase regulator | -4.31 |
| A_11_P168558 | fibronectin 1 | 3.94 | A_11_P000007636 |  | -4.30 |
| A_11_P0000016210 | collagen, type VI, alpha 3 | 3.94 | A_11_P0000013305 |  | -4.30 |
| A_11_P0000038813 | collagen, type IV, alpha 1 | 3.94 | A_11_P0000017551 |  | -4.30 |
| A_11_P0000021447 | Lix1 homolog (mouse)-like | 3.93 | A_11_P074856 | DnaJ (Hsp40) homolog, subfamily B, member 8 | -4.30 |
| A_11_P123421 | lysozyme | 3.93 | A_11_P0000039696 |  | -4.29 |
| A_11_P121641 | neurofilament, medium polypeptide | 3.93 | A_11_P0000013530 |  | -4.29 |
| A_11_P0000014206 |  | 3.93 | A_11_P190368 |  | -4.29 |
| A_11_P00000810 | pre-mRNA processing factor 19 | 3.92 | A_11_P0000020403 |  | -4.29 |
| A_11_P0000022922 |  | 3.92 | A_11_P197743 |  | -4.29 |
| A_11_P0000039617 |  | 3.92 | A_11_P075421 | family with sequence similarity 116, member A | -4.28 |
| A_11_P145803 |  | 3.92 | A_11_P0000016607 | proprotein convertase subtilisin/kexin type 4 | -4.27 |
| A_11_P173983 |  | 3.92 | A_11_P205298 | dual specificity phosphatase 6 | -4.27 |
| A_11_P171573 | calponin 3, acidic | 3.92 | A_11_P141768 |  | -4.26 |
| A_11_P105631 | transmembrane protein 218 | 3.91 | A_11_P062501 |  | -4.26 |
| A_11_P0000025231 | SPARC related modular calcium binding 1 | 3.91 | A_11_P0000017606 |  | -4.25 |
| A_11_P0000029115 | protease, serine, 57 | 3.89 | A_11_P0000026944 |  | -4.25 |
| A_11_P0000030654 | carbonic anhydrase VIII | 3.89 | A_11_P0000015561 | CD7 molecule | -4.25 |
| A_11_P171628 | collagen, type V, alpha 2 | 3.89 | A_11_P053811 | laminin, beta 3 | -4.25 |
| A_11_P173053 |  | 3.89 | A_11_P188943 |  | -4.23 |
| A_11_P00000299 | ClpB caseinolytic peptidase B homolog (E. coli) | 3.89 | A_11_P208793 |  | -4.23 |
| A_11_P150433 | clusterin | 3.88 | A_11_P0000014896 | dual specificity phosphatase 6 | -4.23 |
| A_11_P060231 |  | 3.88 | A_11_P0000026003 | zeta-chain (TCR) associated protein kinase 70kDa | -4.22 |
| A_11_P086886 | transmembrane protein 214 | 3.88 | A_11_P120431 |  | -4.22 |
| A_11_P0000021917 |  | 3.88 | A_11_P187858 |  | -4.22 |
| A_11_P0000016946 |  | 3.88 | A_11_P0000015647 |  | -4.21 |
| A_11_P137316 | transgelin | 3.87 | A_11_P140451 |  | -4.21 |
| A_11_P0000034685 | melanoma antigen family H, 1 | 3.86 | A_11_P183738 |  | -4.21 |
| A_11_P119216 |  | 3.86 | A_11_P0000013956 |  | -4.21 |
| A_11_P0000025474 | serpin peptidase inhibitor, clade F (alpha-2 antiplasmin, pigment epithelium derived factor), member 1 | 3.85 | A_11_P0000019828 | Niemann-Pick disease, type C1 | -4.20 |
| A_11_P0000024341 |  | 3.85 | A_11_P0000025200 | chromosome 8 open reading frame, human C14orf37 | -4.20 |
| A_11_P161938 | LIM and cysteine-rich domains 1 | 3.85 | A_11_P000008377 |  | -4.20 |
| A_11_P0000015291 |  | 3.85 | A_11_P0000015118 |  | -4.20 |
| A_11_P0000032720 | microfibrillar-associated protein 4 | 3.85 | A_11_P184798 | solute carrier family 20 (phosphate transporter), member 1 | -4.20 |
| A_11_P054380 |  | 3.85 | A_11_P0000024761 |  | -4.19 |
| A_11_P115591 | plastin 3 | 3.84 | A_11_P098746 | basic leucine zipper transcription factor, ATF-like 3 | -4.19 |
| A_11_P171588 |  | 3.84 | A_11_P0000041670 |  | -4.19 |
| A_11_P0000039580 |  | 3.83 | A_11_P0000041488 |  | -4.18 |
| A_11_P121626 | L-3-hydroxyproline dehydratase (trans-) | 3.83 | A_11_P0000041347 |  | -4.18 |
| A_11_P0000020047 | microphthalmia-associated transcription factor | 3.83 | A_11_P0000038356 |  | -4.18 |
| A_11_P0000041473 | calcium/calmodulin-dependent protein kinase ID | 3.82 | A_11_P187853 |  | -4.18 |
| A_11_P184623 | moesin | 3.82 | A_11_P0000017415 |  | -4.17 |
| A_11_P117051 | connective tissue growth factor-like | 3.82 | A_11_P0000040094 |  | -4.17 |
| A_11_P195413 |  | 3.82 | A_11_P133941 |  | -4.16 |
| A_11_P0000019685 | prostaglandin E receptor 3 (subtype EP3) | 3.82 | A_11_P152398 | O-linked N-acetylglucosamine (GlcNAc) transferase | -4.16 |
| A_11_P194363 | serpin peptidase inhibitor, clade A (alpha-1 antiproteinase, antitrypsin), member 1 | 3.82 | A_11_P183633 |  | -4.16 |
| A_11_P0000030987 | UDP-galactose-4-epimerase | 3.81 | A_11_P0000023904 | integrin, alpha 6 | -4.15 |
| A_11_P054976 | clusterin | 3.81 | A_11_P0000020996 |  | -4.14 |
| A_11_P0000020877 | platelet-derived growth factor receptor, alpha polypeptide | 3.81 | A_11_P0000023702 | nuclear factor of kappa light polypeptide gene enhancer in B-cells inhibitor, zeta | -4.14 |
| A_11_P0000023599 | beta-site APP-cleaving enzyme 2 | 3.81 | A_11_P094421 | tripartite motif containing 36 | -4.14 |
| A_11_P000001029 |  | 3.80 | A_11_P000005905 |  | -4.14 |
| A_11_P0000010200 |  | 3.79 | A_11_P198983 |  | -4.14 |
| A_11_P0000020815 |  | 3.79 | A_11_P136606 |  | -4.14 |
| A_11_P118841 |  | 3.78 | A_11_P190943 |  | -4.13 |
| A_11_P087721 | sepiapterin reductase (7,8-dihydrobiopterin:NADP+ oxidoreductase) | 3.78 | A_11_P183938 |  | -4.13 |
| A_11_P101696 | plexin domain containing 2 | 3.78 | A_11_P0000032239 | mixed lineage kinase 4 | -4.13 |
| A_11_P050401 | serpin peptidase inhibitor, clade A (alpha-1 antiproteinase, antitrypsin), member 1 | 3.77 | A_11_P124226 |  | -4.12 |
| A_11_P207478 |  | 3.77 | A_11_P0000038318 |  | -4.12 |
| A_11_P0000015575 |  | 3.77 | A_11_P0000041749 |  | -4.12 |
| A_11_P191688 | interferon-related developmental regulator 2 | 3.77 | A_11_P0000032854 |  | -4.12 |
| A_11_P0000023442 |  | 3.77 | A_11_P084646 |  | -4.11 |
| A_11_P0000041934 | myelin protein zero | 3.77 | A_11_P0000036272 |  | -4.11 |
| A_11_P059381 | **endomucin** | 3.77 | A_11_P176203 |  | -4.11 |
| A_11_P0000017014 |  | 3.77 | A_11_P204903 | IQ motif containing GTPase activating protein 2 | -4.11 |
| A_11_P000005569 |  | 3.75 | A_11_P0000025926 | chromobox homolog 6 | -4.10 |
| A_11_P0000015005 | protein tyrosine phosphatase, receptor type, O | 3.75 | A_11_P0000034820 | LON peptidase N-terminal domain and ring finger 3 | -4.10 |
| A_11_P00000269 | vestigial like 4 (Drosophila) | 3.75 | A_11_P0000033677 | neuronal PAS domain protein 3 | -4.10 |
| A_11_P0000033287 | chloride channel accessory 1 | 3.75 | A_11_P198198 |  | -4.10 |
| A_11_P093201 | RAB15, member RAS onocogene family | 3.75 | A_11_P098296 | adenylate kinase 5 | -4.10 |
| A_11_P050521 | carcinoembryonic antigen-related cell adhesion molecule 30 | 3.75 | A_11_P0000041805 |  | -4.09 |
| A_11_P0000021862 | phosphoglucomutase 5 | 3.74 | A_11_P0000012153 |  | -4.09 |
| A_11_P0000026311 |  | 3.74 | A_11_P126511 |  | -4.09 |
| A_11_P078196 | secreted frizzled-related protein 1 | 3.74 | A_11_P0000036612 |  | -4.08 |
| A_11_P092961 |  | 3.74 | A_11_P201358 |  | -4.08 |
| A_11_P051281 | sulfatase 1 | 3.74 | A_11_P083426 |  | -4.07 |
| A_11_P212218 |  | 3.73 | A_11_P0000012015 |  | -4.07 |
| A_11_P071571 | collagen, type VI, alpha 5 | 3.73 | A_11_P0000039193 |  | -4.07 |
| A_11_P054956 | heat shock 22kDa protein 8 | 3.73 | A_11_P0000039704 |  | -4.06 |
| A_11_P0000019200 |  | 3.73 | A_11_P0000018147 |  | -4.06 |
| A_11_P054726 |  | 3.73 | A_11_P101550 |  | -4.06 |
| A_11_P00000250 | translocase of outer mitochondrial membrane 40 homolog (yeast) | 3.73 | A_11_P0000024169 | phosphatidylinositol 4-kinase type 2 beta | -4.06 |
| A_11_P193933 |  | 3.72 | A_11_P083496 | family with sequence similarity 102, member A | -4.06 |
| A_11_P082331 | T-box 2 | 3.72 | A_11_P066206 |  | -4.05 |
| A_11_P0000016986 | filamin A interacting protein 1-like | 3.72 | A_11_P091386 | runt-related transcription factor 2 | -4.05 |
| A_11_P0000030955 | replication protein A2, 32kDa | 3.72 | A_11_P0000041129 |  | -4.05 |
| A_11_P0000020174 | caveolin 2 | 3.71 | A_11_P216923 |  | -4.04 |
| A_11_P116226 | deoxyribonuclease I-like 1 | 3.71 | A_11_P0000010987 |  | -4.04 |
| A_11_P0000016689 |  | 3.71 | A_11_P0000026042 | lectin, galactoside-binding-like | -4.04 |
| A_11_P107076 |  | 3.71 | A_11_P160953 |  | -4.04 |
| A_11_P066376 | complement component 1, s subcomponent | 3.70 | A_11_P184328 |  | -4.04 |
| A_11_P0000026449 | opioid growth factor receptor-like 1 | 3.70 | A_11_P071396 | solute carrier family 4, sodium bicarbonate cotransporter, member 7 | -4.04 |
| A_11_P0000027228 | plasminogen activator, tissue | 3.69 | A_11_P0000040213 |  | -4.03 |
| A_11_P0000020076 | clusterin | 3.69 | A_11_P051261 | phospholipase A2, group VII (platelet-activating factor acetylhydrolase, plasma) | -4.03 |
| A_11_P190383 | complement component 1, q subcomponent binding protein | 3.69 | A_11_P136856 | ankyrin repeat domain-containing protein 26-like | -4.03 |
| A_11_P120751 | phosphoserine aminotransferase 1 | 3.69 | A_11_P000003112 |  | -4.03 |
| A_11_P186393 |  | 3.68 | A_11_P207748 |  | -4.02 |
| A_11_P000005012 | dedicator of cytokinesis 4 | 3.68 | A_11_P0000010257 |  | -4.02 |
| A_11_P063271 | cartilage acidic protein 1 | 3.68 | A_11_P190503 |  | -4.02 |
| A_11_P0000016638 |  | 3.67 | A_11_P000006558 |  | -4.02 |
| A_11_P067541 | transglutaminase 2 | 3.67 | A_11_P171673 | ring finger protein 19B | -4.01 |
| A_11_P171293 | TYRO3 protein tyrosine kinase | 3.67 | A_11_P096991 |  | -4.01 |
| A_11_P0000031048 | procollagen-lysine, 2-oxoglutarate 5-dioxygenase 1 | 3.66 | A_11_P0000015690 |  | -4.01 |
| A_11_P138336 | microfibrillar-associated protein 4 | 3.66 | A_11_P0000020861 |  | -4.00 |
| A_11_P050611 | cubilin (intrinsic factor-cobalamin receptor) | 3.66 | A_11_P0000033557 | meprin A, beta | -4.00 |
| A_11_P0000021252 | deleted in liver cancer 1 | 3.66 | A_11_P195978 |  | -3.99 |
| A_11_P0000027413 |  | 3.65 | A_11_P0000032895 | nuclear factor of activated T-cells 5, tonicity-responsive | -3.99 |
| A_11_P065961 |  | 3.65 | A_11_P193338 |  | -3.99 |
| A_11_P050696 | glucagon | 3.64 | A_11_P0000033481 | interferon stimulated exonuclease gene 20kDa-like 2 | -3.98 |
| A_11_P0000032690 | major facilitator superfamily domain containing 6-like | 3.64 | A_11_P000006225 |  | -3.98 |
| A_11_P0000025530 | adenylate kinase 1 | 3.64 | A_11_P000004108 |  | -3.98 |
| A_11_P098761 | vasohibin 2 | 3.64 | A_11_P00000499 | interferon-induced protein with tetratricopeptide repeats 1 | -3.98 |
| A_11_P051061 | glutathione peroxidase 1 | 3.64 | A_11_P00000828 |  | -3.98 |
| A_11_P109131 | family with sequence similarity 174, member B | 3.64 | A_11_P175488 |  | -3.97 |
| A_11_P0000017174 | solute carrier family 1 (glutamate/neutral amino acid transporter), member 4 | 3.64 | A_11_P210423 |  | -3.97 |
| A_11_P189848 |  | 3.64 | A_11_P0000025096 |  | -3.97 |
| A_11_P155363 | WD repeat domain, phosphoinositide interacting 1 | 3.64 | A_11_P0000019933 | ATP-binding cassette, sub-family B (MDR/TAP), member 1 | -3.97 |
| A_11_P0000033920 | dephospho-CoA kinase domain containing | 3.63 | A_11_P136496 |  | -3.96 |
| A_11_P166743 |  | 3.63 | A_11_P0000011374 |  | -3.95 |
| A_11_P0000025581 | THO complex 3 | 3.63 | A_11_P0000020308 | zinc finger, C3H1-type containing | -3.95 |
| A_11_P149363 |  | 3.63 | A_11_P0000027390 | potassium voltage-gated channel, subfamily G, member 3 | -3.95 |
| A_11_P0000017755 |  | 3.63 | A_11_P070661 |  | -3.94 |
| A_11_P0000031247 | protein BTG3-like | 3.63 | A_11_P184398 |  | -3.94 |
| A_11_P120791 | reelin | 3.63 | A_11_P0000039792 |  | -3.93 |
| A_11_P0000028452 | uncharacterized LOC484356 | 3.61 | A_11_P141713 |  | -3.93 |
| A_11_P00000209 | potassium voltage-gated channel, delayed-rectifier, subfamily S, member 3 | 3.61 | A_11_P0000026675 | KIAA1211 ortholog | -3.93 |
| A_11_P057356 | calcitonin receptor-like | 3.61 | A_11_P0000030809 | heparin-binding EGF-like growth factor | -3.92 |
| A_11_P054237 |  | 3.60 | A_11_P215508 |  | -3.92 |
| A_11_P0000026327 | major histocompatibility complex, class II, DM alpha | 3.60 | A_11_P152443 |  | -3.92 |
| A_11_P0000030175 | derlin 3 | 3.59 | A_11_P0000017120 |  | -3.92 |
| A_11_P0000019059 |  | 3.59 | A_11_P057501 | serine/threonine kinase 17b | -3.92 |
| A_11_P114646 | FtsJ RNA methyltransferase homolog 1 (E. coli) | 3.59 | A_11_P098271 | interferon-induced protein 44-like | -3.92 |
| A_11_P0000026054 |  | 3.59 | A_11_P0000018936 |  | -3.92 |
| A_11_P0000019960 | tissue factor pathway inhibitor (lipoprotein-associated coagulation inhibitor) | 3.59 | A_11_P0000040239 |  | -3.91 |
| A_11_P0000019785 | flavin containing monooxygenase 1 | 3.59 | A_11_P000004156 |  | -3.90 |
| A_11_P0000022912 | actin, alpha 2, smooth muscle, aorta | 3.58 | A_11_P000007547 |  | -3.90 |
| A_11_P0000041829 |  | 3.58 | A_11_P0000018787 |  | -3.90 |
| A_11_P00000984 | adenylate kinase 1 | 3.58 | A_11_P126356 |  | -3.90 |
| A_11_P0000026757 | interferon regulatory factor 5 | 3.58 | A_11_P0000040043 |  | -3.90 |
| A_11_P000005799 | thioesterase superfamily member 4 | 3.58 | A_11_P0000040654 |  | -3.89 |
| A_11_P000004074 |  | 3.57 | A_11_P0000015995 |  | -3.89 |
| A_11_P095261 | DnaJ (Hsp40) homolog, subfamily B, member 5 | 3.57 | A_11_P093296 | KIAA0247 ortholog | -3.89 |
| A_11_P0000040925 |  | 3.57 | A_11_P181898 |  | -3.89 |
| A_11_P188048 |  | 3.57 | A_11_P000009801 |  | -3.89 |
| A_11_P0000020541 | TEK tyrosine kinase, endothelial | 3.57 | A_11_P0000033796 | apoptosis resistant E3 ubiquitin protein ligase 1 | -3.88 |
| A_11_P062681 | progestin and adipoQ receptor family member V | 3.57 | A_11_P0000027056 | solute carrier family 5 (sodium/monocarboxylate cotransporter), member 8 | -3.88 |
| A_11_P055246 | MHC class II DLA DRB1 beta chain | 3.57 | A_11_P000004226 |  | -3.87 |
| A_11_P0000034272 | NIMA-related kinase 6 | 3.57 | A_11_P0000032416 | solute carrier family 36 (proton/amino acid symporter), member 1 | -3.87 |
| A_11_P0000020007 | heat shock 27kDa protein 1 | 3.56 | A_11_P0000014244 |  | -3.87 |
| A_11_P0000016302 |  | 3.56 | A_11_P0000024094 | abhydrolase domain containing 2 | -3.87 |
| A_11_P0000041280 | myosin IC | 3.56 | A_11_P103371 | interferon, lambda receptor 1 | -3.87 |
| A_11_P0000039510 | D-amino-acid oxidase | 3.55 | A_11_P161193 |  | -3.86 |
| A_11_P057376 | collagen, type III, alpha 1 | 3.55 | A_11_P0000014507 |  | -3.86 |
| A_11_P093391 | acyl-CoA thioesterase 6 | 3.55 | A_11_P173758 |  | -3.85 |
| A_11_P058311 | transmembrane protein 45A | 3.55 | A_11_P0000035194 |  | -3.85 |
| A_11_P075166 | LIM and cysteine-rich domains 1 | 3.55 | A_11_P050581 | endothelin 2 | -3.85 |
| A_11_P076931 | complement component 3 | 3.55 | A_11_P157703 | solute carrier family 6 (neutral amino acid transporter), member 19 | -3.84 |
| A_11_P191393 | transmembrane protein 176A | 3.55 | A_11_P0000024409 | CD3d molecule, delta (CD3-TCR complex) | -3.84 |
| A_11_P117106 | plastin 3 | 3.55 | A_11_P163433 | ubiquitin specific peptidase 2 | -3.84 |
| A_11_P118916 | melanoma cell adhesion molecule | 3.54 | A_11_P160983 |  | -3.84 |
| A_11_P0000020625 | transcription factor 19 | 3.54 | A_11_P090336 | lectin, galactoside-binding-like | -3.84 |
| A_11_P0000016604 | nephronectin | 3.54 | A_11_P200828 | adaptor-related protein complex 4, epsilon 1 subunit | -3.84 |
| A_11_P0000020301 | lysozyme | 3.53 | A_11_P0000021248 | tankyrase, TRF1-interacting ankyrin-related ADP-ribose polymerase | -3.84 |
| A_11_P107761 | retinol binding protein 7, cellular | 3.53 | A_11_P0000014262 |  | -3.84 |
| A_11_P0000034768 | Bruton agammaglobulinemia tyrosine kinase | 3.53 | A_11_P0000033666 |  | -3.83 |
| A_11_P159353 | oligonucleotide/oligosaccharide-binding fold containing 1 | 3.53 | A_11_P114366 |  | -3.83 |
| A_11_P136776 | eukaryotic translation elongation factor 2 | 3.53 | A_11_P214488 |  | -3.83 |
| A_11_P0000021957 | apolipoprotein E | 3.52 | A_11_P197408 | dual specificity phosphatase 16 | -3.82 |
| A_11_P0000019292 | dihydropyrimidinase-like 3 | 3.52 | A_11_P0000020102 | chemokine (C-C motif) ligand 3 | -3.82 |
| A_11_P078161 | plasminogen activator, tissue | 3.52 | A_11_P183073 | tripartite motif containing 36 | -3.82 |
| A_11_P0000033986 | insulin-like growth factor binding protein 4 | 3.52 | A_11_P075701 | hyaluronoglucosaminidase 1 | -3.82 |
| A_11_P058656 | ELL associated factor 2 | 3.52 | A_11_P0000016608 |  | -3.82 |
| A_11_P0000016394 | 4-hydroxy-2-oxoglutarate aldolase 1 | 3.52 | A_11_P0000028727 |  | -3.82 |
| A_11_P138621 |  | 3.52 | A_11_P098876 | laminin, gamma 2 | -3.82 |
| A_11_P139356 | homeobox B8 | 3.52 | A_11_P0000036528 | chromosome 27 open reading frame, human C12orf35 | -3.81 |
| A_11_P107636 | adherens junctions associated protein 1 | 3.52 | A_11_P050261 | NPC1 (Niemann-Pick disease, type C1, gene)-like 1 | -3.81 |
| A_11_P0000027388 |  | 3.51 | A_11_P0000040283 |  | -3.81 |
| A_11_P0000020510 | sparc/osteonectin, cwcv and kazal-like domains proteoglycan (testican) 1 | 3.51 | A_11_P209153 |  | -3.80 |
| A_11_P0000031748 | UDP-N-acetyl-alpha-D-galactosamine:polypeptide N-acetylgalactosaminyltransferase 5 (GalNAc-T5) | 3.51 | A_11_P000005705 |  | -3.80 |
| A_11_P0000020104 | chemokine (C-C motif) ligand 26 | 3.50 | A_11_P0000034284 | DAB2 interacting protein | -3.80 |
| A_11_P122696 |  | 3.50 | A_11_P169958 |  | -3.79 |
| A_11_P0000033539 | solute carrier family 14 (urea transporter), member 1 | 3.50 | A_11_P0000028612 | actinin, alpha 4 | -3.78 |
| A_11_P175118 |  | 3.50 | A_11_P148298 | regulatory factor X, 2 (influences HLA class II expression) | -3.78 |
| A_11_P0000014 | cyclin-dependent kinase 4 | 3.49 | A_11_P0000019238 | interleukin 15 | -3.78 |
| A_11_P100296 |  | 3.49 | A_11_P119016 | phospholipase A2, group XVI-like | -3.78 |
| A_11_P085806 | NDRG family member 2 | 3.49 | A_11_P0000020059 | laminin, gamma 2 | -3.78 |
| A_11_P067811 | phospholipid transfer protein | 3.49 | A_11_P0000028402 | transmembrane protein 150B | -3.77 |
| A_11_P0000032571 | melanoma cell adhesion molecule | 3.48 | A_11_P0000038402 |  | -3.77 |
| A_11_P166378 |  | 3.48 | A_11_P202553 |  | -3.77 |
| A_11_P051466 | secreted phosphoprotein 1 | 3.48 | A_11_P0000012062 |  | -3.77 |
| A_11_P095226 | sigma non-opioid intracellular receptor 1 | 3.48 | A_11_P102316 | Rho GTPase activating protein 26 | -3.77 |
| A_11_P0000020841 | KH domain containing, RNA binding, signal transduction associated 3 | 3.48 | A_11_P098691 | plexin A2 | -3.76 |
| A_11_P051116 | CD40 molecule, TNF receptor superfamily member 5 | 3.47 | A_11_P159668 | v-maf avian musculoaponeurotic fibrosarcoma oncogene homolog F | -3.76 |
| A_11_P000003656 |  | 3.47 | A_11_P193033 |  | -3.76 |
| A_11_P093756 | serpin peptidase inhibitor, clade A (alpha-1 antiproteinase, antitrypsin), member 1 | 3.47 | A_11_P0000031313 | phosphodiesterase 9A | -3.76 |
| A_11_P0000015602 | methionine sulfoxide reductase B3 | 3.47 | A_11_P079446 | deafness, autosomal dominant 5 | -3.75 |
| A_11_P0000024914 | cystathionase (cystathionine gamma-lyase) | 3.47 | A_11_P0000021920 | electron-transfer-flavoprotein, beta polypeptide | -3.75 |
| A_11_P0000023202 |  | 3.46 | A_11_P185028 | transforming, acidic coiled-coil containing protein 2 | -3.75 |
| A_11_P121116 | coagulation factor II (thrombin) receptor-like 2 | 3.46 | A_11_P117676 |  | -3.74 |
| A_11_P080311 |  | 3.46 | A_11_P0000039409 |  | -3.74 |
| A_11_P0000039909 |  | 3.45 | A_11_P0000014478 | interleukin 2 receptor, beta | -3.74 |
| A_11_P0000032449 | insulin gene enhancer protein ISL-1-like | 3.45 | A_11_P0000033290 | B-cell CLL/lymphoma 10 | -3.74 |
| A_11_P0000015954 |  | 3.45 | A_11_P217508 |  | -3.74 |
| A_11_P123411 | cysteine and glycine-rich protein 1-like | 3.44 | A_11_P173383 |  | -3.74 |
| A_11_P0000023740 | uridine monophosphate synthetase | 3.44 | A_11_P000006759 |  | -3.74 |
| A_11_P0000039621 |  | 3.43 | A_11_P0000023572 | ubiquitin specific peptidase 25 | -3.73 |
| A_11_P163913 |  | 3.43 | A_11_P0000014320 |  | -3.73 |
| A_11_P053706 | toll-like receptor 2 | 3.43 | A_11_P0000029435 | ligase IV, DNA, ATP-dependent | -3.73 |
| A_11_P0000019790 | caveolin 2 | 3.43 | A_11_P054411 |  | -3.73 |
| A_11_P061526 | beta-site APP-cleaving enzyme 2 | 3.43 | A_11_P057886 | IKAROS family zinc finger 2 (Helios) | -3.73 |
| A_11_P0000021675 | annexin A5 | 3.43 | A_11_P0000041676 |  | -3.73 |
| A_11_P0000033648 | CCAAT/enhancer binding protein (C/EBP), epsilon | 3.43 | A_11_P203268 |  | -3.73 |
| A_11_P065126 | serine palmitoyltransferase, small subunit B | 3.43 | A_11_P0000027404 | solute carrier family 20 (phosphate transporter), member 1 | -3.73 |
| A_11_P0000021654 | mitotic spindle assembly checkpoint protein MAD2A-like | 3.42 | A_11_P112491 | electron-transfer-flavoprotein, beta polypeptide | -3.72 |
| A_11_P090271 | transmembrane protein 17 | 3.42 | A_11_P0000017308 | agrin | -3.72 |
| A_11_P0000011966 |  | 3.42 | A_11_P150258 |  | -3.72 |
| A_11_P091586 | bone morphogenetic protein 5 | 3.42 | A_11_P070851 |  | -3.72 |
| A_11_P0000022001 | FXYD domain containing ion transport regulator 1 | 3.41 | A_11_P051366 | NADH dehydrogenase subunit 6 | -3.72 |
| A_11_P0000018887 | myosin, light chain 9, regulatory | 3.41 | A_11_P222535 | inositol polyphosphate-5-phosphatase F | -3.72 |
| A_11_P066301 | alpha-2-macroglobulin | 3.41 | A_11_P077326 | proprotein convertase subtilisin/kexin type 4 | -3.72 |
| A_11_P0000021424 | polyadenylate-binding protein-interacting protein 2B-like | 3.41 | A_11_P0000033402 | proteoglycan 4 | -3.71 |
| A_11_P221843 | nuclear factor I/B | 3.41 | A_11_P061886 | Bcl2 modifying factor | -3.71 |
| A_11_P0000041013 | F-box and leucine-rich repeat protein 7 | 3.40 | A_11_P0000017574 |  | -3.71 |
| A_11_P171253 | chloride intracellular channel 2 | 3.40 | A_11_P0000041393 |  | -3.71 |
| A_11_P076561 | peroxiredoxin 2 | 3.39 | A_11_P050866 | CD3e molecule, epsilon (CD3-TCR complex) | -3.71 |
| A_11_P061876 | thrombospondin 1 | 3.39 | A_11_P0000025859 | protein tyrosine phosphatase, receptor type, R | -3.70 |
| A_11_P0000015886 |  | 3.39 | A_11_P068801 | folate receptor 1 (adult) | -3.70 |
| A_11_P0000032103 | carboxypeptidase Z | 3.39 | A_11_P0000029121 |  | -3.70 |
| A_11_P0000034651 | complement factor properdin | 3.38 | A_11_P0000033294 | lysophosphatidic acid receptor 3 | -3.70 |
| A_11_P088446 | myosin, light chain 6B, alkali, smooth muscle and non-muscle | 3.38 | A_11_P151413 | bone marrow stromal cell antigen 1 | -3.70 |
| A_11_P052649 | major histocompatibility complex, class II, DQ beta 1 | 3.37 | A_11_P0000023709 | T cell receptor associated transmembrane adaptor 1 | -3.70 |
| A_11_P0000041507 | cysteine and glycine-rich protein 1-like | 3.37 | A_11_P0000039710 |  | -3.69 |
| A_11_P000001229 |  | 3.37 | A_11_P0000018279 |  | -3.69 |
| A_11_P078206 | chromosome 16 open reading frame, human C8orf4 | 3.36 | A_11_P0000040336 |  | -3.68 |
| A_11_P0000029078 | nuclear factor I/C (CCAAT-binding transcription factor) | 3.36 | A_11_P0000025943 | caspase recruitment domain family, member 10 | -3.68 |
| A_11_P100111 | zinc finger protein 521 | 3.36 | A_11_P0000039871 |  | -3.67 |
| A_11_P0000040788 | guanylate cyclase 1, soluble, beta 3 | 3.36 | A_11_P0000019657 |  | -3.67 |
| A_11_P0000040623 | retinoic acid receptor responder (tazarotene induced) 1 | 3.36 | A_11_P0000030967 | stratifin | -3.66 |
| A_11_P077926 | transmembrane protein 176B | 3.36 | A_11_P000006259 |  | -3.66 |
| A_11_P0000023641 |  | 3.36 | A_11_P0000021452 | pleckstrin homology domain containing, family O member 1 | -3.66 |
| A_11_P063781 | regulator of G-protein signaling 10 | 3.36 | A_11_P186323 | integrin, alpha 6 | -3.65 |
| A_11_P0000027580 | reelin | 3.36 | A_11_P080306 | G protein-coupled receptor 18 | -3.65 |
| A_11_P196068 |  | 3.36 | A_11_P165843 | transmembrane protein 38B | -3.64 |
| A_11_P055571 | spermatogenesis associated 17 | 3.35 | A_11_P190258 |  | -3.64 |
| A_11_P162768 | D-amino-acid oxidase | 3.35 | A_11_P184788 |  | -3.64 |
| A_11_P119896 | FLYWCH family member 2 | 3.35 | A_11_P174978 | 1-acylglycerol-3-phosphate O-acyltransferase 9 | -3.63 |
| A_11_P167743 | CD248 molecule, endosialin | 3.35 | A_11_P063716 | pancreatic lipase-related protein 2 | -3.63 |
| A_11_P062926 | WDFY family member 4 | 3.35 | A_11_P136506 |  | -3.63 |
| A_11_P0000022141 | leucine zipper transcription factor-like 1 | 3.34 | A_11_P0000027739 |  | -3.62 |
| A_11_P108026 | centromere protein N | 3.34 | A_11_P155103 | ring finger protein 128, E3 ubiquitin protein ligase | -3.62 |
| A_11_P000003275 | kelch-like family member 5 | 3.34 | A_11_P074686 |  | -3.62 |
| A_11_P151018 |  | 3.34 | A_11_P196413 |  | -3.62 |
| A_11_P051056 | egf-like module containing, mucin-like, hormone receptor-like 3 | 3.34 | A_11_P000008090 |  | -3.62 |
| A_11_P121731 | apolipoprotein L, 5 | 3.34 | A_11_P193913 |  | -3.61 |
| A_11_P0000024942 | SET and MYND domain containing 2 | 3.34 | A_11_P200418 |  | -3.61 |
| A_11_P166458 | RNA-binding protein Nova-1-like | 3.33 | A_11_P200883 |  | -3.61 |
| A_11_P0000088 | nuclear factor I/B | 3.33 | A_11_P217988 |  | -3.60 |
| A_11_P0000016445 | protein kinase C and casein kinase substrate in neurons 1 | 3.33 | A_11_P000004510 |  | -3.60 |
| A_11_P0000022878 |  | 3.33 | A_11_P00000948 | sterol carrier protein 2 | -3.60 |
| A_11_P0000035706 |  | 3.33 | A_11_P195798 |  | -3.60 |
| A_11_P0000032259 | family with sequence similarity 13, member C | 3.33 | A_11_P205843 | tetratricopeptide repeat, ankyrin repeat and coiled-coil containing 1 | -3.60 |
| A_11_P081461 |  | 3.33 | A_11_P065457 | keratin 5 | -3.59 |
| A_11_P0000031507 | 3-hydroxybutyrate dehydrogenase, type 1 | 3.33 | A_11_P087061 | calpain 13 | -3.59 |
| A_11_P0000025028 | chaperonin containing TCP1, subunit 3 (gamma) | 3.32 | A_11_P0000024210 | IQ motif containing GTPase activating protein 2 | -3.59 |
| A_11_P180518 |  | 3.32 | A_11_P179448 |  | -3.59 |
| A_11_P000002202 | chemokine (C-X-C motif) ligand 12 | 3.32 | A_11_P0000026505 | PR domain containing 1, with ZNF domain | -3.59 |
| A_11_P080546 |  | 3.32 | A_11_P139711 |  | -3.58 |
| A_11_P0000040439 | protein disulfide isomerase family A, member 6 | 3.31 | A_11_P133086 |  | -3.58 |
| A_11_P0000013747 | WWC family member 3 | 3.31 | A_11_P0000040313 |  | -3.57 |
| A_11_P0000013279 |  | 3.31 | A_11_P0000033870 | exocyst complex component 3-like 4 | -3.56 |
| A_11_P118441 | malic enzyme 1, NADP(+)-dependent, cytosolic | 3.31 | A_11_P0000039967 | lysine (K)-specific demethylase 4C | -3.56 |
| A_11_P0000037506 |  | 3.30 | A_11_P172538 | BTB (POZ) domain containing 11 | -3.56 |
| A_11_P098996 |  | 3.30 | A_11_P0000032305 | phospholipase A2, group XIIB | -3.56 |
| A_11_P0000024894 | cysteine-rich, angiogenic inducer, 61 | 3.30 | A_11_P0000030527 | tolloid-like 2 | -3.55 |
| A_11_P000005676 | sideroflexin 2 | 3.30 | A_11_P167278 |  | -3.55 |
| A_11_P053541 | bactericidal/permeability-increasing protein | 3.30 | A_11_P170388 |  | -3.55 |
| A_11_P097151 |  | 3.30 | A_11_P000004482 |  | -3.55 |
| A_11_P0000019048 |  | 3.30 | A_11_P0000041604 |  | -3.55 |
| A_11_P0000038930 |  | 3.30 | A_11_P0000010396 |  | -3.55 |
| A_11_P0000038911 |  | 3.29 | A_11_P0000033560 | desmoglein 2 | -3.55 |
| A_11_P0000021203 | SWI/SNF related, matrix associated, actin dependent regulator of chromatin, subfamily d, member 3 | 3.29 | A_11_P0000019738 | troponin T type 2 (cardiac) | -3.55 |
| A_11_P092226 | germinal center-associated, signaling and motility-like | 3.29 | A_11_P189368 |  | -3.54 |
| A_11_P0000032341 | drebrin 1 | 3.28 | A_11_P174248 | RPTOR independent companion of MTOR, complex 2 | -3.54 |
| A_11_P074966 | FYVE, RhoGEF and PH domain containing 5 | 3.28 | A_11_P0000030862 | phosphoinositide-3-kinase, regulatory subunit 1 (alpha) | -3.54 |
| A_11_P0000018028 |  | 3.28 | A_11_P127231 |  | -3.54 |
| A_11_P0000038858 | heat shock 22kDa protein 8 | 3.27 | A_11_P000002462 |  | -3.54 |
| A_11_P0000037919 | cytochrome b-245, beta polypeptide (chronic granulomatous disease) | 3.27 | A_11_P0000024022 | Mdm4 p53 binding protein homolog (mouse) | -3.54 |
| A_11_P160738 | fatty acid desaturase 3 | 3.27 | A_11_P063666 | transcription factor 7-like 2 (T-cell specific, HMG-box) | -3.54 |
| A_11_P150178 | MAP/microtubule affinity-regulating kinase 1 | 3.27 | A_11_P091686 | small ArfGAP 1 | -3.54 |
| A_11_P050046 |  | 3.27 | A_11_P0000031879 | cytochrome P450, family 27, subfamily A, polypeptide 1 | -3.53 |
| A_11_P0000023367 | eukaryotic translation initiation factor 3, subunit I | 3.27 | A_11_P186588 | laminin, beta 1 | -3.53 |
| A_11_P0000015897 | peptidylprolyl isomerase F | 3.26 | A_11_P0000016422 |  | -3.53 |
| A_11_P0000034923 | pregnancy up-regulated non-ubiquitously expressed CaM kinase | 3.26 | A_11_P055486 |  | -3.53 |
| A_11_P0000015821 |  | 3.26 | A_11_P186453 | mitogen-activated protein kinase 6 | -3.53 |
| A_11_P166083 |  | 3.25 | A_11_P0000011064 |  | -3.53 |
| A_11_P088621 | methyltransferase like 1 | 3.25 | A_11_P0000011515 |  | -3.53 |
| A_11_P0000029420 | UDP-glucose glycoprotein glucosyltransferase 2 | 3.25 | A_11_P113206 | pleckstrin homology-like domain, family B, member 3 | -3.53 |
| A_11_P064701 | adenylate cyclase 2 (brain) | 3.25 | A_11_P160033 |  | -3.52 |
| A_11_P00000281 | EBNA1 binding protein 2 | 3.25 | A_11_P174043 | troponin T type 2 (cardiac) | -3.52 |
| A_11_P0000022691 | arachidonate 5-lipoxygenase-activating protein | 3.25 | A_11_P136061 |  | -3.52 |
| A_11_P133951 | four and a half LIM domains 1 | 3.24 | A_11_P108981 | myotubularin related protein 10 | -3.52 |
| A_11_P140346 |  | 3.24 | A_11_P189813 |  | -3.52 |
| A_11_P082791 | nucleoredoxin | 3.24 | A_11_P0000021919 |  | -3.52 |
| A_11_P0000026485 | LYR motif containing 2 | 3.24 | A_11_P0000019480 |  | -3.52 |
| A_11_P149558 | chloride intracellular channel 2 | 3.24 | A_11_P085286 | glutamic pyruvate transaminase (alanine aminotransferase) 2 | -3.51 |
| A_11_P169978 |  | 3.24 | A_11_P072201 | KIAA1109 ortholog | -3.51 |
| A_11_P0000032509 | ST3 beta-galactoside alpha-2,3-sialyltransferase 4 | 3.24 | A_11_P0000028812 | sema domain, immunoglobulin domain (Ig), short basic domain, secreted, (semaphorin) 3B | -3.51 |
| A_11_P080601 | pyrroline-5-carboxylate reductase 1 | 3.24 | A_11_P090541 | ectonucleotide pyrophosphatase/phosphodiesterase 3 | -3.51 |
| A_11_P0000021335 | glucokinase (hexokinase 4) regulator | 3.24 | A_11_P102656 | FCH domain only 2 | -3.51 |
| A_11_P070466 | ADAM-like, decysin 1 | 3.23 | A_11_P0000040041 |  | -3.51 |
| A_11_P0000030339 | adhesion molecule with Ig-like domain 2 | 3.23 | A_11_P214858 |  | -3.50 |
| A_11_P0000014276 | guanine nucleotide binding protein (G protein), gamma 11 | 3.23 | A_11_P00000541 |  | -3.50 |
| A_11_P0000019852 | prostaglandin D2 synthase 21kDa (brain) | 3.22 | A_11_P052194 |  | -3.50 |
| A_11_P0000025314 | mast cell immunoglobulin-like receptor 1 | 3.22 | A_11_P0000030418 | serine/threonine/tyrosine kinase 1 | -3.49 |
| A_11_P156773 | protease, serine, 23 | 3.21 | A_11_P097056 | SMG1 phosphatidylinositol 3-kinase-related kinase | -3.49 |
| A_11_P0000015652 | MpV17 mitochondrial inner membrane protein | 3.21 | A_11_P074561 | phospholipase A2, group XVI-like | -3.49 |
| A_11_P000005840 | actin filament associated protein 1-like 1 | 3.21 | A_11_P077816 | solute carrier family 23 member 2-like | -3.49 |
| A_11_P171643 | transmembrane protein 198-like | 3.21 | A_11_P165318 |  | -3.48 |
| A_11_P000009080 |  | 3.21 | A_11_P084416 | kinesin family member C2 | -3.48 |
| A_11_P207763 |  | 3.21 | A_11_P136596 |  | -3.48 |
| A_11_P202673 |  | 3.21 | A_11_P0000036702 |  | -3.48 |
| A_11_P105931 | pleckstrin homology-like domain, family B, member 1 | 3.20 | A_11_P00000740 | endothelin converting enzyme 1 | -3.48 |
| A_11_P078496 | family with sequence similarity 149, member A | 3.20 | A_11_P115306 | zinc finger, matrin-type 1 | -3.47 |
| A_11_P0000024578 | retinol binding protein 7, cellular | 3.20 | A_11_P124811 | transmembrane protein 38B | -3.47 |
| A_11_P0000025188 | nidogen 2 (osteonidogen) | 3.20 | A_11_P0000014514 |  | -3.47 |
| A_11_P195723 |  | 3.20 | A_11_P0000039968 |  | -3.47 |
| A_11_P198258 |  | 3.19 | A_11_P214273 |  | -3.47 |
| A_11_P0000033926 | frizzled family receptor 2 | 3.19 | A_11_P211563 |  | -3.47 |
| A_11_P054646 |  | 3.19 | A_11_P156143 |  | -3.47 |
| A_11_P0000017392 |  | 3.19 | A_11_P193983 | SUN domain containing ossification factor | -3.46 |
| A_11_P0000019805 | TIMP metallopeptidase inhibitor 2 | 3.19 | A_11_P0000030413 | dual specificity phosphatase 16 | -3.46 |
| A_11_P153013 |  | 3.18 | A_11_P0000041986 |  | -3.46 |
| A_11_P219843 | phosphoglucomutase 1 | 3.18 | A_11_P181783 | flightless I homolog (Drosophila) | -3.46 |
| A_11_P116486 | KH domain containing, RNA binding, signal transduction associated 3 | 3.18 | A_11_P0000037047 |  | -3.45 |
| A_11_P110626 | uronyl-2-sulfotransferase | 3.18 | A_11_P0000039739 |  | -3.45 |
| A_11_P0000023496 | solute carrier family 27 (fatty acid transporter), member 2 | 3.18 | A_11_P167778 |  | -3.45 |
| A_11_P0000027898 | oxysterol binding protein-like 5 | 3.17 | A_11_P194963 |  | -3.45 |
| A_11_P0000026024 |  | 3.17 | A_11_P000009762 |  | -3.45 |
| A_11_P194988 | eukaryotic translation initiation factor 3, subunit H | 3.17 | A_11_P052281 | v-raf murine sarcoma viral oncogene homolog B1 | -3.45 |
| A_11_P181278 | chromogranin B (secretogranin 1) | 3.17 | A_11_P000003243 |  | -3.45 |
| A_11_P0000021123 | nucleoporin 37kDa | 3.17 | A_11_P0000033702 | kelch domain containing 1 | -3.44 |
| A_11_P0000029151 | RAB38, member RAS oncogene family | 3.17 | A_11_P110321 | coiled-coil domain containing 68 | -3.44 |
| A_11_P081191 | platelet/endothelial cell adhesion molecule 1 | 3.17 | A_11_P0000041591 | glycerophosphocholine phosphodiesterase GDE1 homolog (S. cerevisiae) | -3.44 |
| A_11_P0000041980 | zinc finger, CCHC domain containing 12 | 3.17 | A_11_P00000988 | family with sequence similarity 129, member B | -3.44 |
| A_11_P203808 | fibroblast growth factor 1 (acidic) | 3.17 | A_11_P0000025643 | glycerol kinase | -3.44 |
| A_11_P167428 |  | 3.17 | A_11_P173898 |  | -3.44 |
| A_11_P0000040170 | matrilin 2 | 3.17 | A_11_P134721 |  | -3.43 |
| A_11_P099261 |  | 3.17 | A_11_P0000027069 | doublecortin-like kinase 2 | -3.43 |
| A_11_P0000035241 |  | 3.16 | A_11_P0000031663 | neural precursor cell expressed, developmentally down-regulated 9 | -3.43 |
| A_11_P175023 | apolipoprotein L, 5 | 3.16 | A_11_P0000033347 | protein tyrosine phosphatase, receptor type, C | -3.43 |
| A_11_P162708 | IMP (inosine 5'-monophosphate) dehydrogenase 2 | 3.16 | A_11_P0000014114 |  | -3.43 |
| A_11_P0000025285 | heat shock protein 90kDa alpha (cytosolic), class A member 1 | 3.16 | A_11_P193833 | cryptochrome 2 (photolyase-like) | -3.43 |
| A_11_P0000021081 | poly (ADP-ribose) polymerase 2 | 3.16 | A_11_P174538 |  | -3.43 |
| A_11_P189818 | GLI pathogenesis-related 1 | 3.16 | A_11_P0000011418 |  | -3.42 |
| A_11_P064566 | cyclin E2 | 3.15 | A_11_P155148 |  | -3.42 |
| A_11_P098731 | transmembrane protein 206 | 3.15 | A_11_P0000019726 | beta-defensin 122 | -3.41 |
| A_11_P0000040223 | STEAP family member 2, metalloreductase | 3.15 | A_11_P137446 |  | -3.41 |
| A_11_P0000037144 | RASD family, member 2 | 3.15 | A_11_P0000019272 |  | -3.41 |
| A_11_P076736 | mitochondrial ribosomal protein L4 | 3.15 | A_11_P077921 | GTPase, IMAP family member 7 | -3.41 |
| A_11_P119826 | abl-interactor 2 | 3.14 | A_11_P0000010753 |  | -3.41 |
| A_11_P0000026085 | transmembrane emp24 domain-containing protein 9-like | 3.14 | A_11_P094926 | lysine (K)-specific demethylase 4C | -3.41 |
| A_11_P0000041965 | chibby homolog 1 (Drosophila) | 3.14 | A_11_P218078 |  | -3.41 |
| A_11_P0000039091 |  | 3.14 | A_11_P000004178 |  | -3.41 |
| A_11_P177043 |  | 3.14 | A_11_P170403 | cytochrome P450 2C21 | -3.41 |
| A_11_P131231 | DnaJ (Hsp40) homolog, subfamily A, member 3 | 3.13 | A_11_P0000025330 | myosin, light chain 4, alkali; atrial, embryonic | -3.41 |
| A_11_P0000019368 |  | 3.13 | A_11_P181593 | uncharacterized LOC607937 | -3.41 |
| A_11_P172033 |  | 3.13 | A_11_P202478 | arginine vasopressin receptor 2 | -3.41 |
| A_11_P0000010954 |  | 3.13 | A_11_P0000040197 |  | -3.41 |
| A_11_P171983 | protocadherin 17 | 3.13 | A_11_P151988 | interferon-induced protein 44-like | -3.40 |
| A_11_P219278 | FAT atypical cadherin 4 | 3.13 | A_11_P0000045 | ectonucleotide pyrophosphatase/phosphodiesterase 2 | -3.40 |
| A_11_P0000014836 |  | 3.13 | A_11_P075731 | uncharacterized LOC476620 | -3.40 |
| A_11_P205533 |  | 3.13 | A_11_P0000019829 | G protein-coupled receptor 83 | -3.40 |
| A_11_P0000015737 | transmembrane protein 109 | 3.13 | A_11_P078041 | insulin induced gene 1 | -3.40 |
| A_11_P0000031672 | guanosine monophosphate reductase | 3.13 | A_11_P0000031615 | SKI-like oncogene | -3.40 |
| A_11_P0000039041 |  | 3.13 | A_11_P084021 | ectonucleotide pyrophosphatase/phosphodiesterase 2 | -3.39 |
| A_11_P0000028469 | prostate tumor overexpressed 1 | 3.13 | A_11_P0000040068 |  | -3.39 |
| A_11_P088441 |  | 3.13 | A_11_P0000022925 | solute carrier family 4, sodium bicarbonate cotransporter, member 8 | -3.39 |
| A_11_P0000034763 | sushi-repeat containing protein, X-linked 2 | 3.12 | A_11_P052921 | desmocollin 2 | -3.39 |
| A_11_P081986 | prohibitin | 3.12 | A_11_P052786 | protein tyrosine phosphatase, receptor type, D | -3.38 |
| A_11_P0000020687 | polymerase (RNA) I polypeptide C, 30kDa | 3.12 | A_11_P0000010433 |  | -3.38 |
| A_11_P0000024808 | FLYWCH family member 2 | 3.12 | A_11_P161488 | dystonin | -3.38 |
| A_11_P050881 | aldehyde oxidase 3 | 3.12 | A_11_P00000304 | UBA domain containing 2 | -3.38 |
| A_11_P154911 | mitochondrial ribosomal protein L45 | 3.12 | A_11_P000005711 |  | -3.38 |
| A_11_P0000024112 | transmembrane 6 superfamily member 1 | 3.12 | A_11_P0000020719 | dystonin | -3.38 |
| A_11_P060151 | dynein, light chain, LC8-type 1 | 3.12 | A_11_P0000040373 |  | -3.38 |
| A_11_P0000030457 | neurotrophin 3 | 3.11 | A_11_P0000039914 |  | -3.38 |
| A_11_P0000014068 | SWAP switching B-cell complex 70kDa subunit | 3.11 | A_11_P164018 | dual specificity phosphatase 16 | -3.38 |
| A_11_P0000014283 |  | 3.11 | A_11_P000004217 |  | -3.37 |
| A_11_P0000014988 |  | 3.11 | A_11_P092906 | mitogen-activated protein kinase kinase kinase kinase 5 | -3.37 |
| A_11_P0000032647 | complement component 1, q subcomponent binding protein | 3.11 | A_11_P214988 |  | -3.37 |
| A_11_P0000040186 |  | 3.11 | A_11_P000008549 |  | -3.37 |
| A_11_P196123 | regulatory associated protein of MTOR, complex 1 | 3.10 | A_11_P130536 |  | -3.37 |
| A_11_P118471 | WAP four-disulfide core domain 2 | 3.10 | A_11_P0000037172 |  | -3.37 |
| A_11_P0000030351 | solute carrier family 2 (facilitated glucose transporter), member 13 | 3.10 | A_11_P0000040135 | family with sequence similarity 98, member C | -3.36 |
| A_11_P0000024889 | LIM domain only 4 | 3.10 | A_11_P0000021600 | protein tyrosine phosphatase, receptor type, C-associated protein | -3.36 |
| A_11_P0000039652 |  | 3.10 | A_11_P108856 | IQ motif containing GTPase activating protein 2 | -3.36 |
| A_11_P064671 | chaperonin containing TCP1, subunit 5 (epsilon) | 3.10 | A_11_P0000018816 |  | -3.36 |
| A_11_P0000024868 | calponin 3, acidic | 3.10 | A_11_P189788 | mindbomb E3 ubiquitin protein ligase 1 | -3.36 |
| A_11_P000005324 |  | 3.09 | A_11_P0000021657 | interleukin 15 | -3.36 |
| A_11_P093196 |  | 3.09 | A_11_P000009414 |  | -3.36 |
| A_11_P0000041940 |  | 3.09 | A_11_P0000034169 | ectonucleoside triphosphate diphosphohydrolase 8 | -3.36 |
| A_11_P0000020328 | tubulin tyrosine ligase-like family, member 12 | 3.09 | A_11_P0000015403 |  | -3.35 |
| A_11_P054291 |  | 3.09 | A_11_P179738 |  | -3.35 |
| A_11_P0000034020 |  | 3.09 | A_11_P184518 |  | -3.35 |
| A_11_P180838 |  | 3.09 | A_11_P0000010692 |  | -3.35 |
| A_11_P143118 |  | 3.09 | A_11_P0000025366 | ORM1-like 3 (S. cerevisiae) | -3.35 |
| A_11_P0000031812 | calcitonin receptor-like | 3.09 | A_11_P0000017054 | copine IV | -3.35 |
| A_11_P183763 |  | 3.09 | A_11_P133131 | choline/ethanolamine phosphotransferase 1 | -3.35 |
| A_11_P0000021281 | nei endonuclease VIII-like 3 (E. coli) | 3.08 | A_11_P212703 |  | -3.35 |
| A_11_P0000034532 | cytokine receptor-like factor 2 | 3.08 | A_11_P0000029757 | family with sequence similarity 65, member C | -3.35 |
| A_11_P087821 | bile acid receptor-like | 3.08 | A_11_P093961 |  | -3.34 |
| A_11_P063466 | ADP-ribosylation factor-like 3 | 3.08 | A_11_P000003414 |  | -3.34 |
| A_11_P0000014954 |  | 3.08 | A_11_P0000031544 |  | -3.33 |
| A_11_P0000033216 | hydroxyacylglutathione hydrolase-like | 3.08 | A_11_P0000034446 | platelet-derived growth factor alpha polypeptide | -3.33 |
| A_11_P0000029668 | cytochrome c oxidase subunit IV isoform 2 (lung) | 3.08 | A_11_P0000024012 |  | -3.33 |
| A_11_P123181 | selenoprotein H | 3.08 | A_11_P080161 | MYC binding protein 2, E3 ubiquitin protein ligase | -3.33 |
| A_11_P086601 | membrane bound O-acyltransferase domain containing 2 | 3.08 | A_11_P000007519 |  | -3.33 |
| A_11_P0000029629 | CDP-diacylglycerol synthase (phosphatidate cytidylyltransferase) 2 | 3.08 | A_11_P0000026914 | small ArfGAP2 | -3.32 |
| A_11_P121616 | beta-site APP-cleaving enzyme 1 | 3.07 | A_11_P077006 | regulatory factor X, 2 (influences HLA class II expression) | -3.32 |
| A_11_P0000019334 | collagen, type VI, alpha 1 | 3.07 | A_11_P0000039221 |  | -3.32 |
| A_11_P064531 |  | 3.07 | A_11_P053261 | angiotensin I converting enzyme (peptidyl-dipeptidase A) 2 | -3.32 |
| A_11_P059501 | coiled-coil domain containing 109B | 3.07 | A_11_P0000041867 |  | -3.32 |
| A_11_P192818 | annexin A1 | 3.07 | A_11_P0000011605 |  | -3.32 |
| A_11_P075131 | calcium/calmodulin-dependent protein kinase I | 3.07 | A_11_P217133 |  | -3.31 |
| A_11_P0000014881 | phosphoribosyl transferase domain containing 1 | 3.07 | A_11_P0000018427 |  | -3.31 |
| A_11_P155388 | kelch-like family member 5 | 3.07 | A_11_P000009145 |  | -3.31 |
| A_11_P052931 | thymidylate synthetase | 3.07 | A_11_P158108 |  | -3.31 |
| A_11_P107311 |  | 3.07 | A_11_P0000032220 | coagulation factor II (thrombin) receptor-like 1 | -3.31 |
| A_11_P0000027524 | repetin | 3.06 | A_11_P0000032128 | bone marrow stromal cell antigen 1 | -3.31 |
| A_11_P097076 | myosin, heavy chain 11, smooth muscle | 3.06 | A_11_P0000018035 |  | -3.31 |
| A_11_P0000039403 |  | 3.06 | A_11_P0000040355 |  | -3.31 |
| A_11_P150853 |  | 3.06 | A_11_P105191 | coiled-coil domain containing 152 | -3.31 |
| A_11_P175888 | nucleosome assembly protein 1-like 1 | 3.06 | A_11_P198638 | RPTOR independent companion of MTOR, complex 2 | -3.31 |
| A_11_P0000041824 |  | 3.06 | A_11_P0000040245 |  | -3.30 |
| A_11_P0000019995 | chemokine (C-X-C motif) receptor 7 | 3.06 | A_11_P066856 | bone morphogenetic protein 2 | -3.30 |
| A_11_P0000022558 | NOP56 ribonucleoprotein | 3.06 | A_11_P116591 |  | -3.30 |
| A_11_P058216 |  | 3.06 | A_11_P000002447 |  | -3.30 |
| A_11_P0000021743 | serpin peptidase inhibitor, clade B (ovalbumin), member 5 | 3.06 | A_11_P00000588 | coiled-coil domain containing 172 | -3.30 |
| A_11_P165488 |  | 3.05 | A_11_P132636 |  | -3.30 |
| A_11_P0000031372 | multimerin 1 | 3.05 | A_11_P054941 | ataxia telangiectasia mutated | -3.30 |
| A_11_P161333 | nephronectin | 3.05 | A_11_P0000040164 |  | -3.30 |
| A_11_P117766 | FAT atypical cadherin 4 | 3.05 | A_11_P105836 | tripartite motif containing 29 | -3.29 |
| A_11_P125046 | fibrillin 2 | 3.05 | A_11_P0000033616 | SMAD family member 7 | -3.29 |
| A_11_P132586 | phosphoribosyl transferase domain containing 1 | 3.04 | A_11_P0000031754 |  | -3.29 |
| A_11_P155663 |  | 3.04 | A_11_P0000019958 | CD52 molecule | -3.29 |
| A_11_P187303 | thymidine kinase 1, soluble | 3.04 | A_11_P186438 |  | -3.29 |
| A_11_P0000035532 | regulatory subunit of type II PKA R-subunit (RIIa) domain containing 1 | 3.04 | A_11_P0000044 | ectonucleotide pyrophosphatase/phosphodiesterase 2 | -3.29 |
| A_11_P142413 | X-box binding protein 1 | 3.04 | A_11_P0000015917 |  | -3.28 |
| A_11_P064506 | transmembrane protein 55A | 3.04 | A_11_P000003373 |  | -3.28 |
| A_11_P157193 | uncharacterized LOC475115 | 3.04 | A_11_P114556 | chromosome X open reading frame, human CXorf36 | -3.28 |
| A_11_P223123 | CDC42 effector protein (Rho GTPase binding) 3 | 3.04 | A_11_P087601 |  | -3.28 |
| A_11_P000005199 |  | 3.04 | A_11_P185528 |  | -3.28 |
| A_11_P0000040470 | myosin IC | 3.04 | A_11_P0000032569 | membrane frizzled-related protein | -3.28 |
| A_11_P102856 | iroquois homeobox 5 | 3.04 | A_11_P0000022838 | mediator complex subunit 13-like | -3.28 |
| A_11_P153133 | eukaryotic translation elongation factor 2 | 3.03 | A_11_P0000036408 |  | -3.28 |
| A_11_P062516 | KIAA0101 ortholog | 3.03 | A_11_P187333 |  | -3.28 |
| A_11_P0000033335 | neuron navigator 1 | 3.03 | A_11_P190848 |  | -3.27 |
| A_11_P0000018396 | poliovirus receptor-related 1 (herpesvirus entry mediator C) | 3.03 | A_11_P155513 | 1-acylglycerol-3-phosphate O-acyltransferase 9 | -3.27 |
| A_11_P162163 | ribosomal protein S6 kinase, 90kDa, polypeptide 2 | 3.03 | A_11_P0000017642 |  | -3.27 |
| A_11_P0000040430 |  | 3.03 | A_11_P0000021250 |  | -3.27 |
| A_11_P167523 |  | 3.03 | A_11_P091031 | chromosome 12 open reading frame, human C6orf222 | -3.27 |
| A_11_P0000021071 | ribosomal protein S8 | 3.02 | A_11_P156163 | synaptopodin 2-like | -3.27 |
| A_11_P0000026672 | kinase insert domain receptor (a type III receptor tyrosine kinase) | 3.02 | A_11_P128031 | fatty acid desaturase 2 | -3.27 |
| A_11_P131461 | flotillin 2 | 3.02 | A_11_P0000013908 |  | -3.27 |
| A_11_P178938 |  | 3.02 | A_11_P095146 | DEAD (Asp-Glu-Ala-Asp) box polypeptide 58 | -3.26 |
| A_11_P0000031272 | MIS18 kinetochore protein homolog A (S. pombe) | 3.02 | A_11_P085481 |  | -3.26 |
| A_11_P124686 | spleen tyrosine kinase | 3.02 | A_11_P0000041733 |  | -3.26 |
| A_11_P0000039290 |  | 3.02 | A_11_P178253 |  | -3.26 |
| A_11_P218553 | CD83 molecule | 3.02 | A_11_P103041 | lymphocyte-specific protein tyrosine kinase | -3.26 |
| A_11_P000001759 | X-box binding protein 1 | 3.01 | A_11_P0000014518 | UDP-glucose ceramide glucosyltransferase | -3.26 |
| A_11_P0000017020 |  | 3.01 | A_11_P0000034181 | ectonucleoside triphosphate diphosphohydrolase 2 | -3.26 |
| A_11_P0000018740 | collagen beta(1-O)galactosyltransferase 1 | 3.01 | A_11_P106516 | transmembrane 4 L six family member 5 | -3.26 |
| A_11_P0000033622 | NLR family, pyrin domain containing 3 | 3.01 | A_11_P152189 |  | -3.25 |
| A_11_P203468 |  | 3.01 | A_11_P0000016601 |  | -3.25 |
| A_11_P152313 | chromosome 26 open reading frame, human C22orf39 | 3.01 | A_11_P090021 | protein phosphatase, Mg2+/Mn2+ dependent, 1B | -3.25 |
| A_11_P0000035125 | endothelial cell surface expressed chemotaxis and apoptosis regulator | 3.01 | A_11_P0000014469 | angiopoietin-like 4 | -3.25 |
| A_11_P212098 | transmembrane protein 47 | 3.01 | A_11_P0000016706 |  | -3.25 |
| A_11_P0000040950 |  | 3.01 | A_11_P104076 | ankyrin 3, node of Ranvier (ankyrin G) | -3.24 |
| A_11_P0000041150 |  | 3.01 | A_11_P000004519 |  | -3.24 |
| A_11_P139556 |  | 3.01 | A_11_P0000012753 |  | -3.24 |
| A_11_P0000039433 | integrin, alpha M (complement component 3 receptor 3 subunit) | 3.01 | A_11_P211713 |  | -3.24 |
| A_11_P0000039303 |  | 3.01 | A_11_P174033 | ring finger protein 103 | -3.24 |
| A_11_P201553 | Down syndrome cell adhesion molecule like 1 | 3.01 | A_11_P0000034650 | synapsin I | -3.24 |
| A_11_P152998 | eukaryotic translation initiation factor 2, subunit 1 alpha, 35kDa | 3.01 | A_11_P165468 |  | -3.24 |
| A_11_P0000027576 |  | 3.00 | A_11_P128926 |  | -3.24 |
| A_11_P0000022404 | KDEL (Lys-Asp-Glu-Leu) containing 1 | 3.00 | A_11_P0000028052 | KIAA1109 ortholog | -3.23 |
| A_11_P0000021614 | EGF containing fibulin-like extracellular matrix protein 2 | 3.00 | A_11_P160298 |  | -3.23 |
| A_11_P0000024291 | annexin A8-like 1 | 3.00 | A_11_P119366 | S100 calcium binding protein A2 | -3.23 |
| A_11_P183883 | cerebral endothelial cell adhesion molecule | 3.00 | A_11_P066786 | kinesin family member 16B | -3.23 |
| A_11_P0000023662 | H2A histone family, member Z | 3.00 | A_11_P0000020590 |  | -3.23 |
| A_11_P095476 |  | 3.00 | A_11_P225563 | tripartite motif containing 29 | -3.23 |
| A_11_P168828 |  | 3.00 | A_11_P00000102 | transmembrane protein 263 | -3.23 |
| A_11_P206413 | hairy and enhancer of split 6 (Drosophila) | 3.00 | A_11_P186898 |  | -3.22 |
| A_11_P198378 |  | 3.00 | A_11_P106961 | sperm antigen with calponin homology and coiled-coil domains 1 | -3.22 |
| A_11_P210923 | crystallin, alpha B | 2.99 | A_11_P0000014509 |  | -3.22 |
| A_11_P097921 | KIAA1324 ortholog | 2.99 | A_11_P172238 |  | -3.22 |
| A_11_P0000039528 | phosphoserine phosphatase | 2.99 | A_11_P069431 | transmembrane protein 41B | -3.21 |
| A_11_P202988 | NIMA-related kinase 6 | 2.99 | A_11_P0000034159 | olfactory receptor family 1 subfamily E | -3.21 |
| A_11_P0000019867 | cubilin (intrinsic factor-cobalamin receptor) | 2.99 | A_11_P124896 | myelin associated glycoprotein | -3.21 |
| A_11_P0000030143 | POZ (BTB) and AT hook containing zinc finger 1 | 2.98 | A_11_P0000024029 |  | -3.21 |
| A_11_P105251 | Dab, mitogen-responsive phosphoprotein, homolog 2 (Drosophila) | 2.98 | A_11_P0000027089 | Rap guanine nucleotide exchange factor (GEF) 2 | -3.21 |
| A_11_P0000025950 | thiosulfate sulfurtransferase (rhodanese) | 2.98 | A_11_P216978 |  | -3.21 |
| A_11_P209683 | heat shock protein 70 | 2.98 | A_11_P125081 |  | -3.21 |
| A_11_P195633 | phospholipase A2, group IB (pancreas) | 2.98 | A_11_P000003295 |  | -3.21 |
| A_11_P0000018808 | atlastin GTPase 3 | 2.98 | A_11_P202158 |  | -3.21 |
| A_11_P080086 |  | 2.98 | A_11_P061056 | 3'-phosphoadenosine 5'-phosphosulfate synthase 2 | -3.21 |
| A_11_P0000021902 | isochorismatase domain containing 2 | 2.98 | A_11_P109446 | KIAA0232 ortholog | -3.21 |
| A_11_P0000018145 | anterior gradient 2 | 2.98 | A_11_P0000032121 | UV-stimulated scaffold protein A | -3.20 |
| A_11_P088381 |  | 2.98 | A_11_P0000032858 | gigaxonin | -3.20 |
| A_11_P00000442 | Meis homeobox 2 | 2.98 | A_11_P164078 |  | -3.20 |
| A_11_P060171 | calcium binding protein 1 | 2.97 | A_11_P0000029880 | hydroxyprostaglandin dehydrogenase 15-(NAD) | -3.20 |
| A_11_P150223 | translocase of inner mitochondrial membrane 44 homolog (yeast) | 2.97 | A_11_P0000021212 | insulin induced gene 1 | -3.20 |
| A_11_P000003843 |  | 2.97 | A_11_P181338 |  | -3.19 |
| A_11_P0000033444 | exonuclease 1 | 2.97 | A_11_P0000040686 |  | -3.19 |
| A_11_P134076 |  | 2.97 | A_11_P0000040261 |  | -3.19 |
| A_11_P0000020395 | interleukin 1 receptor, type II | 2.97 | A_11_P0000026702 | Ras association (RalGDS/AF-6) domain family member 6 | -3.19 |
| A_11_P080981 | CD300c molecule | 2.96 | A_11_P180208 | neighbor of BRCA1 gene 1 | -3.19 |
| A_11_P0000025666 | FtsJ RNA methyltransferase homolog 1 (E. coli) | 2.96 | A_11_P181378 |  | -3.19 |
| A_11_P153398 | cysteine-rich secretory protein LCCL domain containing 2 | 2.96 | A_11_P128256 | WD repeat domain 44 | -3.19 |
| A_11_P176838 | cyclin B2 | 2.96 | A_11_P085986 | dual specificity phosphatase 6 | -3.19 |
| A_11_P058181 |  | 2.96 | A_11_P061336 |  | -3.18 |
| A_11_P0000032559 | CXADR-like membrane protein | 2.96 | A_11_P0000039829 |  | -3.18 |
| A_11_P057196 | chimerin 1 | 2.96 | A_11_P194218 |  | -3.18 |
| A_11_P130911 |  | 2.96 | A_11_P0000041054 | hyaluronan and proteoglycan link protein 4 | -3.18 |
| A_11_P053796 | matrix metallopeptidase 9 (gelatinase B, 92kDa gelatinase, 92kDa type IV collagenase) | 2.96 | A_11_P205363 |  | -3.18 |
| A_11_P0000040739 |  | 2.96 | A_11_P0000027391 | AT rich interactive domain 5A (MRF1-like) | -3.18 |
| A_11_P105606 | checkpoint kinase 1 | 2.95 | A_11_P182178 |  | -3.18 |
| A_11_P193023 | protein kinase, cGMP-dependent, type I | 2.95 | A_11_P0000011594 |  | -3.18 |
| A_11_P076591 | acid phosphatase 5, tartrate resistant | 2.95 | A_11_P000003061 |  | -3.18 |
| A_11_P198778 |  | 2.94 | A_11_P118496 | monocyte to macrophage differentiation-associated | -3.17 |
| A_11_P0000027307 |  | 2.94 | A_11_P051621 | ATP-binding cassette, sub-family C (CFTR/MRP), member 5 | -3.17 |
| A_11_P154763 |  | 2.94 | A_11_P0000041598 |  | -3.17 |
| A_11_P121456 | NDRG family member 3 | 2.94 | A_11_P197093 |  | -3.17 |
| A_11_P0000022620 | phosphatidylinositol glycan anchor biosynthesis, class T | 2.94 | A_11_P0000020657 | chromosome 12 open reading frame, human C6orf106 | -3.17 |
| A_11_P0000031945 | T-cell surface glycoprotein CD1a-like | 2.94 | A_11_P0000028733 |  | -3.17 |
| A_11_P0000021437 |  | 2.94 | A_11_P0000040595 | component of oligomeric golgi complex 1 | -3.17 |
| A_11_P061036 | protein kinase, cGMP-dependent, type I | 2.94 | A_11_P000008498 |  | -3.17 |
| A_11_P0000030094 | uracil-DNA glycosylase | 2.94 | A_11_P000002392 |  | -3.17 |
| A_11_P054262 |  | 2.93 | A_11_P220473 | protein tyrosine phosphatase, receptor type, C-associated protein | -3.16 |
| A_11_P0000017101 | hepatocyte nuclear factor 1-beta-like | 2.93 | A_11_P180573 |  | -3.16 |
| A_11_P116361 | transforming growth factor, beta receptor II (70/80kDa) | 2.93 | A_11_P0000023380 |  | -3.16 |
| A_11_P114246 | peroxiredoxin 4 | 2.93 | A_11_P0000017789 |  | -3.16 |
| A_11_P191833 |  | 2.93 | A_11_P150673 |  | -3.16 |
| A_11_P066876 | chromogranin B (secretogranin 1) | 2.93 | A_11_P072491 | R3H domain containing 1 | -3.16 |
| A_11_P0000034704 | acyl-CoA wax alcohol acyltransferase 2 | 2.92 | A_11_P151273 |  | -3.16 |
| A_11_P067301 | syntrophin, alpha 1 | 2.92 | A_11_P0000039402 |  | -3.15 |
| A_11_P0000023706 | intraflagellar transport 57 homolog (Chlamydomonas) | 2.92 | A_11_P080721 | ectonucleotide pyrophosphatase/phosphodiesterase 7 | -3.15 |
| A_11_P152863 |  | 2.92 | A_11_P145888 |  | -3.15 |
| A_11_P0000037713 |  | 2.92 | A_11_P217583 | ER membrane protein complex subunit 1 | -3.15 |
| A_11_P0000016571 | peroxiredoxin 4 | 2.92 | A_11_P190883 | TGF-beta activated kinase 1/MAP3K7 binding protein 3 | -3.15 |
| A_11_P0000026424 | minichromosome maintenance complex component 3 | 2.92 | A_11_P181628 |  | -3.15 |
| A_11_P053901 | purinergic receptor P2X, ligand-gated ion channel, 4 | 2.91 | A_11_P00000475 |  | -3.15 |
| A_11_P000002471 |  | 2.91 | A_11_P148063 | cytochrome P450 2C41 | -3.15 |
| A_11_P188973 | ribosomal protein S5 | 2.91 | A_11_P0000020489 | centrosomal protein 120kDa | -3.15 |
| A_11_P146158 | decorin | 2.91 | A_11_P0000026108 | immunity-related GTPase family M protein 1-like | -3.14 |
| A_11_P0000033084 | INO80 complex subunit E | 2.91 | A_11_P000007010 |  | -3.14 |
| A_11_P0000039832 |  | 2.91 | A_11_P000005176 |  | -3.14 |
| A_11_P0000025501 | ribosomal protein L7a | 2.91 | A_11_P0000023859 |  | -3.14 |
| A_11_P213428 | guanine nucleotide binding protein (G protein), beta polypeptide 2-like 1 | 2.91 | A_11_P0000016429 | cAMP responsive element binding protein 3-like 3 | -3.14 |
| A_11_P0000031025 | microfibrillar-associated protein 2 | 2.90 | A_11_P0000039454 |  | -3.14 |
| A_11_P0000035028 | selenoprotein H | 2.90 | A_11_P000007265 |  | -3.14 |
| A_11_P120341 |  | 2.90 | A_11_P174913 |  | -3.14 |
| A_11_P0000041277 |  | 2.90 | A_11_P0000017250 |  | -3.14 |
| A_11_P204653 | prostaglandin E synthase 2 | 2.90 | A_11_P000007743 |  | -3.13 |
| A_11_P096581 | vitamin K epoxide reductase complex, subunit 1 | 2.90 | A_11_P094646 | solute carrier family 22 (organic cation/zwitterion transporter), member 4 | -3.13 |
| A_11_P0000027453 | dysferlin, limb girdle muscular dystrophy 2B (autosomal recessive) | 2.90 | A_11_P0000040215 |  | -3.13 |
| A_11_P098066 | mitochondrial ribosomal protein L45 | 2.90 | A_11_P000004213 |  | -3.13 |
| A_11_P156183 | peptidylprolyl isomerase B (cyclophilin B) | 2.90 | A_11_P180153 |  | -3.13 |
| A_11_P0000020446 | chaperonin containing TCP1, subunit 4 (delta) | 2.90 | A_11_P0000030042 | ras homolog family member F (in filopodia) | -3.13 |
| A_11_P130076 |  | 2.90 | A_11_P0000014494 |  | -3.13 |
| A_11_P058746 | integrin, beta 5 | 2.89 | A_11_P0000038691 |  | -3.13 |
| A_11_P155268 |  | 2.89 | A_11_P0000036303 |  | -3.13 |
| A_11_P177303 | MHC class II DLA DRB1 beta chain | 2.89 | A_11_P177373 |  | -3.13 |
| A_11_P0000023133 | HtrA serine peptidase 1 | 2.89 | A_11_P069561 | phosphatidylinositol-4-phosphate 3-kinase, catalytic subunit type 2 alpha | -3.13 |
| A_11_P000006300 | caldesmon 1 | 2.89 | A_11_P169818 |  | -3.13 |
| A_11_P103101 | fatty acid binding protein 3, muscle and heart (mammary-derived growth inhibitor) | 2.89 | A_11_P0000028208 | synaptotagmin-like 3 | -3.13 |
| A_11_P149613 |  | 2.89 | A_11_P192048 | acyl-CoA dehydrogenase family, member 11 | -3.13 |
| A_11_P000002691 |  | 2.89 | A_11_P079801 |  | -3.13 |
| A_11_P157223 | protein LDOC1-like | 2.89 | A_11_P0000022956 | kinesin family member 21A | -3.12 |
| A_11_P109221 | isocitrate dehydrogenase 2 (NADP+), mitochondrial | 2.89 | A_11_P168843 |  | -3.12 |
| A_11_P172353 |  | 2.88 | A_11_P0000019804 | ATP-binding cassette, sub-family C (CFTR/MRP), member 2 | -3.12 |
| A_11_P0000014995 |  | 2.88 | A_11_P0000025066 | desmocollin 2 | -3.12 |
| A_11_P0000026113 |  | 2.88 | A_11_P0000041911 |  | -3.12 |
| A_11_P0000034122 |  | 2.88 | A_11_P059301 | PDZ and LIM domain 5 | -3.12 |
| A_11_P187623 | ribosomal protein L35 | 2.88 | A_11_P000005046 |  | -3.12 |
| A_11_P148353 |  | 2.88 | A_11_P0000015178 |  | -3.12 |
| A_11_P163508 |  | 2.88 | A_11_P0000020149 | solute carrier family 5 (sodium/glucose cotransporter), member 1 | -3.11 |
| A_11_P0000031402 | ELOVL fatty acid elongase 6 | 2.88 | A_11_P059241 | HECT and RLD domain containing E3 ubiquitin protein ligase 3 | -3.11 |
| A_11_P0000026828 | glycoprotein (transmembrane) nmb | 2.88 | A_11_P209163 | tudor domain containing 7 | -3.11 |
| A_11_P0000030554 | leucine zipper, putative tumor suppressor 2 | 2.88 | A_11_P0000017367 |  | -3.11 |
| A_11_P081496 | N-acetylglucosaminidase, alpha | 2.88 | A_11_P0000015435 |  | -3.11 |
| A_11_P085361 | cyclin-dependent kinase inhibitor 2C (p18, inhibits CDK4) | 2.88 | A_11_P0000033597 | twisted gastrulation homolog 1 (Drosophila) | -3.11 |
| A_11_P0000019937 | matrix metallopeptidase 9 (gelatinase B, 92kDa gelatinase, 92kDa type IV collagenase) | 2.88 | A_11_P0000035725 |  | -3.11 |
| A_11_P0000090 | eukaryotic translation initiation factor 3, subunit D | 2.88 | A_11_P075316 | kelch repeat and BTB (POZ) domain containing 8 | -3.11 |
| A_11_P0000023877 | growth factor receptor-bound protein 14 | 2.87 | A_11_P106026 |  | -3.10 |
| A_11_P165768 | ets homologous factor | 2.87 | A_11_P117516 | glycerophosphocholine phosphodiesterase GDE1 homolog (S. cerevisiae) | -3.10 |
| A_11_P204038 |  | 2.87 | A_11_P221783 | selenoprotein P, plasma, 1 | -3.10 |
| A_11_P0000012546 |  | 2.87 | A_11_P171663 |  | -3.10 |
| A_11_P0000016537 |  | 2.87 | A_11_P0000022075 | Rho guanine nucleotide exchange factor (GEF) 3 | -3.10 |
| A_11_P110811 | t-complex 1 | 2.87 | A_11_P104331 |  | -3.10 |
| A_11_P0000030720 |  | 2.87 | A_11_P000002437 |  | -3.10 |
| A_11_P176288 | sepiapterin reductase (7,8-dihydrobiopterin:NADP+ oxidoreductase) | 2.87 | A_11_P0000041715 |  | -3.10 |
| A_11_P081046 | microtubule-associated protein tau | 2.87 | A_11_P184533 | MAX interactor 1, dimerization protein | -3.10 |
| A_11_P0000020753 | serine/arginine-rich splicing factor 12 | 2.87 | A_11_P0000038989 | v-src sarcoma (Schmidt-Ruppin A-2) viral oncogene homolog (avian) | -3.10 |
| A_11_P203318 | chromogranin B (secretogranin 1) | 2.87 | A_11_P082621 | solute carrier family 46 (folate transporter), member 1 | -3.10 |
| A_11_P157603 | tumor suppressor candidate 3 | 2.87 | A_11_P156188 |  | -3.10 |
| A_11_P000006465 |  | 2.87 | A_11_P0000021196 | GTPase, IMAP family member 2 | -3.09 |
| A_11_P0000025970 | cytoskeleton-associated protein 4 | 2.86 | A_11_P191178 | family with sequence similarity 135, member A | -3.09 |
| A_11_P137601 | nei endonuclease VIII-like 2 (E. coli) | 2.86 | A_11_P109641 | bone marrow stromal cell antigen 1 | -3.09 |
| A_11_P079241 | asparagine synthetase [glutamine-hydrolyzing]-like | 2.86 | A_11_P190513 | O-linked N-acetylglucosamine (GlcNAc) transferase | -3.09 |
| A_11_P221838 | coiled-coil-helix-coiled-coil-helix domain containing 6 | 2.86 | A_11_P0000023646 | HECT and RLD domain containing E3 ubiquitin protein ligase 3 | -3.09 |
| A_11_P178588 | isocitrate dehydrogenase 2 (NADP+), mitochondrial | 2.86 | A_11_P218993 |  | -3.09 |
| A_11_P000006067 |  | 2.86 | A_11_P0000026065 | aspartic peptidase, retroviral-like 1 | -3.09 |
| A_11_P0000023089 | ADP-ribosylation factor-like 3 | 2.85 | A_11_P0000019948 | S-antigen; retina and pineal gland (arrestin) | -3.08 |
| A_11_P0000024406 | intraflagellar transport 46 homolog (Chlamydomonas) | 2.85 | A_11_P0000027418 | retinol saturase (all-trans-retinol 13,14-reductase) | -3.08 |
| A_11_P182398 | protease-associated domain containing 1 | 2.85 | A_11_P0000028298 | guanine deaminase | -3.08 |
| A_11_P121461 |  | 2.85 | A_11_P0000010895 |  | -3.08 |
| A_11_P172533 |  | 2.85 | A_11_P050966 | ATP-binding cassette, sub-family C (CFTR/MRP), member 5 | -3.08 |
| A_11_P0000041830 | teneurin transmembrane protein 4 | 2.85 | A_11_P0000024216 | interferon-induced protein with tetratricopeptide repeats 2 | -3.08 |
| A_11_P095426 | N-acetylneuraminic acid synthase | 2.85 | A_11_P169048 |  | -3.08 |
| A_11_P104221 | H2A histone family, member Y2 | 2.85 | A_11_P155723 |  | -3.08 |
| A_11_P181273 |  | 2.85 | A_11_P052871 | laminin, beta 3 | -3.08 |
| A_11_P0000033046 | dCTP pyrophosphatase 1 | 2.85 | A_11_P071666 | protein phosphatase 2, regulatory subunit B'', alpha | -3.07 |
| A_11_P067181 | histocompatibility (minor) 13 | 2.85 | A_11_P142353 |  | -3.07 |
| A_11_P162488 |  | 2.85 | A_11_P199853 |  | -3.07 |
| A_11_P051471 | secreted phosphoprotein 1 | 2.85 | A_11_P000007361 |  | -3.07 |
| A_11_P00000436 | erythrocyte membrane protein band 4.1-like 1 | 2.84 | A_11_P0000026308 | natural cytotoxicity triggering receptor 3 | -3.07 |
| A_11_P169683 | cell adhesion molecule L1-like | 2.84 | A_11_P086351 |  | -3.07 |
| A_11_P0000020669 | MAM domain containing glycosylphosphatidylinositol anchor 1 | 2.84 | A_11_P214688 |  | -3.07 |
| A_11_P188123 | X-box binding protein 1 | 2.84 | A_11_P0000014213 | 1-acylglycerol-3-phosphate O-acyltransferase 9 | -3.07 |
| A_11_P073766 | damage-specific DNA binding protein 2, 48kDa | 2.84 | A_11_P182553 |  | -3.07 |
| A_11_P053051 | phosphodiesterase 6A, cGMP-specific, rod, alpha | 2.84 | A_11_P218733 | protein tyrosine phosphatase, receptor type, C-associated protein | -3.07 |
| A_11_P153633 | glutathione peroxidase 1 | 2.84 | A_11_P114361 | dystrophin | -3.07 |
| A_11_P0000028805 | mesencephalic astrocyte-derived neurotrophic factor | 2.84 | A_11_P199088 |  | -3.07 |
| A_11_P122076 | mitochondrial ribosomal protein L16 | 2.83 | A_11_P176968 | endonuclease/exonuclease/phosphatase family domain containing 1 | -3.07 |
| A_11_P0000022785 |  | 2.83 | A_11_P183798 |  | -3.06 |
| A_11_P055681 | retinoid X receptor, gamma | 2.83 | A_11_P0000021034 |  | -3.06 |
| A_11_P056141 | enoyl-CoA delta isomerase 2 | 2.83 | A_11_P0000010194 |  | -3.06 |
| A_11_P00000539 | family with sequence similarity 167, member B | 2.83 | A_11_P205933 |  | -3.06 |
| A_11_P155368 | GLI pathogenesis-related 1 | 2.83 | A_11_P000004928 |  | -3.06 |
| A_11_P118826 | dickkopf WNT signaling pathway inhibitor 3 | 2.83 | A_11_P213843 | family with sequence similarity 71, member D | -3.06 |
| A_11_P0000020824 | eukaryotic translation initiation factor 3, subunit H | 2.83 | A_11_P0000022939 | Rho family GTPase 1 | -3.06 |
| A_11_P078461 | N-acylsphingosine amidohydrolase (acid ceramidase) 1 | 2.83 | A_11_P052881 | ribonuclease L (2',5'-oligoisoadenylate synthetase-dependent) | -3.06 |
| A_11_P102556 | centromere protein K | 2.83 | A_11_P184563 | HERPUD family member 2 | -3.06 |
| A_11_P198848 | actin-like 6A | 2.83 | A_11_P210188 |  | -3.05 |
| A_11_P0000018648 |  | 2.83 | A_11_P193163 |  | -3.05 |
| A_11_P0000015120 | pyrroline-5-carboxylate reductase 1 | 2.83 | A_11_P0000015122 |  | -3.05 |
| A_11_P126141 |  | 2.83 | A_11_P0000035217 |  | -3.05 |
| A_11_P051481 | nuclear receptor corepressor 2 | 2.82 | A_11_P0000021739 | suppressor of cytokine signaling 6-like | -3.05 |
| A_11_P0000040818 |  | 2.82 | A_11_P0000022306 | transmembrane protein 41B | -3.05 |
| A_11_P0000021057 |  | 2.82 | A_11_P0000039585 | natural killer-tumor recognition sequence | -3.05 |
| A_11_P087216 | protein kinase domain containing, cytoplasmic | 2.82 | A_11_P132976 |  | -3.05 |
| A_11_P071871 |  | 2.82 | A_11_P053546 | serum/glucocorticoid regulated kinase 1 | -3.05 |
| A_11_P00000255 | mitochondrial ribosomal protein S7 | 2.82 | A_11_P0000030343 | twinfilin actin-binding protein 1 | -3.05 |
| A_11_P0000028477 | CD37 molecule | 2.82 | A_11_P072036 | mastermind-like 3 (Drosophila) | -3.05 |
| A_11_P0000040759 | protein tyrosine phosphatase type IVA, member 3 | 2.82 | A_11_P00000496 |  | -3.05 |
| A_11_P095231 | sigma non-opioid intracellular receptor 1 | 2.82 | A_11_P0000032806 |  | -3.05 |
| A_11_P0000025167 | mirror-image polydactyly 1 | 2.82 | A_11_P0000025521 | spectrin, alpha, non-erythrocytic 1 | -3.04 |
| A_11_P0000039415 |  | 2.82 | A_11_P058741 | kalirin, RhoGEF kinase | -3.04 |
| A_11_P126991 | cellular repressor of E1A-stimulated genes 1 | 2.82 | A_11_P090266 | UDP-GlcNAc:betaGal beta-1,3-N-acetylglucosaminyltransferase 2 | -3.04 |
| A_11_P0000020311 | GLI pathogenesis-related 1 | 2.81 | A_11_P0000019803 | transglutaminase 1 (K polypeptide epidermal type I, protein-glutamine-gamma-glutamyltransferase) | -3.04 |
| A_11_P222388 | CAP, adenylate cyclase-associated protein, 2 (yeast) | 2.81 | A_11_P083606 | zinc finger and BTB domain containing 43 | -3.04 |
| A_11_P142253 | zinc finger protein 271-like | 2.81 | A_11_P108401 | leucine rich repeat containing 36 | -3.04 |
| A_11_P174363 |  | 2.81 | A_11_P0000030767 | ADP-ribosylation factor-like 5B | -3.04 |
| A_11_P0000012914 |  | 2.81 | A_11_P154873 |  | -3.04 |
| A_11_P164083 | fibulin 2 | 2.81 | A_11_P107331 |  | -3.03 |
| A_11_P0000062 | enoyl CoA hydratase domain containing 1 | 2.81 | A_11_P0000032369 | dual specificity phosphatase 1 | -3.03 |
| A_11_P205273 | transmembrane protein 109 | 2.81 | A_11_P0000030667 | valosin containing protein (p97)/p47 complex interacting protein 1 | -3.03 |
| A_11_P0000028216 | t-complex 1 | 2.81 | A_11_P0000030952 | platelet-activating factor receptor | -3.03 |
| A_11_P153703 |  | 2.81 | A_11_P187073 |  | -3.03 |
| A_11_P173248 | FK506 binding protein 4, 59kDa | 2.81 | A_11_P062376 | zinc finger protein 280D | -3.03 |
| A_11_P0000025830 | cyclin-dependent kinase 4 | 2.80 | A_11_P0000024981 | SUN domain containing ossification factor | -3.03 |
| A_11_P0000030784 | enoyl CoA hydratase domain containing 3 | 2.80 | A_11_P0000037898 |  | -3.02 |
| A_11_P0000022319 |  | 2.80 | A_11_P190078 |  | -3.02 |
| A_11_P075356 | protein tyrosine phosphatase, receptor type, G | 2.80 | A_11_P000008247 |  | -3.02 |
| A_11_P177748 |  | 2.80 | A_11_P0000019364 |  | -3.02 |
| A_11_P207656 | guanine nucleotide binding protein (G protein), beta polypeptide 2-like 1 | 2.80 | A_11_P177608 | protein phosphatase 2, regulatory subunit A, beta | -3.02 |
| A_11_P0000030007 | phosphoglycerate mutase family member 5 | 2.80 | A_11_P173153 |  | -3.01 |
| A_11_P105621 | fasciculation and elongation protein zeta 1 (zygin I) | 2.80 | A_11_P200543 |  | -3.01 |
| A_11_P0000039325 | asporin | 2.80 | A_11_P0000023078 | dynamin binding protein | -3.01 |
| A_11_P0000016564 | syndecan 3 | 2.79 | A_11_P171993 | spermatogenesis associated, serine-rich 2-like | -3.01 |
| A_11_P0000028332 | ninjurin 1 | 2.79 | A_11_P0000017633 |  | -3.01 |
| A_11_P0000029573 | COMM domain containing 2 | 2.79 | A_11_P154498 |  | -3.01 |
| A_11_P0000025622 |  | 2.79 | A_11_P186478 |  | -3.01 |
| A_11_P137741 | nucleoporin 88kDa | 2.79 | A_11_P0000041789 |  | -3.01 |
| A_11_P0000031845 | membrane protein, palmitoylated 4 (MAGUK p55 subfamily member 4) | 2.79 | A_11_P081856 | SRC kinase signaling inhibitor 1 | -3.01 |
| A_11_P124086 | ripply transcriptional repressor 1 | 2.79 | A_11_P000004285 |  | -3.00 |
| A_11_P0000023004 | complement component 1, r subcomponent | 2.79 | A_11_P125761 | diacylglycerol O-acyltransferase 2 | -3.00 |
| A_11_P181168 |  | 2.79 | A_11_P0000034227 | phosphatidic acid phosphatase type 2 domain containing 3 | -3.00 |
| A_11_P102646 | mitochondrial ribosomal protein S27 | 2.79 | A_11_P0000033544 | phosphatidylinositol 3-kinase, catalytic subunit type 3 | -3.00 |
| A_11_P0000015671 |  | 2.78 | A_11_P068001 | SPO11 meiotic protein covalently bound to DSB | -3.00 |
| A_11_P175678 | aurora kinase A | 2.78 | A_11_P000009423 |  | -3.00 |
| A_11_P0000026535 | odd-skipped related 2 (Drosophila) | 2.78 | A_11_P184583 | ArfGAP with FG repeats 1 | -3.00 |
| A_11_P095946 |  | 2.78 | A_11_P171163 |  | -3.00 |
| A_11_P158878 |  | 2.78 | A_11_P095951 | claudin 4 | -3.00 |
| A_11_P179153 | regulator of G-protein signaling 4 | 2.78 | A_11_P0000029753 | UDP-Gal:betaGlcNAc beta 1,4- galactosyltransferase, polypeptide 5 | -3.00 |
| A_11_P093461 |  | 2.78 | A_11_P000002435 | factor interacting with PAPOLA and CPSF1 | -3.00 |
| A_11_P174318 | GIPC PDZ domain containing family, member 1 | 2.78 | A_11_P057901 |  | -3.00 |
| A_11_P148743 |  | 2.78 | A_11_P000007445 |  | -3.00 |
| A_11_P0000022219 | eukaryotic translation elongation factor 2 | 2.78 | A_11_P0000024359 | RPTOR independent companion of MTOR, complex 2 | -3.00 |
| A_11_P0000017141 | uronyl-2-sulfotransferase | 2.78 | A_11_P0000040222 |  | -3.00 |
| A_11_P0000017078 | LFNG O-fucosylpeptide 3-beta-N-acetylglucosaminyltransferase | 2.77 | A_11_P128681 | P450 (cytochrome) oxidoreductase | -3.00 |
| A_11_P061481 | proteasome assembly chaperone 1-like | 2.77 | A_11_P169713 |  | -3.00 |
| A_11_P057806 | frizzled family receptor 5 | 2.77 | A_11_P00000678 | crystallin, gamma S | -3.00 |
| A_11_P119461 |  | 2.77 | A_11_P000008146 |  | -2.99 |
| A_11_P0000027059 | glycosyltransferase 8 domain containing 2 | 2.77 | A_11_P122006 |  | -2.99 |
| A_11_P088976 | TraB domain containing | 2.77 | A_11_P0000032010 | gamma-aminobutyric acid (GABA) A receptor, gamma 3 | -2.99 |
| A_11_P165153 |  | 2.77 | A_11_P114091 | glycine receptor, alpha 2 | -2.99 |
| A_11_P103806 | spermidine synthase | 2.77 | A_11_P0000017011 |  | -2.99 |
| A_11_P190853 |  | 2.77 | A_11_P0000020130 | amyloid beta (A4) precursor protein | -2.99 |
| A_11_P217238 |  | 2.77 | A_11_P164043 | heterogeneous nuclear ribonucleoprotein A2/B1 | -2.99 |
| A_11_P000009336 |  | 2.77 | A_11_P0000032292 | sphingosine-1-phosphate lyase 1 | -2.99 |
| A_11_P115456 | phosphoribosyl pyrophosphate synthetase 1 | 2.77 | A_11_P188468 | tubulin tyrosine ligase-like family, member 4 | -2.99 |
| A_11_P050621 | non-metastatic cells 1, protein (NM23A) expressed in | 2.77 | A_11_P109911 | phosphatidylinositol 4-kinase type 2 beta | -2.99 |
| A_11_P0000025554 | mitochondrial ribosome recycling factor | 2.76 | A_11_P093796 | calmin (calponin-like, transmembrane) | -2.99 |
| A_11_P079111 | cyclin-dependent kinase 14 | 2.76 | A_11_P0000020166 | chemokine (C-X-C motif) ligand 10 | -2.99 |
| A_11_P131926 |  | 2.76 | A_11_P164673 | cytochrome P450, family 27, subfamily A, polypeptide 1 | -2.99 |
| A_11_P0000025629 | retinoblastoma binding protein 7 | 2.76 | A_11_P00000849 | O-linked N-acetylglucosamine (GlcNAc) transferase | -2.98 |
| A_11_P0000041800 |  | 2.76 | A_11_P0000013925 |  | -2.98 |
| A_11_P136766 | protein disulfide isomerase family A, member 6 | 2.76 | A_11_P199273 | anti-Mullerian hormone receptor, type II | -2.98 |
| A_11_P196078 | glycine C-acetyltransferase | 2.76 | A_11_P081726 | WAS/WASL interacting protein family, member 2 | -2.98 |
| A_11_P111131 | enoyl CoA hydratase domain containing 1 | 2.76 | A_11_P0000029083 | transducin-like enhancer of split 6 (E(sp1) homolog, Drosophila) | -2.98 |
| A_11_P0000020009 | chemokine (C-C motif) ligand 2 | 2.76 | A_11_P00000172 | HERPUD family member 2 | -2.98 |
| A_11_P082546 | transcription elongation factor, mitochondrial | 2.75 | A_11_P0000030368 | v-Ki-ras2 Kirsten rat sarcoma viral oncogene homolog | -2.98 |
| A_11_P0000040603 |  | 2.75 | A_11_P0000025594 |  | -2.98 |
| A_11_P0000031423 | syntaxin 19 | 2.75 | A_11_P059251 | family with sequence similarity 13, member A | -2.97 |
| A_11_P158593 |  | 2.75 | A_11_P212828 | bromodomain and WD repeat domain containing 1 | -2.97 |
| A_11_P054766 | CD1b molecule | 2.75 | A_11_P084356 | plectin | -2.97 |
| A_11_P064091 | mitochondrial ribosomal protein L15 | 2.75 | A_11_P220978 | mitofusin 2 | -2.97 |
| A_11_P0000020864 |  | 2.75 | A_11_P0000012359 |  | -2.96 |
| A_11_P143643 | sepiapterin reductase (7,8-dihydrobiopterin:NADP+ oxidoreductase) | 2.75 | A_11_P0000024106 | neuromedin B | -2.96 |
| A_11_P000006244 |  | 2.75 | A_11_P107271 | Myb-like, SWIRM and MPN domains 1 | -2.96 |
| A_11_P0000023030 | cat eye syndrome chromosome region, candidate 5 | 2.75 | A_11_P0000036845 |  | -2.96 |
| A_11_P130841 | coiled-coil domain containing 113 | 2.75 | A_11_P183723 |  | -2.96 |
| A_11_P0000017304 |  | 2.75 | A_11_P169203 |  | -2.96 |
| A_11_P0000031531 | lysophosphatidylcholine acyltransferase 1 | 2.75 | A_11_P173368 |  | -2.96 |
| A_11_P149998 |  | 2.75 | A_11_P150843 | delta(4)-desaturase, sphingolipid 1 | -2.96 |
| A_11_P172258 |  | 2.75 | A_11_P0000015068 |  | -2.96 |
| A_11_P000002467 |  | 2.74 | A_11_P000007624 |  | -2.96 |
| A_11_P218943 |  | 2.74 | A_11_P0000031977 |  | -2.96 |
| A_11_P0000015116 | CD276 molecule | 2.74 | A_11_P0000033002 |  | -2.96 |
| A_11_P092151 |  | 2.74 | A_11_P0000014097 |  | -2.96 |
| A_11_P0000017004 | hippocalcin-like 1 | 2.74 | A_11_P067671 | fat storage-inducing transmembrane protein 2 | -2.96 |
| A_11_P0000026678 | ADP-ribosylation factor-like 9 | 2.74 | A_11_P107221 | InaD-like (Drosophila) | -2.95 |
| A_11_P117006 | destrin (actin depolymerizing factor) | 2.74 | A_11_P095621 | lysophosphatidic acid receptor 1 | -2.95 |
| A_11_P0000018623 |  | 2.74 | A_11_P000002795 |  | -2.95 |
| A_11_P125136 | praja ring finger 1, E3 ubiquitin protein ligase | 2.74 | A_11_P123196 |  | -2.95 |
| A_11_P0000015557 |  | 2.74 | A_11_P0000034917 |  | -2.95 |
| A_11_P0000010198 |  | 2.74 | A_11_P198788 |  | -2.94 |
| A_11_P066401 | cell division cycle associated 3 | 2.74 | A_11_P0000030954 | sphingomyelin phosphodiesterase, acid-like 3B | -2.94 |
| A_11_P175503 | fatty acid desaturase 3 | 2.73 | A_11_P0000025584 | carbonyl reductase [NADPH] 1-like | -2.94 |
| A_11_P186343 |  | 2.73 | A_11_P081676 | keratin 20 | -2.94 |
| A_11_P0000015228 |  | 2.73 | A_11_P0000028194 | spectrin repeat containing, nuclear envelope 1 | -2.94 |
| A_11_P079096 | STEAP family member 2, metalloreductase | 2.73 | A_11_P101476 |  | -2.94 |
| A_11_P106456 | complement component 1, q subcomponent binding protein | 2.73 | A_11_P061951 | delta-like 4 (Drosophila) | -2.94 |
| A_11_P0000029683 | translocase of inner mitochondrial membrane 8 homolog A (yeast) | 2.73 | A_11_P0000036400 |  | -2.94 |
| A_11_P076586 |  | 2.73 | A_11_P138656 | T-box 3 | -2.94 |
| A_11_P085876 | nucleosome assembly protein 1-like 1 | 2.73 | A_11_P0000013256 |  | -2.94 |
| A_11_P0000020717 | alcohol dehydrogenase 5 (class III), chi polypeptide | 2.73 | A_11_P206183 |  | -2.94 |
| A_11_P152018 | proteasome (prosome, macropain) 26S subunit, non-ATPase, 3 | 2.73 | A_11_P0000026270 | UDP-glucose ceramide glucosyltransferase | -2.93 |
| A_11_P051016 | interleukin 13 receptor, alpha 2 | 2.73 | A_11_P0000026434 | family with sequence similarity 83, member B | -2.93 |
| A_11_P099256 | myelin protein zero-like 1 | 2.73 | A_11_P0000032326 |  | -2.93 |
| A_11_P0000017722 | caldesmon 1 | 2.73 | A_11_P191148 | ectonucleotide pyrophosphatase/phosphodiesterase 2 | -2.93 |
| A_11_P0000014080 |  | 2.73 | A_11_P0000026074 | tumor necrosis factor, alpha-induced protein 8 | -2.93 |
| A_11_P000004694 |  | 2.72 | A_11_P147853 | endothelin 2 | -2.93 |
| A_11_P055186 | heat shock protein 70 | 2.72 | A_11_P0000022837 |  | -2.93 |
| A_11_P053821 | ceruloplasmin (ferroxidase) | 2.72 | A_11_P000004484 |  | -2.92 |
| A_11_P155563 | UDP-Gal:betaGlcNAc beta 1,4- galactosyltransferase, polypeptide 2 | 2.72 | A_11_P174223 |  | -2.92 |
| A_11_P202643 | phosphatidic acid phosphatase type 2B | 2.72 | A_11_P0000031749 | cytohesin 1 interacting protein | -2.92 |
| A_11_P157393 |  | 2.72 | A_11_P000003081 |  | -2.92 |
| A_11_P0000036943 | CAP, adenylate cyclase-associated protein, 2 (yeast) | 2.72 | A_11_P212878 | E74-like factor 2 (ets domain transcription factor) | -2.92 |
| A_11_P000002949 |  | 2.72 | A_11_P00000695 | C-type lectin domain family 4, member E | -2.92 |
| A_11_P168478 |  | 2.72 | A_11_P188953 |  | -2.91 |
| A_11_P088481 | coenzyme Q10 homolog A (S. cerevisiae) | 2.72 | A_11_P000009525 |  | -2.91 |
| A_11_P0000033269 | transmembrane protein 56 | 2.71 | A_11_P0000027703 |  | -2.91 |
| A_11_P0000022365 | TSC22 domain family, member 1 | 2.71 | A_11_P000009481 |  | -2.91 |
| A_11_P051121 | **toll-like receptor 4** | 2.71 | A_11_P108436 | F-box and leucine-rich repeat protein 8 | -2.91 |
| A_11_P091766 | interleukin-1 receptor-associated kinase 1 binding protein 1 | 2.71 | A_11_P0000029057 | SH3-domain GRB2-like 1 | -2.91 |
| A_11_P0000029159 | transmembrane protein 126B | 2.71 | A_11_P0000039543 | ubiquitin-conjugating enzyme E2R 2 | -2.91 |
| A_11_P203718 | ubiquitin-conjugating enzyme E2S | 2.71 | A_11_P075886 | shisa family member 5 | -2.91 |
| A_11_P172503 | dual specificity phosphatase 7 | 2.71 | A_11_P141853 | SMG1 phosphatidylinositol 3-kinase-related kinase | -2.91 |
| A_11_P151068 | folate receptor 2 (fetal) | 2.71 | A_11_P0000032677 | lysine (K)-specific demethylase 6B | -2.91 |
| A_11_P0000027282 | storkhead box 2 | 2.71 | A_11_P171278 |  | -2.91 |
| A_11_P0000039724 |  | 2.71 | A_11_P213618 | HMG-box transcription factor 1 | -2.91 |
| A_11_P111721 | CDC28 protein kinase regulatory subunit 2 | 2.71 | A_11_P067921 |  | -2.91 |
| A_11_P202258 | ribosomal protein L34 | 2.71 | A_11_P0000010319 |  | -2.91 |
| A_11_P0000023909 | chimerin 1 | 2.71 | A_11_P215423 |  | -2.91 |
| A_11_P000008547 |  | 2.71 | A_11_P0000026410 | chloride intracellular channel 5 | -2.90 |
| A_11_P0000024120 | low density lipoprotein receptor-related protein associated protein 1 | 2.71 | A_11_P081541 | DEXH (Asp-Glu-X-His) box polypeptide 58 | -2.90 |
| A_11_P110051 | ribosome binding factor A (putative) | 2.71 | A_11_P196253 |  | -2.90 |
| A_11_P0000026864 | anillin, actin binding protein | 2.71 | A_11_P000005673 |  | -2.90 |
| A_11_P067791 | ubiquitin-conjugating enzyme E2C | 2.70 | A_11_P0000018242 |  | -2.90 |
| A_11_P0000015913 | syntaxin binding protein 6 (amisyn) | 2.70 | A_11_P0000033468 | delta(4)-desaturase, sphingolipid 1 | -2.90 |
| A_11_P0000024525 | phosphoglucomutase 1 | 2.70 | A_11_P0000039390 |  | -2.90 |
| A_11_P0000039106 | transmembrane protein 132A | 2.70 | A_11_P0000025251 |  | -2.90 |
| A_11_P0000012281 |  | 2.70 | A_11_P158338 |  | -2.90 |
| A_11_P094076 | cysteine-rich protein 2 | 2.70 | A_11_P200268 |  | -2.90 |
| A_11_P190043 |  | 2.70 | A_11_P145998 |  | -2.89 |
| A_11_P0000016191 |  | 2.70 | A_11_P0000020767 | HECT domain and ankyrin repeat containing E3 ubiquitin protein ligase 1 | -2.89 |
| A_11_P053646 |  | 2.70 | A_11_P0000014432 |  | -2.89 |
| A_11_P0000021338 | STAGA complex 65 subunit gamma-like | 2.70 | A_11_P072471 | cyclin T2 | -2.89 |
| A_11_P105091 | endothelial cell-specific molecule 1 | 2.70 | A_11_P000005788 |  | -2.89 |
| A_11_P155208 | AHA1, activator of heat shock 90kDa protein ATPase homolog 1 (yeast) | 2.70 | A_11_P0000033478 | SH2 domain containing 2A | -2.89 |
| A_11_P0000038846 |  | 2.70 | A_11_P056911 | tetratricopeptide repeat, ankyrin repeat and coiled-coil containing 1 | -2.89 |
| A_11_P184718 |  | 2.70 | A_11_P0000020105 | chemokine (C-C motif) ligand 20 | -2.89 |
| A_11_P072016 | calmegin | 2.70 | A_11_P0000010943 |  | -2.89 |
| A_11_P119566 | lipopolysaccharide-induced TNF factor | 2.70 | A_11_P184048 |  | -2.89 |
| A_11_P00000413 | mitochondrial ribosomal protein S27 | 2.69 | A_11_P170263 | suppressor of Ty 5 homolog (S. cerevisiae) | -2.89 |
| A_11_P0000020080 | RAN, member RAS oncogene family | 2.69 | A_11_P000003927 | ribosomal protein S24 | -2.89 |
| A_11_P215848 | A kinase (PRKA) anchor protein 2 | 2.69 | A_11_P104491 | family with sequence similarity 190, member B | -2.89 |
| A_11_P055701 | NUF2, NDC80 kinetochore complex component | 2.69 | A_11_P0000039561 |  | -2.89 |
| A_11_P0000032293 | pterin-4 alpha-carbinolamine dehydratase/dimerization cofactor of hepatocyte nuclear factor 1 alpha | 2.69 | A_11_P053126 | cadherin 1, type 1, E-cadherin (epithelial) | -2.89 |
| A_11_P0000027122 | protein disulfide isomerase family A, member 4 | 2.69 | A_11_P0000038902 | aminomethyltransferase | -2.89 |
| A_11_P0000020588 | protein NipSnap homolog 3A-like | 2.69 | A_11_P000008882 |  | -2.89 |
| A_11_P200708 |  | 2.69 | A_11_P069926 | neurobeachin | -2.88 |
| A_11_P079256 |  | 2.69 | A_11_P0000015363 | TSPY-like 2 | -2.88 |
| A_11_P054386 |  | 2.69 | A_11_P0000028797 | poly (ADP-ribose) polymerase family, member 3 | -2.88 |
| A_11_P119276 |  | 2.69 | A_11_P0000015170 | lipin 2 | -2.88 |
| A_11_P060456 | selenoprotein M | 2.69 | A_11_P142348 |  | -2.88 |
| A_11_P0000016821 |  | 2.69 | A_11_P193753 |  | -2.88 |
| A_11_P0000018413 |  | 2.69 | A_11_P199573 |  | -2.88 |
| A_11_P064381 |  | 2.69 | A_11_P000008378 |  | -2.88 |
| A_11_P132201 |  | 2.69 | A_11_P052821 | cytochrome P-450 3A12 | -2.88 |
| A_11_P192883 |  | 2.69 | A_11_P098571 | protein tyrosine phosphatase, receptor type, C | -2.88 |
| A_11_P070313 | PIN2/TERF1 interacting, telomerase inhibitor 1 | 2.69 | A_11_P0000027216 | extended synaptotagmin-like protein 2 | -2.88 |
| A_11_P0000029106 | polymerase (RNA) II (DNA directed) polypeptide E, 25kDa | 2.68 | A_11_P0000033971 |  | -2.88 |
| A_11_P095446 | collagen, type XV, alpha 1 | 2.68 | A_11_P188373 |  | -2.88 |
| A_11_P103841 |  | 2.68 | A_11_P070756 | S-antigen; retina and pineal gland (arrestin) | -2.88 |
| A_11_P0000032794 |  | 2.68 | A_11_P0000018779 |  | -2.87 |
| A_11_P0000032095 | GrpE-like 1, mitochondrial (E. coli) | 2.68 | A_11_P0000010975 |  | -2.87 |
| A_11_P087421 |  | 2.68 | A_11_P179423 |  | -2.87 |
| A_11_P119426 | dermatopontin | 2.68 | A_11_P0000014550 |  | -2.87 |
| A_11_P0000028747 | sulfatase modifying factor 1 | 2.68 | A_11_P209433 |  | -2.87 |
| A_11_P0000030047 | purinergic receptor P2X, ligand-gated ion channel, 4 | 2.68 | A_11_P000008161 |  | -2.87 |
| A_11_P000003922 | synaptotagmin binding, cytoplasmic RNA interacting protein | 2.68 | A_11_P0000034459 |  | -2.87 |
| A_11_P121281 |  | 2.68 | A_11_P0000021769 | serum/glucocorticoid regulated kinase 1 | -2.87 |
| A_11_P069231 | ADP-ribosylation factor interacting protein 2 | 2.67 | A_11_P181863 | cysteine-serine-rich nuclear protein 1 | -2.87 |
| A_11_P221423 | transcription elongation factor A (SII)-like 1 | 2.67 | A_11_P107421 |  | -2.87 |
| A_11_P094541 | FK506 binding protein 3, 25kDa | 2.67 | A_11_P108876 | polymerase (DNA directed) kappa | -2.87 |
| A_11_P0000024443 |  | 2.67 | A_11_P062256 | transient receptor potential cation channel, subfamily M, member 7 | -2.87 |
| A_11_P156803 |  | 2.67 | A_11_P183253 |  | -2.87 |
| A_11_P172738 | CAP, adenylate cyclase-associated protein, 2 (yeast) | 2.67 | A_11_P110251 | complexin 4 | -2.86 |
| A_11_P0000028054 | cyclin A2 | 2.67 | A_11_P0000033528 | S100 calcium binding protein A2 | -2.86 |
| A_11_P0000017687 |  | 2.67 | A_11_P176528 | serine/threonine kinase 38 like | -2.86 |
| A_11_P171853 |  | 2.67 | A_11_P118451 | v-Ki-ras2 Kirsten rat sarcoma viral oncogene homolog | -2.86 |
| A_11_P0000040688 |  | 2.67 | A_11_P137173 |  | -2.86 |
| A_11_P059981 |  | 2.67 | A_11_P139506 | sperm antigen with calponin homology and coiled-coil domains 1 | -2.86 |
| A_11_P169323 |  | 2.66 | A_11_P084276 | solute carrier family 45, member 4 | -2.86 |
| A_11_P0000028858 | nuclear receptor 2C2-associated protein | 2.66 | A_11_P104666 | cadherin-related family member 2 | -2.86 |
| A_11_P104056 | bicaudal C homolog 1 (Drosophila) | 2.66 | A_11_P000009875 |  | -2.86 |
| A_11_P0000016152 |  | 2.66 | A_11_P192748 |  | -2.86 |
| A_11_P076526 | nuclear factor I/X (CCAAT-binding transcription factor) | 2.66 | A_11_P0000034290 | cAMP responsive element binding protein 3-like 2-like | -2.86 |
| A_11_P172568 |  | 2.66 | A_11_P216228 |  | -2.86 |
| A_11_P142698 |  | 2.66 | A_11_P115991 |  | -2.86 |
| A_11_P086646 | hippocalcin-like 1 | 2.66 | A_11_P0000013005 |  | -2.86 |
| A_11_P0000014530 |  | 2.66 | A_11_P0000025824 | SH3 and cysteine rich domain 3 | -2.86 |
| A_11_P0000018698 | aquaporin 1 (Colton blood group) | 2.66 | A_11_P0000018655 |  | -2.86 |
| A_11_P0000016277 |  | 2.66 | A_11_P0000040171 |  | -2.86 |
| A_11_P0000010328 |  | 2.65 | A_11_P0000031102 | mitogen-activated protein kinase binding protein 1 | -2.86 |
| A_11_P0000033336 | cysteine and glycine-rich protein 1-like | 2.65 | A_11_P077581 | prolactin-induced protein | -2.86 |
| A_11_P063241 | ankyrin repeat domain 2 (stretch responsive muscle) | 2.65 | A_11_P087086 |  | -2.86 |
| A_11_P053006 | podoplanin | 2.65 | A_11_P179548 |  | -2.86 |
| A_11_P0000022608 | v-myb avian myeloblastosis viral oncogene homolog-like 2 | 2.65 | A_11_P0000016819 |  | -2.85 |
| A_11_P0000034095 | notchless homolog 1 (Drosophila) | 2.65 | A_11_P0000011677 |  | -2.85 |
| A_11_P109351 | SH3-domain GRB2-like 3 | 2.65 | A_11_P000009403 |  | -2.85 |
| A_11_P176388 | regulatory subunit of type II PKA R-subunit (RIIa) domain containing 1 | 2.65 | A_11_P0000020217 | melanocortin 1 receptor (alpha melanocyte stimulating hormone receptor) | -2.85 |
| A_11_P214308 | protein disulfide isomerase family A, member 6 | 2.65 | A_11_P170803 | TRAF-type zinc finger domain containing 1 | -2.85 |
| A_11_P0000040520 | adenylate cyclase 2 (brain) | 2.65 | A_11_P0000030281 |  | -2.85 |
| A_11_P077466 |  | 2.65 | A_11_P198603 | peptidyl arginine deiminase, type II | -2.85 |
| A_11_P068166 |  | 2.65 | A_11_P000003319 | chemokine (C-X-C motif) ligand 10 | -2.85 |
| A_11_P0000022972 | Ras association (RalGDS/AF-6) domain family (N-terminal) member 8 | 2.65 | A_11_P201268 |  | -2.85 |
| A_11_P0000034118 | unc-119 homolog (C. elegans) | 2.65 | A_11_P172193 | Rho GTPase activating protein 21 | -2.85 |
| A_11_P066896 | transmembrane protein 230 | 2.64 | A_11_P185738 |  | -2.85 |
| A_11_P053726 | lipoprotein lipase | 2.64 | A_11_P164308 | membrane protein, palmitoylated 6 (MAGUK p55 subfamily member 6) | -2.85 |
| A_11_P0000041504 |  | 2.64 | A_11_P209178 | karyopherin alpha 4 (importin alpha 3) | -2.85 |
| A_11_P0000025316 |  | 2.64 | A_11_P000009027 |  | -2.85 |
| A_11_P146238 | plasmalemma vesicle associated protein | 2.64 | A_11_P000008798 |  | -2.85 |
| A_11_P083746 | matrilin 2 | 2.64 | A_11_P171378 |  | -2.85 |
| A_11_P0000015406 | microtubule-associated protein 2 | 2.64 | A_11_P000008402 |  | -2.85 |
| A_11_P072361 | transmembrane protein 177 | 2.64 | A_11_P149138 | chromodomain protein, Y-like | -2.85 |
| A_11_P000009475 |  | 2.64 | A_11_P194038 |  | -2.84 |
| A_11_P085761 | APEX nuclease (multifunctional DNA repair enzyme) 1 | 2.64 | A_11_P203888 |  | -2.84 |
| A_11_P0000032125 | prominin 1 | 2.64 | A_11_P0000041955 |  | -2.84 |
| A_11_P000003548 |  | 2.64 | A_11_P165118 | Rho guanine nucleotide exchange factor (GEF) 3 | -2.84 |
| A_11_P165788 |  | 2.64 | A_11_P0000028021 | von Willebrand factor C and EGF domains | -2.84 |
| A_11_P0000027 | synuclein, alpha interacting protein | 2.64 | A_11_P065146 | sucrase-isomaltase (alpha-glucosidase) | -2.84 |
| A_11_P129126 |  | 2.64 | A_11_P0000022108 |  | -2.84 |
| A_11_P0000023461 | isovaleryl-CoA dehydrogenase | 2.64 | A_11_P086611 | ArfGAP with SH3 domain, ankyrin repeat and PH domain 2 | -2.84 |
| A_11_P0000010694 |  | 2.64 | A_11_P108266 | nuclear factor of activated T-cells 5, tonicity-responsive | -2.84 |
| A_11_P0000025138 | ER membrane protein complex subunit 9 | 2.64 | A_11_P0000040413 |  | -2.84 |
| A_11_P0000024687 | thyroid hormone receptor interactor 6 | 2.63 | A_11_P000006095 |  | -2.84 |
| A_11_P0000024768 | coenzyme Q7 homolog, ubiquinone (yeast) | 2.63 | A_11_P100416 | coiled-coil domain containing 11 | -2.83 |
| A_11_P0000029811 | tumor protein D52-like 2 | 2.63 | A_11_P000008363 |  | -2.83 |
| A_11_P0000030442 | prohibitin 2 | 2.63 | A_11_P189518 |  | -2.83 |
| A_11_P156823 | neurensin 1 | 2.63 | A_11_P0000026512 | forkhead box O3 | -2.83 |
| A_11_P203638 | mitochondrial ribosomal protein S27 | 2.63 | A_11_P0000021205 | negative regulator of ubiquitin-like proteins 1 | -2.83 |
| A_11_P060061 | RNA binding motif protein 19 | 2.63 | A_11_P0000039893 |  | -2.83 |
| A_11_P0000022995 | C-type lectin domain family 12, member A | 2.63 | A_11_P071931 | TCDD-inducible poly(ADP-ribose) polymerase | -2.83 |
| A_11_P0000032442 | endothelial cell-specific molecule 1 | 2.63 | A_11_P0000019366 |  | -2.83 |
| A_11_P171653 |  | 2.63 | A_11_P000004927 |  | -2.83 |
| A_11_P153523 |  | 2.62 | A_11_P000009306 |  | -2.83 |
| A_11_P0000040637 | androgen-induced 1 | 2.62 | A_11_P148458 | PDZ and LIM domain 5 | -2.83 |
| A_11_P058941 |  | 2.62 | A_11_P193298 | microtubule-associated protein 4 | -2.82 |
| A_11_P051996 | thymidylate synthetase | 2.62 | A_11_P0000017299 |  | -2.82 |
| A_11_P137386 | insulin-like growth factor binding protein 4 | 2.62 | A_11_P101916 | UPF2 regulator of nonsense transcripts homolog (yeast) | -2.82 |
| A_11_P169463 | DEAD (Asp-Glu-Ala-Asp) box polypeptide 54 | 2.62 | A_11_P169173 |  | -2.82 |
| A_11_P0000014922 |  | 2.62 | A_11_P0000012047 |  | -2.82 |
| A_11_P121916 | CDC42 effector protein (Rho GTPase binding) 3 | 2.62 | A_11_P109451 |  | -2.82 |
| A_11_P0000014535 | Bruton agammaglobulinemia tyrosine kinase | 2.62 | A_11_P000009567 |  | -2.82 |
| A_11_P0000016817 | RAB3A interacting protein (rabin3)-like 1 | 2.62 | A_11_P000007559 |  | -2.82 |
| A_11_P183398 | ribosomal protein L8 | 2.62 | A_11_P172678 |  | -2.82 |
| A_11_P080436 | cysteinyl-tRNA synthetase 2, mitochondrial (putative) | 2.62 | A_11_P0000017177 |  | -2.82 |
| A_11_P088261 | S100 calcium binding protein A11 | 2.62 | A_11_P0000015698 | myotubularin related protein 10 | -2.82 |
| A_11_P212303 | collagen, type VI, alpha 3 | 2.62 | A_11_P093051 |  | -2.82 |
| A_11_P00000389 | RNA binding motif protein, X-linked | 2.61 | A_11_P0000031111 | tau tubulin kinase 2 | -2.81 |
| A_11_P167348 |  | 2.61 | A_11_P0000031144 | KIAA1370 ortholog | -2.81 |
| A_11_P097815 | WD repeat domain 77 | 2.61 | A_11_P196558 | transmembrane protein 2 | -2.81 |
| A_11_P115081 |  | 2.61 | A_11_P163078 | 6-phosphofructo-2-kinase/fructose-2,6-biphosphatase 3 | -2.81 |
| A_11_P216618 |  | 2.61 | A_11_P069516 | phosphodiesterase 3B, cGMP-inhibited | -2.81 |
| A_11_P0000025328 | microtubule-associated protein tau | 2.61 | A_11_P0000022321 | related RAS viral (r-ras) oncogene homolog 2 | -2.81 |
| A_11_P058886 | 3-hydroxybutyrate dehydrogenase, type 1 | 2.61 | A_11_P198288 | ras homolog family member V | -2.81 |
| A_11_P0000021785 |  | 2.61 | A_11_P074251 | cornichon homolog 2 (Drosophila) | -2.81 |
| A_11_P164088 | zinc finger protein 496 | 2.61 | A_11_P00000441 | amyloid beta (A4) precursor protein | -2.81 |
| A_11_P0000040835 | homeobox B7 | 2.61 | A_11_P182943 | lysine (K)-specific demethylase 5B | -2.81 |
| A_11_P0000024300 | NOP16 nucleolar protein | 2.60 | A_11_P0000040133 | SIN3 transcription regulator family member B | -2.81 |
| A_11_P0000012808 |  | 2.60 | A_11_P0000023128 | WD repeat domain 11 | -2.81 |
| A_11_P0000022393 | DAZ interacting protein 1 | 2.60 | A_11_P000008427 |  | -2.81 |
| A_11_P056371 | NHL repeat containing 1 | 2.60 | A_11_P0000030226 | N-acylsphingosine amidohydrolase (non-lysosomal ceramidase) 2B | -2.81 |
| A_11_P0000021906 | HSPA (heat shock 70kDa) binding protein, cytoplasmic cochaperone 1 | 2.60 | A_11_P0000039945 |  | -2.81 |
| A_11_P0000030706 | carbonic anhydrase XIII | 2.60 | A_11_P107706 | tumor necrosis factor receptor superfamily, member 9 | -2.81 |
| A_11_P0000023285 |  | 2.60 | A_11_P140586 |  | -2.81 |
| A_11_P085691 | cell division cycle 20 | 2.60 | A_11_P0000035070 |  | -2.81 |
| A_11_P176603 |  | 2.60 | A_11_P0000014305 |  | -2.80 |
| A_11_P0000033486 | SMG5 nonsense mediated mRNA decay factor | 2.60 | A_11_P0000031158 | family with sequence similarity 63, member B | -2.80 |
| A_11_P106019 | transgelin | 2.60 | A_11_P140511 | vacuolar protein sorting 13 homolog D (S. cerevisiae) | -2.80 |
| A_11_P092401 | oxidase (cytochrome c) assembly 1-like | 2.60 | A_11_P120021 |  | -2.80 |
| A_11_P104696 | biorientation of chromosomes in cell division 1 | 2.60 | A_11_P00000992 |  | -2.80 |
| A_11_P0000014793 |  | 2.59 | A_11_P206163 |  | -2.80 |
| A_11_P118256 |  | 2.59 | A_11_P0000022687 | furry homolog (Drosophila) | -2.80 |
| A_11_P087096 | nucleophosmin (nucleolar phosphoprotein B23, numatrin) | 2.59 | A_11_P193878 |  | -2.80 |
| A_11_P0000039672 | bone morphogenetic protein 5 | 2.59 | A_11_P0000023165 | UBX domain protein 2B | -2.80 |
| A_11_P079976 | nucleophosmin (nucleolar phosphoprotein B23, numatrin) | 2.59 | A_11_P0000020535 | perilipin 2 | -2.80 |
| A_11_P202768 | target of EGR1, member 1 (nuclear) | 2.59 | A_11_P0000025797 | integrin, alpha 7 | -2.80 |
| A_11_P0000015558 | chemokine (C-X-C motif) receptor 4 | 2.59 | A_11_P0000040056 |  | -2.80 |
| A_11_P0000038947 |  | 2.59 | A_11_P062746 | promyelocytic leukemia | -2.80 |
| A_11_P152468 | protein arginine methyltransferase 5 | 2.59 | A_11_P068531 |  | -2.80 |
| A_11_P0000021966 | platelet-activating factor acetylhydrolase 1b, catalytic subunit 3 (29kDa) | 2.59 | A_11_P173158 |  | -2.80 |
| A_11_P0000016364 |  | 2.59 | A_11_P0000016557 | 3'-phosphoadenosine 5'-phosphosulfate synthase 2 | -2.80 |
| A_11_P158073 | ectonucleotide pyrophosphatase/phosphodiesterase 6 | 2.59 | A_11_P122566 | poly (ADP-ribose) polymerase family, member 12 | -2.80 |
| A_11_P098151 | LIM domain only 4 | 2.59 | A_11_P0000026259 | transmembrane protein 38B | -2.80 |
| A_11_P0000027749 |  | 2.59 | A_11_P080756 |  | -2.80 |
| A_11_P000003451 |  | 2.59 | A_11_P0000032248 | ARV1 homolog (S. cerevisiae) | -2.80 |
| A_11_P074321 |  | 2.59 | A_11_P0000018454 |  | -2.79 |
| A_11_P0000026018 | mutS homolog 2, colon cancer, nonpolyposis type 1 (E. coli) | 2.59 | A_11_P0000033754 | MYC associated factor X | -2.79 |
| A_11_P0000016896 | TBC1 domain family, member 4 | 2.59 | A_11_P110221 | zinc finger, CCHC domain containing 2 | -2.79 |
| A_11_P0000023157 | mitochondrial ribosomal protein L15 | 2.58 | A_11_P0000018524 | ras homolog family member V | -2.79 |
| A_11_P177778 | cytoskeleton-associated protein 4 | 2.58 | A_11_P173578 | family with sequence similarity 190, member B | -2.79 |
| A_11_P138396 |  | 2.58 | A_11_P0000040926 |  | -2.79 |
| A_11_P0000020188 | tachykinin receptor 2 | 2.58 | A_11_P0000025901 |  | -2.79 |
| A_11_P179608 |  | 2.58 | A_11_P093211 | MYC associated factor X | -2.79 |
| A_11_P0000021444 | ankyrin repeat domain 35 | 2.58 | A_11_P000008602 |  | -2.79 |
| A_11_P052876 | centromere protein F, 350/400kDa (mitosin) | 2.58 | A_11_P093641 | echinoderm microtubule associated protein like 5 | -2.79 |
| A_11_P185148 | complement component 1, r subcomponent | 2.58 | A_11_P0000016590 | V-set and transmembrane domain containing 2 like | -2.79 |
| A_11_P198653 |  | 2.58 | A_11_P0000040514 |  | -2.79 |
| A_11_P0000017246 |  | 2.58 | A_11_P225193 | Meis homeobox 1 | -2.79 |
| A_11_P059831 |  | 2.58 | A_11_P000002450 |  | -2.79 |
| A_11_P000002233 |  | 2.57 | A_11_P0000015133 |  | -2.79 |
| A_11_P0000021253 | tumor suppressor candidate 3 | 2.57 | A_11_P055406 | Mdm4 p53 binding protein homolog (mouse) | -2.79 |
| A_11_P169563 | atlastin GTPase 3 | 2.57 | A_11_P139196 | synaptotagmin I | -2.78 |
| A_11_P0000022116 | glutathione peroxidase 1 | 2.57 | A_11_P000008611 |  | -2.78 |
| A_11_P0000034777 | brain expressed, X-linked 4 | 2.57 | A_11_P168863 |  | -2.78 |
| A_11_P0000029974 | hairy and enhancer of split 6 (Drosophila) | 2.57 | A_11_P202683 |  | -2.78 |
| A_11_P000003642 |  | 2.57 | A_11_P207018 |  | -2.78 |
| A_11_P219718 |  | 2.57 | A_11_P161273 |  | -2.78 |
| A_11_P0000027001 | O-sialoglycoprotein endopeptidase | 2.57 | A_11_P172253 |  | -2.78 |
| A_11_P074821 | thioredoxin reductase 3 | 2.57 | A_11_P000008232 |  | -2.78 |
| A_11_P086211 | insulin-like growth factor 1 (somatomedin C) | 2.57 | A_11_P068331 | pannexin 1 | -2.78 |
| A_11_P0000039591 |  | 2.57 | A_11_P0000011381 |  | -2.78 |
| A_11_P0000019947 | biglycan | 2.57 | A_11_P203848 | SH3-domain binding protein 2 | -2.78 |
| A_11_P099191 | coiled-coil domain containing 181 | 2.57 | A_11_P136636 |  | -2.78 |
| A_11_P00000625 | protein kinase D1 | 2.57 | A_11_P217593 |  | -2.78 |
| A_11_P154123 |  | 2.57 | A_11_P209385 | glycerol kinase | -2.78 |
| A_11_P215003 | ATP citrate lyase | 2.56 | A_11_P110791 |  | -2.78 |
| A_11_P0000017195 | StAR-related lipid transfer (START) domain containing 8 | 2.56 | A_11_P0000017895 |  | -2.77 |
| A_11_P113801 | programmed cell death 2-like | 2.56 | A_11_P207353 | solute carrier family 40 (iron-regulated transporter), member 1 | -2.77 |
| A_11_P125066 |  | 2.56 | A_11_P197788 |  | -2.77 |
| A_11_P196868 | clathrin, heavy chain-like 1 | 2.56 | A_11_P134226 |  | -2.77 |
| A_11_P0000020237 | guanylate cyclase 1, soluble, beta 3 | 2.56 | A_11_P000002335 |  | -2.77 |
| A_11_P0000019109 | selenoprotein M | 2.56 | A_11_P0000040015 |  | -2.77 |
| A_11_P0000019235 |  | 2.56 | A_11_P0000025811 | signal transducer and activator of transcription 2, 113kDa | -2.77 |
| A_11_P061176 | protein BTG3-like | 2.56 | A_11_P000007941 |  | -2.77 |
| A_11_P061911 | isovaleryl-CoA dehydrogenase | 2.56 | A_11_P173123 |  | -2.77 |
| A_11_P075816 | IMP (inosine 5'-monophosphate) dehydrogenase 2 | 2.56 | A_11_P0000030071 | tescalcin | -2.77 |
| A_11_P203493 |  | 2.56 | A_11_P000007000 |  | -2.77 |
| A_11_P0000027326 |  | 2.56 | A_11_P0000041294 |  | -2.77 |
| A_11_P052656 | major histocompatibility complex, class II, DQ alpha 1 | 2.56 | A_11_P079641 | engulfment and cell motility 1 | -2.77 |
| A_11_P0000039279 | phytanoyl-CoA hydroxylase-like | 2.56 | A_11_P092276 |  | -2.76 |
| A_11_P143498 | reticulocalbin 1, EF-hand calcium binding domain | 2.56 | A_11_P0000032749 | protein kinase, AMP-activated, alpha 2 catalytic subunit | -2.76 |
| A_11_P057566 | phospholipase C-like 1 | 2.56 | A_11_P093531 | transmembrane emp24 protein transport domain containing 8 | -2.76 |
| A_11_P0000030189 | cell division cycle 45 | 2.56 | A_11_P0000017390 |  | -2.76 |
| A_11_P055026 | heat shock protein 70 | 2.56 | A_11_P207308 | family with sequence similarity 187, member B | -2.76 |
| A_11_P0000025833 |  | 2.56 | A_11_P081791 | titin-cap | -2.76 |
| A_11_P166003 |  | 2.56 | A_11_P0000017663 |  | -2.76 |
| A_11_P0000041879 |  | 2.56 | A_11_P0000030906 | adenylate cyclase 7 | -2.76 |
| A_11_P179593 | ribonuclease P/MRP 21kDa subunit | 2.56 | A_11_P0000034051 | ATP-binding cassette, sub-family C (CFTR/MRP), member 3 | -2.76 |
| A_11_P0000021556 | eukaryotic translation initiation factor 3, subunit M | 2.56 | A_11_P065221 | solute carrier family 2 (facilitated glucose transporter), member 2 | -2.76 |
| A_11_P061306 | eva-1 homolog C (C. elegans) | 2.56 | A_11_P0000022042 |  | -2.76 |
| A_11_P0000016533 |  | 2.56 | A_11_P0000014475 |  | -2.76 |
| A_11_P0000018603 |  | 2.56 | A_11_P000005395 | chromosome 8 open reading frame, human C14orf37 | -2.76 |
| A_11_P064831 |  | 2.56 | A_11_P111211 | cell division cycle 14B | -2.75 |
| A_11_P0000013691 | family with sequence similarity 210, member B | 2.56 | A_11_P152338 | calpastatin | -2.75 |
| A_11_P194503 | DnaJ (Hsp40) homolog, subfamily A, member 3 | 2.55 | A_11_P0000032062 | furin (paired basic amino acid cleaving enzyme) | -2.75 |
| A_11_P000002985 |  | 2.55 | A_11_P0000024507 | sterol regulatory element binding transcription factor 1 | -2.75 |
| A_11_P115371 | transcription elongation factor A (SII)-like 1 | 2.55 | A_11_P080156 | F-box and leucine-rich repeat protein 3 | -2.75 |
| A_11_P165778 |  | 2.55 | A_11_P166363 |  | -2.75 |
| A_11_P051576 | retinol binding protein 4, plasma | 2.55 | A_11_P155538 |  | -2.75 |
| A_11_P190118 | poly (ADP-ribose) polymerase 1 | 2.55 | A_11_P000001791 | sodium channel, voltage-gated, type II, beta subunit | -2.75 |
| A_11_P220428 | vascular endothelial growth factor B | 2.55 | A_11_P0000030827 | protocadherin 1 | -2.75 |
| A_11_P100571 | DEAD (Asp-Glu-Ala-Asp) box helicase 56 | 2.55 | A_11_P0000021885 | extracellular matrix protein 2, female organ and adipocyte specific | -2.75 |
| A_11_P091226 | bystin-like | 2.55 | A_11_P0000022634 | zinc finger, NFX1-type containing 1 | -2.75 |
| A_11_P0000020755 | Ras-related GTP binding D | 2.55 | A_11_P111321 | solute carrier family 28 (concentrative nucleoside transporter), member 3 | -2.75 |
| A_11_P0000021278 | ectonucleotide pyrophosphatase/phosphodiesterase 6 | 2.55 | A_11_P0000026625 | cysteine/histidine-rich 1 | -2.75 |
| A_11_P0000021824 | HD domain containing 2 | 2.55 | A_11_P0000027173 | zinc finger CCCH-type, antiviral 1 | -2.75 |
| A_11_P079246 | collagen, type XXVIII, alpha 1 | 2.55 | A_11_P110301 | WD repeat domain 7 | -2.75 |
| A_11_P181718 | moesin | 2.55 | A_11_P124931 | zinc finger, CCHC domain containing 6 | -2.75 |
| A_11_P070211 | carbonyl reductase family member 4-like | 2.55 | A_11_P00000829 | CREB3 regulatory factor | -2.75 |
| A_11_P0000016352 |  | 2.55 | A_11_P120231 | olfactomedin-like 2A | -2.74 |
| A_11_P0000024752 |  | 2.55 | A_11_P0000032445 | ADP-ribosylation factor-like 15 | -2.74 |
| A_11_P0000027637 | cytoglobin | 2.54 | A_11_P00000781 | junction plakoglobin | -2.74 |
| A_11_P0000035638 |  | 2.54 | A_11_P060496 | phosphatidylserine decarboxylase | -2.74 |
| A_11_P0000032193 | arylsulfatase family, member K | 2.54 | A_11_P187183 |  | -2.74 |
| A_11_P201178 |  | 2.54 | A_11_P130616 |  | -2.74 |
| A_11_P074801 | solute carrier family 41, member 3 | 2.54 | A_11_P178268 | activating signal cointegrator 1 complex subunit 2 | -2.74 |
| A_11_P085641 | ERI1 exoribonuclease family member 3 | 2.54 | A_11_P0000027958 | EH domain binding protein 1-like 1 | -2.74 |
| A_11_P128278 | eukaryotic translation elongation factor 1 alpha 1 | 2.54 | A_11_P094906 | KIAA2026 ortholog | -2.74 |
| A_11_P143313 |  | 2.54 | A_11_P0000021635 | phospholipase A2, group XVI-like | -2.74 |
| A_11_P000004843 |  | 2.54 | A_11_P158023 |  | -2.74 |
| A_11_P145878 | collagen, type I, alpha 2 | 2.54 | A_11_P170108 |  | -2.73 |
| A_11_P169963 | hydroxysteroid dehydrogenase like 2 | 2.54 | A_11_P099926 | SET binding protein 1 | -2.73 |
| A_11_P109196 |  | 2.54 | A_11_P188063 |  | -2.73 |
| A_11_P168423 |  | 2.54 | A_11_P210433 |  | -2.73 |
| A_11_P097611 | nth endonuclease III-like 1 (E. coli) | 2.53 | A_11_P215123 |  | -2.73 |
| A_11_P0000026839 | homeobox A2 | 2.53 | A_11_P107426 | agrin | -2.73 |
| A_11_P0000019484 |  | 2.53 | A_11_P0000024886 | leucine rich repeat containing 8 family, member B | -2.73 |
| A_11_P053226 | ribosomal protein L13a | 2.53 | A_11_P154738 | BSD domain containing 1 | -2.73 |
| A_11_P0000030304 | Rac GTPase activating protein 1 | 2.53 | A_11_P140476 | interferon-inducible GTPase 1-like | -2.73 |
| A_11_P0000016707 |  | 2.53 | A_11_P000003032 |  | -2.73 |
| A_11_P0000019901 | adrenomedullin | 2.53 | A_11_P0000024725 | quinolinate phosphoribosyltransferase | -2.72 |
| A_11_P060996 | stromal cell-derived factor 2-like 1 | 2.53 | A_11_P0000025709 | choroideremia (Rab escort protein 1) | -2.72 |
| A_11_P187298 | transducin (beta)-like 3 | 2.53 | A_11_P0000020965 |  | -2.72 |
| A_11_P0000041288 | latrophilin 2 | 2.53 | A_11_P196548 |  | -2.72 |
| A_11_P077206 | solute carrier family 39 (zinc transporter), member 3 | 2.53 | A_11_P109156 | A kinase (PRKA) anchor protein 13 | -2.72 |
| A_11_P083831 |  | 2.53 | A_11_P0000010259 |  | -2.72 |
| A_11_P0000028100 | zinc finger E-box binding homeobox 2 | 2.53 | A_11_P0000020726 | family with sequence similarity 135, member A | -2.72 |
| A_11_P0000020458 | partner of NOB1 homolog (S. cerevisiae) | 2.53 | A_11_P0000031309 | ATP-binding cassette, sub-family G (WHITE), member 1 | -2.72 |
| A_11_P082371 | zinc finger, HIT-type containing 3 | 2.53 | A_11_P194543 |  | -2.72 |
| A_11_P0000015437 | fasciculation and elongation protein zeta 1 (zygin I) | 2.53 | A_11_P139741 |  | -2.72 |
| A_11_P0000022123 | IMP (inosine 5'-monophosphate) dehydrogenase 2 | 2.53 | A_11_P0000015651 |  | -2.72 |
| A_11_P100441 | dymeclin | 2.52 | A_11_P092411 | solute carrier family 7 (amino acid transporter light chain, y+L system), member 7 | -2.71 |
| A_11_P0000017974 |  | 2.52 | A_11_P000006756 |  | -2.71 |
| A_11_P0000017342 | valyl-tRNA synthetase | 2.52 | A_11_P138456 |  | -2.71 |
| A_11_P119336 | platelet/endothelial cell adhesion molecule 1 | 2.52 | A_11_P068511 | discs, large homolog 2 (Drosophila) | -2.71 |
| A_11_P0000040496 |  | 2.52 | A_11_P098276 | GIPC PDZ domain containing family, member 2 | -2.71 |
| A_11_P092416 |  | 2.52 | A_11_P163903 |  | -2.71 |
| A_11_P119541 | kelch-like family member 26 | 2.52 | A_11_P0000027456 | protein tyrosine phosphatase, non-receptor type 22 (lymphoid) | -2.71 |
| A_11_P0000029606 | retinoblastoma binding protein 9 | 2.52 | A_11_P169098 | 3-oxo-5-beta-steroid 4-dehydrogenase-like | -2.71 |
| A_11_P0000010403 |  | 2.52 | A_11_P147758 |  | -2.71 |
| A_11_P0000022546 | transmembrane protein 230 | 2.52 | A_11_P172658 | E74-like factor 1 (ets domain transcription factor) | -2.71 |
| A_11_P0000021129 | hedgehog interacting protein | 2.52 | A_11_P091391 | runt-related transcription factor 2 | -2.71 |
| A_11_P0000020349 | solute carrier family 25 (mitochondrial carrier; peroxisomal membrane protein, 34kDa), member 17 | 2.51 | A_11_P0000029589 | cyclin L1 | -2.71 |
| A_11_P211568 |  | 2.51 | A_11_P099736 | pre-B-cell leukemia homeobox interacting protein 1 | -2.71 |
| A_11_P073836 | tumor protein p53 inducible protein 11 | 2.51 | A_11_P000004395 |  | -2.71 |
| A_11_P0000027770 | cleavage and polyadenylation factor I subunit 1 | 2.51 | A_11_P137051 | mesoderm induction early response 1, transcriptional regulator | -2.70 |
| A_11_P194118 | lysophosphatidylcholine acyltransferase 1 | 2.51 | A_11_P097891 |  | -2.70 |
| A_11_P0000017301 |  | 2.51 | A_11_P103301 | aurora kinase A and ninein interacting protein | -2.70 |
| A_11_P194528 | guanine nucleotide binding protein (G protein), beta polypeptide 2-like 1 | 2.51 | A_11_P0000019887 | interleukin 18 (interferon-gamma-inducing factor) | -2.70 |
| A_11_P0000019727 | dynein, light chain, Tctex-type 3 | 2.51 | A_11_P141075 | KIAA2026 ortholog | -2.70 |
| A_11_P062571 | poly (ADP-ribose) polymerase family, member 16 | 2.51 | A_11_P0000028910 | olfactory receptor 7C1-like | -2.70 |
| A_11_P0000011980 |  | 2.51 | A_11_P000007484 |  | -2.70 |
| A_11_P090856 | major histocompatibility complex, class II, DO beta | 2.51 | A_11_P0000015257 |  | -2.70 |
| A_11_P0000019716 | trefoil factor 3 (intestinal) | 2.51 | A_11_P059306 | PDZ and LIM domain 5 | -2.70 |
| A_11_P059371 |  | 2.51 | A_11_P148608 | membrane protein, palmitoylated 6 (MAGUK p55 subfamily member 6) | -2.70 |
| A_11_P056271 | PAK1 interacting protein 1 | 2.51 | A_11_P0000031841 | caspase 8, apoptosis-related cysteine peptidase | -2.70 |
| A_11_P000007661 |  | 2.51 | A_11_P0000028300 | transmembrane protein 2 | -2.69 |
| A_11_P198953 | A kinase (PRKA) anchor protein 2 | 2.51 | A_11_P0000010234 |  | -2.69 |
| A_11_P0000020614 | ribonuclease P/MRP 21kDa subunit | 2.51 | A_11_P0000026161 | protein tyrosine phosphatase, receptor type, D | -2.69 |
| A_11_P117961 |  | 2.51 | A_11_P213983 | cancer susceptibility candidate 3 | -2.69 |
| A_11_P163823 |  | 2.51 | A_11_P0000019650 |  | -2.69 |
| A_11_P211018 | mitochondrial ribosomal protein L37 | 2.51 | A_11_P152988 |  | -2.69 |
| A_11_P081061 |  | 2.51 | A_11_P169213 | WD repeat domain 60 | -2.69 |
| A_11_P096031 | ORAI calcium release-activated calcium modulator 2 | 2.50 | A_11_P0000033090 |  | -2.69 |
| A_11_P105891 | hypoxia up-regulated 1 | 2.50 | A_11_P0000015077 |  | -2.69 |
| A_11_P0000013960 |  | 2.50 | A_11_P0000019030 |  | -2.69 |
| A_11_P050736 | prohibitin | 2.50 | A_11_P0000029576 | G protein-coupled receptor 171 | -2.69 |
| A_11_P212538 | zinc finger protein 428 | 2.50 | A_11_P0000015094 |  | -2.69 |
| A_11_P184973 | glutaminyl-tRNA synthase (glutamine-hydrolyzing)-like 1 | 2.50 | A_11_P000006619 |  | -2.69 |
| A_11_P067041 | signal-regulatory protein alpha | 2.50 | A_11_P193238 |  | -2.69 |
| A_11_P163788 |  | 2.50 | A_11_P096881 | retinoblastoma binding protein 6 | -2.69 |
| A_11_P0000039662 | synaptotagmin XIII | 2.50 | A_11_P066466 | pleckstrin homology domain containing, family G (with RhoGef domain) member 6 | -2.69 |
| A_11_P192393 |  | 2.50 | A_11_P084101 | transmembrane protein 65 | -2.69 |
| A_11_P0000015587 |  | 2.50 | A_11_P0000019916 | phospholipase A2, group VII (platelet-activating factor acetylhydrolase, plasma) | -2.69 |
| A_11_P0000032632 | chromosome 5 open reading frame, human C11orf70 | 2.50 | A_11_P0000026621 | diacylglycerol O-acyltransferase 1 | -2.68 |
| A_11_P204448 | eukaryotic translation initiation factor 3, subunit G | 2.50 | A_11_P217143 |  | -2.68 |
| A_11_P000003692 |  | 2.50 | A_11_P0000027041 | transmembrane and coiled-coil domain family 3 | -2.68 |
| A_11_P0000021616 | ribonuclease H2, subunit C | 2.50 | A_11_P000002525 |  | -2.68 |
| A_11_P0000015092 |  | 2.49 | A_11_P0000041232 |  | -2.68 |
| A_11_P0000024999 | myelin protein zero-like 1 | 2.49 | A_11_P0000014830 |  | -2.68 |
| A_11_P110966 | dermatan sulfate epimerase | 2.49 | A_11_P000003995 |  | -2.68 |
| A_11_P0000046 | DNA replication and sister chromatid cohesion 1 | 2.49 | A_11_P051275 | MHC class I DLA-88 | -2.68 |
| A_11_P0000016854 | Rho GTPase activating protein 42 | 2.49 | A_11_P000008757 |  | -2.68 |
| A_11_P0000016599 |  | 2.49 | A_11_P0000019670 | endothelin 3 | -2.68 |
| A_11_P174658 | zinc finger protein 576 | 2.49 | A_11_P185923 | meningioma expressed antigen 5 (hyaluronidase) | -2.68 |
| A_11_P051196 | CD163 molecule | 2.49 | A_11_P190088 |  | -2.68 |
| A_11_P0000026464 | TTK protein kinase | 2.49 | A_11_P0000012410 |  | -2.68 |
| A_11_P160123 | reticulon 4 interacting protein 1 | 2.49 | A_11_P0000032732 | 5',3'-nucleotidase, mitochondrial | -2.68 |
| A_11_P106896 | elaC ribonuclease Z 2 | 2.49 | A_11_P0000024159 | family with sequence similarity 114, member A1 | -2.67 |
| A_11_P0000032712 | centromere protein V | 2.49 | A_11_P056146 | chromodomain protein, Y-like | -2.67 |
| A_11_P193263 | PR domain containing 6 | 2.49 | A_11_P138356 |  | -2.67 |
| A_11_P113229 |  | 2.49 | A_11_P0000041759 |  | -2.67 |
| A_11_P0000014460 | staphylococcal nuclease and tudor domain containing 1 | 2.49 | A_11_P0000031758 | interferon induced with helicase C domain 1 | -2.67 |
| A_11_P150858 | deoxyribonuclease II, lysosomal | 2.49 | A_11_P144353 | insulin induced gene 1 | -2.67 |
| A_11_P00000944 | nucleoredoxin | 2.49 | A_11_P152173 |  | -2.67 |
| A_11_P125731 | SMG5 nonsense mediated mRNA decay factor | 2.49 | A_11_P183613 | zinc finger protein 513 | -2.67 |
| A_11_P0000022533 | destrin (actin depolymerizing factor) | 2.49 | A_11_P0000029730 | syndecan 4 | -2.67 |
| A_11_P169313 |  | 2.49 | A_11_P127746 |  | -2.67 |
| A_11_P194428 | neuron-derived neurotrophic factor | 2.49 | A_11_P0000014554 | CREB3 regulatory factor | -2.67 |
| A_11_P195333 |  | 2.49 | A_11_P0000010012 |  | -2.67 |
| A_11_P0000022794 | eukaryotic translation initiation factor 2B, subunit 1 alpha, 26kDa | 2.49 | A_11_P00000167 | proline-rich nuclear receptor coactivator 1 | -2.67 |
| A_11_P115926 | four and a half LIM domains 1 | 2.48 | A_11_P0000029836 |  | -2.67 |
| A_11_P113751 | free fatty acid receptor 3 | 2.48 | A_11_P0000013027 |  | -2.67 |
| A_11_P113086 | translocase of outer mitochondrial membrane 40 homolog (yeast) | 2.48 | A_11_P0000041410 |  | -2.67 |
| A_11_P186388 | neuroguidin, EIF4E binding protein | 2.48 | A_11_P0000017148 | transmembrane 4 L six family member 4 | -2.67 |
| A_11_P0000021493 | laminin, beta 1 | 2.48 | A_11_P0000030959 | WAS protein family, member 2 | -2.66 |
| A_11_P0000040915 |  | 2.48 | A_11_P0000019143 |  | -2.66 |
| A_11_P076516 | chromosome 20 open reading frame, human C19orf53 | 2.48 | A_11_P000003 | DnaJ (Hsp40) homolog, subfamily C, member 14 | -2.66 |
| A_11_P198758 |  | 2.48 | A_11_P0000033641 | uncharacterized LOC607937 | -2.66 |
| A_11_P0000022391 |  | 2.48 | A_11_P183843 | family with sequence similarity 190, member B | -2.66 |
| A_11_P117811 | multiple PDZ domain protein | 2.48 | A_11_P0000010153 |  | -2.66 |
| A_11_P190653 |  | 2.48 | A_11_P202528 |  | -2.66 |
| A_11_P064686 | sema domain, seven thrombospondin repeats (type 1 and type 1-like), transmembrane domain (TM) and short cytoplasmic domain, (semaphorin) 5A | 2.48 | A_11_P087241 |  | -2.66 |
| A_11_P093111 | tRNA methyltransferase 5 | 2.48 | A_11_P000002555 |  | -2.66 |
| A_11_P0000013864 |  | 2.48 | A_11_P141095 | CXXC finger protein 4 | -2.66 |
| A_11_P0000014877 |  | 2.48 | A_11_P0000015619 | lectin, galactoside-binding-like | -2.66 |
| A_11_P092841 | FK506 binding protein 3, 25kDa | 2.48 | A_11_P0000037367 |  | -2.66 |
| A_11_P074911 | acyl-CoA dehydrogenase family, member 9 | 2.48 | A_11_P0000041755 |  | -2.66 |
| A_11_P0000015137 |  | 2.48 | A_11_P0000026922 | rhomboid, veinlet-like 2 (Drosophila) | -2.66 |
| A_11_P0000030334 | histone deacetylase 7 | 2.48 | A_11_P0000013352 |  | -2.66 |
| A_11_P0000039037 |  | 2.48 | A_11_P0000018780 |  | -2.66 |
| A_11_P053211 | annexin A1 | 2.47 | A_11_P152728 | calcium binding and coiled-coil domain 2 | -2.66 |
| A_11_P0000020177 | MHC class II DR alpha chain | 2.47 | A_11_P096046 | collagen, type XXVI, alpha 1 | -2.65 |
| A_11_P0000020194 | KIT ligand | 2.47 | A_11_P071471 | K(lysine) acetyltransferase 2B | -2.65 |
| A_11_P0000026894 | zinc finger protein 691 | 2.47 | A_11_P124316 | prostate transmembrane protein, androgen induced 1 | -2.65 |
| A_11_P061816 |  | 2.47 | A_11_P077681 | maltase-glucoamylase (alpha-glucosidase) | -2.65 |
| A_11_P0000026119 | phosphorylated adaptor for RNA export | 2.47 | A_11_P087201 | mitogen-activated protein kinase kinase kinase kinase 3 | -2.65 |
| A_11_P121106 |  | 2.47 | A_11_P0000039420 | LIM domain 7 | -2.65 |
| A_11_P113301 | exosome component 5 | 2.47 | A_11_P115196 | diaphanous-related formin 2 | -2.65 |
| A_11_P126146 | RAB38, member RAS oncogene family | 2.47 | A_11_P0000025392 | sperm associated antigen 9 | -2.65 |
| A_11_P0000023670 | 3'-phosphoadenosine 5'-phosphosulfate synthase 1 | 2.47 | A_11_P0000020280 | kinesin family member 5A | -2.65 |
| A_11_P195888 |  | 2.46 | A_11_P161268 |  | -2.65 |
| A_11_P0000025537 | angiopoietin-like 2 | 2.46 | A_11_P077596 | transient receptor potential cation channel, subfamily V, member 6 | -2.65 |
| A_11_P061891 | BUB1 mitotic checkpoint serine/threonine kinase B | 2.46 | A_11_P0000019761 | solute carrier family 15 (oligopeptide transporter), member 1 | -2.65 |
| A_11_P000001040 |  | 2.46 | A_11_P159573 |  | -2.65 |
| A_11_P0000019879 | S100 calcium binding protein A4 | 2.46 | A_11_P089551 | myosin, heavy chain 9, non-muscle | -2.65 |
| A_11_P0000025529 | prostaglandin E synthase 2 | 2.46 | A_11_P0000039584 |  | -2.65 |
| A_11_P0000041352 |  | 2.46 | A_11_P062086 | stereocilin | -2.65 |
| A_11_P114311 | phosphatidylinositol glycan anchor biosynthesis, class N | 2.46 | A_11_P0000021761 | malic enzyme 2, NAD(+)-dependent, mitochondrial | -2.65 |
| A_11_P050096 | transmembrane protein 47 | 2.46 | A_11_P127041 |  | -2.64 |
| A_11_P0000023828 | lymphocyte antigen 86 | 2.46 | A_11_P061486 | bromodomain and WD repeat domain containing 1 | -2.64 |
| A_11_P111506 | phosphatidylinositol glycan anchor biosynthesis, class N | 2.46 | A_11_P181373 |  | -2.64 |
| A_11_P219773 | centrosomal protein 112kDa | 2.46 | A_11_P191158 |  | -2.64 |
| A_11_P165643 |  | 2.46 | A_11_P139056 |  | -2.64 |
| A_11_P0000039311 | glutathione peroxidase 3 (plasma) | 2.46 | A_11_P0000015872 |  | -2.64 |
| A_11_P0000021945 | SUMO1 activating enzyme subunit 1 | 2.46 | A_11_P0000020345 |  | -2.64 |
| A_11_P0000028944 | RAD23 homolog A (S. cerevisiae) | 2.46 | A_11_P191123 | 5'-nucleotidase, cytosolic IB | -2.64 |
| A_11_P0000034639 |  | 2.46 | A_11_P00000609 | solute carrier family 26 (anion exchanger), member 6 | -2.64 |
| A_11_P116181 | signal sequence receptor, delta | 2.46 | A_11_P0000027836 | cOR4C3 olfactory receptor family 4 subfamily X-like | -2.64 |
| A_11_P150753 | transmembrane protein 47 | 2.46 | A_11_P0000031 | ring finger protein 19A, E3 ubiquitin protein ligase | -2.64 |
| A_11_P0000038948 |  | 2.46 | A_11_P189913 | basic leucine zipper nuclear factor 1 | -2.64 |
| A_11_P170683 | collagen, type XII, alpha 1 | 2.45 | A_11_P079181 | protein phosphatase 1, regulatory subunit 9A | -2.64 |
| A_11_P050681 | phosphatidylethanolamine binding protein 1 | 2.45 | A_11_P135131 |  | -2.64 |
| A_11_P161168 |  | 2.45 | A_11_P056341 | jumonji, AT rich interactive domain 2 | -2.64 |
| A_11_P0000029830 | SMAD family member 9 | 2.45 | A_11_P0000021698 | cyclin T2 | -2.64 |
| A_11_P184833 |  | 2.45 | A_11_P185373 |  | -2.63 |
| A_11_P0000040550 |  | 2.45 | A_11_P0000029104 | midnolin | -2.63 |
| A_11_P104106 |  | 2.45 | A_11_P0000025240 | ectonucleoside triphosphate diphosphohydrolase 5 | -2.63 |
| A_11_P000002766 |  | 2.45 | A_11_P0000022973 | intermediate filament tail domain containing 1 | -2.63 |
| A_11_P0000022895 | RAN binding protein 1 | 2.45 | A_11_P055111 | serine peptidase inhibitor, Kazal type 5 | -2.63 |
| A_11_P0000041360 |  | 2.45 | A_11_P062001 |  | -2.63 |
| A_11_P126441 | fibronectin leucine rich transmembrane protein 3 | 2.45 | A_11_P0000023132 | pleckstrin homology domain containing, family A (phosphoinositide binding specific) member 1 | -2.63 |
| A_11_P051231 | sulfatase 2 | 2.45 | A_11_P0000024782 | sorting nexin 29 | -2.63 |
| A_11_P186303 | heat shock protein 90kDa beta (Grp94), member 1 | 2.45 | A_11_P064291 | XK, Kell blood group complex subunit-related family, member 9 | -2.63 |
| A_11_P171168 | tumor protein D52-like 2 | 2.45 | A_11_P0000040264 |  | -2.63 |
| A_11_P066046 | fatty acid binding protein 5 (psoriasis-associated) | 2.45 | A_11_P000004859 |  | -2.63 |
| A_11_P0000029516 | thyroid hormone receptor, beta | 2.45 | A_11_P173388 |  | -2.63 |
| A_11_P179878 |  | 2.45 | A_11_P0000041914 |  | -2.63 |
| A_11_P000005707 |  | 2.45 | A_11_P000005766 |  | -2.63 |
| A_11_P0000016326 |  | 2.44 | A_11_P0000017424 |  | -2.63 |
| A_11_P0000020115 | toll-like receptor 2 | 2.44 | A_11_P154053 | pleckstrin homology domain containing, family A (phosphoinositide binding specific) member 1 | -2.62 |
| A_11_P0000015344 |  | 2.44 | A_11_P0000020471 | cysteine dioxygenase type 1 | -2.62 |
| A_11_P0000027999 | WD repeat domain 74 | 2.44 | A_11_P094286 | interferon-inducible GTPase 1-like | -2.62 |
| A_11_P0000038840 |  | 2.44 | A_11_P066906 | adrenergic, alpha-1D-, receptor | -2.62 |
| A_11_P110641 | phosphatidylinositol glycan anchor biosynthesis, class N | 2.44 | A_11_P0000015509 | patatin-like phospholipase domain containing 2 | -2.62 |
| A_11_P0000040999 | heat shock 70kDa protein 4-like | 2.44 | A_11_P149488 |  | -2.62 |
| A_11_P067481 | DSN1, MIS12 kinetochore complex component | 2.44 | A_11_P0000039833 | lectin, galactoside-binding-like | -2.62 |
| A_11_P106056 | cell adhesion molecule 1 | 2.44 | A_11_P0000012059 |  | -2.62 |
| A_11_P000002961 |  | 2.44 | A_11_P164233 |  | -2.62 |
| A_11_P0000024165 | TBC1 domain family, member 19 | 2.44 | A_11_P194613 |  | -2.62 |
| A_11_P0000039246 |  | 2.44 | A_11_P168498 |  | -2.62 |
| A_11_P0000020297 | Mdm1 nuclear protein homolog (mouse) | 2.44 | A_11_P059101 | lin-54 homolog (C. elegans) | -2.62 |
| A_11_P097566 | cyclin F | 2.44 | A_11_P150198 | selenoprotein P, plasma, 1 | -2.62 |
| A_11_P000003640 |  | 2.44 | A_11_P000002814 | AMMECR1-like | -2.62 |
| A_11_P196303 |  | 2.44 | A_11_P000009729 |  | -2.62 |
| A_11_P144933 |  | 2.44 | A_11_P0000028193 | zinc finger and BTB domain containing 2 | -2.62 |
| A_11_P188043 | NIMA-related kinase 2 | 2.44 | A_11_P0000032881 | cap methyltransferase 2 | -2.62 |
| A_11_P050741 | frataxin | 2.44 | A_11_P071841 |  | -2.62 |
| A_11_P078286 |  | 2.44 | A_11_P058766 | oxysterol binding protein-like 11 | -2.62 |
| A_11_P0000023824 | enoyl-CoA delta isomerase 2 | 2.44 | A_11_P087056 | lysocardiolipin acyltransferase 1 | -2.62 |
| A_11_P0000028793 | POC1 centriolar protein A | 2.44 | A_11_P0000029847 | GS homeobox 1 | -2.62 |
| A_11_P072706 | mitochondrial ribosomal protein L32 | 2.44 | A_11_P104706 | CREB3 regulatory factor | -2.61 |
| A_11_P056816 | RAN, member RAS oncogene family | 2.44 | A_11_P207738 |  | -2.61 |
| A_11_P0000031692 | histone H1.1-like | 2.44 | A_11_P208068 |  | -2.61 |
| A_11_P191828 |  | 2.44 | A_11_P171768 |  | -2.61 |
| A_11_P0000023688 |  | 2.44 | A_11_P199098 | solute carrier family 39 (zinc transporter), member 4 | -2.61 |
| A_11_P187533 | plexin domain containing 1 | 2.44 | A_11_P075401 | deoxyribonuclease I-like 3 | -2.61 |
| A_11_P184428 |  | 2.44 | A_11_P154523 |  | -2.61 |
| A_11_P182878 |  | 2.44 | A_11_P0000026624 | solute carrier family 39 (zinc transporter), member 4 | -2.61 |
| A_11_P0000036383 |  | 2.44 | A_11_P099546 | leucine rich repeat containing 71 | -2.61 |
| A_11_P191033 |  | 2.43 | A_11_P0000014289 |  | -2.61 |
| A_11_P075036 | TSEN2 tRNA splicing endonuclease subunit | 2.43 | A_11_P0000029569 | Hermansky-Pudlak syndrome 3 | -2.61 |
| A_11_P152483 | fibrillin-1 | 2.43 | A_11_P0000017888 |  | -2.61 |
| A_11_P0000024669 | general transcription factor IIi | 2.43 | A_11_P0000033627 |  | -2.61 |
| A_11_P170423 |  | 2.43 | A_11_P000009681 |  | -2.61 |
| A_11_P067581 | family with sequence similarity 83, member D | 2.43 | A_11_P0000033429 | basic leucine zipper nuclear factor 1 | -2.61 |
| A_11_P050916 | angiopoietin 2 | 2.43 | A_11_P057251 |  | -2.60 |
| A_11_P00000685 | hemicentin 1 | 2.43 | A_11_P150703 | cyclin-dependent kinase inhibitor 1A (p21, Cip1) | -2.60 |
| A_11_P0000022139 | FYVE and coiled-coil domain containing 1 | 2.43 | A_11_P053551 | interleukin 18 (interferon-gamma-inducing factor) | -2.60 |
| A_11_P180678 |  | 2.43 | A_11_P100251 | structural maintenance of chromosomes flexible hinge domain containing 1 | -2.60 |
| A_11_P168428 | carbonyl reductase family member 4-like | 2.43 | A_11_P210993 |  | -2.60 |
| A_11_P172673 |  | 2.43 | A_11_P092316 | uncharacterized LOC490595 | -2.60 |
| A_11_P0000022353 | emopamil binding protein-like | 2.43 | A_11_P0000027125 | Rho guanine nucleotide exchange factor (GEF) 5 | -2.60 |
| A_11_P098371 | cystathionase (cystathionine gamma-lyase) | 2.43 | A_11_P0000016550 | cryptochrome 2 (photolyase-like) | -2.60 |
| A_11_P064456 | carbonic anhydrase II | 2.43 | A_11_P067366 | tumor protein p53 inducible nuclear protein 2 | -2.60 |
| A_11_P000007489 |  | 2.43 | A_11_P000006714 |  | -2.60 |
| A_11_P125541 |  | 2.43 | A_11_P152303 | FRY-like | -2.60 |
| A_11_P0000016328 | potassium inwardly-rectifying channel, subfamily J, member 8 | 2.42 | A_11_P0000024344 | poly (ADP-ribose) polymerase family, member 8 | -2.60 |
| A_11_P115497 | ER membrane protein complex subunit 2-like | 2.42 | A_11_P218018 |  | -2.60 |
| A_11_P0000019963 | v-myc myelocytomatosis viral oncogene homolog (avian) | 2.42 | A_11_P063936 | clarin 3 | -2.60 |
| A_11_P210408 |  | 2.42 | A_11_P0000019074 |  | -2.60 |
| A_11_P0000021031 | mitochondrial ribosomal protein S15 | 2.42 | A_11_P00000516 | PDZ and LIM domain 5 | -2.60 |
| A_11_P199653 | minichromosome maintenance complex component 6 | 2.42 | A_11_P105541 | suppression of tumorigenicity 14 (colon carcinoma) | -2.60 |
| A_11_P0000023547 | kinesin family member 23 | 2.42 | A_11_P0000019380 |  | -2.60 |
| A_11_P0000028813 | sema domain, immunoglobulin domain (Ig), short basic domain, secreted, (semaphorin) 3F | 2.42 | A_11_P000003689 |  | -2.60 |
| A_11_P090661 | allograft inflammatory factor 1 | 2.42 | A_11_P0000016662 |  | -2.60 |
| A_11_P000003321 |  | 2.42 | A_11_P0000041160 |  | -2.60 |
| A_11_P0000031772 | SPC25, NDC80 kinetochore complex component | 2.42 | A_11_P0000034753 | premature ovarian failure, 1B | -2.60 |
| A_11_P0000020267 | coenzyme Q10 homolog A (S. cerevisiae) | 2.42 | A_11_P092476 | interleukin 25 | -2.60 |
| A_11_P0000016399 |  | 2.42 | A_11_P000004129 |  | -2.60 |
| A_11_P070371 | elongator acetyltransferase complex subunit 3 | 2.42 | A_11_P0000035430 |  | -2.59 |
| A_11_P086978 | glucokinase (hexokinase 4) regulator | 2.42 | A_11_P0000012118 |  | -2.59 |
| A_11_P078326 | RNA binding protein with multiple splicing | 2.42 | A_11_P096546 | alpha hemoglobin stabilizing protein | -2.59 |
| A_11_P0000032918 | family with sequence similarity 65, member A | 2.42 | A_11_P0000024552 | agrin | -2.59 |
| A_11_P0000021124 | insulin-like growth factor 1 (somatomedin C) | 2.42 | A_11_P0000010626 |  | -2.59 |
| A_11_P000003306 |  | 2.41 | A_11_P0000015902 |  | -2.59 |
| A_11_P107916 | GINS complex subunit 2 (Psf2 homolog) | 2.41 | A_11_P149833 |  | -2.59 |
| A_11_P000004855 | collagen, type XII, alpha 1 | 2.41 | A_11_P122016 | cyclin-dependent kinase inhibitor 1A (p21, Cip1) | -2.59 |
| A_11_P00000289 |  | 2.41 | A_11_P053751 | spectrin, alpha, non-erythrocytic 1 | -2.59 |
| A_11_P0000023085 | nucleophosmin/nucleoplasmin 3 | 2.41 | A_11_P0000018609 |  | -2.59 |
| A_11_P146113 |  | 2.41 | A_11_P0000015139 |  | -2.59 |
| A_11_P0000021065 | nuclear autoantigenic sperm protein (histone-binding) | 2.41 | A_11_P058521 | GRAM domain containing 1C | -2.59 |
| A_11_P0000015471 |  | 2.41 | A_11_P171908 |  | -2.59 |
| A_11_P187133 |  | 2.41 | A_11_P064796 | coiled-coil domain containing 39 | -2.59 |
| A_11_P0000038796 |  | 2.41 | A_11_P0000016349 | solute carrier family 13 (sodium-dependent dicarboxylate transporter), member 2 | -2.59 |
| A_11_P177093 | cell adhesion molecule 3 | 2.41 | A_11_P0000025358 | junction plakoglobin | -2.59 |
| A_11_P148598 | RAB11 family interacting protein 5 (class I) | 2.41 | A_11_P0000040548 |  | -2.59 |
| A_11_P0000040918 |  | 2.41 | A_11_P0000030759 | OTU domain containing 1 | -2.58 |
| A_11_P086641 | ribonucleotide reductase M2 | 2.41 | A_11_P051106 | BCL2-like 1 | -2.58 |
| A_11_P00000115 | very low density lipoprotein receptor | 2.41 | A_11_P0000020147 | reticulon 1 | -2.58 |
| A_11_P0000026196 | ubiquitin associated protein 2 | 2.41 | A_11_P096236 |  | -2.58 |
| A_11_P0000026563 | DEP domain containing MTOR-interacting protein | 2.40 | A_11_P123456 | elongation factor, RNA polymerase II, 2 | -2.58 |
| A_11_P00000578 | karyopherin alpha 2 (RAG cohort 1, importin alpha 1) | 2.40 | A_11_P0000016175 | 1-acylglycerol-3-phosphate O-acyltransferase 3 | -2.58 |
| A_11_P0000021364 | MORN repeat containing 2 | 2.40 | A_11_P069521 | cytochrome P450, family 2, subfamily R, polypeptide 1 | -2.58 |
| A_11_P076601 | calponin 1, basic, smooth muscle | 2.40 | A_11_P0000036196 |  | -2.58 |
| A_11_P168733 | thimet oligopeptidase 1 | 2.40 | A_11_P055436 |  | -2.58 |
| A_11_P0000015683 | sarcospan | 2.40 | A_11_P0000040204 |  | -2.58 |
| A_11_P0000017170 | protocadherin 12 | 2.40 | A_11_P00000756 | tumor necrosis factor (ligand) superfamily, member 10 | -2.58 |
| A_11_P152658 |  | 2.40 | A_11_P0000040618 |  | -2.58 |
| A_11_P0000017152 |  | 2.40 | A_11_P000002262 |  | -2.58 |
| A_11_P0000024723 |  | 2.40 | A_11_P181698 | prostate transmembrane protein, androgen induced 1 | -2.58 |
| A_11_P074501 | protein phosphatase 1, regulatory (inhibitor) subunit 14B | 2.40 | A_11_P128226 |  | -2.58 |
| A_11_P142503 | peptide deformylase (mitochondrial) | 2.40 | A_11_P0000041518 |  | -2.58 |
| A_11_P0000022178 | growth arrest and DNA-damage-inducible, gamma interacting protein 1 | 2.40 | A_11_P066961 | attractin | -2.58 |
| A_11_P052936 | peptidylprolyl isomerase A (cyclophilin A) pseudogene | 2.40 | A_11_P174093 |  | -2.58 |
| A_11_P0000015912 |  | 2.40 | A_11_P065536 | UDP-N-acetyl-alpha-D-galactosamine:polypeptide N-acetylgalactosaminyltransferase 6 (GalNAc-T6) | -2.57 |
| A_11_P137461 |  | 2.39 | A_11_P058691 | deltex 3-like (Drosophila) | -2.57 |
| A_11_P139106 |  | 2.39 | A_11_P158193 |  | -2.57 |
| A_11_P0000039664 |  | 2.39 | A_11_P212968 | glutathione reductase | -2.57 |
| A_11_P0000016459 | tyrosine kinase with immunoglobulin-like and EGF-like domains 1 | 2.39 | A_11_P098726 | lysophosphatidylglycerol acyltransferase 1 | -2.57 |
| A_11_P052296 | beta-defensin 108B-like | 2.39 | A_11_P055201 | myosin, heavy chain 3, skeletal muscle, embryonic | -2.57 |
| A_11_P0000022130 |  | 2.39 | A_11_P0000015699 |  | -2.57 |
| A_11_P0000018906 | centromere protein B, 80kDa | 2.39 | A_11_P085921 | protein phosphatase 1, regulatory subunit 12A | -2.57 |
| A_11_P120121 |  | 2.39 | A_11_P063921 |  | -2.57 |
| A_11_P0000040367 |  | 2.39 | A_11_P083591 | Ral GEF with PH domain and SH3 binding motif 1 | -2.57 |
| A_11_P0000023811 | serpin peptidase inhibitor, clade I (neuroserpin), member 1 | 2.39 | A_11_P0000024408 | ubiquitination factor E4A | -2.57 |
| A_11_P0000024775 | myosin, heavy chain 11, smooth muscle | 2.39 | A_11_P0000038561 |  | -2.57 |
| A_11_P0000027239 |  | 2.39 | A_11_P0000025545 | golgin A1 | -2.57 |
| A_11_P080636 | mitochondrial ribosomal protein L12 | 2.39 | A_11_P000008447 |  | -2.57 |
| A_11_P106771 | RAN guanine nucleotide release factor | 2.39 | A_11_P000006086 |  | -2.57 |
| A_11_P0000018364 |  | 2.39 | A_11_P0000027909 | two pore segment channel 2 | -2.57 |
| A_11_P0000016596 |  | 2.39 | A_11_P0000039359 |  | -2.57 |
| A_11_P125436 | enolase 2 (gamma, neuronal) | 2.39 | A_11_P210413 | calsyntenin 1 | -2.56 |
| A_11_P077066 | chromosome 20 open reading frame, human C19orf10 | 2.39 | A_11_P078276 | BCL2-associated athanogene 4 | -2.56 |
| A_11_P056351 | CAP, adenylate cyclase-associated protein, 2 (yeast) | 2.39 | A_11_P0000012566 |  | -2.56 |
| A_11_P151813 | coenzyme Q10 homolog A (S. cerevisiae) | 2.38 | A_11_P193748 |  | -2.56 |
| A_11_P093241 | eukaryotic translation initiation factor 2, subunit 1 alpha, 35kDa | 2.38 | A_11_P0000018564 |  | -2.56 |
| A_11_P0000020567 | polymerase (RNA) I polypeptide E, 53kDa | 2.38 | A_11_P0000027414 |  | -2.56 |
| A_11_P106951 | centromere protein V | 2.38 | A_11_P0000027769 |  | -2.56 |
| A_11_P059461 | 3'-phosphoadenosine 5'-phosphosulfate synthase 1 | 2.38 | A_11_P199948 |  | -2.56 |
| A_11_P0000028696 | minichromosome maintenance complex component 2 | 2.38 | A_11_P149908 | protein phosphatase 1, catalytic subunit, beta isozyme | -2.56 |
| A_11_P00000132 | isoleucyl-tRNA synthetase | 2.38 | A_11_P113386 | sterile alpha motif domain containing 4B | -2.56 |
| A_11_P069751 | coiled-coil domain containing 34 | 2.38 | A_11_P0000029495 | 5-beta-cholestane-3-alpha,7-alpha-diol 12-alpha-hydroxylase-like | -2.56 |
| A_11_P0000029877 | Sin3A-associated protein, 30kDa | 2.38 | A_11_P0000023585 | synaptojanin 1 | -2.56 |
| A_11_P187528 |  | 2.38 | A_11_P000001292 | carnitine palmitoyltransferase 1A (liver) | -2.55 |
| A_11_P0000023297 |  | 2.38 | A_11_P0000030514 | cytoplasmic polyadenylation element binding protein 3 | -2.55 |
| A_11_P202213 |  | 2.38 | A_11_P0000021818 |  | -2.55 |
| A_11_P167728 | zinc finger protein 496 | 2.38 | A_11_P0000015854 | exocyst complex component 1 | -2.55 |
| A_11_P153858 | cyclin B2 | 2.38 | A_11_P0000028705 | IQ motif and Sec7 domain 1 | -2.55 |
| A_11_P0000020556 | stomatin (EPB72)-like 2 | 2.38 | A_11_P203213 | tudor domain containing 7 | -2.55 |
| A_11_P0000015991 | DnaJ (Hsp40) homolog, subfamily A, member 3 | 2.38 | A_11_P000004175 |  | -2.55 |
| A_11_P000005667 |  | 2.38 | A_11_P0000027145 |  | -2.55 |
| A_11_P0000020262 | ribosomal protein S26 | 2.38 | A_11_P093486 | feline leukemia virus subgroup C cellular receptor family, member 2 | -2.55 |
| A_11_P050911 | thioredoxin reductase 3 | 2.38 | A_11_P211498 | laminin, beta 3 | -2.55 |
| A_11_P0000016297 | TEA domain family member 2 | 2.38 | A_11_P221540 | apoptosis resistant E3 ubiquitin protein ligase 1 | -2.55 |
| A_11_P000003712 |  | 2.38 | A_11_P0000022434 | cAMP-regulated phosphoprotein, 21kDa | -2.55 |
| A_11_P054227 |  | 2.38 | A_11_P000003325 |  | -2.55 |
| A_11_P0000018517 |  | 2.38 | A_11_P0000029700 | Src-like-adaptor 2 | -2.55 |
| A_11_P0000030025 |  | 2.37 | A_11_P080366 | uncharacterized LOC100855512 | -2.55 |
| A_11_P140316 |  | 2.37 | A_11_P0000024179 | adenomatous polyposis coli | -2.55 |
| A_11_P0000020734 | CD109 molecule | 2.37 | A_11_P0000034737 | ATPase, Cu++ transporting, alpha polypeptide | -2.55 |
| A_11_P085636 | ERI1 exoribonuclease family member 3 | 2.37 | A_11_P105406 | cadherin 10, type 2 (T2-cadherin) | -2.55 |
| A_11_P0000028215 | acetyl-CoA acetyltransferase 2 | 2.37 | A_11_P173418 | 1,4-alpha-glucan-branching enzyme-like | -2.55 |
| A_11_P088376 |  | 2.37 | A_11_P0000039745 |  | -2.55 |
| A_11_P067141 | family with sequence similarity 110, member A | 2.37 | A_11_P0000017634 |  | -2.55 |
| A_11_P078401 | tumor suppressor candidate 3 | 2.37 | A_11_P053941 |  | -2.55 |
| A_11_P0000024850 | chloride channel CLIC-like 1 | 2.37 | A_11_P168703 |  | -2.55 |
| A_11_P119706 | zinc finger protein 70 | 2.37 | A_11_P0000040689 | G protein-coupled receptor 133 | -2.55 |
| A_11_P0000038713 |  | 2.37 | A_11_P106201 |  | -2.55 |
| A_11_P0000040237 |  | 2.37 | A_11_P200603 |  | -2.55 |
| A_11_P132371 |  | 2.37 | A_11_P0000029564 | procollagen-lysine, 2-oxoglutarate 5-dioxygenase 2 | -2.55 |
| A_11_P0000028520 | solute carrier family 1 (neutral amino acid transporter), member 5 | 2.37 | A_11_P059651 | E1A binding protein p400 | -2.54 |
| A_11_P0000028512 | EH-domain containing 2 | 2.37 | A_11_P0000019959 | WAP four-disulfide core domain 2 | -2.54 |
| A_11_P161973 | hydroxysteroid (17-beta) dehydrogenase 7 | 2.37 | A_11_P0000031374 | glutamate receptor, ionotropic, delta 2 | -2.54 |
| A_11_P0000034218 | mitochondrial ribosomal protein S2 | 2.37 | A_11_P130951 |  | -2.54 |
| A_11_P107381 | NDC1 transmembrane nucleoporin | 2.37 | A_11_P134551 |  | -2.54 |
| A_11_P140156 |  | 2.37 | A_11_P182688 |  | -2.54 |
| A_11_P0000040242 |  | 2.37 | A_11_P174568 | REV3-like, polymerase (DNA directed), zeta, catalytic subunit | -2.54 |
| A_11_P0000023631 |  | 2.37 | A_11_P00000555 | serine/threonine kinase 32C | -2.54 |
| A_11_P064766 | thyroid hormone receptor interactor 13 | 2.37 | A_11_P0000041229 |  | -2.54 |
| A_11_P092006 | glutaminyl-tRNA synthase (glutamine-hydrolyzing)-like 1 | 2.37 | A_11_P207788 | spermatogenesis associated, serine-rich 2-like | -2.54 |
| A_11_P000005142 | translocase of outer mitochondrial membrane 20 homolog (yeast) | 2.37 | A_11_P000004455 | lysophosphatidylglycerol acyltransferase 1 | -2.54 |
| A_11_P087116 |  | 2.37 | A_11_P000005312 |  | -2.54 |
| A_11_P0000029423 | importin 5 | 2.36 | A_11_P086081 | cyclin-dependent kinase 17 | -2.54 |
| A_11_P139706 | endothelin receptor type B | 2.36 | A_11_P0000016899 |  | -2.54 |
| A_11_P0000026118 | aldehyde dehydrogenase 7 family, member A1 | 2.36 | A_11_P0000010683 |  | -2.54 |
| A_11_P083001 | mitochondrial ribosomal protein L41 | 2.36 | A_11_P0000019437 |  | -2.54 |
| A_11_P056946 | proteasome (prosome, macropain) 26S subunit, non-ATPase, 14 | 2.36 | A_11_P147159 | TLC domain containing 1 | -2.54 |
| A_11_P0000024748 |  | 2.36 | A_11_P0000019773 | uncoupling protein 2 (mitochondrial, proton carrier) | -2.54 |
| A_11_P064681 |  | 2.36 | A_11_P000008851 |  | -2.54 |
| A_11_P097646 | heparan sulfate (glucosamine) 3-O-sulfotransferase 6 | 2.36 | A_11_P165583 |  | -2.54 |
| A_11_P151808 | stomatin (EPB72)-like 2 | 2.36 | A_11_P194493 |  | -2.54 |
| A_11_P0000024154 | ribosomal protein L9 | 2.36 | A_11_P057041 | serine threonine kinase 39 | -2.54 |
| A_11_P0000019677 | toll-like receptor 4 | 2.36 | A_11_P108671 |  | -2.54 |
| A_11_P0000036403 | platelet derived growth factor D | 2.36 | A_11_P105881 | C2CD2-like | -2.53 |
| A_11_P0000025937 | glycine C-acetyltransferase | 2.36 | A_11_P212848 | cytohesin 1 | -2.53 |
| A_11_P121776 | SUB1 homolog (S. cerevisiae) | 2.36 | A_11_P0000028403 | BR serine/threonine kinase 1 | -2.53 |
| A_11_P0000022696 | general transcription factor IIIA | 2.36 | A_11_P0000032805 |  | -2.53 |
| A_11_P0000033590 | TGFB-induced factor homeobox 1 | 2.36 | A_11_P095536 | solute carrier family 44 (choline transporter), member 1 | -2.53 |
| A_11_P169253 | A kinase (PRKA) anchor protein 12 | 2.36 | A_11_P053626 | sterol regulatory element binding transcription factor 1 | -2.53 |
| A_11_P0000024490 | elongation factor 1-alpha 1-like | 2.36 | A_11_P178498 |  | -2.53 |
| A_11_P0000016500 | ubiquitin-conjugating enzyme E2T (putative) | 2.36 | A_11_P0000026005 | ankyrin repeat domain 23 | -2.53 |
| A_11_P0000021725 | thioredoxin-like 4A | 2.36 | A_11_P0000017595 |  | -2.53 |
| A_11_P0000016615 | interferon-related developmental regulator 2 | 2.36 | A_11_P110471 | microtubule-associated protein 7 | -2.53 |
| A_11_P0000028158 | transcription factor 21 | 2.36 | A_11_P00000175 | endonuclease/exonuclease/phosphatase family domain containing 1 | -2.53 |
| A_11_P0000029671 | hemopoietic cell kinase | 2.36 | A_11_P192783 | sestrin 1 | -2.53 |
| A_11_P193008 |  | 2.36 | A_11_P0000018741 |  | -2.52 |
| A_11_P0000038868 |  | 2.35 | A_11_P150598 | zinc finger, NFX1-type containing 1 | -2.52 |
| A_11_P114341 | dystrophin | 2.35 | A_11_P225108 | transforming growth factor, beta receptor III | -2.52 |
| A_11_P111041 | anti-silencing function 1A histone chaperone | 2.35 | A_11_P0000019759 | protein phosphatase 1, catalytic subunit, beta isozyme | -2.52 |
| A_11_P0000019768 | RAD51 homolog (S. cerevisiae) | 2.35 | A_11_P0000022073 |  | -2.52 |
| A_11_P0000030062 | DEAD (Asp-Glu-Ala-Asp) box polypeptide 54 | 2.35 | A_11_P176423 |  | -2.52 |
| A_11_P076266 | mitochondrial ribosomal protein L34 | 2.35 | A_11_P073866 | mucin-6-like | -2.52 |
| A_11_P125501 | transmembrane protein 230 | 2.35 | A_11_P00000164 | ubiquitin specific peptidase 34 | -2.52 |
| A_11_P0000033 | poly(A) binding protein, cytoplasmic 1 | 2.35 | A_11_P0000011343 |  | -2.52 |
| A_11_P123941 | phosphohistidine phosphatase 1 | 2.35 | A_11_P0000023131 | transforming, acidic coiled-coil containing protein 2 | -2.52 |
| A_11_P0000018732 | dephospho-CoA kinase domain containing | 2.35 | A_11_P094271 | immunity-related GTPase family M protein-like | -2.52 |
| A_11_P0000039313 | leucine-rich repeat containing G protein-coupled receptor 4 | 2.35 | A_11_P0000019802 | UDP glucuronosyltransferase 1 family, polypeptide A6 | -2.52 |
| A_11_P150423 | valosin containing protein | 2.35 | A_11_P050156 | transient receptor potential cation channel, subfamily M, member 8 | -2.52 |
| A_11_P224923 |  | 2.35 | A_11_P000003839 |  | -2.52 |
| A_11_P055711 | hydroxysteroid (17-beta) dehydrogenase 7 | 2.35 | A_11_P0000027168 | poly (ADP-ribose) polymerase family, member 12 | -2.52 |
| A_11_P00000318 | exosome component 8 | 2.35 | A_11_P104101 | jumonji domain containing 1C | -2.52 |
| A_11_P0000028577 |  | 2.35 | A_11_P0000024853 | vav 3 guanine nucleotide exchange factor | -2.51 |
| A_11_P0000018829 |  | 2.35 | A_11_P169508 | G protein-coupled receptor 116 | -2.51 |
| A_11_P0000017277 | beta-site APP-cleaving enzyme 1 | 2.35 | A_11_P0000019830 | solute carrier family 3 (cystine, dibasic and neutral amino acid transporters, activator of cystine, dibasic and neutral amino acid transport), member 1 | -2.51 |
| A_11_P101436 |  | 2.35 | A_11_P196438 | jumonji, AT rich interactive domain 2 | -2.51 |
| A_11_P184943 | histocompatibility (minor) 13 | 2.35 | A_11_P0000020597 | lysophosphatidic acid receptor 1 | -2.51 |
| A_11_P084896 | COX18 cytochrome c oxidase assembly homolog (S. cerevisiae) | 2.35 | A_11_P0000027726 | DEP domain containing 7 | -2.51 |
| A_11_P074236 | UDP-GlcNAc:betaGal beta-1,3-N-acetylglucosaminyltransferase 1 | 2.35 | A_11_P000004943 |  | -2.51 |
| A_11_P082796 | RNA methyltransferase like 1 | 2.35 | A_11_P052616 | MHC class I DLA-64 | -2.51 |
| A_11_P074271 | barrier to autointegration factor 1 | 2.35 | A_11_P0000038078 |  | -2.51 |
| A_11_P173228 | adducin 3 (gamma) | 2.34 | A_11_P0000030949 | phosphatase and actin regulator 4 | -2.51 |
| A_11_P110286 | asparaginyl-tRNA synthetase | 2.34 | A_11_P000007594 |  | -2.51 |
| A_11_P0000020984 | glycyl-tRNA synthetase | 2.34 | A_11_P099921 | solute carrier family 14 (urea transporter), member 2 | -2.51 |
| A_11_P054116 | ribosomal protein L18 | 2.34 | A_11_P070761 | diacylglycerol kinase, delta 130kDa | -2.51 |
| A_11_P205798 | immunoglobulin J polypeptide, linker protein for immunoglobulin alpha and mu polypeptides | 2.34 | A_11_P058971 |  | -2.51 |
| A_11_P0000041776 |  | 2.34 | A_11_P068351 | chromosome 21 open reading frame, human C11orf54 | -2.51 |
| A_11_P0000033526 | S100 calcium binding protein A13 | 2.34 | A_11_P104496 | family with sequence similarity 190, member B | -2.51 |
| A_11_P194723 | kinesin family member 22 | 2.34 | A_11_P0000021390 | ring finger protein 103 | -2.51 |
| A_11_P0000039505 | glycosyltransferase 8 domain containing 1 | 2.34 | A_11_P099046 | RAB GTPase activating protein 1-like | -2.51 |
| A_11_P0000020168 | solute carrier family 12 (potassium/chloride transporters), member 4 | 2.34 | A_11_P0000014965 |  | -2.51 |
| A_11_P0000022026 | acyl-CoA dehydrogenase family, member 9 | 2.34 | A_11_P0000032057 | sema domain, immunoglobulin domain (Ig), transmembrane domain (TM) and short cytoplasmic domain, (semaphorin) 4B | -2.51 |
| A_11_P058426 | pleckstrin homology-like domain, family B, member 2 | 2.34 | A_11_P061461 |  | -2.51 |
| A_11_P192028 | ribosomal protein S3A | 2.34 | A_11_P086426 | lecithin retinol acyltransferase (phosphatidylcholine--retinol O-acyltransferase) | -2.51 |
| A_11_P117706 | fibroblast growth factor 13-like | 2.34 | A_11_P106086 | zinc finger and BTB domain containing 16 | -2.51 |
| A_11_P138786 |  | 2.34 | A_11_P0000022283 | ring finger protein 121 | -2.51 |
| A_11_P208628 | dihydrouridine synthase 1-like (S. cerevisiae) | 2.34 | A_11_P0000013686 |  | -2.51 |
| A_11_P076746 | peter pan homolog (Drosophila) | 2.34 | A_11_P0000017410 |  | -2.51 |
| A_11_P0000022372 | kelch repeat and BTB (POZ) domain containing 6 | 2.34 | A_11_P000005318 |  | -2.51 |
| A_11_P070261 | centrosomal protein 44kDa | 2.34 | A_11_P206428 | NEDD4 binding protein 1 | -2.50 |
| A_11_P0000015420 | selenoprotein X, 1 | 2.34 | A_11_P210153 |  | -2.50 |
| A_11_P0000011273 |  | 2.34 | A_11_P158988 |  | -2.50 |
| A_11_P177188 | ribosomal protein L15 | 2.34 | A_11_P094546 | GRAM domain containing 3 | -2.50 |
| A_11_P109356 | SH3-domain GRB2-like 3 | 2.34 | A_11_P00000447 | Rap guanine nucleotide exchange factor (GEF) 2 | -2.50 |
| A_11_P168433 | protein O-linked mannose N-acetylglucosaminyltransferase 1 (beta 1,2-) | 2.34 | A_11_P000002584 |  | -2.50 |
| A_11_P182728 | guanosine monophosphate reductase | 2.34 | A_11_P0000015542 | histocompatibility (minor) HA-1 | -2.50 |
| A_11_P139571 |  | 2.34 | A_11_P0000039340 |  | -2.50 |
| A_11_P0000021625 | tRNA methyltransferase 11-2 homolog (S. cerevisiae) | 2.33 | A_11_P122011 |  | -2.50 |
| A_11_P0000024295 | PRELI domain containing 1 | 2.33 | A_11_P0000031045 | tumor necrosis factor receptor superfamily, member 1B | -2.50 |
| A_11_P0000015226 |  | 2.33 | A_11_P0000033722 | GTP cyclohydrolase 1 | -2.50 |
| A_11_P130976 | ubiquitin family domain containing 1 | 2.33 | A_11_P0000018434 |  | -2.50 |
| A_11_P0000025602 |  | 2.33 | A_11_P0000033767 | KIAA0247 ortholog | -2.50 |
| A_11_P213598 | importin 5 | 2.33 | A_11_P185898 |  | -2.50 |
| A_11_P099901 | HAUS augmin-like complex, subunit 1 | 2.33 | A_11_P0000026833 | membrane protein, palmitoylated 6 (MAGUK p55 subfamily member 6) | -2.50 |
| A_11_P0000021977 | fibrillarin | 2.33 | A_11_P128961 |  | -2.49 |
| A_11_P0000024950 | neutrophil cytosolic factor 2 | 2.33 | A_11_P0000032497 | suppression of tumorigenicity 14 (colon carcinoma) | -2.49 |
| A_11_P0000039201 |  | 2.33 | A_11_P0000023732 | karyopherin alpha 1 (importin alpha 5) | -2.49 |
| A_11_P0000022968 | mitochondrial ribosomal protein S35 | 2.33 | A_11_P187773 | CCR4-NOT transcription complex, subunit 4 | -2.49 |
| A_11_P0000025401 |  | 2.33 | A_11_P0000017913 |  | -2.49 |
| A_11_P116286 | C-x(9)-C motif containing 4 homolog (S. cerevisiae) | 2.33 | A_11_P000004087 |  | -2.49 |
| A_11_P100491 | 2-oxoglutarate and iron-dependent oxygenase domain containing 3 | 2.33 | A_11_P0000011243 |  | -2.49 |
| A_11_P131006 |  | 2.33 | A_11_P0000021623 | EH-domain containing 1 | -2.49 |
| A_11_P0000040901 | DnaJ (Hsp40) homolog, subfamily C, member 10 | 2.33 | A_11_P165173 | cell death-inducing DFFA-like effector a | -2.49 |
| A_11_P050306 | ubiquitin A-52 residue ribosomal protein fusion product 1 | 2.33 | A_11_P0000028490 | fibroblast growth factor 21 | -2.49 |
| A_11_P163183 | adaptor-related protein complex 1, sigma 2 subunit | 2.33 | A_11_P0000030427 | apolipoprotein B mRNA editing enzyme, catalytic polypeptide 1 | -2.49 |
| A_11_P0000032594 | RNA exonuclease 2 | 2.33 | A_11_P058056 | solute carrier family 23, member 3 | -2.49 |
| A_11_P106701 | eukaryotic translation initiation factor 4A1 | 2.33 | A_11_P0000032410 | SAP30-like | -2.49 |
| A_11_P081151 |  | 2.33 | A_11_P071081 | zinc finger protein 445 | -2.49 |
| A_11_P198383 |  | 2.33 | A_11_P050841 | ATP-binding cassette, sub-family B (MDR/TAP), member 1 | -2.49 |
| A_11_P0000028048 | sprouty homolog 1, antagonist of FGF signaling (Drosophila) | 2.33 | A_11_P111046 |  | -2.49 |
| A_11_P219183 | aquaporin 5 | 2.33 | A_11_P0000030375 | ethanolamine kinase 1 | -2.49 |
| A_11_P0000027294 | vascular endothelial growth factor C | 2.33 | A_11_P201853 |  | -2.49 |
| A_11_P182193 | KAT8 regulatory NSL complex subunit 2 | 2.33 | A_11_P203933 | basic helix-loop-helix domain containing, class B, 9 | -2.49 |
| A_11_P179988 |  | 2.33 | A_11_P145583 |  | -2.49 |
| A_11_P000005118 | SHC SH2-domain binding protein 1 | 2.33 | A_11_P0000027157 | maltase-glucoamylase, intestinal-like | -2.49 |
| A_11_P0000025953 | parvalbumin | 2.33 | A_11_P051596 | inositol polyphosphate-5-phosphatase, 40kDa | -2.49 |
| A_11_P0000029316 | UDP-N-acetyl-alpha-D-galactosamine:polypeptide N-acetylgalactosaminyltransferase 18 | 2.32 | A_11_P159518 |  | -2.49 |
| A_11_P196163 |  | 2.32 | A_11_P000007548 |  | -2.48 |
| A_11_P0000027638 | ST6 (alpha-N-acetyl-neuraminyl-2,3-beta-galactosyl-1,3)-N-acetylgalactosaminide alpha-2,6-sialyltransferase 1 | 2.32 | A_11_P0000020479 | CDC-like kinase 4 | -2.48 |
| A_11_P100761 |  | 2.32 | A_11_P0000011577 |  | -2.48 |
| A_11_P0000029608 | sorting nexin 5 | 2.32 | A_11_P0000015522 |  | -2.48 |
| A_11_P190678 |  | 2.32 | A_11_P0000024633 | polyamine modulated factor 1 binding protein 1 | -2.48 |
| A_11_P0000021258 |  | 2.32 | A_11_P194253 |  | -2.48 |
| A_11_P089306 | solute carrier family 25 (mitochondrial carrier; peroxisomal membrane protein, 34kDa), member 17 | 2.32 | A_11_P0000031450 | phosphatidylinositol-specific phospholipase C, X domain containing 2 | -2.48 |
| A_11_P0000026946 | origin recognition complex, subunit 6 | 2.32 | A_11_P068796 | inositol polyphosphate phosphatase-like 1 | -2.48 |
| A_11_P080121 | dachshund homolog 1 (Drosophila) | 2.32 | A_11_P190833 | ataxin 7 | -2.48 |
| A_11_P192168 |  | 2.32 | A_11_P0000041120 |  | -2.48 |
| A_11_P172868 | FK506 binding protein 4, 59kDa | 2.32 | A_11_P0000040612 |  | -2.48 |
| A_11_P0000039361 |  | 2.32 | A_11_P0000030539 | R3H domain and coiled-coil containing 1-like | -2.48 |
| A_11_P0000030111 | tyrosylprotein sulfotransferase 2 | 2.32 | A_11_P0000040341 |  | -2.48 |
| A_11_P080131 | progesterone immunomodulatory binding factor 1 | 2.32 | A_11_P160623 |  | -2.48 |
| A_11_P085581 | 4-hydroxyphenylpyruvate dioxygenase-like | 2.32 | A_11_P0000023271 | diaphanous-related formin 1 | -2.48 |
| A_11_P050591 | aquaporin 1 (Colton blood group) | 2.32 | A_11_P109261 | furin (paired basic amino acid cleaving enzyme) | -2.48 |
| A_11_P160588 | adenylate cyclase 2 (brain) | 2.32 | A_11_P197803 | dystonin | -2.48 |
| A_11_P121201 |  | 2.31 | A_11_P185948 |  | -2.48 |
| A_11_P0000020269 | canopy FGF signaling regulator 2 | 2.31 | A_11_P171793 |  | -2.47 |
| A_11_P0000013744 |  | 2.31 | A_11_P109936 |  | -2.47 |
| A_11_P000007746 |  | 2.31 | A_11_P0000025572 | chromosome 18 open reading frame, human C7orf57 | -2.47 |
| A_11_P052446 | signal recognition particle 72kDa | 2.31 | A_11_P057426 |  | -2.47 |
| A_11_P0000026840 | homeobox A5 | 2.31 | A_11_P0000031362 | kelch-like family member 8 | -2.47 |
| A_11_P0000033150 | N-acetylglucosamine-1-phosphodiester alpha-N-acetylglucosaminidase | 2.31 | A_11_P050391 | eukaryotic translation initiation factor 2-alpha kinase 2 | -2.47 |
| A_11_P0000020748 | synaptotagmin binding, cytoplasmic RNA interacting protein | 2.31 | A_11_P0000013954 |  | -2.47 |
| A_11_P097641 | selenoprotein X, 1 | 2.31 | A_11_P053701 | gap junction protein, beta 2, 26kDa | -2.47 |
| A_11_P113436 | mitochondrial ribosomal protein S12 | 2.31 | A_11_P0000029690 | matrix metallopeptidase 24 (membrane-inserted) | -2.47 |
| A_11_P0000040180 |  | 2.31 | A_11_P200928 |  | -2.47 |
| A_11_P168798 | membrane-associated ring finger (C3HC4) 9 | 2.31 | A_11_P000007045 |  | -2.47 |
| A_11_P0000031564 | presenilin associated, rhomboid-like | 2.31 | A_11_P194778 |  | -2.47 |
| A_11_P0000040936 |  | 2.31 | A_11_P181663 |  | -2.47 |
| A_11_P0000018886 | polymeric immunoglobulin receptor | 2.31 | A_11_P0000011100 | hepcidin antimicrobial peptide | -2.46 |
| A_11_P116821 | coiled-coil-helix-coiled-coil-helix domain containing 6 | 2.31 | A_11_P0000030437 | calsyntenin 3 | -2.46 |
| A_11_P194348 |  | 2.31 | A_11_P000009953 |  | -2.46 |
| A_11_P0000016356 | cell division cycle associated 8 | 2.31 | A_11_P0000017398 |  | -2.46 |
| A_11_P073036 |  | 2.31 | A_11_P0000031574 | enoyl-CoA, hydratase/3-hydroxyacyl CoA dehydrogenase | -2.46 |
| A_11_P0000021245 |  | 2.31 | A_11_P150733 |  | -2.46 |
| A_11_P123506 | family with sequence similarity 184, member B | 2.31 | A_11_P0000033000 | zinc finger and SCAN domain containing 25 | -2.46 |
| A_11_P0000027381 | cytochrome P450, family 1, subfamily B, polypeptide 1 | 2.31 | A_11_P0000032108 | SH3-domain binding protein 2 | -2.46 |
| A_11_P117406 | flap structure-specific endonuclease 1 | 2.31 | A_11_P195313 |  | -2.46 |
| A_11_P000004336 |  | 2.31 | A_11_P0000012957 |  | -2.46 |
| A_11_P0000033141 | class II, major histocompatibility complex, transactivator | 2.31 | A_11_P198443 |  | -2.46 |
| A_11_P0000038779 | translocase of inner mitochondrial membrane 22 homolog (yeast) | 2.31 | A_11_P000009764 |  | -2.46 |
| A_11_P000005187 |  | 2.30 | A_11_P175974 |  | -2.46 |
| A_11_P0000034246 | chromosome 9 open reading frame, human C9orf114 | 2.30 | A_11_P116196 | renin binding protein | -2.46 |
| A_11_P156048 |  | 2.30 | A_11_P0000016384 | period circadian clock 2 | -2.46 |
| A_11_P084471 |  | 2.30 | A_11_P0000033709 | mitogen-activated protein kinase kinase kinase kinase 5 | -2.46 |
| A_11_P0000013941 |  | 2.30 | A_11_P0000026867 | THAP domain containing 5 | -2.46 |
| A_11_P216603 | fibrillarin | 2.30 | A_11_P093096 |  | -2.46 |
| A_11_P0000029610 | beaded filament structural protein 1, filensin | 2.30 | A_11_P000008028 |  | -2.46 |
| A_11_P0000020404 | lipoyltransferase 1 | 2.30 | A_11_P202623 |  | -2.46 |
| A_11_P0000024091 | mitochondrial ribosomal protein L46 | 2.30 | A_11_P178248 |  | -2.46 |
| A_11_P0000031255 | hemK methyltransferase family member 2-like | 2.30 | A_11_P0000040845 | cadherin 1, type 1, E-cadherin (epithelial) | -2.46 |
| A_11_P160463 |  | 2.30 | A_11_P0000031515 | catenin (cadherin-associated protein), delta 2 | -2.46 |
| A_11_P103846 | phosphogluconate dehydrogenase | 2.30 | A_11_P193253 |  | -2.46 |
| A_11_P0000040917 |  | 2.30 | A_11_P187493 |  | -2.46 |
| A_11_P193718 |  | 2.30 | A_11_P0000022709 | intraflagellar transport 88 homolog (Chlamydomonas) | -2.46 |
| A_11_P093891 | tryptophanyl-tRNA synthetase | 2.30 | A_11_P095776 |  | -2.45 |
| A_11_P050436 | RAB27A, member RAS oncogene family | 2.30 | A_11_P053386 | ATP-binding cassette, sub-family B (MDR/TAP), member 1 | -2.45 |
| A_11_P203668 | protein TBRG4-like | 2.30 | A_11_P0000022242 | chromosome 21 open reading frame, human C11orf54 | -2.45 |
| A_11_P0000010363 |  | 2.30 | A_11_P192073 | Bardet-Biedl syndrome 2 | -2.45 |
| A_11_P182268 |  | 2.30 | A_11_P0000027146 | transient receptor potential cation channel, subfamily V, member 5 | -2.45 |
| A_11_P098401 | DEP domain containing 1 | 2.30 | A_11_P0000023962 | CDC-like kinase 1 | -2.45 |
| A_11_P0000021816 | dermatan sulfate epimerase | 2.30 | A_11_P140731 |  | -2.45 |
| A_11_P0000030399 | matrix Gla protein | 2.30 | A_11_P0000018446 |  | -2.45 |
| A_11_P0000022716 | carbonyl reductase family member 4-like | 2.30 | A_11_P137406 |  | -2.45 |
| A_11_P057141 | solute carrier family 25 (aspartate/glutamate carrier), member 12 | 2.29 | A_11_P161578 | transmembrane protein 2 | -2.45 |
| A_11_P179393 | eukaryotic translation initiation factor 5A | 2.29 | A_11_P0000024576 | calsyntenin 1 | -2.45 |
| A_11_P107346 | mitochondrial ribosomal protein L37 | 2.29 | A_11_P213543 |  | -2.45 |
| A_11_P197298 | family with sequence similarity 149, member A | 2.29 | A_11_P132896 | transmembrane protein 2 | -2.45 |
| A_11_P050776 | dystrophin | 2.29 | A_11_P087636 | tetratricopeptide repeat domain 31 | -2.45 |
| A_11_P085156 | chromosome 15 open reading frame, human C1orf109 | 2.29 | A_11_P083676 | DENN/MADD domain containing 1A | -2.45 |
| A_11_P082361 | chromosome 9 open reading frame, human C17orf64 | 2.29 | A_11_P113121 |  | -2.45 |
| A_11_P114801 | hydroxysteroid (17-beta) dehydrogenase 10 | 2.29 | A_11_P069711 | leucine zipper protein 2-like | -2.45 |
| A_11_P0000018916 |  | 2.29 | A_11_P084626 | solute carrier family 10 (sodium/bile acid cotransporter family), member 4 | -2.45 |
| A_11_P0000021891 | ribosomal protein S5 | 2.29 | A_11_P066881 | chromosome 24 open reading frame, human C20orf196 | -2.45 |
| A_11_P148283 |  | 2.29 | A_11_P088211 |  | -2.45 |
| A_11_P0000029862 | crystallin, lambda 1 | 2.29 | A_11_P0000010040 |  | -2.44 |
| A_11_P0000014508 | ring finger protein 125, E3 ubiquitin protein ligase | 2.29 | A_11_P169488 |  | -2.44 |
| A_11_P0000033530 | S100 calcium binding protein A8 | 2.29 | A_11_P0000025858 | protein tyrosine phosphatase, receptor type, B | -2.44 |
| A_11_P0000026821 | cell division cycle associated 7-like | 2.29 | A_11_P0000038499 |  | -2.44 |
| A_11_P0000022796 | Rab interacting lysosomal protein-like 1 | 2.29 | A_11_P0000019397 |  | -2.44 |
| A_11_P0000025489 | chloride intracellular channel 3 | 2.29 | A_11_P0000032426 | N-deacetylase/N-sulfotransferase (heparan glucosaminyl) 1 | -2.44 |
| A_11_P0000030056 |  | 2.29 | A_11_P093396 | paraneoplastic Ma antigen 1 | -2.44 |
| A_11_P168328 |  | 2.29 | A_11_P174778 |  | -2.44 |
| A_11_P0000014457 |  | 2.28 | A_11_P000008901 |  | -2.44 |
| A_11_P0000023773 | actin-like 6A | 2.28 | A_11_P0000039599 |  | -2.44 |
| A_11_P089071 |  | 2.28 | A_11_P0000013846 |  | -2.44 |
| A_11_P113526 | Yip1 interacting factor homolog B (S. cerevisiae) | 2.28 | A_11_P0000010537 |  | -2.44 |
| A_11_P0000019678 | gap junction protein, alpha 1, 43kDa | 2.28 | A_11_P138206 |  | -2.44 |
| A_11_P139011 |  | 2.28 | A_11_P116021 | fragile X mental retardation 1 | -2.44 |
| A_11_P077201 | thimet oligopeptidase 1 | 2.28 | A_11_P0000028966 | lipid phosphate phosphatase-related protein type 2 | -2.44 |
| A_11_P133566 |  | 2.28 | A_11_P0000033577 | mindbomb E3 ubiquitin protein ligase 1 | -2.44 |
| A_11_P0000034843 | UTP14, U3 small nucleolar ribonucleoprotein, homolog A (yeast) | 2.28 | A_11_P105861 | PDZ domain containing 3 | -2.44 |
| A_11_P082186 |  | 2.28 | A_11_P000008440 |  | -2.44 |
| A_11_P0000041834 | serpin peptidase inhibitor, clade F (alpha-2 antiplasmin, pigment epithelium derived factor), member 2 | 2.28 | A_11_P169948 | PRP4 pre-mRNA processing factor 4 homolog B (yeast) | -2.43 |
| A_11_P097936 |  | 2.28 | A_11_P159048 |  | -2.43 |
| A_11_P0000022477 | propionyl CoA carboxylase, beta polypeptide | 2.28 | A_11_P0000017248 | cholinergic receptor, nicotinic, beta 2 (neuronal) | -2.43 |
| A_11_P00000129 | asporin | 2.28 | A_11_P0000031039 | DnaJ (Hsp40) homolog, subfamily C, member 16 | -2.43 |
| A_11_P210913 | deoxycytidylate deaminase-like | 2.28 | A_11_P0000021132 | OTU domain containing 4 | -2.43 |
| A_11_P160438 |  | 2.28 | A_11_P0000030456 | anoctamin 2 | -2.43 |
| A_11_P093266 | actinin, alpha 1 | 2.28 | A_11_P0000038746 |  | -2.43 |
| A_11_P0000033079 | kinesin family member 22 | 2.28 | A_11_P0000031601 | serpin peptidase inhibitor, clade I (pancpin), member 2 | -2.43 |
| A_11_P085081 | peptidylprolyl isomerase E (cyclophilin E) | 2.28 | A_11_P070521 | bone morphogenetic protein 1 | -2.43 |
| A_11_P158328 | Rho guanine nucleotide exchange factor (GEF) 28 | 2.28 | A_11_P185653 |  | -2.43 |
| A_11_P058726 | protein tyrosine phosphatase-like (proline instead of catalytic arginine), member b | 2.28 | A_11_P062916 | mitogen-activated protein kinase 8 | -2.43 |
| A_11_P0000033654 | thiamine triphosphatase | 2.28 | A_11_P171568 |  | -2.43 |
| A_11_P0000037206 |  | 2.28 | A_11_P058751 |  | -2.43 |
| A_11_P0000025880 | angiopoietin-related protein 5-like | 2.28 | A_11_P0000020045 | cytochrome P450c21 | -2.43 |
| A_11_P00000495 | chitinase domain containing 1 | 2.28 | A_11_P0000024008 | protein tyrosine phosphatase, receptor type, N | -2.43 |
| A_11_P173393 | EGF containing fibulin-like extracellular matrix protein 1 | 2.28 | A_11_P0000017803 |  | -2.43 |
| A_11_P194518 |  | 2.28 | A_11_P0000012418 |  | -2.43 |
| A_11_P000003753 |  | 2.27 | A_11_P0000012457 |  | -2.43 |
| A_11_P0000030742 | cleavage stimulation factor, 3' pre-RNA, subunit 2, 64kDa | 2.27 | A_11_P0000012713 |  | -2.43 |
| A_11_P000009799 |  | 2.27 | A_11_P075416 |  | -2.43 |
| A_11_P0000017181 | coiled-coil domain containing 146 | 2.27 | A_11_P128361 | transmembrane protein 41B | -2.43 |
| A_11_P000005837 |  | 2.27 | A_11_P202738 |  | -2.43 |
| A_11_P0000014427 |  | 2.27 | A_11_P139186 |  | -2.43 |
| A_11_P0000028974 | KRI1 homolog (S. cerevisiae) | 2.27 | A_11_P0000013331 |  | -2.43 |
| A_11_P0000040989 |  | 2.27 | A_11_P0000015132 |  | -2.43 |
| A_11_P205402 | tropomyosin 1 (alpha) | 2.27 | A_11_P0000022550 | attractin | -2.43 |
| A_11_P082401 | apoptosis antagonizing transcription factor | 2.27 | A_11_P065421 |  | -2.43 |
| A_11_P090876 | kinesin family member C1 | 2.27 | A_11_P0000042013 |  | -2.43 |
| A_11_P0000029165 | teneurin transmembrane protein 4 | 2.27 | A_11_P078271 | DDHD domain containing 2 | -2.43 |
| A_11_P0000017202 |  | 2.27 | A_11_P134301 |  | -2.42 |
| A_11_P212583 |  | 2.27 | A_11_P082391 | mitochondrial rRNA methyltransferase 1 homolog (S. cerevisiae) | -2.42 |
| A_11_P056386 | membrane bound O-acyltransferase domain containing 1 | 2.27 | A_11_P122181 |  | -2.42 |
| A_11_P0000031781 | methionyl aminopeptidase type 1D (mitochondrial) | 2.27 | A_11_P215213 |  | -2.42 |
| A_11_P0000022305 | DENN/MADD domain containing 5A | 2.27 | A_11_P065896 | FYVE, RhoGEF and PH domain containing 4 | -2.42 |
| A_11_P087176 | gem (nuclear organelle) associated protein 6 | 2.27 | A_11_P0000017313 |  | -2.42 |
| A_11_P0000032340 | docking protein 3 | 2.27 | A_11_P137076 |  | -2.42 |
| A_11_P0000024379 | zinc finger protein 622 | 2.27 | A_11_P000005307 |  | -2.42 |
| A_11_P0000038924 |  | 2.27 | A_11_P0000017443 |  | -2.42 |
| A_11_P066576 | wingless-type MMTV integration site family, member 5B | 2.27 | A_11_P198228 | signal transducer and activator of transcription 3 (acute-phase response factor) | -2.42 |
| A_11_P0000031821 | serum deprivation response | 2.27 | A_11_P00000758 | neutral cholesterol ester hydrolase 1 | -2.42 |
| A_11_P0000020438 |  | 2.27 | A_11_P131836 |  | -2.42 |
| A_11_P139786 | pancreatic alpha-amylase-like | 2.27 | A_11_P208748 |  | -2.42 |
| A_11_P184983 | interleukin 13 receptor, alpha 2 | 2.27 | A_11_P0000028310 | Janus kinase 2 | -2.42 |
| A_11_P162613 | ectonucleotide pyrophosphatase/phosphodiesterase 6 | 2.27 | A_11_P0000012155 |  | -2.42 |
| A_11_P090746 |  | 2.27 | A_11_P0000028299 | family with sequence similarity 108, member B1 | -2.42 |
| A_11_P0000039188 |  | 2.27 | A_11_P0000029750 | phosphatidylinositol-3,4,5-trisphosphate-dependent Rac exchange factor 1 | -2.42 |
| A_11_P0000025813 | SPRY domain containing 4 | 2.27 | A_11_P148883 | ring finger protein 19B | -2.42 |
| A_11_P173913 | chaperonin containing TCP1, subunit 6A (zeta 1) | 2.27 | A_11_P0000013375 |  | -2.42 |
| A_11_P108451 | Ras-related associated with diabetes | 2.27 | A_11_P000005752 |  | -2.42 |
| A_11_P0000029296 | PTPRF interacting protein, binding protein 2 (liprin beta 2) | 2.26 | A_11_P096006 | uroplakin 3B | -2.41 |
| A_11_P0000023051 |  | 2.26 | A_11_P000007788 |  | -2.41 |
| A_11_P116275 | dyskeratosis congenita 1, dyskerin | 2.26 | A_11_P0000039687 |  | -2.41 |
| A_11_P0000021683 | glycophorin C (Gerbich blood group) | 2.26 | A_11_P0000013536 |  | -2.41 |
| A_11_P062356 |  | 2.26 | A_11_P0000040873 |  | -2.41 |
| A_11_P199668 | ribosomal protein L3 | 2.26 | A_11_P079451 | chromosome 14 open reading frame, human C7orf31 | -2.41 |
| A_11_P059131 |  | 2.26 | A_11_P076256 | bone marrow stromal cell antigen 2 | -2.41 |
| A_11_P0000023164 | inositol monophosphatase domain containing 1 | 2.26 | A_11_P193673 | OTU domain containing 5 | -2.41 |
| A_11_P059201 |  | 2.26 | A_11_P108611 | calpastatin | -2.41 |
| A_11_P070201 | IMP4, U3 small nucleolar ribonucleoprotein, homolog (yeast) | 2.26 | A_11_P0000030685 | junctophilin 1 | -2.41 |
| A_11_P204258 |  | 2.26 | A_11_P0000012107 |  | -2.41 |
| A_11_P158093 | solute carrier family 25 (mitochondrial carrier; peroxisomal membrane protein, 34kDa), member 17 | 2.26 | A_11_P0000013633 |  | -2.41 |
| A_11_P079391 | translocase of outer mitochondrial membrane 7 homolog (yeast) | 2.26 | A_11_P0000081 | catenin (cadherin-associated protein), alpha 1, 102kDa | -2.41 |
| A_11_P052641 | major histocompatibility complex, class II, DQ beta 1 | 2.26 | A_11_P0000019500 |  | -2.41 |
| A_11_P0000021021 | tRNA isopentenyltransferase 1 | 2.26 | A_11_P000008039 |  | -2.41 |
| A_11_P177283 |  | 2.26 | A_11_P0000025217 |  | -2.41 |
| A_11_P090461 | NFU1 iron-sulfur cluster scaffold homolog (S. cerevisiae) | 2.26 | A_11_P0000028939 | immediate early response 2 | -2.40 |
| A_11_P067016 | isocitrate dehydrogenase 3 (NAD+) beta | 2.26 | A_11_P0000032362 | sideroflexin 1 | -2.40 |
| A_11_P0000017406 | uncharacterized LOC100687347 | 2.26 | A_11_P0000040063 |  | -2.40 |
| A_11_P104741 | spindle apparatus coiled-coil protein 1 | 2.26 | A_11_P051166 | peroxisome proliferator-activated receptor gamma | -2.40 |
| A_11_P161923 | SPARC related modular calcium binding 2 | 2.26 | A_11_P162263 | AT rich interactive domain 4B (RBP1-like) | -2.40 |
| A_11_P203943 |  | 2.26 | A_11_P0000020434 | spectrin, beta, non-erythrocytic 1 | -2.40 |
| A_11_P000001129 |  | 2.26 | A_11_P0000039744 |  | -2.40 |
| A_11_P0000014531 | translocase of outer mitochondrial membrane 7 homolog (yeast) | 2.26 | A_11_P0000015552 |  | -2.40 |
| A_11_P055021 | heat shock protein 70 | 2.26 | A_11_P165168 | apolipoprotein H (beta-2-glycoprotein I) | -2.40 |
| A_11_P0000015345 | cofilin 2 (muscle) | 2.26 | A_11_P148383 | sterile alpha motif domain containing 4A | -2.40 |
| A_11_P139476 | lipopolysaccharide-induced TNF factor | 2.26 | A_11_P0000035538 |  | -2.40 |
| A_11_P119316 | fidgetin-like 1 | 2.26 | A_11_P0000028967 | low density lipoprotein receptor | -2.40 |
| A_11_P0000023189 |  | 2.26 | A_11_P067881 | zinc finger, NFX1-type containing 1 | -2.40 |
| A_11_P0000022971 | mediator complex subunit 21 | 2.26 | A_11_P0000010119 |  | -2.40 |
| A_11_P088251 | thioesterase superfamily member 4 | 2.26 | A_11_P0000041569 |  | -2.40 |
| A_11_P0000021765 | connective tissue growth factor-like | 2.26 | A_11_P108976 | myotubularin related protein 10 | -2.40 |
| A_11_P162053 |  | 2.26 | A_11_P217543 |  | -2.40 |
| A_11_P199723 |  | 2.25 | A_11_P139766 | regulator of G-protein signaling 3 | -2.40 |
| A_11_P0000032510 | decapping enzyme, scavenger | 2.25 | A_11_P105811 | sortilin-related receptor, L(DLR class) A repeats containing | -2.40 |
| A_11_P053661 | ribosomal protein L19 | 2.25 | A_11_P0000033359 | Fc receptor, IgA, IgM, high affinity | -2.40 |
| A_11_P171903 | KIT ligand | 2.25 | A_11_P0000019123 |  | -2.40 |
| A_11_P0000025405 |  | 2.25 | A_11_P125661 | neutral cholesterol ester hydrolase 1 | -2.40 |
| A_11_P143848 |  | 2.25 | A_11_P0000029713 | lipin 3 | -2.40 |
| A_11_P056006 | CD1e molecule | 2.25 | A_11_P0000022504 | TSC22 domain family, member 2 | -2.39 |
| A_11_P125711 | atlastin GTPase 3 | 2.25 | A_11_P0000024517 | mesoderm induction early response 1, transcriptional regulator | -2.39 |
| A_11_P194483 | family with sequence similarity 188, member B | 2.25 | A_11_P161643 |  | -2.39 |
| A_11_P172628 | protein kinase, AMP-activated, gamma 1 non-catalytic subunit | 2.25 | A_11_P0000025095 | ankyrin repeat domain 12 | -2.39 |
| A_11_P194548 | uncharacterized LOC475115 | 2.25 | A_11_P0000023092 | 5'-nucleotidase, cytosolic II | -2.39 |
| A_11_P0000024479 | RAN guanine nucleotide release factor | 2.25 | A_11_P0000031342 | sosondowah ankyrin repeat domain family member B | -2.39 |
| A_11_P000003086 |  | 2.25 | A_11_P0000031010 | phospholipase A2, group IID | -2.39 |
| A_11_P0000025054 |  | 2.25 | A_11_P076126 | homer homolog 3 (Drosophila) | -2.39 |
| A_11_P141115 |  | 2.25 | A_11_P0000012869 |  | -2.39 |
| A_11_P133191 |  | 2.25 | A_11_P092676 | sec1 family domain containing 1 | -2.39 |
| A_11_P0000022124 | NADH dehydrogenase (ubiquinone) complex I, assembly factor 3 | 2.25 | A_11_P000009056 |  | -2.39 |
| A_11_P0000020337 | NHP2 non-histone chromosome protein 2-like 1 (S. cerevisiae) | 2.25 | A_11_P161538 |  | -2.39 |
| A_11_P00000690 | serglycin | 2.25 | A_11_P169938 |  | -2.39 |
| A_11_P0000030018 | frizzled family receptor 10 | 2.25 | A_11_P202793 | RAR-related orphan receptor A | -2.39 |
| A_11_P126236 |  | 2.25 | A_11_P0000022798 | strawberry notch homolog 1 (Drosophila) | -2.39 |
| A_11_P0000040801 |  | 2.25 | A_11_P211438 |  | -2.39 |
| A_11_P0000015997 | protein kinase domain containing, cytoplasmic | 2.25 | A_11_P0000028869 | elongation factor RNA polymerase II | -2.39 |
| A_11_P145973 |  | 2.25 | A_11_P0000011110 |  | -2.39 |
| A_11_P137036 | lactate dehydrogenase A | 2.25 | A_11_P0000019009 |  | -2.39 |
| A_11_P205303 |  | 2.25 | A_11_P090481 | MAX dimerization protein 1 | -2.39 |
| A_11_P201968 | topoisomerase (DNA) II alpha 170kDa | 2.25 | A_11_P096086 | acetylcholinesterase | -2.39 |
| A_11_P087711 | protease-associated domain containing 1 | 2.25 | A_11_P0000039887 |  | -2.39 |
| A_11_P084456 | ribosomal protein L8 | 2.25 | A_11_P166698 |  | -2.38 |
| A_11_P000002224 | beta-site APP-cleaving enzyme 1 | 2.25 | A_11_P063131 |  | -2.38 |
| A_11_P0000024924 | importin 9 | 2.24 | A_11_P091431 | phospholipase A2, group VII (platelet-activating factor acetylhydrolase, plasma) | -2.38 |
| A_11_P0000034882 |  | 2.24 | A_11_P0000041763 |  | -2.38 |
| A_11_P157933 |  | 2.24 | A_11_P0000012608 |  | -2.38 |
| A_11_P148153 | leucine zipper transcription factor-like 1 | 2.24 | A_11_P0000025045 | S100 calcium binding protein A6 | -2.38 |
| A_11_P0000028947 | ribonuclease H2, subunit A | 2.24 | A_11_P183133 | poly(A)-specific ribonuclease | -2.38 |
| A_11_P186058 | adenosylhomocysteinase | 2.24 | A_11_P0000014907 |  | -2.38 |
| A_11_P212713 | eukaryotic translation elongation factor 2 | 2.24 | A_11_P062166 | sorbitol dehydrogenase | -2.38 |
| A_11_P059271 | synuclein, alpha (non A4 component of amyloid precursor) | 2.24 | A_11_P082986 | ATPase, Ca++ transporting, ubiquitous | -2.38 |
| A_11_P061801 |  | 2.24 | A_11_P084901 | albumin | -2.38 |
| A_11_P0000037267 | StAR-related lipid transfer (START) domain containing 4 | 2.24 | A_11_P0000029469 | phospholipase C, delta 1 | -2.38 |
| A_11_P118331 | peptidylprolyl isomerase A (cyclophilin A) pseudogene | 2.24 | A_11_P056876 |  | -2.38 |
| A_11_P0000032575 | hydroxymethylbilane synthase | 2.24 | A_11_P0000018128 |  | -2.38 |
| A_11_P0000020832 | TatD DNase domain containing 1 | 2.24 | A_11_P0000018362 | desmocollin 2 | -2.38 |
| A_11_P206608 | centromere protein V | 2.24 | A_11_P139746 |  | -2.38 |
| A_11_P0000032831 | mevalonate (diphospho) decarboxylase | 2.24 | A_11_P094626 | Rap guanine nucleotide exchange factor (GEF) 6 | -2.38 |
| A_11_P0000040141 |  | 2.24 | A_11_P061396 | runt-related transcription factor 1 | -2.38 |
| A_11_P065406 | extra spindle pole bodies homolog 1 (S. cerevisiae) | 2.24 | A_11_P0000032752 | family with sequence similarity 151, member A | -2.38 |
| A_11_P056161 | phenylalanyl-tRNA synthetase 2, mitochondrial | 2.24 | A_11_P0000041704 |  | -2.38 |
| A_11_P185613 | gem (nuclear organelle) associated protein 6 | 2.24 | A_11_P175438 |  | -2.37 |
| A_11_P0000025462 | translocase of inner mitochondrial membrane 22 homolog (yeast) | 2.24 | A_11_P0000013788 |  | -2.37 |
| A_11_P088061 | histone H2A type 2-B-like | 2.24 | A_11_P0000038748 |  | -2.37 |
| A_11_P118116 | vaccinia related kinase 1 | 2.24 | A_11_P208293 |  | -2.37 |
| A_11_P164928 | junctional adhesion molecule 3 | 2.24 | A_11_P0000023350 | NEDD4 binding protein 1 | -2.37 |
| A_11_P141170 |  | 2.24 | A_11_P0000010522 |  | -2.37 |
| A_11_P139471 | class II, major histocompatibility complex, transactivator | 2.24 | A_11_P0000020245 | peroxisome proliferator-activated receptor gamma | -2.37 |
| A_11_P070126 | spindle and kinetochore associated complex subunit 3 | 2.24 | A_11_P0000021839 | zinc finger, CCHC domain containing 6 | -2.37 |
| A_11_P0000028065 | polymerase (RNA) II (DNA directed) polypeptide D | 2.23 | A_11_P0000031107 | vacuolar protein sorting 39 homolog (S. cerevisiae) | -2.37 |
| A_11_P0000040455 | NDRG family member 2 | 2.23 | A_11_P0000015564 |  | -2.37 |
| A_11_P0000026893 | solute carrier family 2 (facilitated glucose transporter), member 1 | 2.23 | A_11_P169548 |  | -2.37 |
| A_11_P183768 | solute carrier family 25 (mitochondrial carrier; peroxisomal membrane protein, 34kDa), member 17 | 2.23 | A_11_P0000013152 |  | -2.37 |
| A_11_P063851 | phospholysine phosphohistidine inorganic pyrophosphate phosphatase | 2.23 | A_11_P0000033083 | TAO kinase 2 | -2.37 |
| A_11_P0000027980 | multiple endocrine neoplasia I | 2.23 | A_11_P0000032743 | InaD-like (Drosophila) | -2.37 |
| A_11_P180198 | glycyl-tRNA synthetase | 2.23 | A_11_P0000016112 |  | -2.37 |
| A_11_P0000019917 | phosphofructokinase, muscle | 2.23 | A_11_P089081 | uroplakin 3A | -2.37 |
| A_11_P0000030455 | CD9 molecule | 2.23 | A_11_P177543 | HERPUD family member 2 | -2.37 |
| A_11_P145193 | dual specificity phosphatase 14 | 2.23 | A_11_P0000035253 |  | -2.37 |
| A_11_P079671 |  | 2.23 | A_11_P0000039448 |  | -2.37 |
| A_11_P0000021481 | mitochondrial ribosomal protein L32 | 2.23 | A_11_P218783 | DENN/MADD domain containing 1C | -2.37 |
| A_11_P148478 | ribosome production factor 1 homolog (S. cerevisiae) | 2.23 | A_11_P212863 | deltex 3-like (Drosophila) | -2.37 |
| A_11_P110486 | phosphatidylinositol glycan anchor biosynthesis, class N | 2.23 | A_11_P0000021243 | glutathione reductase | -2.37 |
| A_11_P161778 | ST3 beta-galactoside alpha-2,3-sialyltransferase 6 | 2.23 | A_11_P0000010068 |  | -2.37 |
| A_11_P0000017563 | zinc finger protein 532 | 2.23 | A_11_P0000027798 | olfactory receptor 1052-like | -2.37 |
| A_11_P089591 | minichromosome maintenance complex component 5 | 2.23 | A_11_P192148 | Nipped-B homolog (Drosophila) | -2.37 |
| A_11_P0000026195 | nucleolar protein 6 (RNA-associated) | 2.23 | A_11_P0000014038 | target of myb1 (chicken) | -2.37 |
| A_11_P153718 | mitochondrial ribosomal protein L35 | 2.23 | A_11_P189013 | elongation factor, RNA polymerase II, 2 | -2.36 |
| A_11_P0000014208 |  | 2.23 | A_11_P105066 | solute carrier family 26 (anion exchanger), member 2 | -2.36 |
| A_11_P000002465 |  | 2.23 | A_11_P0000023760 |  | -2.36 |
| A_11_P174308 |  | 2.23 | A_11_P0000039189 | perilipin 2 | -2.36 |
| A_11_P082686 | dehydrogenase/reductase (SDR family) member 13 | 2.23 | A_11_P0000023026 | adiponectin receptor 2 | -2.36 |
| A_11_P158243 | four and a half LIM domains 1 | 2.23 | A_11_P180323 |  | -2.36 |
| A_11_P0000022872 | HORMA domain containing 2 | 2.23 | A_11_P0000041575 |  | -2.36 |
| A_11_P097176 | lipopolysaccharide-induced TNF factor | 2.23 | A_11_P125211 | cation channel, sperm associated 2 | -2.36 |
| A_11_P0000015438 |  | 2.23 | A_11_P0000040216 |  | -2.36 |
| A_11_P0000023705 | CD47 molecule | 2.23 | A_11_P201533 |  | -2.36 |
| A_11_P095036 | protein tyrosine phosphatase-like A domain containing 2 | 2.23 | A_11_P086346 | LPS-responsive vesicle trafficking, beach and anchor containing | -2.36 |
| A_11_P0000028728 |  | 2.23 | A_11_P059436 | tet methylcytosine dioxygenase 2 | -2.36 |
| A_11_P089126 |  | 2.23 | A_11_P063311 | ectonucleoside triphosphate diphosphohydrolase 7 | -2.36 |
| A_11_P106211 | colorectal cancer associated 2 | 2.23 | A_11_P0000021415 |  | -2.36 |
| A_11_P137621 | ALG8, alpha-1,3-glucosyltransferase | 2.23 | A_11_P212193 | microtubule-associated protein 4 | -2.36 |
| A_11_P108031 |  | 2.22 | A_11_P0000023038 |  | -2.36 |
| A_11_P052696 | serine/arginine-rich splicing factor 3 | 2.22 | A_11_P0000012331 |  | -2.36 |
| A_11_P165998 |  | 2.22 | A_11_P0000038965 | Sec23 homolog A (S. cerevisiae) | -2.36 |
| A_11_P0000031203 |  | 2.22 | A_11_P0000028726 | TatD DNase domain containing 2 | -2.36 |
| A_11_P111696 | nuclear factor, interleukin 3 regulated | 2.22 | A_11_P00000784 | family with sequence similarity 190, member B | -2.36 |
| A_11_P092556 |  | 2.22 | A_11_P110161 | CD226 molecule | -2.36 |
| A_11_P075341 | proteasome (prosome, macropain) 26S subunit, non-ATPase, 6 | 2.22 | A_11_P052661 | transporter 1, ATP-binding cassette, sub-family B (MDR/TAP) | -2.36 |
| A_11_P121251 | peptidylprolyl isomerase B (cyclophilin B) | 2.22 | A_11_P124976 |  | -2.36 |
| A_11_P058291 | cms1 ribosomal small subunit homolog (yeast) | 2.22 | A_11_P063431 | golgi brefeldin A resistant guanine nucleotide exchange factor 1 | -2.36 |
| A_11_P055656 | family with sequence similarity 78, member B | 2.22 | A_11_P098656 | 6-phosphofructo-2-kinase/fructose-2,6-biphosphatase 2 | -2.36 |
| A_11_P182863 |  | 2.22 | A_11_P000008842 |  | -2.36 |
| A_11_P0000029975 | TNF receptor-associated factor 3 interacting protein 1 | 2.22 | A_11_P0000021204 | negative regulator of ubiquitin-like proteins 1 | -2.36 |
| A_11_P118131 |  | 2.22 | A_11_P118166 | chromosome 19 open reading frame, human C4orf29 | -2.36 |
| A_11_P0000031872 | 5-aminoimidazole-4-carboxamide ribonucleotide formyltransferase/IMP cyclohydrolase | 2.22 | A_11_P108616 | elongation factor, RNA polymerase II, 2 | -2.36 |
| A_11_P0000019950 | calnexin | 2.22 | A_11_P200548 |  | -2.36 |
| A_11_P136801 | ribosomal protein L15 | 2.22 | A_11_P0000014843 |  | -2.35 |
| A_11_P158208 |  | 2.22 | A_11_P000003703 |  | -2.35 |
| A_11_P0000016837 |  | 2.22 | A_11_P105936 | archain 1 | -2.35 |
| A_11_P106946 | transient receptor potential cation channel, subfamily V, member 2 | 2.22 | A_11_P0000016694 | UBX domain protein 8 | -2.35 |
| A_11_P070796 |  | 2.22 | A_11_P084636 | FRY-like | -2.35 |
| A_11_P114516 |  | 2.22 | A_11_P201433 |  | -2.35 |
| A_11_P0000025803 | sulfite oxidase | 2.22 | A_11_P000001639 |  | -2.35 |
| A_11_P0000021880 | F-box/WD repeat-containing protein 12-like | 2.22 | A_11_P000001570 |  | -2.35 |
| A_11_P117097 |  | 2.22 | A_11_P0000038658 |  | -2.35 |
| A_11_P151798 | lysine (K)-specific demethylase 1B | 2.22 | A_11_P055626 | melanoma inhibitory activity family, member 3 | -2.35 |
| A_11_P076061 | transmembrane protein 42 | 2.22 | A_11_P0000032965 | P450 (cytochrome) oxidoreductase | -2.35 |
| A_11_P0000021978 | translocase of inner mitochondrial membrane 50 homolog (S. cerevisiae) | 2.22 | A_11_P0000027498 | OTU domain containing 7B | -2.35 |
| A_11_P0000035502 |  | 2.22 | A_11_P195528 | lysine (K)-specific demethylase 2A | -2.35 |
| A_11_P0000013962 |  | 2.22 | A_11_P0000019021 |  | -2.35 |
| A_11_P121451 | fibroblast growth factor 14 | 2.22 | A_11_P0000023728 | golgin B1 | -2.34 |
| A_11_P0000033333 | arginyl aminopeptidase (aminopeptidase B) | 2.22 | A_11_P112931 |  | -2.34 |
| A_11_P162178 |  | 2.22 | A_11_P160098 | calcium activated nucleotidase 1 | -2.34 |
| A_11_P0000024596 | ER membrane protein complex subunit 8 | 2.22 | A_11_P0000015828 | OTU domain containing 4 | -2.34 |
| A_11_P127788 | chaperonin containing TCP1, subunit 6A (zeta 1) | 2.22 | A_11_P200648 | phospholipase A2, group XVI-like | -2.34 |
| A_11_P0000020517 | eukaryotic translation termination factor 1 | 2.21 | A_11_P084211 | PHD finger protein 20-like 1 | -2.34 |
| A_11_P0000024571 | parkinson protein 7 | 2.21 | A_11_P0000036539 |  | -2.34 |
| A_11_P069811 | cytoskeleton associated protein 2 | 2.21 | A_11_P0000039362 | junction plakoglobin | -2.34 |
| A_11_P078281 | BRF2, RNA polymerase III transcription initiation factor 50 kDa subunit | 2.21 | A_11_P0000025379 | src kinase associated phosphoprotein 1 | -2.34 |
| A_11_P0000031476 | follistatin-like 1 | 2.21 | A_11_P0000038916 |  | -2.34 |
| A_11_P052676 | HLA class II histocompatibility antigen, DO alpha chain-like | 2.21 | A_11_P069456 | SET binding factor 2 | -2.34 |
| A_11_P0000023873 | proteasome (prosome, macropain) 26S subunit, non-ATPase, 14 | 2.21 | A_11_P168028 |  | -2.34 |
| A_11_P0000020899 | UTP3, small subunit (SSU) processome component, homolog (S. cerevisiae) | 2.21 | A_11_P0000027051 | apoptotic peptidase activating factor 1 | -2.34 |
| A_11_P148428 | zinc finger protein 555 | 2.21 | A_11_P0000032151 | solute carrier family 34 (type II sodium/phosphate contransporter), member 2 | -2.34 |
| A_11_P089516 | thiosulfate sulfurtransferase (rhodanese) | 2.21 | A_11_P060081 |  | -2.34 |
| A_11_P190393 |  | 2.21 | A_11_P119811 |  | -2.34 |
| A_11_P0000023190 | carbonic anhydrase II | 2.21 | A_11_P000008147 |  | -2.34 |
| A_11_P093851 | vaccinia related kinase 1 | 2.21 | A_11_P077111 | cAMP responsive element binding protein 3-like 3 | -2.34 |
| A_11_P107711 | parkinson protein 7 | 2.21 | A_11_P0000021264 | FAT atypical cadherin 1 | -2.34 |
| A_11_P0000022021 | monoglyceride lipase | 2.21 | A_11_P147813 | ATPase, Cu++ transporting, alpha polypeptide | -2.34 |
| A_11_P0000025639 | eukaryotic translation initiation factor 2, subunit 3 gamma, 52kDa | 2.21 | A_11_P000008224 |  | -2.34 |
| A_11_P182873 | CD151 molecule (Raph blood group) | 2.21 | A_11_P192858 | ring finger protein 19B | -2.33 |
| A_11_P0000022633 | DEAD (Asp-Glu-Ala-Asp) box polypeptide 27 | 2.21 | A_11_P052606 | spectrin, beta, non-erythrocytic 1 | -2.33 |
| A_11_P203538 | ribonuclease, RNase A family, 4 | 2.21 | A_11_P000006618 |  | -2.33 |
| A_11_P078906 | tetraspanin 33 | 2.21 | A_11_P155328 | family with sequence similarity 190, member B | -2.33 |
| A_11_P0000023095 | oligonucleotide/oligosaccharide-binding fold containing 1 | 2.21 | A_11_P0000033043 | septin 1 | -2.33 |
| A_11_P108341 |  | 2.21 | A_11_P0000012201 |  | -2.33 |
| A_11_P082101 | UTP18 small subunit (SSU) processome component homolog (yeast) | 2.21 | A_11_P0000013931 | transducin-like enhancer of split 3 (E(sp1) homolog, Drosophila) | -2.33 |
| A_11_P0000023521 | cyclin B2 | 2.21 | A_11_P0000038699 | kinesin family member 16B | -2.33 |
| A_11_P090046 | S1 RNA binding domain 1 | 2.21 | A_11_P200658 |  | -2.33 |
| A_11_P116626 | proliferating cell nuclear antigen | 2.21 | A_11_P115936 | G protein-coupled receptor 112 | -2.33 |
| A_11_P060311 |  | 2.21 | A_11_P052081 | carnitine palmitoyltransferase 1A (liver) | -2.33 |
| A_11_P060451 | smoothelin | 2.21 | A_11_P00000529 | ring finger protein 19B | -2.33 |
| A_11_P122271 | forkhead box M1 | 2.21 | A_11_P094621 | Rap guanine nucleotide exchange factor (GEF) 6 | -2.33 |
| A_11_P0000025595 |  | 2.21 | A_11_P115761 | zinc finger protein 280C | -2.33 |
| A_11_P0000028866 | kelch-like family member 26 | 2.21 | A_11_P0000025281 |  | -2.33 |
| A_11_P072501 | minichromosome maintenance complex component 6 | 2.20 | A_11_P000002594 |  | -2.33 |
| A_11_P193588 |  | 2.20 | A_11_P198323 |  | -2.33 |
| A_11_P089771 |  | 2.20 | A_11_P0000012822 |  | -2.33 |
| A_11_P0000015129 |  | 2.20 | A_11_P098801 |  | -2.33 |
| A_11_P103296 | stathmin 1 | 2.20 | A_11_P206578 |  | -2.33 |
| A_11_P0000020064 | hypoxanthine phosphoribosyltransferase 1 | 2.20 | A_11_P102311 | Rho GTPase activating protein 26 | -2.33 |
| A_11_P087776 |  | 2.20 | A_11_P132276 | ataxin 1 | -2.33 |
| A_11_P195013 |  | 2.20 | A_11_P00000149 | spectrin, beta, non-erythrocytic 1 | -2.33 |
| A_11_P113956 |  | 2.20 | A_11_P000004206 |  | -2.33 |
| A_11_P0000020849 | uncharacterized LOC475115 | 2.20 | A_11_P055366 | zinc finger, BED-type containing 6 | -2.33 |
| A_11_P191153 | DnaJ (Hsp40) homolog, subfamily C, member 10 | 2.20 | A_11_P000007975 |  | -2.33 |
| A_11_P165628 | TGFB-induced factor homeobox 2 | 2.20 | A_11_P000004061 |  | -2.33 |
| A_11_P0000021405 | mitochondrial ribosomal protein L19 | 2.20 | A_11_P194393 | acyl-CoA synthetase long-chain family member 5 | -2.33 |
| A_11_P161513 | ATP-binding cassette, sub-family F (GCN20), member 1 | 2.20 | A_11_P099961 |  | -2.33 |
| A_11_P0000020376 | PWP1 homolog (S. cerevisiae) | 2.20 | A_11_P050146 | glucose-6-phosphatase, catalytic subunit | -2.32 |
| A_11_P0000024783 |  | 2.20 | A_11_P050186 | solute carrier family 6 (neurotransmitter transporter, serotonin), member 4 | -2.32 |
| A_11_P171243 | inositol-trisphosphate 3-kinase A | 2.20 | A_11_P000008360 |  | -2.32 |
| A_11_P078966 | staphylococcal nuclease and tudor domain containing 1 | 2.20 | A_11_P0000029542 | protein phosphatase 2, regulatory subunit B'', alpha | -2.32 |
| A_11_P122951 |  | 2.20 | A_11_P0000028597 | zinc finger protein 36, C3H type, homolog (mouse) | -2.32 |
| A_11_P00000461 | chromosome 26 open reading frame, human C12orf52 | 2.20 | A_11_P058461 | solute carrier family 35, member A5 | -2.32 |
| A_11_P163343 |  | 2.20 | A_11_P092801 | Sec23 homolog A (S. cerevisiae) | -2.32 |
| A_11_P0000032751 | prolyl-tRNA synthetase 2, mitochondrial (putative) | 2.20 | A_11_P0000030406 | epithelial membrane protein 1 | -2.32 |
| A_11_P0000028099 | glycosyltransferase-like domain containing 1 | 2.20 | A_11_P0000022017 | aldehyde dehydrogenase 1 family, member L1 | -2.32 |
| A_11_P215188 | nucleolar complex associated 2 homolog (S. cerevisiae) | 2.20 | A_11_P180878 | autophagy related 2A | -2.32 |
| A_11_P0000031669 |  | 2.20 | A_11_P0000029131 |  | -2.32 |
| A_11_P0000024693 | COP9 signalosome subunit 6 | 2.20 | A_11_P0000015460 |  | -2.32 |
| A_11_P0000026427 | glutathione S-transferase A4-like | 2.20 | A_11_P0000021344 | yippee-like 5 (Drosophila) | -2.32 |
| A_11_P149278 | lysine (K)-specific demethylase 1A | 2.20 | A_11_P0000024018 | ethanolamine kinase 2 | -2.32 |
| A_11_P083231 | surfeit 1 | 2.20 | A_11_P126421 | tetratricopeptide repeat, ankyrin repeat and coiled-coil containing 1 | -2.32 |
| A_11_P0000022679 | replication factor C (activator 1) 3, 38kDa | 2.20 | A_11_P0000028479 | orphan sodium- and chloride-dependent neurotransmitter transporter NTT5-like | -2.32 |
| A_11_P055781 | Purkinje cell protein 4 like 1 | 2.20 | A_11_P0000017556 |  | -2.32 |
| A_11_P105476 |  | 2.19 | A_11_P116436 | tetratricopeptide repeat domain 19 | -2.32 |
| A_11_P085341 | nardilysin (N-arginine dibasic convertase) | 2.19 | A_11_P215588 |  | -2.32 |
| A_11_P0000034697 | moesin | 2.19 | A_11_P121186 | paraneoplastic Ma antigen 1 | -2.32 |
| A_11_P0000027872 | synaptotagmin XIII | 2.19 | A_11_P0000033256 | solute carrier family 25 (mitochondrial carrier; phosphate carrier), member 24 | -2.32 |
| A_11_P0000038888 | lactate dehydrogenase A | 2.19 | A_11_P0000024689 |  | -2.32 |
| A_11_P099211 |  | 2.19 | A_11_P106161 | DIX domain containing 1 | -2.32 |
| A_11_P0000039162 | immunoglobulin J polypeptide, linker protein for immunoglobulin alpha and mu polypeptides | 2.19 | A_11_P088726 |  | -2.32 |
| A_11_P213013 |  | 2.19 | A_11_P050976 | cathepsin A | -2.32 |
| A_11_P0000032624 | aminoadipate-semialdehyde dehydrogenase-phosphopantetheinyl transferase | 2.19 | A_11_P124816 |  | -2.32 |
| A_11_P087486 | mitochondrial ribosomal protein L35 | 2.19 | A_11_P00000766 | mitogen-activated protein kinase 6 | -2.31 |
| A_11_P059786 | cyclin-dependent kinase 2 associated protein 1 | 2.19 | A_11_P141105 |  | -2.31 |
| A_11_P0000024960 | chromosome 7 open reading frame, human C1orf27 | 2.19 | A_11_P106381 | spinster homolog 3 (Drosophila) | -2.31 |
| A_11_P147108 |  | 2.19 | A_11_P194208 | tumor necrosis factor receptor superfamily, member 1B | -2.31 |
| A_11_P0000021028 | guanine nucleotide binding protein-like 2 (nucleolar) | 2.19 | A_11_P0000015168 |  | -2.31 |
| A_11_P059726 |  | 2.19 | A_11_P0000022786 | unc-51 like autophagy activating kinase 1 | -2.31 |
| A_11_P185518 | ets homologous factor | 2.19 | A_11_P077456 | mucosal vascular addressin cell adhesion molecule 1 | -2.31 |
| A_11_P0000016757 | betaine--homocysteine S-methyltransferase 2 | 2.19 | A_11_P000002402 |  | -2.31 |
| A_11_P0000019896 | adenosine A3 receptor | 2.19 | A_11_P133921 |  | -2.31 |
| A_11_P0000039653 |  | 2.19 | A_11_P0000011863 |  | -2.31 |
| A_11_P0000023370 |  | 2.19 | A_11_P171333 |  | -2.31 |
| A_11_P109981 | C-terminal-binding protein 1-like | 2.19 | A_11_P110521 | headcase homolog (Drosophila) | -2.31 |
| A_11_P052526 | glutaminyl-peptide cyclotransferase | 2.19 | A_11_P071616 | acyl-CoA dehydrogenase family, member 11 | -2.31 |
| A_11_P107186 | phosphoglucomutase 1 | 2.19 | A_11_P178478 | titin-cap | -2.31 |
| A_11_P100001 | zinc finger protein 397 | 2.19 | A_11_P0000032202 | LysM, putative peptidoglycan-binding, domain containing 3 | -2.31 |
| A_11_P075266 | SHQ1, H/ACA ribonucleoprotein assembly factor | 2.19 | A_11_P068461 | folate hydrolase (prostate-specific membrane antigen) 1 | -2.31 |
| A_11_P0000032452 | serine/threonine-protein kinase NIM1 | 2.19 | A_11_P0000023684 | SEC24 family, member D (S. cerevisiae) | -2.31 |
| A_11_P0000024714 | FtsJ RNA methyltransferase homolog 2 (E. coli) | 2.19 | A_11_P0000013648 |  | -2.31 |
| A_11_P0000018103 |  | 2.19 | A_11_P089976 | ankyrin repeat domain 23 | -2.31 |
| A_11_P0000016462 | solute carrier family 25 (mitochondrial carrier, brain), member 14 | 2.19 | A_11_P163628 |  | -2.31 |
| A_11_P0000030965 | nudC nuclear distribution protein | 2.19 | A_11_P0000040214 |  | -2.31 |
| A_11_P0000014780 |  | 2.19 | A_11_P0000033812 | KIAA1737 ortholog | -2.31 |
| A_11_P0000021381 | polymerase (RNA) I polypeptide B, 128kDa | 2.19 | A_11_P0000020589 |  | -2.31 |
| A_11_P119156 |  | 2.19 | A_11_P0000033328 | protein phosphatase 1, regulatory subunit 12B | -2.31 |
| A_11_P094771 |  | 2.19 | A_11_P199933 |  | -2.31 |
| A_11_P074506 | vascular endothelial growth factor B | 2.19 | A_11_P0000041497 |  | -2.31 |
| A_11_P184783 | asparaginyl-tRNA synthetase | 2.19 | A_11_P0000026571 | metastasis suppressor 1 | -2.31 |
| A_11_P085531 | nuclear autoantigenic sperm protein (histone-binding) | 2.18 | A_11_P116754 | programmed cell death 7 | -2.31 |
| A_11_P0000025571 | calcium binding tyrosine-(Y)-phosphorylation regulated | 2.18 | A_11_P096321 | lemur tyrosine kinase 2 | -2.31 |
| A_11_P050076 | dynein, light chain, Tctex-type 3 | 2.18 | A_11_P118006 |  | -2.31 |
| A_11_P0000030202 | solute carrier family 7, member 4 | 2.18 | A_11_P059881 |  | -2.30 |
| A_11_P101836 |  | 2.18 | A_11_P0000040841 | solute carrier family 35 (UDP-GlcNAc/UDP-glucose transporter), member D2 | -2.30 |
| A_11_P092881 | polymerase (DNA directed), epsilon 2, accessory subunit | 2.18 | A_11_P0000033130 | phospholipase A2, group X | -2.30 |
| A_11_P0000022027 | coiled-coil-helix-coiled-coil-helix domain containing 4 | 2.18 | A_11_P116066 | myotubularin 1 | -2.30 |
| A_11_P0000020638 |  | 2.18 | A_11_P0000012612 |  | -2.30 |
| A_11_P118031 | cat eye syndrome chromosome region, candidate 5 | 2.18 | A_11_P0000041951 |  | -2.30 |
| A_11_P121816 | asparaginyl-tRNA synthetase | 2.18 | A_11_P000002491 |  | -2.30 |
| A_11_P0000020037 | Sec61 beta subunit | 2.18 | A_11_P0000026502 | lin-28 homolog B (C. elegans) | -2.30 |
| A_11_P0000031777 | golgi reassembly stacking protein 2, 55kDa | 2.18 | A_11_P0000026380 | trem-like transcript 2 protein-like | -2.30 |
| A_11_P0000039245 | nucleolar and spindle associated protein 1 | 2.18 | A_11_P0000034065 | tripartite motif containing 25 | -2.30 |
| A_11_P195043 | chromosome 7 open reading frame, human C1orf85 | 2.18 | A_11_P0000030970 | uncharacterized LOC612166 | -2.30 |
| A_11_P000003991 | neuronal regeneration related protein | 2.18 | A_11_P0000035199 |  | -2.30 |
| A_11_P000002652 | signal sequence receptor, delta | 2.18 | A_11_P000007479 |  | -2.30 |
| A_11_P0000040611 | NDRG family member 4 | 2.18 | A_11_P170673 | junction plakoglobin | -2.30 |
| A_11_P0000033407 | torsin family 3, member A | 2.18 | A_11_P0000011907 |  | -2.30 |
| A_11_P208278 |  | 2.18 | A_11_P193813 |  | -2.30 |
| A_11_P086046 | methionyl aminopeptidase 2 | 2.18 | A_11_P0000029560 | plastin 1 | -2.30 |
| A_11_P158918 | polymerase (RNA) III (DNA directed) polypeptide F, 39 kDa | 2.18 | A_11_P0000012090 |  | -2.30 |
| A_11_P063221 | exosome component 1 | 2.18 | A_11_P178373 |  | -2.30 |
| A_11_P0000021316 |  | 2.18 | A_11_P119761 |  | -2.30 |
| A_11_P0000025067 | nascent polypeptide-associated complex alpha subunit | 2.18 | A_11_P212308 | SMAD family member 2 | -2.30 |
| A_11_P076146 |  | 2.18 | A_11_P0000040132 |  | -2.30 |
| A_11_P0000025664 | ubiquitously-expressed, prefoldin-like chaperone | 2.18 | A_11_P115441 | RNA binding motif protein 41 | -2.29 |
| A_11_P000003298 |  | 2.18 | A_11_P164808 |  | -2.29 |
| A_11_P0000033931 | glucose 6 phosphatase, catalytic, 3 | 2.18 | A_11_P00000580 | myotubularin related protein 11 | -2.29 |
| A_11_P111596 | very low density lipoprotein receptor | 2.18 | A_11_P0000037211 |  | -2.29 |
| A_11_P0000037236 |  | 2.18 | A_11_P0000013012 |  | -2.29 |
| A_11_P0000022937 | peripherin | 2.18 | A_11_P000009326 |  | -2.29 |
| A_11_P051306 | non-histone chromosomal protein HMG-17 | 2.18 | A_11_P0000011233 |  | -2.29 |
| A_11_P0000028970 | transmembrane emp24 protein transport domain containing 1 | 2.18 | A_11_P050111 | cingulin | -2.29 |
| A_11_P000006061 | EP300 interacting inhibitor of differentiation 1 | 2.18 | A_11_P102936 | NEDD4 binding protein 1 | -2.29 |
| A_11_P099681 | thrombospondin 3 | 2.17 | A_11_P107751 | phosphatidylinositol-4,5-bisphosphate 3-kinase, catalytic subunit delta | -2.29 |
| A_11_P173688 | receptor tyrosine kinase-like orphan receptor 2 | 2.17 | A_11_P0000033749 | zinc finger and BTB domain containing 25 | -2.29 |
| A_11_P118816 |  | 2.17 | A_11_P084246 | collagen, type XXII, alpha 1 | -2.29 |
| A_11_P0000023833 | eukaryotic translation elongation factor 1 epsilon 1 | 2.17 | A_11_P000004748 |  | -2.29 |
| A_11_P000004462 | nuclear transport factor 2-like export factor 2 | 2.17 | A_11_P0000011734 |  | -2.29 |
| A_11_P0000023936 | collagen, type III, alpha 1 | 2.17 | A_11_P080141 | Kruppel-like factor 12 | -2.29 |
| A_11_P0000028876 |  | 2.17 | A_11_P0000016505 | mitogen-activated protein kinase kinase 3 | -2.29 |
| A_11_P198123 | retinol binding protein 3, interstitial | 2.17 | A_11_P055356 | ATPase, Ca++ transporting, plasma membrane 4 | -2.29 |
| A_11_P0000028026 | coiled-coil domain containing 86 | 2.17 | A_11_P070626 | ArfGAP with FG repeats 1 | -2.29 |
| A_11_P0000020026 | signal peptidase complex subunit 3 homolog (S. cerevisiae) | 2.17 | A_11_P0000027889 | interferon induced transmembrane protein 10 | -2.29 |
| A_11_P0000031572 | EPH receptor B3 | 2.17 | A_11_P0000023938 | solute carrier family 40 (iron-regulated transporter), member 1 | -2.29 |
| A_11_P0000017280 | replication initiator 1 | 2.17 | A_11_P138401 | G protein-coupled receptor 107 | -2.29 |
| A_11_P0000016788 | uncharacterized LOC485710 | 2.17 | A_11_P0000013973 |  | -2.29 |
| A_11_P0000029023 | translocase of inner mitochondrial membrane 44 homolog (yeast) | 2.17 | A_11_P165918 |  | -2.29 |
| A_11_P186753 |  | 2.17 | A_11_P115736 | X-prolyl aminopeptidase (aminopeptidase P) 2, membrane-bound | -2.29 |
| A_11_P122286 | replication factor C (activator 1) 5, 36.5kDa | 2.17 | A_11_P0000028207 | transmembrane protein 181 | -2.29 |
| A_11_P078186 | GINS complex subunit 4 (Sld5 homolog) | 2.17 | A_11_P050226 | lipase, gastric | -2.29 |
| A_11_P151793 |  | 2.17 | A_11_P0000028594 | dual-specificity tyrosine-(Y)-phosphorylation regulated kinase 1B | -2.29 |
| A_11_P086256 | ATP-binding cassette, sub-family E (OABP), member 1 | 2.17 | A_11_P0000016806 | FERM domain containing 8 | -2.29 |
| A_11_P179043 |  | 2.17 | A_11_P206473 |  | -2.29 |
| A_11_P209708 |  | 2.17 | A_11_P214828 |  | -2.29 |
| A_11_P0000015553 |  | 2.17 | A_11_P0000021327 | abhydrolase domain containing 1 | -2.29 |
| A_11_P0000011111 | microtubule-associated protein 2 | 2.17 | A_11_P055341 | BTG family, member 2 | -2.29 |
| A_11_P000009928 |  | 2.17 | A_11_P117651 | pyruvate dehydrogenase kinase, isozyme 4 | -2.29 |
| A_11_P081551 | NFKB inhibitor interacting Ras-like 2 | 2.17 | A_11_P0000020976 | chromosome 14 open reading frame, human C7orf31 | -2.29 |
| A_11_P179063 | RAN binding protein 1 | 2.17 | A_11_P073271 | protein associated with topoisomerase II homolog 1 (yeast) | -2.29 |
| A_11_P0000015504 |  | 2.17 | A_11_P140466 | aquaporin 3 (Gill blood group) | -2.29 |
| A_11_P110201 | serpin peptidase inhibitor, clade B (ovalbumin), member 5 | 2.17 | A_11_P125656 |  | -2.29 |
| A_11_P064826 | methylcrotonoyl-CoA carboxylase 1 (alpha) | 2.17 | A_11_P065111 |  | -2.28 |
| A_11_P0000023797 | replication factor C (activator 1) 4, 37kDa | 2.17 | A_11_P000008536 |  | -2.28 |
| A_11_P095041 | protein tyrosine phosphatase-like A domain containing 2 | 2.17 | A_11_P173733 | family with sequence similarity 46, member B | -2.28 |
| A_11_P0000039777 | proteasome (prosome, macropain) assembly chaperone 4 | 2.17 | A_11_P171478 | unc-93 homolog B1 (C. elegans) | -2.28 |
| A_11_P122591 | transcription elongation factor A (SII)-like 8 | 2.17 | A_11_P0000018222 |  | -2.28 |
| A_11_P0000021550 | APAF1 interacting protein | 2.17 | A_11_P181893 | 6-phosphofructo-2-kinase/fructose-2,6-biphosphatase 3 | -2.28 |
| A_11_P0000015535 | kallikrein 1 | 2.17 | A_11_P0000027137 | EPH receptor A1 | -2.28 |
| A_11_P0000020562 | natriuretic peptide receptor B/guanylate cyclase B (atrionatriuretic peptide receptor B) | 2.17 | A_11_P215898 |  | -2.28 |
| A_11_P208768 |  | 2.17 | A_11_P144033 |  | -2.28 |
| A_11_P178168 | eukaryotic translation elongation factor 1 alpha 1 | 2.17 | A_11_P0000010969 |  | -2.28 |
| A_11_P158508 | calcitonin receptor-like | 2.17 | A_11_P0000033585 | v-yes-1 Yamaguchi sarcoma viral oncogene homolog 1 | -2.28 |
| A_11_P0000025437 | zinc finger protein 207 | 2.16 | A_11_P00000302 |  | -2.28 |
| A_11_P0000016204 |  | 2.16 | A_11_P0000024937 | solute carrier family 30 (zinc transporter), member 1 | -2.28 |
| A_11_P000005893 | general transcription factor IIi | 2.16 | A_11_P00000358 | phosphatidic acid phosphatase type 2A | -2.28 |
| A_11_P093661 | tyrosyl-DNA phosphodiesterase 1 | 2.16 | A_11_P000009555 |  | -2.28 |
| A_11_P0000024815 | RNA binding protein S1, serine-rich domain | 2.16 | A_11_P000006439 |  | -2.28 |
| A_11_P196703 |  | 2.16 | A_11_P085256 |  | -2.28 |
| A_11_P0000026605 | rhophilin, Rho GTPase binding protein 1 | 2.16 | A_11_P0000033891 | guanine nucleotide binding protein (G protein), alpha 13 | -2.28 |
| A_11_P149018 | ribosomal protein L15 | 2.16 | A_11_P0000029451 | cystatin F (leukocystatin) | -2.28 |
| A_11_P0000038974 |  | 2.16 | A_11_P0000020737 | pleckstrin homology domain interacting protein | -2.28 |
| A_11_P0000020242 | MHC class Ib | 2.16 | A_11_P0000014111 |  | -2.28 |
| A_11_P110934 | suppression of tumorigenicity 13 (colon carcinoma) (Hsp70 interacting protein) | 2.16 | A_11_P0000041936 |  | -2.28 |
| A_11_P0000034942 | dyskeratosis congenita 1, dyskerin | 2.16 | A_11_P0000027965 | tigger transposable element derived 3 | -2.28 |
| A_11_P128286 | oligosaccharyltransferase complex subunit | 2.16 | A_11_P094081 | cysteine-rich protein 1 (intestinal) | -2.28 |
| A_11_P089061 | wingless-type MMTV integration site family, member 7B | 2.16 | A_11_P050571 | phosphodiesterase 6B, cGMP-specific, rod, beta | -2.28 |
| A_11_P0000021815 | SPARC related modular calcium binding 2 | 2.16 | A_11_P0000021775 | interferon gamma receptor 1 | -2.28 |
| A_11_P181463 | ATP-binding cassette, sub-family F (GCN20), member 1 | 2.16 | A_11_P063616 | MAX interactor 1, dimerization protein | -2.28 |
| A_11_P0000030200 | kelch-like family member 22 | 2.16 | A_11_P0000029479 | cysteine-serine-rich nuclear protein 1 | -2.28 |
| A_11_P109421 | DnaJ (Hsp40) homolog, subfamily A, member 4 | 2.16 | A_11_P0000013187 |  | -2.28 |
| A_11_P0000039456 | cyclin-dependent kinase 1 | 2.16 | A_11_P082276 | protein phosphatase, Mg2+/Mn2+ dependent, 1E | -2.28 |
| A_11_P0000017797 |  | 2.16 | A_11_P098611 | zinc finger and BTB domain containing 41 | -2.27 |
| A_11_P070146 | crystallin, lambda 1 | 2.16 | A_11_P0000033852 | B-cell CLL/lymphoma 11B (zinc finger protein) | -2.27 |
| A_11_P0000022106 | nitrogen permease regulator-like 2 (S. cerevisiae) | 2.16 | A_11_P000002476 |  | -2.27 |
| A_11_P0000013927 |  | 2.16 | A_11_P0000026887 | solute carrier family 13 (sodium/sulfate symporter), member 1 | -2.27 |
| A_11_P086846 |  | 2.16 | A_11_P192093 | inositol hexakisphosphate kinase 1 | -2.27 |
| A_11_P0000038820 |  | 2.16 | A_11_P000009294 |  | -2.27 |
| A_11_P164588 | ribosomal protein L15 | 2.16 | A_11_P0000018796 |  | -2.27 |
| A_11_P151083 | ornithine aminotransferase | 2.16 | A_11_P168483 |  | -2.27 |
| A_11_P075521 |  | 2.15 | A_11_P091041 |  | -2.27 |
| A_11_P0000016683 |  | 2.15 | A_11_P0000041153 |  | -2.27 |
| A_11_P063226 | exosome component 1 | 2.15 | A_11_P087506 |  | -2.27 |
| A_11_P0000032617 | KDEL (Lys-Asp-Glu-Leu) containing 2 | 2.15 | A_11_P084941 | amphiregulin-like | -2.27 |
| A_11_P155598 |  | 2.15 | A_11_P161143 |  | -2.27 |
| A_11_P0000025076 | small nuclear ribonucleoprotein D1 polypeptide 16kDa | 2.15 | A_11_P0000031283 | MORC family CW-type zinc finger 3 | -2.27 |
| A_11_P073366 |  | 2.15 | A_11_P0000039198 | FK506 binding protein 5 | -2.27 |
| A_11_P0000032546 | von Willebrand factor A domain containing 5A | 2.15 | A_11_P0000041471 |  | -2.27 |
| A_11_P0000040042 |  | 2.15 | A_11_P0000028312 | KIAA1432 ortholog | -2.27 |
| A_11_P00000752 | ER membrane protein complex subunit 1 | 2.15 | A_11_P0000027421 |  | -2.27 |
| A_11_P206278 | thioredoxin-like 4A | 2.15 | A_11_P188233 | transducer of ERBB2, 1 | -2.27 |
| A_11_P123663 | spermidine synthase | 2.15 | A_11_P00000600 | SEC22 vesicle trafficking protein homolog A (S. cerevisiae) | -2.27 |
| A_11_P0000039236 | protocadherin alpha 5 | 2.15 | A_11_P0000011902 |  | -2.27 |
| A_11_P0000040500 | aldehyde dehydrogenase 2 family (mitochondrial) | 2.15 | A_11_P059996 |  | -2.27 |
| A_11_P078831 | coiled-coil-helix-coiled-coil-helix domain containing 3 | 2.15 | A_11_P174208 | membrane-associated ring finger (C3HC4) 7, E3 ubiquitin protein ligase | -2.27 |
| A_11_P00000251 | basal cell adhesion molecule (Lutheran blood group) | 2.15 | A_11_P110821 | solute carrier family 22 (organic cation transporter), member 3 | -2.27 |
| A_11_P0000023172 | COP9 signalosome subunit 5 | 2.15 | A_11_P0000023554 |  | -2.27 |
| A_11_P217268 |  | 2.15 | A_11_P090056 | protein kinase C, epsilon | -2.27 |
| A_11_P174163 |  | 2.15 | A_11_P192654 |  | -2.27 |
| A_11_P0000026978 | UDP-Gal:betaGlcNAc beta 1,4- galactosyltransferase, polypeptide 2 | 2.15 | A_11_P0000041757 |  | -2.27 |
| A_11_P149958 | solute carrier family 6 (neurotransmitter transporter), member 1 | 2.15 | A_11_P0000010531 |  | -2.27 |
| A_11_P177828 | MIS18 kinetochore protein homolog A (S. pombe) | 2.15 | A_11_P166868 | KIAA0430 ortholog | -2.27 |
| A_11_P173868 |  | 2.15 | A_11_P0000013682 |  | -2.27 |
| A_11_P0000023768 | centrosomal protein 72kDa | 2.15 | A_11_P169603 | zinc finger, CCHC domain containing 6 | -2.26 |
| A_11_P0000035021 | protease-associated domain containing 1 | 2.15 | A_11_P0000041252 |  | -2.26 |
| A_11_P091531 | glutathione S-transferase A4-like | 2.15 | A_11_P0000016414 |  | -2.26 |
| A_11_P0000018263 | N-myc downstream regulated 1 | 2.15 | A_11_P092896 |  | -2.26 |
| A_11_P184113 | DNA cross-link repair 1B | 2.15 | A_11_P0000015768 | calcium binding and coiled-coil domain 1 | -2.26 |
| A_11_P0000033681 | cofilin 2 (muscle) | 2.15 | A_11_P0000010213 |  | -2.26 |
| A_11_P068771 | phosphodiesterase 2A, cGMP-stimulated | 2.14 | A_11_P0000025266 | tetratricopeptide repeat domain 7B | -2.26 |
| A_11_P0000023993 |  | 2.14 | A_11_P0000018254 |  | -2.26 |
| A_11_P000004546 | adducin 3 (gamma) | 2.14 | A_11_P0000031576 | lipase, member H | -2.26 |
| A_11_P0000023139 | ornithine aminotransferase | 2.14 | A_11_P099851 |  | -2.26 |
| A_11_P0000034081 | chromosome 9 open reading frame, human C17orf64 | 2.14 | A_11_P058026 | tubulin tyrosine ligase-like family, member 4 | -2.26 |
| A_11_P0000019978 | signal recognition particle 72kDa | 2.14 | A_11_P181298 |  | -2.26 |
| A_11_P0000038936 |  | 2.14 | A_11_P210458 |  | -2.26 |
| A_11_P0000034007 | polycomb group ring finger 2 | 2.14 | A_11_P0000026261 | Kruppel-like factor 4 (gut) | -2.26 |
| A_11_P061966 | Opa interacting protein 5 | 2.14 | A_11_P000006770 |  | -2.26 |
| A_11_P158290 | TBC1 domain family, member 7 | 2.14 | A_11_P155798 |  | -2.26 |
| A_11_P0000015251 |  | 2.14 | A_11_P093956 | CDC42 binding protein kinase beta (DMPK-like) | -2.26 |
| A_11_P00000853 | early B-cell factor 1 | 2.14 | A_11_P0000025402 | myotubularin related protein 4 | -2.26 |
| A_11_P0000016981 | CTP synthase | 2.14 | A_11_P169303 |  | -2.26 |
| A_11_P0000040349 |  | 2.14 | A_11_P0000021720 |  | -2.26 |
| A_11_P00000955 | LSM7 homolog, U6 small nuclear RNA associated (S. cerevisiae) | 2.14 | A_11_P0000023483 | beta-2-microglobulin | -2.26 |
| A_11_P110716 |  | 2.14 | A_11_P184173 | glycerophosphodiester phosphodiesterase domain containing 2 | -2.25 |
| A_11_P073206 | glutamine and serine rich 1 | 2.14 | A_11_P0000017753 |  | -2.25 |
| A_11_P123036 | mitochondrial ribosomal protein S9 | 2.14 | A_11_P093156 | A kinase (PRKA) anchor protein 5 | -2.25 |
| A_11_P0000023336 | nucleoporin 93kDa | 2.14 | A_11_P105096 |  | -2.25 |
| A_11_P140201 | ubiquitin-conjugating enzyme E2-24 kDa-like | 2.14 | A_11_P0000015753 |  | -2.25 |
| A_11_P120981 | growth associated protein 43 | 2.14 | A_11_P159913 |  | -2.25 |
| A_11_P0000025162 | protein phosphatase 2, regulatory subunit B'', gamma | 2.14 | A_11_P218263 |  | -2.25 |
| A_11_P0000039386 | ankyrin repeat domain 29 | 2.14 | A_11_P0000013824 |  | -2.25 |
| A_11_P094256 | butyrophilin-like 9 | 2.14 | A_11_P0000034556 | shroom family member 2 | -2.25 |
| A_11_P0000041180 |  | 2.14 | A_11_P159988 |  | -2.25 |
| A_11_P0000023635 | Rho GTPase activating protein 24 | 2.14 | A_11_P0000025966 | PR domain containing 4 | -2.25 |
| A_11_P110191 | serpin peptidase inhibitor, clade B (ovalbumin), member 8 | 2.14 | A_11_P0000028408 | EPS8-like 1 | -2.25 |
| A_11_P0000039802 |  | 2.14 | A_11_P0000041698 | CD28 molecule | -2.25 |
| A_11_P0000016435 |  | 2.14 | A_11_P090646 | lymphotoxin beta (TNF superfamily, member 3) | -2.25 |
| A_11_P104911 | mitochondrial ribosomal protein L22 | 2.14 | A_11_P000008188 |  | -2.25 |
| A_11_P191653 | copper metabolism (Murr1) domain containing 1 | 2.14 | A_11_P179658 | methyltransferase like 14 | -2.25 |
| A_11_P0000039092 |  | 2.14 | A_11_P0000012798 |  | -2.25 |
| A_11_P0000025411 |  | 2.14 | A_11_P000006289 |  | -2.25 |
| A_11_P156488 | mitochondrial ribosomal protein L35 | 2.14 | A_11_P0000013896 |  | -2.25 |
| A_11_P155093 |  | 2.14 | A_11_P0000014598 |  | -2.25 |
| A_11_P0000024598 | ATPase, Ca++ transporting, type 2C, member 2 | 2.14 | A_11_P195198 | phosphatidylinositol binding clathrin assembly protein | -2.25 |
| A_11_P0000022514 | guanine monphosphate synthase | 2.13 | A_11_P115626 | septin 6 | -2.25 |
| A_11_P0000038519 |  | 2.13 | A_11_P199688 | WD and tetratricopeptide repeats 1 | -2.25 |
| A_11_P147533 | fatty acid binding protein 5 (psoriasis-associated) | 2.13 | A_11_P0000018791 |  | -2.25 |
| A_11_P154853 | low affinity immunoglobulin gamma Fc region receptor III-like | 2.13 | A_11_P0000017654 |  | -2.25 |
| A_11_P165563 |  | 2.13 | A_11_P0000024248 | jumonji domain containing 1C | -2.25 |
| A_11_P147018 | peptidylprolyl isomerase (cyclophilin A) pseudogene 1 | 2.13 | A_11_P00000866 | La ribonucleoprotein domain family, member 1 | -2.25 |
| A_11_P082416 | transcriptional adaptor 2A | 2.13 | A_11_P0000026372 | potassium channel, subfamily K, member 5 | -2.25 |
| A_11_P102791 | ADP-ribosylation factor-like 2 binding protein | 2.13 | A_11_P056896 | activin A receptor, type I | -2.25 |
| A_11_P0000022529 | polymerase (RNA) III (DNA directed) polypeptide F, 39 kDa | 2.13 | A_11_P000008057 |  | -2.25 |
| A_11_P0000034467 | insulin-like growth factor binding protein 3 | 2.13 | A_11_P187688 | spectrin, alpha, non-erythrocytic 1 | -2.24 |
| A_11_P097186 | RMI2, RecQ mediated genome instability 2, homolog (S. cerevisiae) | 2.13 | A_11_P0000023344 | retinoblastoma-like 2 (p130) | -2.24 |
| A_11_P080761 | thymidine kinase 1, soluble | 2.13 | A_11_P081346 | G patch domain containing 8 | -2.24 |
| A_11_P0000029378 | mitochondrial translational release factor 1 | 2.13 | A_11_P000002199 | runt-related transcription factor 2 | -2.24 |
| A_11_P137451 | nicotinamide phosphoribosyltransferase | 2.13 | A_11_P000009902 |  | -2.24 |
| A_11_P0000020350 |  | 2.13 | A_11_P0000035152 |  | -2.24 |
| A_11_P136741 |  | 2.13 | A_11_P0000013899 | sparc/osteonectin, cwcv and kazal-like domains proteoglycan (testican) 2 | -2.24 |
| A_11_P0000015482 |  | 2.13 | A_11_P0000021019 | major facilitator superfamily domain containing 2A | -2.24 |
| A_11_P0000017303 | laminin, gamma 1 (formerly LAMB2) | 2.13 | A_11_P0000026447 | collagen, type XIX, alpha 1 | -2.24 |
| A_11_P0000021879 | cyclin-dependent kinase 20 | 2.13 | A_11_P000007811 |  | -2.24 |
| A_11_P0000026832 | family with sequence similarity 221, member A | 2.13 | A_11_P0000028209 | ezrin | -2.24 |
| A_11_P072486 | non-histone chromosomal protein HMG-17 | 2.13 | A_11_P105131 | embigin | -2.24 |
| A_11_P052496 | ribosomal protein S3A | 2.13 | A_11_P186018 |  | -2.24 |
| A_11_P115341 | G protein-coupled receptor associated sorting protein 2 | 2.13 | A_11_P063081 | fibroblast growth factor binding protein 3 | -2.24 |
| A_11_P113786 | FXYD domain containing ion transport regulator 3 | 2.13 | A_11_P0000018685 |  | -2.24 |
| A_11_P0000025902 | malonyl CoA:ACP acyltransferase (mitochondrial) | 2.13 | A_11_P182998 |  | -2.24 |
| A_11_P0000030614 | BCL2-associated athanogene 3 | 2.13 | A_11_P058851 | F-box protein 45 | -2.24 |
| A_11_P000005819 | GULP, engulfment adaptor PTB domain containing 1 | 2.13 | A_11_P0000022962 | DENN/MADD domain containing 5B | -2.24 |
| A_11_P0000021040 |  | 2.13 | A_11_P066501 | poly (ADP-ribose) polymerase family, member 11 | -2.24 |
| A_11_P0000016813 |  | 2.13 | A_11_P0000030307 | aquaporin 2 (collecting duct) | -2.24 |
| A_11_P099206 | NME/NM23 family member 7 | 2.13 | A_11_P075591 | aminolevulinate, delta-, synthase 1 | -2.24 |
| A_11_P000004561 | protein kinase D3 | 2.13 | A_11_P0000010605 |  | -2.23 |
| A_11_P061981 | NADH dehydrogenase (ubiquinone) complex I, assembly factor 1 | 2.13 | A_11_P137711 | nuclear fragile X mental retardation protein interacting protein 2 | -2.23 |
| A_11_P073133 | ets homologous factor | 2.13 | A_11_P0000016748 |  | -2.23 |
| A_11_P0000024820 | NADH dehydrogenase (ubiquinone) 1 beta subcomplex, 10, 22kDa | 2.13 | A_11_P141918 |  | -2.23 |
| A_11_P149568 |  | 2.13 | A_11_P119486 |  | -2.23 |
| A_11_P0000023541 | ribosomal protein L4 | 2.13 | A_11_P0000021014 |  | -2.23 |
| A_11_P110355 | translocase of inner mitochondrial membrane 9 homolog (yeast) | 2.13 | A_11_P125996 | sterile alpha motif domain containing 11 | -2.23 |
| A_11_P000005675 |  | 2.13 | A_11_P0000041798 |  | -2.23 |
| A_11_P166953 |  | 2.13 | A_11_P0000010022 |  | -2.23 |
| A_11_P165388 |  | 2.13 | A_11_P177243 |  | -2.23 |
| A_11_P116601 | t-complex 11, testis-specific-like 2 | 2.13 | A_11_P176438 |  | -2.23 |
| A_11_P0000024606 |  | 2.12 | A_11_P189963 |  | -2.23 |
| A_11_P169053 | protein phosphatase 2, regulatory subunit B'', gamma | 2.12 | A_11_P0000017978 |  | -2.23 |
| A_11_P0000015491 |  | 2.12 | A_11_P176443 |  | -2.23 |
| A_11_P0000015607 | calcium/calmodulin-dependent protein kinase II alpha | 2.12 | A_11_P0000023366 | lymphocyte-specific protein tyrosine kinase | -2.23 |
| A_11_P0000039052 |  | 2.12 | A_11_P172833 |  | -2.23 |
| A_11_P0000041179 | high mobility group box 3 | 2.12 | A_11_P155718 |  | -2.23 |
| A_11_P163188 | carboxypeptidase E | 2.12 | A_11_P133471 |  | -2.23 |
| A_11_P0000022195 | peter pan homolog (Drosophila) | 2.12 | A_11_P0000010878 |  | -2.23 |
| A_11_P163153 | integrin, beta 5 | 2.12 | A_11_P0000030446 |  | -2.23 |
| A_11_P0000038795 | tropomyosin 1 (alpha) | 2.12 | A_11_P0000027343 | additional sex combs like 2 (Drosophila) | -2.23 |
| A_11_P094551 | aldehyde dehydrogenase 7 family, member A1 | 2.12 | A_11_P151898 | SET domain containing 2 | -2.23 |
| A_11_P195190 |  | 2.12 | A_11_P153053 |  | -2.23 |
| A_11_P0000012717 |  | 2.12 | A_11_P062426 | myosin IE | -2.23 |
| A_11_P0000014924 |  | 2.12 | A_11_P0000032738 | UDP-N-acetylglucosamine/UDP-glucose/GDP-mannose transporter-like | -2.23 |
| A_11_P000001643 | mutS homolog 2, colon cancer, nonpolyposis type 1 (E. coli) | 2.12 | A_11_P0000024445 | dynein, cytoplasmic 2, heavy chain 1 | -2.23 |
| A_11_P157563 | arginase 1 | 2.12 | A_11_P183463 | IQ motif containing GTPase activating protein 1 | -2.23 |
| A_11_P0000023613 | scavenger receptor class B, member 2 | 2.12 | A_11_P216738 | kinesin family member 21A | -2.23 |
| A_11_P0000023976 | eukaryotic translation elongation factor 1 beta 2 | 2.12 | A_11_P0000026319 |  | -2.23 |
| A_11_P0000029947 | delta/notch-like EGF repeat containing | 2.12 | A_11_P0000019709 | desmoglein 3 | -2.23 |
| A_11_P0000024738 | Tu translation elongation factor, mitochondrial | 2.12 | A_11_P190568 | TEA domain family member 4 | -2.23 |
| A_11_P150663 |  | 2.12 | A_11_P0000020435 | spectrin, beta, non-erythrocytic 1 | -2.23 |
| A_11_P0000025204 |  | 2.12 | A_11_P215153 | mitochondrial fission factor | -2.23 |
| A_11_P0000019174 |  | 2.12 | A_11_P0000031685 | cytidine monophosphate-N-acetylneuraminic acid hydroxylase-like | -2.23 |
| A_11_P0000024098 | isocitrate dehydrogenase 2 (NADP+), mitochondrial | 2.12 | A_11_P098221 |  | -2.23 |
| A_11_P079271 |  | 2.12 | A_11_P0000015160 |  | -2.23 |
| A_11_P101926 | GATA binding protein 3 | 2.12 | A_11_P0000010212 |  | -2.23 |
| A_11_P0000035935 |  | 2.12 | A_11_P0000026573 | tribbles homolog 1 (Drosophila) | -2.23 |
| A_11_P082896 | LSM5 homolog, U6 small nuclear RNA associated (S. cerevisiae) | 2.12 | A_11_P132986 | interleukin 7 | -2.22 |
| A_11_P077396 | WD repeat domain 18 | 2.12 | A_11_P072301 | mitogen-activated protein kinase kinase kinase 2 | -2.22 |
| A_11_P0000021345 | steroid-5-alpha-reductase, alpha polypeptide 2 (3-oxo-5 alpha-steroid delta 4-dehydrogenase alpha 2) | 2.12 | A_11_P053761 | junction plakoglobin | -2.22 |
| A_11_P207168 |  | 2.12 | A_11_P213373 |  | -2.22 |
| A_11_P107391 | mago-nashi homolog, proliferation-associated (Drosophila) | 2.12 | A_11_P0000016517 |  | -2.22 |
| A_11_P089781 | chromosome 10 open reading frame, human C2orf40 | 2.12 | A_11_P214008 |  | -2.22 |
| A_11_P156503 |  | 2.12 | A_11_P104236 |  | -2.22 |
| A_11_P0000034185 | phosphohistidine phosphatase 1 | 2.12 | A_11_P105846 | ubiquitin specific peptidase 2 | -2.22 |
| A_11_P141120 |  | 2.11 | A_11_P0000012332 |  | -2.22 |
| A_11_P000004295 |  | 2.11 | A_11_P0000019798 | progesterone receptor | -2.22 |
| A_11_P156028 | ADP-ribosylation-like factor 6 interacting protein 4 | 2.11 | A_11_P202983 |  | -2.22 |
| A_11_P081426 |  | 2.11 | A_11_P00000532 | cortactin | -2.22 |
| A_11_P118211 | transmembrane protein 43 | 2.11 | A_11_P0000040640 | pre-B-cell leukemia homeobox interacting protein 1 | -2.22 |
| A_11_P0000021343 | WD repeat domain 43 | 2.11 | A_11_P000008592 |  | -2.22 |
| A_11_P075301 |  | 2.11 | A_11_P114081 | oral-facial-digital syndrome 1 | -2.22 |
| A_11_P087466 | CD8b molecule | 2.11 | A_11_P112481 | sialic acid binding Ig-like lectin 10 | -2.22 |
| A_11_P057296 | ubiquitin-conjugating enzyme E2E 3 | 2.11 | A_11_P0000014581 |  | -2.22 |
| A_11_P102226 | protocadherin beta 15 | 2.11 | A_11_P124421 | ring finger protein 128, E3 ubiquitin protein ligase | -2.22 |
| A_11_P0000031850 | abl-interactor 2 | 2.11 | A_11_P201858 |  | -2.22 |
| A_11_P117191 | ribosomal protein S3 | 2.11 | A_11_P0000030600 | family with sequence similarity 160, member B1 | -2.22 |
| A_11_P0000020079 | complement component 5a receptor 1 | 2.11 | A_11_P00000249 |  | -2.22 |
| A_11_P0000021606 | mitochondrial ribosomal protein L11 | 2.11 | A_11_P138851 |  | -2.22 |
| A_11_P065801 | NEL-like 2 (chicken) | 2.11 | A_11_P175758 | jumonji, AT rich interactive domain 2 | -2.22 |
| A_11_P172763 |  | 2.11 | A_11_P0000037414 |  | -2.22 |
| A_11_P0000038981 | platelet derived growth factor D | 2.11 | A_11_P160068 |  | -2.22 |
| A_11_P109241 |  | 2.11 | A_11_P195148 |  | -2.22 |
| A_11_P000006356 | Obg-like ATPase 1 | 2.11 | A_11_P059126 | heparanase | -2.22 |
| A_11_P090496 | small nuclear ribonucleoprotein polypeptide G | 2.11 | A_11_P0000012641 |  | -2.22 |
| A_11_P124636 |  | 2.11 | A_11_P0000017540 |  | -2.22 |
| A_11_P165633 | thimet oligopeptidase 1 | 2.11 | A_11_P100936 | signal-regulatory protein beta-1 isoform 3-like | -2.22 |
| A_11_P157868 |  | 2.11 | A_11_P000004465 |  | -2.22 |
| A_11_P161493 |  | 2.11 | A_11_P0000010138 |  | -2.22 |
| A_11_P0000041996 | caseinolytic mitochondrial matrix peptidase proteolytic subunit | 2.11 | A_11_P000006861 |  | -2.22 |
| A_11_P0000032899 |  | 2.11 | A_11_P00000598 | ring finger protein 31 | -2.22 |
| A_11_P0000017758 | small glutamine-rich tetratricopeptide repeat (TPR)-containing, beta | 2.11 | A_11_P199979 |  | -2.22 |
| A_11_P0000024758 | crystallin, mu | 2.11 | A_11_P169058 |  | -2.22 |
| A_11_P115051 |  | 2.11 | A_11_P000007996 |  | -2.22 |
| A_11_P0000027556 | Yae1 domain containing 1 | 2.11 | A_11_P0000013635 |  | -2.21 |
| A_11_P109971 | non-SMC condensin I complex, subunit G | 2.11 | A_11_P0000032887 | PH domain and leucine rich repeat protein phosphatase 2 | -2.21 |
| A_11_P089301 | solute carrier family 25 (mitochondrial carrier; peroxisomal membrane protein, 34kDa), member 17 | 2.11 | A_11_P169763 | malic enzyme 2, NAD(+)-dependent, mitochondrial | -2.21 |
| A_11_P0000017231 | GDNF family receptor alpha 2 | 2.11 | A_11_P0000023263 | solute carrier family 23 (nucleobase transporters), member 1 | -2.21 |
| A_11_P0000020419 | phosphatidylinositol glycan anchor biosynthesis, class F | 2.11 | A_11_P133316 |  | -2.21 |
| A_11_P155013 |  | 2.11 | A_11_P188283 |  | -2.21 |
| A_11_P0000019434 |  | 2.11 | A_11_P168603 |  | -2.21 |
| A_11_P0000031330 |  | 2.11 | A_11_P117441 | related RAS viral (r-ras) oncogene homolog 2 | -2.21 |
| A_11_P0000019393 |  | 2.11 | A_11_P0000011727 |  | -2.21 |
| A_11_P188983 | tropomyosin 2 (beta) | 2.11 | A_11_P149428 | atlastin GTPase 2 | -2.21 |
| A_11_P0000016767 | transmembrane protein 206 | 2.11 | A_11_P0000022580 |  | -2.21 |
| A_11_P0000029320 | TEA domain family member 1 (SV40 transcriptional enhancer factor) | 2.11 | A_11_P196978 |  | -2.21 |
| A_11_P065166 | serpin peptidase inhibitor, clade I (neuroserpin), member 1 | 2.11 | A_11_P065911 | DENN/MADD domain containing 5B | -2.21 |
| A_11_P139896 |  | 2.11 | A_11_P0000032781 | pleckstrin homology domain containing, family N member 1 | -2.21 |
| A_11_P0000038585 |  | 2.10 | A_11_P0000012462 |  | -2.21 |
| A_11_P124426 | SEH1-like (S. cerevisiae) | 2.10 | A_11_P0000017068 | runt-related transcription factor 2 | -2.21 |
| A_11_P182038 | ribosomal protein S3 | 2.10 | A_11_P0000041451 |  | -2.21 |
| A_11_P162393 | NIN1/RPN12 binding protein 1 homolog (S. cerevisiae) | 2.10 | A_11_P0000024709 | ring finger protein 216 | -2.21 |
| A_11_P222323 | SWI/SNF related, matrix associated, actin dependent regulator of chromatin, subfamily d, member 3 | 2.10 | A_11_P146963 |  | -2.21 |
| A_11_P061411 | carbonyl reductase 3 | 2.10 | A_11_P127751 | family with sequence similarity 190, member B | -2.21 |
| A_11_P153898 | heat shock 60kDa protein 1 (chaperonin) | 2.10 | A_11_P0000031539 |  | -2.21 |
| A_11_P0000028160 | v-myb avian myeloblastosis viral oncogene homolog | 2.10 | A_11_P0000031906 | BTG family, member 2 | -2.21 |
| A_11_P000004094 |  | 2.10 | A_11_P0000023525 | RAR-related orphan receptor A | -2.21 |
| A_11_P176903 |  | 2.10 | A_11_P0000029744 | solute carrier family 13 (sodium-dependent dicarboxylate transporter), member 3 | -2.21 |
| A_11_P0000029966 | ADP-ribosylation factor-like 4C | 2.10 | A_11_P0000010783 |  | -2.21 |
| A_11_P205078 | G protein pathway suppressor 1 | 2.10 | A_11_P100913 | signal-regulatory protein beta-1 isoform 3-like | -2.21 |
| A_11_P000008299 |  | 2.10 | A_11_P0000010407 |  | -2.21 |
| A_11_P076616 | SWIM-type zinc finger 7 associated protein 1 | 2.10 | A_11_P0000031475 | G protein-coupled receptor 156 | -2.21 |
| A_11_P0000019001 |  | 2.10 | A_11_P0000032871 |  | -2.21 |
| A_11_P0000039023 |  | 2.10 | A_11_P218513 | flavin containing monooxygenase 5 | -2.21 |
| A_11_P0000017802 |  | 2.10 | A_11_P080891 | RecQ protein-like 5 | -2.21 |
| A_11_P0000039506 |  | 2.10 | A_11_P0000032411 | microfibrillar-associated protein 3 | -2.21 |
| A_11_P199108 | tumor protein D52-like 2 | 2.10 | A_11_P187283 | MHC class I DLA-12 | -2.21 |
| A_11_P054391 |  | 2.10 | A_11_P053291 | X-linked inhibitor of apoptosis | -2.21 |
| A_11_P163263 | phosphatidylglycerophosphate synthase 1 | 2.10 | A_11_P187278 |  | -2.21 |
| A_11_P091636 | primase, DNA, polypeptide 2 (58kDa) | 2.10 | A_11_P124166 |  | -2.21 |
| A_11_P0000033953 | N-acetylglucosaminidase, alpha | 2.10 | A_11_P070231 | chloride channel, voltage-sensitive 3 | -2.20 |
| A_11_P0000033710 | abhydrolase domain containing 12B | 2.10 | A_11_P076111 | solute carrier family 25, member 42 | -2.20 |
| A_11_P0000018389 | achaete-scute complex homolog 2 (Drosophila) | 2.10 | A_11_P066941 |  | -2.20 |
| A_11_P000006266 |  | 2.10 | A_11_P072446 |  | -2.20 |
| A_11_P0000017928 | ST6 (alpha-N-acetyl-neuraminyl-2,3-beta-galactosyl-1,3)-N-acetylgalactosaminide alpha-2,6-sialyltransferase 6 | 2.10 | A_11_P199428 |  | -2.20 |
| A_11_P119331 |  | 2.10 | A_11_P063651 | acyl-CoA synthetase long-chain family member 5 | -2.20 |
| A_11_P219943 | DEAD (Asp-Glu-Ala-Asp) box helicase 1 | 2.10 | A_11_P000008126 |  | -2.20 |
| A_11_P0000025544 | heat shock 70kDa protein 5 (glucose-regulated protein, 78kDa) | 2.10 | A_11_P000009817 |  | -2.20 |
| A_11_P118661 |  | 2.10 | A_11_P0000035341 |  | -2.20 |
| A_11_P218888 | anaphase promoting complex subunit 10 | 2.10 | A_11_P0000015172 |  | -2.20 |
| A_11_P0000017214 | mitochondrial ribosome recycling factor | 2.10 | A_11_P0000030027 | zinc finger protein 664 | -2.20 |
| A_11_P0000020164 | endothelin receptor type B | 2.10 | A_11_P0000010120 |  | -2.20 |
| A_11_P0000039181 |  | 2.10 | A_11_P169383 |  | -2.20 |
| A_11_P073741 | proteasome (prosome, macropain) 26S subunit, ATPase, 3 | 2.10 | A_11_P0000031137 | SECIS binding protein 2-like | -2.20 |
| A_11_P0000023256 | pitrilysin metallopeptidase 1 | 2.10 | A_11_P0000028425 |  | -2.20 |
| A_11_P076961 | caseinolytic mitochondrial matrix peptidase proteolytic subunit | 2.10 | A_11_P0000034132 | slingshot protein phosphatase 2 | -2.20 |
| A_11_P122307 |  | 2.10 | A_11_P208289 | phosphorylase, glycogen, muscle | -2.20 |
| A_11_P085491 | NOP2/Sun domain family, member 4 | 2.10 | A_11_P0000016159 |  | -2.20 |
| A_11_P0000024315 | pituitary tumor-transforming 1 | 2.10 | A_11_P0000041499 |  | -2.20 |
| A_11_P073146 | ets homologous factor | 2.10 | A_11_P200028 |  | -2.20 |
| A_11_P0000022729 | elongator acetyltransferase complex subunit 3 | 2.10 | A_11_P0000012758 |  | -2.20 |
| A_11_P00000348 | alkB, alkylation repair homolog 3 (E. coli) | 2.10 | A_11_P0000020709 |  | -2.20 |
| A_11_P0000013826 | non-metastatic cells 2, protein (NM23B) expressed in | 2.09 | A_11_P0000030849 | mesoderm induction early response 1, family member 3 | -2.20 |
| A_11_P0000040448 | BCL2/adenovirus E1B 19 kDa protein-interacting protein 3-like | 2.09 | A_11_P0000026080 | fem-1 homolog c (C. elegans) | -2.20 |
| A_11_P051496 | ribosomal protein, large, P0 | 2.09 | A_11_P0000027978 | autophagy related 2A | -2.20 |
| A_11_P0000029642 | inosine triphosphatase (nucleoside triphosphate pyrophosphatase) | 2.09 | A_11_P0000037734 |  | -2.20 |
| A_11_P0000031747 |  | 2.09 | A_11_P000008848 |  | -2.20 |
| A_11_P072431 | LY6/PLAUR domain containing 1 | 2.09 | A_11_P074051 | protein phosphatase 6, regulatory subunit 3 | -2.19 |
| A_11_P053666 | phosphatidylinositol glycan anchor biosynthesis, class N | 2.09 | A_11_P139686 |  | -2.19 |
| A_11_P000009970 |  | 2.09 | A_11_P188153 |  | -2.19 |
| A_11_P0000041413 |  | 2.09 | A_11_P120136 | mesoderm induction early response 1, family member 3 | -2.19 |
| A_11_P0000019836 |  | 2.09 | A_11_P000003860 |  | -2.19 |
| A_11_P0000028188 | nucleoporin 43kDa | 2.09 | A_11_P0000013769 | interleukin 6 signal transducer (gp130, oncostatin M receptor) | -2.19 |
| A_11_P205818 |  | 2.09 | A_11_P057786 | Kruppel-like factor 7 (ubiquitous) | -2.19 |
| A_11_P0000025282 | tryptophanyl-tRNA synthetase | 2.09 | A_11_P071376 | glycerol-3-phosphate dehydrogenase 1-like | -2.19 |
| A_11_P168358 | UDP-GlcNAc:betaGal beta-1,3-N-acetylglucosaminyltransferase 1 | 2.09 | A_11_P0000035228 |  | -2.19 |
| A_11_P0000032947 | general transcription factor II-I repeat domain-containing protein 2-like | 2.09 | A_11_P197977 |  | -2.19 |
| A_11_P121586 | hydroxysteroid dehydrogenase like 2 | 2.09 | A_11_P145818 | family with sequence similarity 20, member B | -2.19 |
| A_11_P083931 | ribosomal protein L7 | 2.09 | A_11_P182028 | kinesin heavy chain member 2A | -2.19 |
| A_11_P066136 | matrix Gla protein | 2.09 | A_11_P0000013982 |  | -2.19 |
| A_11_P117021 |  | 2.09 | A_11_P0000030979 | progestin and adipoQ receptor family member VII | -2.19 |
| A_11_P111881 | ribosomal protein S5 | 2.09 | A_11_P0000024881 |  | -2.19 |
| A_11_P0000030020 | glycosyltransferase 1 domain containing 1 | 2.09 | A_11_P000006084 |  | -2.19 |
| A_11_P0000027439 | methylenetetrahydrofolate dehydrogenase (NADP+ dependent) 2, methenyltetrahydrofolate cyclohydrolase | 2.09 | A_11_P068301 | family with sequence similarity 76, member B | -2.19 |
| A_11_P0000010722 |  | 2.09 | A_11_P0000023104 | acyl-CoA synthetase long-chain family member 5 | -2.19 |
| A_11_P0000014222 |  | 2.09 | A_11_P079636 | engulfment and cell motility 1 | -2.19 |
| A_11_P079471 | heterogeneous nuclear ribonucleoprotein A2/B1 | 2.09 | A_11_P125286 |  | -2.19 |
| A_11_P000004787 |  | 2.09 | A_11_P094311 | mitogen-activated protein kinase 9 | -2.19 |
| A_11_P193468 |  | 2.09 | A_11_P098831 | syntaxin 6 | -2.19 |
| A_11_P139516 | T-cell immunoglobulin and mucin domain containing 4 | 2.09 | A_11_P0000040286 |  | -2.19 |
| A_11_P071121 | cartilage associated protein | 2.09 | A_11_P188238 | integrator complex subunit 9 | -2.19 |
| A_11_P0000024939 |  | 2.09 | A_11_P000004118 |  | -2.19 |
| A_11_P0000034150 | KIAA0664 ortholog | 2.09 | A_11_P0000014367 |  | -2.19 |
| A_11_P0000024320 | clathrin interactor 1 | 2.09 | A_11_P0000027329 | mesogenin 1 | -2.19 |
| A_11_P0000028058 |  | 2.09 | A_11_P208398 | potassium voltage-gated channel, KQT-like subfamily, member 5 | -2.19 |
| A_11_P083376 | exosome component 2 | 2.09 | A_11_P060201 |  | -2.19 |
| A_11_P0000029980 | NADH dehydrogenase [ubiquinone] 1 alpha subcomplex subunit 10, mitochondrial-like | 2.09 | A_11_P0000016677 |  | -2.19 |
| A_11_P0000017533 |  | 2.09 | A_11_P166408 |  | -2.19 |
| A_11_P153813 | exophilin 5 | 2.09 | A_11_P065331 | uncharacterized LOC607625 | -2.18 |
| A_11_P0000040604 | DNA (cytosine-5-)-methyltransferase 1 | 2.09 | A_11_P200538 | epithelial membrane protein 1 | -2.18 |
| A_11_P077976 |  | 2.09 | A_11_P0000036230 |  | -2.18 |
| A_11_P083481 |  | 2.09 | A_11_P0000024866 | dihydropyrimidine dehydrogenase | -2.18 |
| A_11_P051526 |  | 2.08 | A_11_P0000038057 |  | -2.18 |
| A_11_P0000019452 |  | 2.08 | A_11_P130866 |  | -2.18 |
| A_11_P137961 | 3'-phosphoadenosine 5'-phosphosulfate synthase 1 | 2.08 | A_11_P0000024745 | dynactin 5 (p25) | -2.18 |
| A_11_P081876 | mitochondrial ribosomal protein L45 | 2.08 | A_11_P062651 |  | -2.18 |
| A_11_P0000041145 |  | 2.08 | A_11_P0000029674 | chromosome 24 open reading frame, human C20orf112 | -2.18 |
| A_11_P0000029448 | growth arrest-specific 6 | 2.08 | A_11_P0000030168 | ureidopropionase, beta | -2.18 |
| A_11_P109926 | peroxisome proliferator-activated receptor gamma, coactivator 1 alpha | 2.08 | A_11_P0000033662 |  | -2.18 |
| A_11_P157838 | sulfite oxidase | 2.08 | A_11_P159003 |  | -2.18 |
| A_11_P0000032701 | elaC ribonuclease Z 2 | 2.08 | A_11_P172338 | DEAD (Asp-Glu-Ala-Asp) box helicase 3, X-linked | -2.18 |
| A_11_P0000024575 | solute carrier family 25 (pyrimidine nucleotide carrier), member 33 | 2.08 | A_11_P104231 | SAR1 homolog A (S. cerevisiae) | -2.18 |
| A_11_P0000020675 | bystin-like | 2.08 | A_11_P000002349 |  | -2.18 |
| A_11_P0000029384 | protocadherin 17 | 2.08 | A_11_P112396 |  | -2.18 |
| A_11_P215748 | ribosome production factor 1 homolog (S. cerevisiae) | 2.08 | A_11_P194443 | protein-L-isoaspartate (D-aspartate) O-methyltransferase domain containing 2 | -2.18 |
| A_11_P0000020249 | non-metastatic cells 2, protein (NM23B) expressed in | 2.08 | A_11_P150118 | MHC class I DLA-64 | -2.18 |
| A_11_P0000031898 | monoacylglycerol O-acyltransferase 1 | 2.08 | A_11_P0000041947 |  | -2.18 |
| A_11_P189473 | adenosylmethionine decarboxylase 1 | 2.08 | A_11_P110636 |  | -2.18 |
| A_11_P187553 | potassium channel tetramerization domain containing 3 | 2.08 | A_11_P202993 |  | -2.18 |
| A_11_P0000040745 |  | 2.08 | A_11_P000003636 |  | -2.18 |
| A_11_P0000041250 |  | 2.08 | A_11_P0000030144 | eukaryotic translation initiation factor 4E nuclear import factor 1 | -2.18 |
| A_11_P096101 | processing of precursor 7, ribonuclease P/MRP subunit (S. cerevisiae) | 2.08 | A_11_P0000034670 | protein phosphatase 1, regulatory subunit 3F | -2.18 |
| A_11_P085326 | zinc finger, FYVE domain containing 9 | 2.08 | A_11_P210048 | talin 1 | -2.18 |
| A_11_P108231 | dihydroorotate dehydrogenase (quinone) | 2.08 | A_11_P0000014397 |  | -2.18 |
| A_11_P114161 | sex comb on midleg-like 2 (Drosophila) | 2.08 | A_11_P0000038229 |  | -2.18 |
| A_11_P0000021627 | protein phosphatase 1, regulatory (inhibitor) subunit 14B | 2.08 | A_11_P149453 |  | -2.18 |
| A_11_P063446 | transmembrane protein 180 | 2.08 | A_11_P000005446 |  | -2.18 |
| A_11_P00000467 | runt-related transcription factor 1; translocated to, 1 (cyclin D-related) | 2.08 | A_11_P186848 |  | -2.18 |
| A_11_P191743 | COP9 signalosome subunit 3 | 2.08 | A_11_P0000032290 | perforin 1 (pore forming protein) | -2.18 |
| A_11_P155888 | Obg-like ATPase 1 | 2.08 | A_11_P187178 |  | -2.18 |
| A_11_P155908 |  | 2.08 | A_11_P0000027863 | diacylglycerol kinase, zeta | -2.18 |
| A_11_P0000022841 | replication factor C (activator 1) 5, 36.5kDa | 2.08 | A_11_P0000016251 |  | -2.18 |
| A_11_P0000016451 | torsin family 3, member A | 2.08 | A_11_P156528 | membrane-associated ring finger (C3HC4) 7, E3 ubiquitin protein ligase | -2.18 |
| A_11_P140166 |  | 2.08 | A_11_P117646 |  | -2.17 |
| A_11_P102516 | leucine rich repeat containing 70 | 2.08 | A_11_P074356 | solute carrier family 22 member 20-like | -2.17 |
| A_11_P0000019529 |  | 2.08 | A_11_P0000024032 | complement factor H-like | -2.17 |
| A_11_P147093 |  | 2.08 | A_11_P0000023627 | lin-54 homolog (C. elegans) | -2.17 |
| A_11_P172828 |  | 2.08 | A_11_P119976 | polymerase (RNA) II (DNA directed) polypeptide A, 220kDa | -2.17 |
| A_11_P0000022505 | eukaryotic translation initiation factor 2A, 65kDa | 2.08 | A_11_P000008919 |  | -2.17 |
| A_11_P163438 | RNA methyltransferase like 1 | 2.08 | A_11_P179093 |  | -2.17 |
| A_11_P072136 | polo-like kinase 4 | 2.08 | A_11_P0000020440 |  | -2.17 |
| A_11_P166853 | tachykinin receptor 1 | 2.08 | A_11_P0000027190 |  | -2.17 |
| A_11_P096921 | polymerase (RNA) III (DNA directed) polypeptide E (80kD) | 2.08 | A_11_P205493 | vacuolar protein sorting 13 homolog D (S. cerevisiae) | -2.17 |
| A_11_P190843 | myozenin 1 | 2.08 | A_11_P172048 |  | -2.17 |
| A_11_P0000030008 | peroxisomal membrane protein 2, 22kDa | 2.08 | A_11_P125886 | calcium activated nucleotidase 1 | -2.17 |
| A_11_P063376 | mitochondrial ribosomal protein L43 | 2.08 | A_11_P177328 |  | -2.17 |
| A_11_P0000022464 | biotinidase | 2.08 | A_11_P055421 | retinoblastoma binding protein 5 | -2.17 |
| A_11_P0000034191 | DNL-type zinc finger | 2.08 | A_11_P160253 |  | -2.17 |
| A_11_P0000030198 | zinc finger protein 74 | 2.07 | A_11_P066496 | poly (ADP-ribose) polymerase family, member 11 | -2.17 |
| A_11_P0000021214 | non-SMC condensin II complex, subunit G2 | 2.07 | A_11_P0000028069 | myosin VIIB | -2.17 |
| A_11_P172718 |  | 2.07 | A_11_P0000018712 |  | -2.17 |
| A_11_P138681 |  | 2.07 | A_11_P098456 | protein phosphatase 1, regulatory subunit 12B | -2.17 |
| A_11_P0000027927 | adrenergic, beta, receptor kinase 1 | 2.07 | A_11_P0000015808 | karyopherin alpha 4 (importin alpha 3) | -2.17 |
| A_11_P097376 | DnaJ (Hsp40) homolog, subfamily A, member 3 | 2.07 | A_11_P0000026138 | SEC24 family, member A (S. cerevisiae) | -2.17 |
| A_11_P0000021643 | heterogeneous nuclear ribonucleoprotein U-like 2 | 2.07 | A_11_P079911 | leucine-rich repeats and calponin homology (CH) domain containing 1 | -2.17 |
| A_11_P076651 | SPC24, NDC80 kinetochore complex component | 2.07 | A_11_P150748 |  | -2.17 |
| A_11_P110236 | collagen and calcium binding EGF domains 1 | 2.07 | A_11_P0000039287 | myosin light chain kinase 3 | -2.17 |
| A_11_P0000031617 | solute carrier family 7, member 14 | 2.07 | A_11_P102126 |  | -2.17 |
| A_11_P0000027890 | mitochondrial ribosomal protein L23 | 2.07 | A_11_P0000019853 | relaxin 1 | -2.17 |
| A_11_P0000020354 | ribosomal protein L3 | 2.07 | A_11_P178303 | chymotrypsin-like elastase family, member 3B | -2.17 |
| A_11_P0000023242 | selenophosphate synthetase 1 | 2.07 | A_11_P0000031371 | GPRIN family member 3 | -2.17 |
| A_11_P0000023363 | tyrosyl-tRNA synthetase | 2.07 | A_11_P0000029864 | gap junction protein, beta 2, 26kDa | -2.17 |
| A_11_P098286 | nexilin (F actin binding protein) | 2.07 | A_11_P000006360 |  | -2.17 |
| A_11_P088631 | XRCC6 binding protein 1 | 2.07 | A_11_P0000031094 | ras homolog family member V | -2.17 |
| A_11_P128281 |  | 2.07 | A_11_P106971 |  | -2.17 |
| A_11_P191978 | required for meiotic nuclear division 5 homolog B (S. cerevisiae) | 2.07 | A_11_P0000039692 |  | -2.17 |
| A_11_P190983 | Ras-related GTP binding D | 2.07 | A_11_P0000014828 |  | -2.17 |
| A_11_P0000040129 | chromobox homolog 1 | 2.07 | A_11_P0000029099 | ADAMTS-like 5 | -2.17 |
| A_11_P00000471 | RNA polymerase II associated protein 3 | 2.07 | A_11_P198373 | protein phosphatase 1, catalytic subunit, gamma isozyme | -2.17 |
| A_11_P168058 | ribosomal protein, large, P0 | 2.07 | A_11_P0000029347 | membrane-spanning 4-domains, subfamily A, member 1 | -2.16 |
| A_11_P000002569 |  | 2.07 | A_11_P0000031087 | bromo adjacent homology domain containing 1 | -2.16 |
| A_11_P0000031020 | Rho guanine nucleotide exchange factor (GEF) 10-like | 2.07 | A_11_P214673 |  | -2.16 |
| A_11_P138003 | non-histone chromosomal protein HMG-17 | 2.06 | A_11_P000004258 |  | -2.16 |
| A_11_P0000021767 |  | 2.06 | A_11_P127711 |  | -2.16 |
| A_11_P155058 | deoxycytidylate deaminase-like | 2.06 | A_11_P206908 |  | -2.16 |
| A_11_P0000024257 | DEAD (Asp-Glu-Ala-Asp) box helicase 21 | 2.06 | A_11_P211348 | plexin B1 | -2.16 |
| A_11_P184453 |  | 2.06 | A_11_P000008270 |  | -2.16 |
| A_11_P138666 |  | 2.06 | A_11_P0000018023 |  | -2.16 |
| A_11_P0000038803 |  | 2.06 | A_11_P064286 | nuclear receptor coactivator 2 | -2.16 |
| A_11_P155533 | deleted in liver cancer 1 | 2.06 | A_11_P0000015798 | family with sequence similarity 189, member A2 | -2.16 |
| A_11_P077481 |  | 2.06 | A_11_P000007153 |  | -2.16 |
| A_11_P080331 | propionyl CoA carboxylase, alpha polypeptide | 2.06 | A_11_P0000018073 |  | -2.16 |
| A_11_P0000040812 | BMX non-receptor tyrosine kinase | 2.06 | A_11_P000007113 |  | -2.16 |
| A_11_P0000024498 |  | 2.06 | A_11_P119671 | mitogen-activated protein kinase 1 interacting protein 1-like | -2.16 |
| A_11_P0000022991 | DEAD (Asp-Glu-Ala-Asp) box polypeptide 47 | 2.06 | A_11_P0000037140 | cytoplasmic polyadenylation element binding protein 4 | -2.16 |
| A_11_P0000023062 | retinol binding protein 4, plasma | 2.06 | A_11_P0000010833 |  | -2.16 |
| A_11_P0000033527 | S100 calcium binding protein A16 | 2.06 | A_11_P189233 |  | -2.16 |
| A_11_P094786 | transforming growth factor, beta-induced, 68kDa | 2.06 | A_11_P0000016947 | C2CD2-like | -2.16 |
| A_11_P138086 |  | 2.06 | A_11_P0000013969 |  | -2.16 |
| A_11_P098161 | 15 kDa selenoprotein | 2.06 | A_11_P169808 |  | -2.16 |
| A_11_P188398 | ribosomal protein S7 | 2.06 | A_11_P101741 | Rho GTPase activating protein 12 | -2.16 |
| A_11_P099386 | saccharopine dehydrogenase (putative) | 2.06 | A_11_P0000018451 | heparin-binding EGF-like growth factor | -2.16 |
| A_11_P0000020829 | TBC1 domain family, member 31 | 2.06 | A_11_P088791 |  | -2.16 |
| A_11_P169233 |  | 2.06 | A_11_P0000018730 |  | -2.16 |
| A_11_P0000012525 |  | 2.06 | A_11_P000003799 |  | -2.16 |
| A_11_P0000028960 | ECSIT signalling integrator | 2.06 | A_11_P057581 | spermatogenesis associated, serine-rich 2-like | -2.16 |
| A_11_P0000033682 | KIAA0391 ortholog | 2.06 | A_11_P204343 |  | -2.16 |
| A_11_P085596 | eukaryotic translation initiation factor 2B, subunit 3 gamma, 58kDa | 2.06 | A_11_P0000011859 |  | -2.16 |
| A_11_P0000017498 |  | 2.06 | A_11_P0000040859 |  | -2.16 |
| A_11_P113851 | centrosomal protein 89kDa | 2.06 | A_11_P0000041157 |  | -2.16 |
| A_11_P00000656 | one cut domain family member 3-like | 2.06 | A_11_P000008060 |  | -2.16 |
| A_11_P0000019749 | prostaglandin-endoperoxide synthase 1 (prostaglandin G/H synthase and cyclooxygenase) | 2.06 | A_11_P0000023959 | spermatogenesis associated, serine-rich 2-like | -2.16 |
| A_11_P0000023531 | peptidylprolyl isomerase B (cyclophilin B) | 2.06 | A_11_P181623 | programmed cell death 6 interacting protein | -2.16 |
| A_11_P000005869 |  | 2.05 | A_11_P199503 | MHC class I DLA-12 | -2.16 |
| A_11_P0000026777 | GTP-binding protein 10 (putative) | 2.05 | A_11_P0000026771 | ATP-binding cassette, sub-family B (MDR/TAP), member 4 | -2.16 |
| A_11_P082806 | gem (nuclear organelle) associated protein 4 | 2.05 | A_11_P101726 |  | -2.15 |
| A_11_P123001 | FK506 binding protein 4, 59kDa | 2.05 | A_11_P000006000 |  | -2.15 |
| A_11_P138501 |  | 2.05 | A_11_P0000025908 | N-acetylgalactosaminidase, alpha- | -2.15 |
| A_11_P000006128 |  | 2.05 | A_11_P000004075 |  | -2.15 |
| A_11_P0000033108 | polo-like kinase 1 | 2.05 | A_11_P175353 | P450 (cytochrome) oxidoreductase | -2.15 |
| A_11_P000001741 |  | 2.05 | A_11_P087436 |  | -2.15 |
| A_11_P155028 | moesin | 2.05 | A_11_P0000032977 |  | -2.15 |
| A_11_P0000030653 | thymocyte selection-associated high mobility group box | 2.05 | A_11_P203048 | tuftelin interacting protein 11 | -2.15 |
| A_11_P000005037 | SERPINE1 mRNA binding protein 1 | 2.05 | A_11_P094831 | family with sequence similarity 13, member B | -2.15 |
| A_11_P063261 | arginine vasopressin-induced 1 | 2.05 | A_11_P0000018462 |  | -2.15 |
| A_11_P0000031293 | v-ets avian erythroblastosis virus E26 oncogene homolog | 2.05 | A_11_P0000032572 | Cbl proto-oncogene, E3 ubiquitin protein ligase | -2.15 |
| A_11_P0000018400 | splicing factor 3b, subunit 5, 10kDa | 2.05 | A_11_P106746 | hairy and enhancer of split 7 (Drosophila) | -2.15 |
| A_11_P146928 |  | 2.05 | A_11_P0000022045 | SET domain containing 5 | -2.15 |
| A_11_P0000023730 | coiled-coil domain containing 58 | 2.05 | A_11_P0000027037 | suppressor of cytokine signaling 2 | -2.15 |
| A_11_P0000032916 | glucose-fructose oxidoreductase domain containing 2 | 2.05 | A_11_P000009035 |  | -2.15 |
| A_11_P0000021454 |  | 2.05 | A_11_P104096 | nuclear receptor binding factor 2 | -2.15 |
| A_11_P064351 |  | 2.05 | A_11_P0000018386 |  | -2.15 |
| A_11_P0000030961 | mitogen-activated protein kinase kinase kinase 6 | 2.05 | A_11_P101881 | optineurin | -2.15 |
| A_11_P00000404 | storkhead box 2 | 2.05 | A_11_P0000040941 |  | -2.15 |
| A_11_P182663 | retinol binding protein 4, plasma | 2.05 | A_11_P0000023731 |  | -2.15 |
| A_11_P071296 |  | 2.05 | A_11_P0000033305 | acyl-CoA dehydrogenase, C-4 to C-12 straight chain | -2.15 |
| A_11_P0000020056 | baculoviral IAP repeat containing 5 | 2.05 | A_11_P0000038841 |  | -2.15 |
| A_11_P0000036517 |  | 2.05 | A_11_P0000031484 | poly (ADP-ribose) polymerase family, member 9 | -2.15 |
| A_11_P0000029371 | nuclear fragile X mental retardation protein interacting protein 1 | 2.05 | A_11_P108696 | G protein-coupled receptor 98 | -2.15 |
| A_11_P094856 |  | 2.05 | A_11_P0000010167 |  | -2.15 |
| A_11_P191218 | kinase insert domain receptor (a type III receptor tyrosine kinase) | 2.05 | A_11_P000009183 |  | -2.15 |
| A_11_P0000028767 | protein tyrosine phosphatase, receptor type, G | 2.05 | A_11_P148903 |  | -2.15 |
| A_11_P121866 | translocase of outer mitochondrial membrane 5 homolog (yeast) | 2.05 | A_11_P107926 | KIAA0513 ortholog | -2.15 |
| A_11_P0000016603 | gem (nuclear organelle) associated protein 2 | 2.05 | A_11_P087521 | transmembrane protein 150A | -2.15 |
| A_11_P0000020582 | Myb/SANT-like DNA-binding domain containing 3 | 2.04 | A_11_P0000032745 | cytochrome P450, family 2, subfamily J, polypeptide 2 | -2.15 |
| A_11_P111796 | centromere protein P | 2.04 | A_11_P0000025267 |  | -2.15 |
| A_11_P0000015134 |  | 2.04 | A_11_P000005901 |  | -2.15 |
| A_11_P0000035995 | spindle and kinetochore associated complex subunit 3 | 2.04 | A_11_P063076 | tankyrase, TRF1-interacting ankyrin-related ADP-ribose polymerase 2 | -2.15 |
| A_11_P111556 | COBW domain containing 2 | 2.04 | A_11_P0000031901 | WD repeat and FYVE domain containing 1 | -2.15 |
| A_11_P095456 | ALG2, alpha-1,3/1,6-mannosyltransferase | 2.04 | A_11_P00000493 | patatin-like phospholipase domain containing 2 | -2.15 |
| A_11_P188813 | adducin 3 (gamma) | 2.04 | A_11_P157073 | signal transducer and activator of transcription 3 (acute-phase response factor) | -2.15 |
| A_11_P000003760 | NADH dehydrogenase (ubiquinone) complex I, assembly factor 4 | 2.04 | A_11_P000009855 |  | -2.15 |
| A_11_P000002475 | hypoxia up-regulated 1 | 2.04 | A_11_P0000031499 | UBX domain protein 7 | -2.15 |
| A_11_P205323 | regulator of G-protein signaling 1 | 2.04 | A_11_P0000021665 | progesterone receptor membrane component 2 | -2.15 |
| A_11_P0000022921 | keratin 18 | 2.04 | A_11_P0000019119 |  | -2.15 |
| A_11_P099291 | regulator of G-protein signaling 7 | 2.04 | A_11_P0000023307 | erbb2 interacting protein | -2.15 |
| A_11_P052421 | aldolase C, fructose-bisphosphate | 2.04 | A_11_P141963 |  | -2.15 |
| A_11_P211423 | v-myb avian myeloblastosis viral oncogene homolog | 2.04 | A_11_P157328 |  | -2.14 |
| A_11_P052396 | peptide YY | 2.04 | A_11_P00000504 | tetratricopeptide repeat domain 3 | -2.14 |
| A_11_P085576 |  | 2.04 | A_11_P175873 | RNA binding motif protein 12 | -2.14 |
| A_11_P058651 | IQ motif containing B1 | 2.04 | A_11_P0000028762 | ataxin 7 | -2.14 |
| A_11_P053236 | BCL2-associated X protein | 2.04 | A_11_P0000027086 | platelet derived growth factor C | -2.14 |
| A_11_P0000023151 | minichromosome maintenance complex component 4 | 2.04 | A_11_P147653 | myosin, heavy chain 6, cardiac muscle, alpha | -2.14 |
| A_11_P215008 | inducible T-cell co-stimulator | 2.04 | A_11_P202313 | Rho family GTPase 2 | -2.14 |
| A_11_P0000016904 |  | 2.04 | A_11_P107126 | mesoderm induction early response 1, transcriptional regulator | -2.14 |
| A_11_P0000028413 |  | 2.04 | A_11_P0000041048 | Tax1 (human T-cell leukemia virus type I) binding protein 1 | -2.14 |
| A_11_P110826 | mitogen-activated protein kinase kinase kinase 4 | 2.04 | A_11_P0000040370 |  | -2.14 |
| A_11_P0000035940 |  | 2.04 | A_11_P0000033974 | keratin 33A | -2.14 |
| A_11_P085886 | oxysterol binding protein-like 8 | 2.04 | A_11_P086591 | kinase D-interacting substrate, 220kDa | -2.14 |
| A_11_P200133 |  | 2.04 | A_11_P0000013284 |  | -2.14 |
| A_11_P0000011079 | glycerol-3-phosphate acyltransferase, mitochondrial | 2.04 | A_11_P000009147 |  | -2.14 |
| A_11_P126161 | CDK5 regulatory subunit associated protein 1-like 1 | 2.04 | A_11_P199943 |  | -2.14 |
| A_11_P100236 | RING finger protein 113A-like | 2.04 | A_11_P077836 | transmembrane protein 140 | -2.14 |
| A_11_P0000036559 |  | 2.04 | A_11_P0000016563 |  | -2.14 |
| A_11_P171553 | Purkinje cell protein 4 like 1 | 2.04 | A_11_P165668 |  | -2.14 |
| A_11_P0000013881 |  | 2.04 | A_11_P0000039553 |  | -2.14 |
| A_11_P063611 | adducin 3 (gamma) | 2.04 | A_11_P0000019672 | adenosine A2b receptor | -2.14 |
| A_11_P000007302 |  | 2.04 | A_11_P106926 |  | -2.14 |
| A_11_P050356 | hypoxanthine phosphoribosyltransferase 1 | 2.04 | A_11_P0000014956 | tropomodulin 2 (neuronal) | -2.14 |
| A_11_P122466 |  | 2.04 | A_11_P0000010715 |  | -2.14 |
| A_11_P144043 |  | 2.04 | A_11_P0000019385 |  | -2.14 |
| A_11_P00000928 | lon peptidase 1, mitochondrial | 2.04 | A_11_P105276 | chromosome 4 open reading frame, human C5orf42 | -2.14 |
| A_11_P098246 | tubulin tyrosine ligase-like family, member 7 | 2.04 | A_11_P107246 | cytochrome P450, family 2, subfamily J, polypeptide 2 | -2.14 |
| A_11_P0000020169 | hydroxy-delta-5-steroid dehydrogenase, 3 beta- and steroid delta-isomerase 2 | 2.04 | A_11_P224508 |  | -2.14 |
| A_11_P00000221 |  | 2.04 | A_11_P101606 | G protein-coupled receptor 158 | -2.14 |
| A_11_P083151 | peptidase (mitochondrial processing) alpha | 2.04 | A_11_P113161 |  | -2.14 |
| A_11_P183868 | ribosomal protein L35 | 2.04 | A_11_P0000010350 |  | -2.14 |
| A_11_P0000026392 | ribosomal protein L7-like 1 | 2.03 | A_11_P0000016004 |  | -2.14 |
| A_11_P000006274 | ring finger protein 208 | 2.03 | A_11_P0000022371 | N(alpha)-acetyltransferase 16, NatA auxiliary subunit | -2.14 |
| A_11_P102426 | superkiller viralicidic activity 2-like 2 (S. cerevisiae) | 2.03 | A_11_P000007889 |  | -2.14 |
| A_11_P0000022280 | folate receptor 2 (fetal) | 2.03 | A_11_P0000017880 |  | -2.14 |
| A_11_P056221 | eukaryotic translation elongation factor 1 epsilon 1 | 2.03 | A_11_P115466 |  | -2.14 |
| A_11_P127851 | translocase of inner mitochondrial membrane 9 homolog (yeast) | 2.03 | A_11_P0000041231 |  | -2.14 |
| A_11_P108951 |  | 2.03 | A_11_P061446 | tetratricopeptide repeat domain 3 | -2.14 |
| A_11_P0000052 | death-domain associated protein | 2.03 | A_11_P0000025346 | neighbor of BRCA1 gene 1 | -2.14 |
| A_11_P0000040790 | protein arginine methyltransferase 1 | 2.03 | A_11_P0000019547 | histamine receptor H1 | -2.14 |
| A_11_P127586 | beta-defensin 103 | 2.03 | A_11_P177168 |  | -2.14 |
| A_11_P0000021829 | ring finger protein 146 | 2.03 | A_11_P0000040314 |  | -2.14 |
| A_11_P143948 | thioredoxin domain containing 5 (endoplasmic reticulum) | 2.03 | A_11_P0000027588 |  | -2.14 |
| A_11_P0000018101 |  | 2.03 | A_11_P0000021392 | lysine (K)-specific demethylase 3A | -2.13 |
| A_11_P0000020659 | TAF11 RNA polymerase II, TATA box binding protein (TBP)-associated factor, 28kDa | 2.03 | A_11_P081531 | heat shock protein, alpha-crystallin-related, B9 | -2.13 |
| A_11_P091881 | Ras-related GTP binding D | 2.03 | A_11_P071311 | SEC22 vesicle trafficking protein homolog C (S. cerevisiae) | -2.13 |
| A_11_P163748 | COP9 signalosome subunit 4 | 2.03 | A_11_P0000010515 |  | -2.13 |
| A_11_P000002684 | RAN, member RAS oncogene family | 2.03 | A_11_P051916 | chloride channel, voltage-sensitive 3 | -2.13 |
| A_11_P206633 | ribosomal protein S7 | 2.03 | A_11_P0000041646 | FBJ murine osteosarcoma viral oncogene homolog | -2.13 |
| A_11_P094441 | COMM domain containing 10 | 2.03 | A_11_P0000029332 | protein tyrosine phosphatase, non-receptor type 5 (striatum-enriched) | -2.13 |
| A_11_P0000021174 | nucleoporin 205kDa | 2.03 | A_11_P0000030990 | E2F transcription factor 2 | -2.13 |
| A_11_P000005846 | leucine zipper, putative tumor suppressor 2 | 2.03 | A_11_P000004631 |  | -2.13 |
| A_11_P090216 | B-cell CLL/lymphoma 11A (zinc finger protein) | 2.03 | A_11_P0000011073 |  | -2.13 |
| A_11_P0000016546 | GrpE-like 1, mitochondrial (E. coli) | 2.03 | A_11_P081421 | neighbor of BRCA1 gene 1 | -2.13 |
| A_11_P225203 | tumor necrosis factor (ligand) superfamily, member 9 | 2.03 | A_11_P0000033830 | potassium channel, subfamily K, member 10 | -2.13 |
| A_11_P102451 | SET domain containing 9 | 2.03 | A_11_P0000017333 |  | -2.13 |
| A_11_P0000040469 |  | 2.03 | A_11_P216168 |  | -2.13 |
| A_11_P0000024119 | WD repeat domain 61 | 2.03 | A_11_P204438 | potassium channel, subfamily K, member 5 | -2.13 |
| A_11_P00000484 | MTERF domain containing 1 | 2.03 | A_11_P154563 | dystonin | -2.13 |
| A_11_P0000024067 |  | 2.03 | A_11_P217833 |  | -2.13 |
| A_11_P118966 |  | 2.03 | A_11_P081811 | F-box and leucine-rich repeat protein 20 | -2.13 |
| A_11_P162018 | toll-like receptor 7 | 2.03 | A_11_P0000013874 | protein kinase C, epsilon | -2.13 |
| A_11_P0000023914 | metaxin 2 | 2.03 | A_11_P198038 | SPT2, Suppressor of Ty, domain containing 1 (S. cerevisiae) | -2.13 |
| A_11_P055746 | dual specificity phosphatase 12 | 2.03 | A_11_P099196 | basic leucine zipper nuclear factor 1 | -2.13 |
| A_11_P0000032619 | acetyl-CoA acetyltransferase 1 | 2.03 | A_11_P0000041182 |  | -2.13 |
| A_11_P0000030 | myc target 1 | 2.03 | A_11_P185933 |  | -2.13 |
| A_11_P0000041971 | cell death-inducing DFFA-like effector a | 2.03 | A_11_P183568 | FCH domain only 1 | -2.13 |
| A_11_P0000030192 | HIR histone cell cycle regulation defective homolog A (S. cerevisiae) | 2.03 | A_11_P0000033572 | oxysterol binding protein-like 1A | -2.13 |
| A_11_P175567 | eukaryotic translation elongation factor 1 alpha 1 | 2.03 | A_11_P0000014419 | ER degradation enhancer, mannosidase alpha-like 1 | -2.13 |
| A_11_P0000024927 | asp (abnormal spindle) homolog, microcephaly associated (Drosophila) | 2.02 | A_11_P156553 | splicing factor 3b, subunit 1, 155kDa | -2.13 |
| A_11_P0000014405 | F-box protein 21 | 2.02 | A_11_P183418 | spastic paraplegia 20 (Troyer syndrome) | -2.13 |
| A_11_P156293 |  | 2.02 | A_11_P071671 | protein phosphatase 2, regulatory subunit B'', alpha | -2.13 |
| A_11_P0000019723 | solute carrier family 46, member 2 | 2.02 | A_11_P0000029760 | molybdenum cofactor synthesis 3 | -2.13 |
| A_11_P186428 | elongator acetyltransferase complex subunit 2 | 2.02 | A_11_P0000015648 | COP9 signalosome subunit 3 | -2.13 |
| A_11_P0000032574 | dolichyl-phosphate (UDP-N-acetylglucosamine) N-acetylglucosaminephosphotransferase 1 (GlcNAc-1-P transferase) | 2.02 | A_11_P154023 |  | -2.13 |
| A_11_P0000039838 | Src-like-adaptor | 2.02 | A_11_P0000010016 |  | -2.13 |
| A_11_P149413 |  | 2.02 | A_11_P110206 | 3-ketodihydrosphingosine reductase | -2.13 |
| A_11_P086621 | tyrosine 3-monooxygenase/tryptophan 5-monooxygenase activation protein, theta polypeptide | 2.02 | A_11_P090631 | coiled-coil alpha-helical rod protein 1 | -2.13 |
| A_11_P107066 | COP9 signalosome subunit 3 | 2.02 | A_11_P214713 |  | -2.13 |
| A_11_P0000016927 |  | 2.02 | A_11_P00000777 |  | -2.13 |
| A_11_P0000021617 |  | 2.02 | A_11_P063676 | chromosome 28 open reading frame, human C10orf118 | -2.13 |
| A_11_P061501 |  | 2.02 | A_11_P0000029640 |  | -2.13 |
| A_11_P0000015180 |  | 2.02 | A_11_P068641 | UV radiation resistance associated | -2.13 |
| A_11_P156663 | zinc finger protein 653 | 2.02 | A_11_P062766 | semaphorin 7A, GPI membrane anchor (John Milton Hagen blood group) | -2.13 |
| A_11_P050546 | hepcidin antimicrobial peptide | 2.02 | A_11_P091451 | CD2-associated protein | -2.13 |
| A_11_P201883 | frizzled-related protein | 2.02 | A_11_P117426 |  | -2.13 |
| A_11_P109011 |  | 2.02 | A_11_P153588 | phosphatidylinositol transfer protein, alpha | -2.13 |
| A_11_P000006696 |  | 2.02 | A_11_P0000039426 | relaxin 1 | -2.13 |
| A_11_P0000015937 | lipoyl(octanoyl) transferase 2 (putative) | 2.02 | A_11_P0000023717 | solute carrier family 35, member A5 | -2.13 |
| A_11_P0000021567 | translocase of inner mitochondrial membrane 10 homolog (yeast) | 2.02 | A_11_P209473 |  | -2.12 |
| A_11_P0000017534 | steroid-5-alpha-reductase, alpha polypeptide 2 (3-oxo-5 alpha-steroid delta 4-dehydrogenase alpha 2) | 2.02 | A_11_P088386 |  | -2.12 |
| A_11_P075741 | TRAF interacting protein | 2.02 | A_11_P196483 |  | -2.12 |
| A_11_P0000019224 | RNA pseudouridylate synthase domain containing 1 | 2.02 | A_11_P0000024316 | SLU7 splicing factor homolog (S. cerevisiae) | -2.12 |
| A_11_P180898 |  | 2.02 | A_11_P0000013387 |  | -2.12 |
| A_11_P163218 |  | 2.02 | A_11_P065056 | interleukin 1 receptor accessory protein | -2.12 |
| A_11_P138171 |  | 2.02 | A_11_P151943 |  | -2.12 |
| A_11_P0000028789 | BRCA1 associated protein-1 (ubiquitin carboxy-terminal hydrolase) | 2.02 | A_11_P0000041019 | annexin A7 | -2.12 |
| A_11_P0000020916 | coatomer protein complex, subunit gamma 2 | 2.02 | A_11_P154943 | thioredoxin interacting protein | -2.12 |
| A_11_P084131 | squalene epoxidase | 2.02 | A_11_P0000039575 |  | -2.12 |
| A_11_P0000017902 |  | 2.02 | A_11_P0000012730 |  | -2.12 |
| A_11_P167413 | PRELI domain containing 1 | 2.02 | A_11_P0000033125 | transmembrane channel-like 5 | -2.12 |
| A_11_P150048 | dipeptidyl-peptidase 3 | 2.02 | A_11_P213423 | spermatogenesis associated, serine-rich 2-like | -2.12 |
| A_11_P00000789 | methylenetetrahydrofolate dehydrogenase (NADP+ dependent) 1, methenyltetrahydrofolate cyclohydrolase, formyltetrahydrofolate synthetase | 2.02 | A_11_P000009952 |  | -2.12 |
| A_11_P166578 | epithelial cell transforming sequence 2 oncogene | 2.02 | A_11_P096831 | general transcription factor IIIC, polypeptide 1, alpha 220kDa | -2.12 |
| A_11_P167868 | FtsJ RNA methyltransferase homolog 1 (E. coli) | 2.02 | A_11_P159443 | F-box protein 33 | -2.12 |
| A_11_P0000031099 | inositol-trisphosphate 3-kinase A | 2.02 | A_11_P0000038977 |  | -2.12 |
| A_11_P0000020459 |  | 2.02 | A_11_P0000024271 | annexin A7 | -2.12 |
| A_11_P194733 |  | 2.02 | A_11_P0000028895 | solute carrier family 35, member E1 | -2.12 |
| A_11_P0000016818 | F-box and leucine-rich repeat protein 16 | 2.02 | A_11_P181618 |  | -2.12 |
| A_11_P0000040437 | ribosomal protein L15 | 2.02 | A_11_P0000023800 | LIM domain containing preferred translocation partner in lipoma | -2.12 |
| A_11_P069601 | lactate dehydrogenase A | 2.01 | A_11_P0000023925 | sperm specific antigen 2 | -2.12 |
| A_11_P091056 | mitochondrial carrier 1 | 2.01 | A_11_P0000014391 | ATPase, aminophospholipid transporter (APLT), class I, type 8A, member 1 | -2.12 |
| A_11_P0000024081 | threonyl-tRNA synthetase-like 2 | 2.01 | A_11_P082216 |  | -2.12 |
| A_11_P0000016281 | like-glycosyltransferase | 2.01 | A_11_P0000026441 | protein tyrosine phosphatase type IVA, member 1 | -2.12 |
| A_11_P171833 | NADH dehydrogenase (ubiquinone) Fe-S protein 2, 49kDa (NADH-coenzyme Q reductase) | 2.01 | A_11_P119876 | testis-specific serine kinase 6 | -2.12 |
| A_11_P194828 |  | 2.01 | A_11_P0000032141 | toll-like receptor 10 | -2.12 |
| A_11_P101781 | Ras suppressor protein 1 | 2.01 | A_11_P0000023103 | soc-2 suppressor of clear homolog (C. elegans) | -2.12 |
| A_11_P0000031970 | phosphoprotein enriched in astrocytes 15 | 2.01 | A_11_P110696 | F-box protein 5 | -2.12 |
| A_11_P0000016259 |  | 2.01 | A_11_P099096 | dynamin 3 | -2.12 |
| A_11_P090656 | allograft inflammatory factor 1 | 2.01 | A_11_P0000026129 | interferon regulatory factor 1 | -2.12 |
| A_11_P0000039346 | glioblastoma amplified sequence | 2.01 | A_11_P000002407 |  | -2.12 |
| A_11_P150308 | NADH dehydrogenase (ubiquinone) flavoprotein 1, 51kDa | 2.01 | A_11_P162498 |  | -2.12 |
| A_11_P0000031051 | MAD2 mitotic arrest deficient-like 2 (yeast) | 2.01 | A_11_P0000029710 | v-maf avian musculoaponeurotic fibrosarcoma oncogene homolog B | -2.12 |
| A_11_P0000024972 | calcyclin binding protein | 2.01 | A_11_P128291 |  | -2.12 |
| A_11_P0000032891 | zinc finger homeobox 3 | 2.01 | A_11_P064561 | dpy-19-like 4 (C. elegans) | -2.12 |
| A_11_P105661 | roundabout, axon guidance receptor, homolog 3 (Drosophila) | 2.01 | A_11_P096791 | CD19 molecule | -2.12 |
| A_11_P0000024839 |  | 2.01 | A_11_P0000016410 | Ras association (RalGDS/AF-6) domain family member 5 | -2.12 |
| A_11_P050441 | signal peptidase complex subunit 3 homolog (S. cerevisiae) | 2.01 | A_11_P0000025321 | LIM domain containing 2 | -2.12 |
| A_11_P194403 | mediator complex subunit 16 | 2.01 | A_11_P060501 | proline rich 14-like | -2.12 |
| A_11_P0000028880 | UDP-GlcNAc:betaGal beta-1,3-N-acetylglucosaminyltransferase 3 | 2.01 | A_11_P0000014431 |  | -2.12 |
| A_11_P075046 |  | 2.01 | A_11_P0000025523 | GLE1 RNA export mediator | -2.12 |
| A_11_P0000029109 | R3H domain containing 4 | 2.01 | A_11_P115506 | KCNE1-like | -2.12 |
| A_11_P072211 | annexin A5 | 2.01 | A_11_P0000016466 | monoacylglycerol O-acyltransferase 3 | -2.12 |
| A_11_P102881 |  | 2.01 | A_11_P221018 |  | -2.12 |
| A_11_P200388 |  | 2.01 | A_11_P0000028155 | trace amine associated receptor 5 | -2.12 |
| A_11_P0000028784 | guanine nucleotide binding protein-like 3 (nucleolar) | 2.01 | A_11_P0000016562 | solute carrier family 37 (glucose-6-phosphate transporter), member 4 | -2.12 |
| A_11_P125721 |  | 2.01 | A_11_P0000019939 | cAMP responsive element modulator | -2.11 |
| A_11_P0000035087 | McKusick-Kaufman syndrome | 2.01 | A_11_P092716 | Rho GTPase activating protein 5 | -2.11 |
| A_11_P127846 | 40S ribosomal protein S2-like | 2.01 | A_11_P0000035621 | SID1 transmembrane family, member 2 | -2.11 |
| A_11_P0000034915 |  | 2.01 | A_11_P0000011507 |  | -2.11 |
| A_11_P0000041395 | zinc finger and BTB domain containing 47 | 2.01 | A_11_P188738 |  | -2.11 |
| A_11_P0000029687 | phosphatidylinositol glycan anchor biosynthesis, class U | 2.01 | A_11_P0000024744 |  | -2.11 |
| A_11_P00000523 | nucleosome assembly protein 1-like 4 | 2.01 | A_11_P0000015369 | N-myristoyltransferase 2 | -2.11 |
| A_11_P121856 | mitochondrial ribosomal protein 63 | 2.01 | A_11_P0000022096 | aminolevulinate, delta-, synthase 1 | -2.11 |
| A_11_P0000041599 |  | 2.01 | A_11_P072551 | methyl-CpG binding domain protein 5 | -2.11 |
| A_11_P166823 |  | 2.01 | A_11_P0000031238 | charged multivesicular body protein 2B | -2.11 |
| A_11_P092971 | cyclin-dependent kinase inhibitor 3 | 2.01 | A_11_P089276 | thyrotrophic embryonic factor | -2.11 |
| A_11_P000003363 |  | 2.01 | A_11_P0000029020 |  | -2.11 |
| A_11_P104636 | PRELI domain containing 1 | 2.01 | A_11_P0000010904 |  | -2.11 |
| A_11_P0000024455 | cholinergic receptor, nicotinic, epsilon (muscle) | 2.01 | A_11_P0000013433 |  | -2.11 |
| A_11_P00000326 | TELO2 interacting protein 2 | 2.01 | A_11_P00000355 | transmembrane protein 127 | -2.11 |
| A_11_P121361 | protein tyrosine phosphatase, receptor type, G | 2.01 | A_11_P215738 | zinc finger protein 598 | -2.11 |
| A_11_P057611 | NIF3 NGG1 interacting factor 3-like 1 (S. cerevisiae) | 2.01 | A_11_P0000019435 |  | -2.11 |
| A_11_P0000022023 | eukaryotic elongation factor, selenocysteine-tRNA-specific | 2.01 | A_11_P0000033649 | solute carrier family 7 (amino acid transporter light chain, L system), member 8 | -2.11 |
| A_11_P0000030698 | zinc finger, AN1-type domain 1 | 2.01 | A_11_P0000032928 |  | -2.11 |
| A_11_P112211 |  | 2.01 | A_11_P0000029105 | serine/threonine kinase 11 | -2.11 |
| A_11_P0000020472 | coiled-coil domain containing 112 | 2.01 | A_11_P124731 | CREB3 regulatory factor | -2.11 |
| A_11_P161703 |  | 2.01 | A_11_P150808 |  | -2.11 |
| A_11_P155188 |  | 2.01 | A_11_P083731 | tubulin tyrosine ligase-like family, member 11 | -2.11 |
| A_11_P0000039401 |  | 2.01 | A_11_P209173 |  | -2.11 |
| A_11_P00000592 | phosphatidylinositol glycan anchor biosynthesis, class M | 2.01 | A_11_P0000026206 | family with sequence similarity 166, member B | -2.11 |
| A_11_P0000018304 | exophilin 5 | 2.00 | A_11_P0000040748 |  | -2.11 |
| A_11_P0000023872 | RNA binding motif, single stranded interacting protein 1 | 2.00 | A_11_P0000014437 |  | -2.11 |
| A_11_P086406 | meiotic nuclear divisions 1 homolog (S. cerevisiae) | 2.00 | A_11_P0000027071 | SH3 domain containing 19 | -2.11 |
| A_11_P108001 | phospholipase C, gamma 2 (phosphatidylinositol-specific) | 2.00 | A_11_P0000032150 | sel-1 suppressor of lin-12-like 3 (C. elegans) | -2.11 |
| A_11_P0000026829 | mitochondrial assembly of ribosomal large subunit 1 | 2.00 | A_11_P057976 |  | -2.11 |
| A_11_P149673 | zinc finger protein 32 | 2.00 | A_11_P059276 | family with sequence similarity 190, member A | -2.11 |
| A_11_P185138 |  | 2.00 | A_11_P113861 |  | -2.11 |
| A_11_P000005679 |  | 2.00 | A_11_P122341 |  | -2.11 |
| A_11_P0000030672 | proteasome subunit alpha type-1-like | 2.00 | A_11_P0000028181 | F-box protein 30 | -2.11 |
| A_11_P199953 | RNA binding protein, fox-1 homolog (C. elegans) 2 | 2.00 | A_11_P0000010010 |  | -2.11 |
| A_11_P181658 |  | 2.00 | A_11_P175298 |  | -2.11 |
|  |  |  | A_11_P0000040886 | signal transducer and activator of transcription 3 (acute-phase response factor) | -2.11 |
|  |  |  | A_11_P0000015385 |  | -2.11 |
|  |  |  | A_11_P082881 | reticulon 4 receptor-like 1 | -2.10 |
|  |  |  | A_11_P218923 | poly(A) polymerase gamma | -2.10 |
|  |  |  | A_11_P210533 |  | -2.10 |
|  |  |  | A_11_P117581 |  | -2.10 |
|  |  |  | A_11_P176998 |  | -2.10 |
|  |  |  | A_11_P0000016458 |  | -2.10 |
|  |  |  | A_11_P144423 |  | -2.10 |
|  |  |  | A_11_P0000039329 |  | -2.10 |
|  |  |  | A_11_P0000031673 | ataxin 1 | -2.10 |
|  |  |  | A_11_P000002490 |  | -2.10 |
|  |  |  | A_11_P0000016749 |  | -2.10 |
|  |  |  | A_11_P0000026960 | EF-hand calcium binding domain 14 | -2.10 |
|  |  |  | A_11_P0000022988 | epidermal growth factor receptor pathway substrate 8 | -2.10 |
|  |  |  | A_11_P139536 |  | -2.10 |
|  |  |  | A_11_P215788 | neighbor of BRCA1 gene 1 | -2.10 |
|  |  |  | A_11_P085106 |  | -2.10 |
|  |  |  | A_11_P106471 | rabaptin, RAB GTPase binding effector protein 1 | -2.10 |
|  |  |  | A_11_P166413 |  | -2.10 |
|  |  |  | A_11_P0000021233 | fibroblast growth factor receptor 1 | -2.10 |
|  |  |  | A_11_P0000018782 |  | -2.10 |
|  |  |  | A_11_P0000015376 |  | -2.10 |
|  |  |  | A_11_P0000016122 |  | -2.10 |
|  |  |  | A_11_P205093 | protein inhibitor of activated STAT, 1 | -2.10 |
|  |  |  | A_11_P125331 | platelet-activating factor acetylhydrolase 1b, regulatory subunit 1 (45kDa) | -2.10 |
|  |  |  | A_11_P000008487 |  | -2.10 |
|  |  |  | A_11_P169788 | protein phosphatase 4, regulatory subunit 1 | -2.10 |
|  |  |  | A_11_P000009273 |  | -2.10 |
|  |  |  | A_11_P0000033426 | methyltransferase like 11B | -2.10 |
|  |  |  | A_11_P186943 | potassium channel, subfamily K, member 5 | -2.10 |
|  |  |  | A_11_P0000014388 |  | -2.10 |
|  |  |  | A_11_P171583 |  | -2.10 |
|  |  |  | A_11_P201083 | serine/arginine repetitive matrix 2 | -2.10 |
|  |  |  | A_11_P137211 |  | -2.10 |
|  |  |  | A_11_P0000016649 |  | -2.10 |
|  |  |  | A_11_P0000022827 | disintegrin and metalloproteinase domain-containing protein 1a-like | -2.10 |
|  |  |  | A_11_P067281 |  | -2.10 |
|  |  |  | A_11_P000008055 |  | -2.10 |
|  |  |  | A_11_P152163 |  | -2.10 |
|  |  |  | A_11_P147963 |  | -2.10 |
|  |  |  | A_11_P169333 |  | -2.10 |
|  |  |  | A_11_P0000014711 | Ras association (RalGDS/AF-6) domain family member 3 | -2.10 |
|  |  |  | A_11_P161013 | PBX/knotted 1 homeobox 1 | -2.10 |
|  |  |  | A_11_P0000029070 | amyloid beta (A4) precursor protein-binding, family A, member 3 | -2.10 |
|  |  |  | A_11_P0000020016 | zona pellucida glycoprotein 2 (sperm receptor) | -2.10 |
|  |  |  | A_11_P000007013 |  | -2.10 |
|  |  |  | A_11_P082721 | abhydrolase domain containing 15 | -2.10 |
|  |  |  | A_11_P090686 | lymphocyte antigen 6 complex, locus G5B | -2.09 |
|  |  |  | A_11_P000008369 |  | -2.09 |
|  |  |  | A_11_P144753 |  | -2.09 |
|  |  |  | A_11_P097396 | CREB binding protein | -2.09 |
|  |  |  | A_11_P000008013 |  | -2.09 |
|  |  |  | A_11_P197978 | nudix (nucleoside diphosphate linked moiety X)-type motif 4 | -2.09 |
|  |  |  | A_11_P124011 | family with sequence similarity 76, member B | -2.09 |
|  |  |  | A_11_P0000019267 | transmembrane protein 231 | -2.09 |
|  |  |  | A_11_P0000025477 | platelet-activating factor acetylhydrolase 1b, regulatory subunit 1 (45kDa) | -2.09 |
|  |  |  | A_11_P0000012426 |  | -2.09 |
|  |  |  | A_11_P151433 | ubiquitin-like modifier activating enzyme 7 | -2.09 |
|  |  |  | A_11_P197773 | attractin | -2.09 |
|  |  |  | A_11_P0000014697 | protein tyrosine phosphatase, receptor type, F | -2.09 |
|  |  |  | A_11_P0000024050 | coatomer protein complex, subunit alpha | -2.09 |
|  |  |  | A_11_P0000010418 |  | -2.09 |
|  |  |  | A_11_P206858 | ORM1-like 3 (S. cerevisiae) | -2.09 |
|  |  |  | A_11_P0000032190 | glutaredoxin (thioltransferase) | -2.09 |
|  |  |  | A_11_P063196 | ligand dependent nuclear receptor corepressor | -2.09 |
|  |  |  | A_11_P0000019767 | caspase 3, apoptosis-related cysteine peptidase | -2.09 |
|  |  |  | A_11_P0000040118 | chromosome 11 open reading frame, human C5orf45 | -2.09 |
|  |  |  | A_11_P0000039469 |  | -2.09 |
|  |  |  | A_11_P000005910 |  | -2.09 |
|  |  |  | A_11_P058676 | family with sequence similarity 162, member A | -2.09 |
|  |  |  | A_11_P062091 |  | -2.09 |
|  |  |  | A_11_P182208 |  | -2.09 |
|  |  |  | A_11_P0000032281 | tetraspanin 15 | -2.09 |
|  |  |  | A_11_P072151 |  | -2.09 |
|  |  |  | A_11_P209493 | solute carrier family 3 (cystine, dibasic and neutral amino acid transporters, activator of cystine, dibasic and neutral amino acid transport), member 1 | -2.09 |
|  |  |  | A_11_P086361 | serine protease 27-like | -2.09 |
|  |  |  | A_11_P164733 | GC-rich promoter binding protein 1 | -2.09 |
|  |  |  | A_11_P199783 | lamin A/C | -2.09 |
|  |  |  | A_11_P110586 | SNF2 histone linker PHD RING helicase, E3 ubiquitin protein ligase | -2.09 |
|  |  |  | A_11_P056951 | solute carrier family 4, sodium bicarbonate transporter, member 10 | -2.09 |
|  |  |  | A_11_P000005561 |  | -2.09 |
|  |  |  | A_11_P148803 | NEDD4 binding protein 1 | -2.09 |
|  |  |  | A_11_P0000024425 | PIH1 domain containing 2 | -2.09 |
|  |  |  | A_11_P212873 |  | -2.09 |
|  |  |  | A_11_P0000015964 |  | -2.09 |
|  |  |  | A_11_P0000024913 |  | -2.09 |
|  |  |  | A_11_P000006526 |  | -2.09 |
|  |  |  | A_11_P0000015756 |  | -2.09 |
|  |  |  | A_11_P0000019494 |  | -2.09 |
|  |  |  | A_11_P0000016125 |  | -2.09 |
|  |  |  | A_11_P215828 | YTH domain family, member 3 | -2.09 |
|  |  |  | A_11_P0000017629 |  | -2.09 |
|  |  |  | A_11_P0000040291 |  | -2.09 |
|  |  |  | A_11_P162628 |  | -2.09 |
|  |  |  | A_11_P087876 | prostaglandin F2 receptor inhibitor | -2.09 |
|  |  |  | A_11_P0000023636 | mitogen-activated protein kinase 10 | -2.09 |
|  |  |  | A_11_P0000029736 | zinc finger, SWIM-type containing 3 | -2.09 |
|  |  |  | A_11_P122441 |  | -2.09 |
|  |  |  | A_11_P0000014346 |  | -2.09 |
|  |  |  | A_11_P000003611 |  | -2.09 |
|  |  |  | A_11_P000002403 |  | -2.09 |
|  |  |  | A_11_P0000032180 | diphosphoinositol pentakisphosphate kinase 2 | -2.09 |
|  |  |  | A_11_P139801 |  | -2.08 |
|  |  |  | A_11_P0000013586 |  | -2.08 |
|  |  |  | A_11_P094631 | folliculin interacting protein 1 | -2.08 |
|  |  |  | A_11_P000001030 |  | -2.08 |
|  |  |  | A_11_P077381 | histocompatibility (minor) HA-1 | -2.08 |
|  |  |  | A_11_P210148 | NUAK family, SNF1-like kinase, 2 | -2.08 |
|  |  |  | A_11_P0000032863 | v-maf avian musculoaponeurotic fibrosarcoma oncogene homolog | -2.08 |
|  |  |  | A_11_P0000010964 |  | -2.08 |
|  |  |  | A_11_P0000019313 |  | -2.08 |
|  |  |  | A_11_P0000027604 | solute carrier family 9, subfamily A (NHE3, cation proton antiporter 3), member 3 regulator 1 | -2.08 |
|  |  |  | A_11_P134256 |  | -2.08 |
|  |  |  | A_11_P083986 | trichorhinophalangeal syndrome I | -2.08 |
|  |  |  | A_11_P118811 | nudix (nucleoside diphosphate linked moiety X)-type motif 3 | -2.08 |
|  |  |  | A_11_P059356 | microsomal triglyceride transfer protein | -2.08 |
|  |  |  | A_11_P0000020029 | RAB5A, member RAS oncogene family | -2.08 |
|  |  |  | A_11_P0000034757 | poly(A) binding protein, cytoplasmic 5 | -2.08 |
|  |  |  | A_11_P169698 |  | -2.08 |
|  |  |  | A_11_P0000027630 | galanin receptor 2 | -2.08 |
|  |  |  | A_11_P151358 |  | -2.08 |
|  |  |  | A_11_P067361 |  | -2.08 |
|  |  |  | A_11_P0000026170 | alkaline ceramidase 2 | -2.08 |
|  |  |  | A_11_P000009997 |  | -2.08 |
|  |  |  | A_11_P000006171 |  | -2.08 |
|  |  |  | A_11_P174958 |  | -2.08 |
|  |  |  | A_11_P199593 |  | -2.08 |
|  |  |  | A_11_P000007503 |  | -2.08 |
|  |  |  | A_11_P0000026168 |  | -2.08 |
|  |  |  | A_11_P216883 | beaded filament structural protein 2, phakinin | -2.08 |
|  |  |  | A_11_P136281 |  | -2.08 |
|  |  |  | A_11_P201223 | aquaporin 8 | -2.08 |
|  |  |  | A_11_P066261 | C-type lectin domain family 9, member A | -2.08 |
|  |  |  | A_11_P134456 |  | -2.08 |
|  |  |  | A_11_P155633 | REC8 meiotic recombination protein | -2.08 |
|  |  |  | A_11_P0000041283 |  | -2.08 |
|  |  |  | A_11_P000005053 |  | -2.08 |
|  |  |  | A_11_P0000036 | ubiquitin protein ligase E3 component n-recognin 5 | -2.08 |
|  |  |  | A_11_P0000020277 | Rho GTPase activating protein 9 | -2.08 |
|  |  |  | A_11_P0000018399 |  | -2.08 |
|  |  |  | A_11_P0000033507 | zinc finger and BTB domain containing 7B | -2.08 |
|  |  |  | A_11_P127296 |  | -2.08 |
|  |  |  | A_11_P0000015980 |  | -2.08 |
|  |  |  | A_11_P0000016993 | inositol 1,4,5-trisphosphate receptor, type 3 | -2.08 |
|  |  |  | A_11_P169493 | coagulation factor XI | -2.08 |
|  |  |  | A_11_P0000016311 | chromosome 12 open reading frame, human C6orf106 | -2.08 |
|  |  |  | A_11_P0000018932 |  | -2.08 |
|  |  |  | A_11_P0000025116 | golgin A5 | -2.08 |
|  |  |  | A_11_P202603 |  | -2.08 |
|  |  |  | A_11_P000009760 |  | -2.08 |
|  |  |  | A_11_P061836 | formin 1 | -2.08 |
|  |  |  | A_11_P123621 |  | -2.08 |
|  |  |  | A_11_P079526 |  | -2.08 |
|  |  |  | A_11_P000003938 |  | -2.08 |
|  |  |  | A_11_P153888 | dynamin 2 | -2.07 |
|  |  |  | A_11_P066456 | TAP binding protein-like | -2.07 |
|  |  |  | A_11_P0000032256 |  | -2.07 |
|  |  |  | A_11_P000007815 |  | -2.07 |
|  |  |  | A_11_P0000017228 |  | -2.07 |
|  |  |  | A_11_P000008297 |  | -2.07 |
|  |  |  | A_11_P056131 | PX domain containing 1 | -2.07 |
|  |  |  | A_11_P208828 | NODAL modulator 1 | -2.07 |
|  |  |  | A_11_P0000015459 | valyl-tRNA synthetase | -2.07 |
|  |  |  | A_11_P000006442 |  | -2.07 |
|  |  |  | A_11_P0000011932 |  | -2.07 |
|  |  |  | A_11_P218833 | chimerin 2 | -2.07 |
|  |  |  | A_11_P0000017256 |  | -2.07 |
|  |  |  | A_11_P213768 | remodeling and spacing factor 1 | -2.07 |
|  |  |  | A_11_P0000032859 | beta-carotene 15,15'-monooxygenase 1 | -2.07 |
|  |  |  | A_11_P064361 | zinc finger homeobox 4 | -2.07 |
|  |  |  | A_11_P0000010360 | polyglutamine binding protein 1 | -2.07 |
|  |  |  | A_11_P0000018814 | vascular endothelial growth factor A | -2.07 |
|  |  |  | A_11_P084486 |  | -2.07 |
|  |  |  | A_11_P0000013891 | sodium channel, voltage-gated, type II, beta subunit | -2.07 |
|  |  |  | A_11_P137686 | G protein-coupled receptor 21 | -2.07 |
|  |  |  | A_11_P107731 | solute carrier family 2 (facilitated glucose/fructose transporter), member 5 | -2.07 |
|  |  |  | A_11_P000001681 |  | -2.07 |
|  |  |  | A_11_P0000021359 | atlastin GTPase 2 | -2.07 |
|  |  |  | A_11_P197383 |  | -2.07 |
|  |  |  | A_11_P0000017659 |  | -2.07 |
|  |  |  | A_11_P066176 | KIAA1467 ortholog | -2.07 |
|  |  |  | A_11_P0000010581 |  | -2.07 |
|  |  |  | A_11_P092806 | trafficking protein particle complex 6B | -2.07 |
|  |  |  | A_11_P0000025874 | myo-inositol oxygenase | -2.07 |
|  |  |  | A_11_P088696 | helicase (DNA) B | -2.07 |
|  |  |  | A_11_P188513 | serine/threonine kinase 38 like | -2.07 |
|  |  |  | A_11_P0000015184 |  | -2.07 |
|  |  |  | A_11_P154403 | solute carrier family 35, member A5 | -2.07 |
|  |  |  | A_11_P087441 | eukaryotic translation initiation factor 2-alpha kinase 3 | -2.07 |
|  |  |  | A_11_P186383 |  | -2.07 |
|  |  |  | A_11_P0000028845 | LIM domains containing 1 | -2.07 |
|  |  |  | A_11_P158573 | ariadne homolog, ubiquitin-conjugating enzyme E2 binding protein, 1 (Drosophila) | -2.07 |
|  |  |  | A_11_P0000028513 | glioma tumor suppressor candidate region gene 1 | -2.07 |
|  |  |  | A_11_P086936 | solute carrier family 30 (zinc transporter), member 3 | -2.07 |
|  |  |  | A_11_P154753 |  | -2.07 |
|  |  |  | A_11_P097361 | chromosome 6 open reading frame, human C16orf5 | -2.07 |
|  |  |  | A_11_P188698 |  | -2.07 |
|  |  |  | A_11_P0000017346 |  | -2.07 |
|  |  |  | A_11_P077061 | toll-like receptor adaptor molecule 1 | -2.07 |
|  |  |  | A_11_P0000031457 | KIAA2018 ortholog | -2.07 |
|  |  |  | A_11_P111111 |  | -2.07 |
|  |  |  | A_11_P072306 | mitogen-activated protein kinase kinase kinase 2 | -2.06 |
|  |  |  | A_11_P0000015565 |  | -2.06 |
|  |  |  | A_11_P212618 | nudix (nucleoside diphosphate linked moiety X)-type motif 4 | -2.06 |
|  |  |  | A_11_P0000019308 |  | -2.06 |
|  |  |  | A_11_P0000020431 | proteasome (prosome, macropain) activator subunit 4 | -2.06 |
|  |  |  | A_11_P0000010775 |  | -2.06 |
|  |  |  | A_11_P172098 |  | -2.06 |
|  |  |  | A_11_P124876 | ELAV (embryonic lethal, abnormal vision, Drosophila)-like 4 | -2.06 |
|  |  |  | A_11_P181883 |  | -2.06 |
|  |  |  | A_11_P055651 | dimethylaniline monooxygenase [N-oxide-forming] 5-like | -2.06 |
|  |  |  | A_11_P088776 | leucine-rich repeat containing G protein-coupled receptor 5 | -2.06 |
|  |  |  | A_11_P106626 |  | -2.06 |
|  |  |  | A_11_P0000012146 |  | -2.06 |
|  |  |  | A_11_P000006034 |  | -2.06 |
|  |  |  | A_11_P159043 |  | -2.06 |
|  |  |  | A_11_P061096 |  | -2.06 |
|  |  |  | A_11_P094616 | CDC42 small effector 2 | -2.06 |
|  |  |  | A_11_P175578 |  | -2.06 |
|  |  |  | A_11_P075746 | ubiquitin-like modifier activating enzyme 7 | -2.06 |
|  |  |  | A_11_P000008045 |  | -2.06 |
|  |  |  | A_11_P077676 |  | -2.06 |
|  |  |  | A_11_P0000013583 |  | -2.06 |
|  |  |  | A_11_P0000017356 |  | -2.06 |
|  |  |  | A_11_P0000018995 |  | -2.06 |
|  |  |  | A_11_P064021 | olfactory receptor 226-like | -2.06 |
|  |  |  | A_11_P000008135 |  | -2.06 |
|  |  |  | A_11_P098131 | guanylate binding protein 1, interferon-inducible | -2.06 |
|  |  |  | A_11_P171923 |  | -2.06 |
|  |  |  | A_11_P0000025576 | uridine phosphorylase 1-like | -2.06 |
|  |  |  | A_11_P0000027349 | dihydropyrimidinase-like 5 | -2.06 |
|  |  |  | A_11_P0000014536 |  | -2.06 |
|  |  |  | A_11_P0000033762 |  | -2.06 |
|  |  |  | A_11_P0000021551 | F-box protein 3 | -2.06 |
|  |  |  | A_11_P0000022578 |  | -2.06 |
|  |  |  | A_11_P0000039592 | coiled-coil alpha-helical rod protein 1 | -2.06 |
|  |  |  | A_11_P0000027806 | olfactory receptor 1052-like | -2.06 |
|  |  |  | A_11_P0000028231 | myeloid/lymphoid or mixed-lineage leukemia (trithorax homolog, Drosophila); translocated to, 4 | -2.06 |
|  |  |  | A_11_P092546 | interferon regulatory factor 9 | -2.05 |
|  |  |  | A_11_P000005320 |  | -2.05 |
|  |  |  | A_11_P0000021520 | hematological and neurological expressed 1 | -2.05 |
|  |  |  | A_11_P0000022863 |  | -2.05 |
|  |  |  | A_11_P0000017350 |  | -2.05 |
|  |  |  | A_11_P068686 | sialidase 3 (membrane sialidase) | -2.05 |
|  |  |  | A_11_P0000020480 | zinc finger protein 354A | -2.05 |
|  |  |  | A_11_P0000033965 | keratin 17 | -2.05 |
|  |  |  | A_11_P054426 |  | -2.05 |
|  |  |  | A_11_P0000040811 |  | -2.05 |
|  |  |  | A_11_P062021 | phospholipase A2, group IVF | -2.05 |
|  |  |  | A_11_P0000038742 |  | -2.05 |
|  |  |  | A_11_P191328 |  | -2.05 |
|  |  |  | A_11_P102951 | integrin alpha FG-GAP repeat containing 1 | -2.05 |
|  |  |  | A_11_P153303 | mitogen-activated protein kinase 8 interacting protein 3 | -2.05 |
|  |  |  | A_11_P0000021599 | glutathione S-transferase P-like | -2.05 |
|  |  |  | A_11_P0000037 | antizyme inhibitor 1 | -2.05 |
|  |  |  | A_11_P0000010268 |  | -2.05 |
|  |  |  | A_11_P0000038731 |  | -2.05 |
|  |  |  | A_11_P054061 | v-ets avian erythroblastosis virus E26 oncogene homolog 1 | -2.05 |
|  |  |  | A_11_P00000183 | zona pellucida binding protein | -2.05 |
|  |  |  | A_11_P0000018401 | cadherin 1, type 1, E-cadherin (epithelial) | -2.05 |
|  |  |  | A_11_P209543 | intersectin 1 (SH3 domain protein) | -2.05 |
|  |  |  | A_11_P0000015966 |  | -2.05 |
|  |  |  | A_11_P0000031614 | polyhomeotic homolog 3 (Drosophila) | -2.05 |
|  |  |  | A_11_P000009903 |  | -2.05 |
|  |  |  | A_11_P140571 |  | -2.05 |
|  |  |  | A_11_P0000030116 | tetratricopeptide repeat domain 28 | -2.05 |
|  |  |  | A_11_P082551 | cytokine receptor-like factor 3 | -2.05 |
|  |  |  | A_11_P000007354 |  | -2.05 |
|  |  |  | A_11_P0000014299 |  | -2.05 |
|  |  |  | A_11_P0000033624 | putative olfactory receptor 2W6-like | -2.05 |
|  |  |  | A_11_P167078 |  | -2.05 |
|  |  |  | A_11_P0000019936 | tribbles homolog 2 (Drosophila) | -2.05 |
|  |  |  | A_11_P0000010865 |  | -2.05 |
|  |  |  | A_11_P000003821 |  | -2.05 |
|  |  |  | A_11_P0000015183 |  | -2.05 |
|  |  |  | A_11_P0000021235 | Wolf-Hirschhorn syndrome candidate 1-like 1 | -2.05 |
|  |  |  | A_11_P054081 | proteoglycan 4 | -2.05 |
|  |  |  | A_11_P0000028507 |  | -2.05 |
|  |  |  | A_11_P091516 | progestin and adipoQ receptor family member VIII | -2.05 |
|  |  |  | A_11_P208993 |  | -2.05 |
|  |  |  | A_11_P173498 | CASP8 and FADD-like apoptosis regulator | -2.05 |
|  |  |  | A_11_P000007774 |  | -2.05 |
|  |  |  | A_11_P0000027473 | mannosidase, alpha, class 1A, member 2 | -2.05 |
|  |  |  | A_11_P000001493 | phospholipid hydroperoxide glutathione peroxidase | -2.05 |
|  |  |  | A_11_P095881 | autism susceptibility candidate 2 | -2.05 |
|  |  |  | A_11_P217353 | TAO kinase 1 | -2.05 |
|  |  |  | A_11_P184923 | NHL repeat containing 2 | -2.04 |
|  |  |  | A_11_P157798 | phosphatidylinositol 4-kinase, catalytic, alpha | -2.04 |
|  |  |  | A_11_P000009375 |  | -2.04 |
|  |  |  | A_11_P170283 | plectin | -2.04 |
|  |  |  | A_11_P000005948 |  | -2.04 |
|  |  |  | A_11_P0000017505 | paired related homeobox 1 | -2.04 |
|  |  |  | A_11_P000001329 |  | -2.04 |
|  |  |  | A_11_P0000027217 | WD repeat domain 60 | -2.04 |
|  |  |  | A_11_P0000017285 |  | -2.04 |
|  |  |  | A_11_P0000038329 |  | -2.04 |
|  |  |  | A_11_P000007018 |  | -2.04 |
|  |  |  | A_11_P000007388 |  | -2.04 |
|  |  |  | A_11_P151023 |  | -2.04 |
|  |  |  | A_11_P000006921 |  | -2.04 |
|  |  |  | A_11_P0000017582 |  | -2.04 |
|  |  |  | A_11_P00000667 |  | -2.04 |
|  |  |  | A_11_P0000027865 |  | -2.04 |
|  |  |  | A_11_P178448 |  | -2.04 |
|  |  |  | A_11_P086751 | pumilio RNA-binding family member 2 | -2.04 |
|  |  |  | A_11_P000006839 |  | -2.04 |
|  |  |  | A_11_P161743 |  | -2.04 |
|  |  |  | A_11_P0000024828 | WD repeat domain 90 | -2.04 |
|  |  |  | A_11_P191733 | inositol polyphosphate-5-phosphatase, 72 kDa | -2.04 |
|  |  |  | A_11_P0000027628 | cyclin-dependent kinase 3 | -2.04 |
|  |  |  | A_11_P000007321 |  | -2.04 |
|  |  |  | A_11_P0000037678 |  | -2.04 |
|  |  |  | A_11_P0000028335 | bicaudal D homolog 2 (Drosophila) | -2.04 |
|  |  |  | A_11_P100331 | protein phosphatase 4, regulatory subunit 1 | -2.04 |
|  |  |  | A_11_P149088 |  | -2.04 |
|  |  |  | A_11_P000006760 |  | -2.04 |
|  |  |  | A_11_P093451 | ribosomal protein S6 kinase-like 1 | -2.04 |
|  |  |  | A_11_P199258 | Mov10, Moloney leukemia virus 10, homolog (mouse) | -2.04 |
|  |  |  | A_11_P0000029946 | thiamine transporter 2-like | -2.04 |
|  |  |  | A_11_P089406 | DEAD (Asp-Glu-Ala-Asp) box helicase 17 | -2.04 |
|  |  |  | A_11_P162893 |  | -2.04 |
|  |  |  | A_11_P0000020802 | ring finger protein 19A, E3 ubiquitin protein ligase | -2.04 |
|  |  |  | A_11_P099011 | pappalysin 2 | -2.04 |
|  |  |  | A_11_P0000016824 | neurocalcin delta | -2.04 |
|  |  |  | A_11_P0000021053 |  | -2.04 |
|  |  |  | A_11_P0000011332 |  | -2.04 |
|  |  |  | A_11_P0000027085 | cathepsin O | -2.04 |
|  |  |  | A_11_P142418 | serum amyloid A1 | -2.04 |
|  |  |  | A_11_P133808 | coronin 6 | -2.04 |
|  |  |  | A_11_P0000023900 | cytochrome b reductase 1 | -2.04 |
|  |  |  | A_11_P000007859 |  | -2.04 |
|  |  |  | A_11_P000006351 |  | -2.04 |
|  |  |  | A_11_P0000033279 | leucine rich repeat containing 8 family, member C | -2.04 |
|  |  |  | A_11_P152428 |  | -2.04 |
|  |  |  | A_11_P0000019742 | myeloid cell leukemia sequence 1 (BCL2-related) | -2.04 |
|  |  |  | A_11_P00000922 | KIAA0430 ortholog | -2.04 |
|  |  |  | A_11_P000009144 |  | -2.04 |
|  |  |  | A_11_P0000012826 |  | -2.04 |
|  |  |  | A_11_P159778 | homer homolog 3 (Drosophila) | -2.04 |
|  |  |  | A_11_P161123 | oxysterol binding protein-like 1A | -2.04 |
|  |  |  | A_11_P0000014278 |  | -2.04 |
|  |  |  | A_11_P0000038843 |  | -2.04 |
|  |  |  | A_11_P0000026004 | sema domain, immunoglobulin domain (Ig), transmembrane domain (TM) and short cytoplasmic domain, (semaphorin) 4C | -2.04 |
|  |  |  | A_11_P087801 | tripartite motif containing 33 | -2.04 |
|  |  |  | A_11_P184213 | laminin, beta 2 (laminin S) | -2.03 |
|  |  |  | A_11_P096141 |  | -2.03 |
|  |  |  | A_11_P0000024350 | selenoprotein P, plasma, 1 | -2.03 |
|  |  |  | A_11_P0000025930 | transmembrane protein 184B | -2.03 |
|  |  |  | A_11_P0000013807 |  | -2.03 |
|  |  |  | A_11_P0000012720 |  | -2.03 |
|  |  |  | A_11_P0000011475 |  | -2.03 |
|  |  |  | A_11_P000009127 |  | -2.03 |
|  |  |  | A_11_P0000033576 | GATA binding protein 6 | -2.03 |
|  |  |  | A_11_P0000029219 | olfactory receptor 52I2-like | -2.03 |
|  |  |  | A_11_P0000019472 |  | -2.03 |
|  |  |  | A_11_P203513 | zinc finger, C3H1-type containing | -2.03 |
|  |  |  | A_11_P107261 |  | -2.03 |
|  |  |  | A_11_P0000017343 |  | -2.03 |
|  |  |  | A_11_P0000018632 |  | -2.03 |
|  |  |  | A_11_P109611 | leucine aminopeptidase 3 | -2.03 |
|  |  |  | A_11_P158598 |  | -2.03 |
|  |  |  | A_11_P0000010609 |  | -2.03 |
|  |  |  | A_11_P0000038491 |  | -2.03 |
|  |  |  | A_11_P000007605 |  | -2.03 |
|  |  |  | A_11_P000009016 |  | -2.03 |
|  |  |  | A_11_P091996 | absent in melanoma 1 | -2.03 |
|  |  |  | A_11_P0000040394 |  | -2.03 |
|  |  |  | A_11_P074061 | suppressor of variegation 4-20 homolog 1 (Drosophila) | -2.03 |
|  |  |  | A_11_P0000027167 | jumonji C domain containing histone demethylase 1 homolog D (S. cerevisiae) | -2.03 |
|  |  |  | A_11_P0000021443 | PDZ domain containing 1 | -2.03 |
|  |  |  | A_11_P0000011530 |  | -2.03 |
|  |  |  | A_11_P0000018721 |  | -2.03 |
|  |  |  | A_11_P079316 | alkylglycerol monooxygenase | -2.03 |
|  |  |  | A_11_P0000013116 |  | -2.03 |
|  |  |  | A_11_P0000028725 | solute carrier family 6 (neurotransmitter transporter), member 1 | -2.03 |
|  |  |  | A_11_P131426 | casein kinase 1, gamma 1 | -2.03 |
|  |  |  | A_11_P0000014214 |  | -2.03 |
|  |  |  | A_11_P000002665 |  | -2.03 |
|  |  |  | A_11_P0000025632 | phosphorylase kinase, alpha 2 (liver) | -2.03 |
|  |  |  | A_11_P051091 | hematological and neurological expressed 1 | -2.03 |
|  |  |  | A_11_P0000027055 | growth arrest-specific 2 like 3 | -2.03 |
|  |  |  | A_11_P0000039278 |  | -2.03 |
|  |  |  | A_11_P0000011699 |  | -2.03 |
|  |  |  | A_11_P000004517 |  | -2.03 |
|  |  |  | A_11_P100161 | Niemann-Pick disease, type C1 | -2.03 |
|  |  |  | A_11_P0000013464 |  | -2.03 |
|  |  |  | A_11_P0000023536 | zinc finger protein 609 | -2.03 |
|  |  |  | A_11_P0000018959 |  | -2.03 |
|  |  |  | A_11_P0000024554 | sterile alpha motif domain containing 11 | -2.03 |
|  |  |  | A_11_P0000033158 |  | -2.03 |
|  |  |  | A_11_P217963 | G patch domain containing 8 | -2.03 |
|  |  |  | A_11_P074006 | cortactin | -2.03 |
|  |  |  | A_11_P0000015860 | mitogen-activated protein kinase 9 | -2.03 |
|  |  |  | A_11_P200958 | Scm-like with four mbt domains 1 | -2.02 |
|  |  |  | A_11_P095351 | putative olfactory receptor ENSP00000348552-like | -2.02 |
|  |  |  | A_11_P101152 | interferon, alpha 7 | -2.02 |
|  |  |  | A_11_P0000014186 |  | -2.02 |
|  |  |  | A_11_P0000014688 |  | -2.02 |
|  |  |  | A_11_P099756 | aquaporin 10 | -2.02 |
|  |  |  | A_11_P0000017825 |  | -2.02 |
|  |  |  | A_11_P0000012766 |  | -2.02 |
|  |  |  | A_11_P170343 |  | -2.02 |
|  |  |  | A_11_P0000033919 | phospholipase C, delta 3 | -2.02 |
|  |  |  | A_11_P083321 | senataxin | -2.02 |
|  |  |  | A_11_P0000022676 | spastic paraplegia 20 (Troyer syndrome) | -2.02 |
|  |  |  | A_11_P082966 | integrin, alpha E (antigen CD103, human mucosal lymphocyte antigen 1; alpha polypeptide) | -2.02 |
|  |  |  | A_11_P0000033110 | sodium channel, non-voltage-gated 1, gamma subunit | -2.02 |
|  |  |  | A_11_P0000019276 |  | -2.02 |
|  |  |  | A_11_P140146 |  | -2.02 |
|  |  |  | A_11_P127401 |  | -2.02 |
|  |  |  | A_11_P080226 |  | -2.02 |
|  |  |  | A_11_P0000040117 |  | -2.02 |
|  |  |  | A_11_P0000028872 | pyroglutamyl-peptidase I | -2.02 |
|  |  |  | A_11_P200123 | myeloid/lymphoid or mixed-lineage leukemia (trithorax homolog, Drosophila); translocated to, 4 | -2.02 |
|  |  |  | A_11_P081731 | male-specific lethal 1 homolog (Drosophila) | -2.02 |
|  |  |  | A_11_P0000011206 |  | -2.02 |
|  |  |  | A_11_P063701 | GDNF family receptor alpha 1 | -2.02 |
|  |  |  | A_11_P0000041619 |  | -2.02 |
|  |  |  | A_11_P0000017792 |  | -2.02 |
|  |  |  | A_11_P0000040395 |  | -2.02 |
|  |  |  | A_11_P000009102 |  | -2.02 |
|  |  |  | A_11_P0000030806 | neuregulin 2 | -2.02 |
|  |  |  | A_11_P168633 | family with sequence similarity 40, member A | -2.02 |
|  |  |  | A_11_P207258 | shisa family member 5 | -2.02 |
|  |  |  | A_11_P145181 | ankyrin repeat domain 23 | -2.02 |
|  |  |  | A_11_P082836 | solute carrier family 43 (amino acid system L transporter), member 2 | -2.02 |
|  |  |  | A_11_P054746 | defensin, beta 124 | -2.02 |
|  |  |  | A_11_P0000025089 | erythrocyte membrane protein band 4.1-like 3 | -2.02 |
|  |  |  | A_11_P0000040569 |  | -2.02 |
|  |  |  | A_11_P0000025513 |  | -2.02 |
|  |  |  | A_11_P0000032956 | vacuolar protein sorting 37 homolog D (S. cerevisiae) | -2.02 |
|  |  |  | A_11_P0000010461 |  | -2.02 |
|  |  |  | A_11_P0000023726 | homogentisate 1,2-dioxygenase | -2.02 |
|  |  |  | A_11_P000009913 |  | -2.02 |
|  |  |  | A_11_P0000032691 | ubiquitin specific peptidase 43 | -2.02 |
|  |  |  | A_11_P0000011537 |  | -2.02 |
|  |  |  | A_11_P000007734 |  | -2.02 |
|  |  |  | A_11_P0000011874 |  | -2.02 |
|  |  |  | A_11_P128496 |  | -2.02 |
|  |  |  | A_11_P065196 |  | -2.02 |
|  |  |  | A_11_P0000023249 | Sec61 alpha 2 subunit (S. cerevisiae) | -2.02 |
|  |  |  | A_11_P092976 | glia maturation factor, beta | -2.02 |
|  |  |  | A_11_P0000041175 |  | -2.02 |
|  |  |  | A_11_P074066 | choline kinase alpha | -2.02 |
|  |  |  | A_11_P0000025235 |  | -2.02 |
|  |  |  | A_11_P0000016258 | solute carrier family 27 (fatty acid transporter), member 5 | -2.02 |
|  |  |  | A_11_P000009225 |  | -2.02 |
|  |  |  | A_11_P0000010008 |  | -2.02 |
|  |  |  | A_11_P177508 |  | -2.02 |
|  |  |  | A_11_P0000029203 |  | -2.02 |
|  |  |  | A_11_P215928 |  | -2.02 |
|  |  |  | A_11_P139846 | glia maturation factor, beta | -2.02 |
|  |  |  | A_11_P196633 | collagen, type XII, alpha 1 | -2.02 |
|  |  |  | A_11_P0000015968 |  | -2.02 |
|  |  |  | A_11_P105956 | ubiquitination factor E4A | -2.02 |
|  |  |  | A_11_P156613 |  | -2.01 |
|  |  |  | A_11_P075716 | solute carrier family 38, member 3 | -2.01 |
|  |  |  | A_11_P0000041953 |  | -2.01 |
|  |  |  | A_11_P0000023086 | meningioma expressed antigen 5 (hyaluronidase) | -2.01 |
|  |  |  | A_11_P192213 | ubiquitin specific peptidase 34 | -2.01 |
|  |  |  | A_11_P055296 | cyclin-dependent kinase inhibitor 1B (p27, Kip1) | -2.01 |
|  |  |  | A_11_P126351 |  | -2.01 |
|  |  |  | A_11_P050901 | peroxisome proliferator-activated receptor alpha | -2.01 |
|  |  |  | A_11_P159723 | RAB5C, member RAS oncogene family | -2.01 |
|  |  |  | A_11_P175813 | transmembrane protein 63A | -2.01 |
|  |  |  | A_11_P119581 | down-regulator of transcription 1, TBP-binding (negative cofactor 2) | -2.01 |
|  |  |  | A_11_P0000034835 | stromal antigen 2 | -2.01 |
|  |  |  | A_11_P105366 | myotubularin related protein 12 | -2.01 |
|  |  |  | A_11_P0000023431 | vacuolar protein sorting 13 homolog D (S. cerevisiae) | -2.01 |
|  |  |  | A_11_P160368 |  | -2.01 |
|  |  |  | A_11_P089916 | mannosyl (alpha-1,3-)-glycoprotein beta-1,4-N-acetylglucosaminyltransferase, isozyme A | -2.01 |
|  |  |  | A_11_P104246 | leucine rich repeat containing 20 | -2.01 |
|  |  |  | A_11_P000008853 |  | -2.01 |
|  |  |  | A_11_P194093 | GC-rich sequence DNA-binding factor 2 | -2.01 |
|  |  |  | A_11_P0000031446 | Cbl proto-oncogene B, E3 ubiquitin protein ligase | -2.01 |
|  |  |  | A_11_P140126 | TCDD-inducible poly(ADP-ribose) polymerase | -2.01 |
|  |  |  | A_11_P085496 | leucine rich repeat containing 41 | -2.01 |
|  |  |  | A_11_P0000010390 |  | -2.01 |
|  |  |  | A_11_P0000022860 | acetyl-CoA carboxylase beta | -2.01 |
|  |  |  | A_11_P0000015630 | troponin I type 1 (skeletal, slow) | -2.01 |
|  |  |  | A_11_P152823 |  | -2.01 |
|  |  |  | A_11_P093496 | tubulin tyrosine ligase-like family, member 5 | -2.01 |
|  |  |  | A_11_P0000022369 |  | -2.01 |
|  |  |  | A_11_P0000025259 |  | -2.01 |
|  |  |  | A_11_P000008094 |  | -2.01 |
|  |  |  | A_11_P0000016120 |  | -2.01 |
|  |  |  | A_11_P075501 | Scm-like with four mbt domains 1 | -2.01 |
|  |  |  | A_11_P168348 | transcriptional regulating factor 1 | -2.01 |
|  |  |  | A_11_P154888 |  | -2.01 |
|  |  |  | A_11_P056206 | small nuclear ribonucleoprotein 48kDa (U11/U12) | -2.01 |
|  |  |  | A_11_P0000015608 |  | -2.01 |
|  |  |  | A_11_P0000024621 | pyruvate dehydrogenase phosphatase regulatory subunit | -2.01 |
|  |  |  | A_11_P0000014025 |  | -2.01 |
|  |  |  | A_11_P0000022315 |  | -2.01 |
|  |  |  | A_11_P0000034245 | leucine rich repeat containing 8 family, member A | -2.01 |
|  |  |  | A_11_P214023 | leucine rich repeat containing 8 family, member A | -2.01 |
|  |  |  | A_11_P0000040233 |  | -2.01 |
|  |  |  | A_11_P0000013897 |  | -2.01 |
|  |  |  | A_11_P0000030774 | family with sequence similarity 188, member A | -2.01 |
|  |  |  | A_11_P0000041796 |  | -2.01 |
|  |  |  | A_11_P0000041333 |  | -2.01 |
|  |  |  | A_11_P155218 |  | -2.01 |
|  |  |  | A_11_P179343 |  | -2.01 |
|  |  |  | A_11_P000009390 | putative speedy protein-like protein 3-like | -2.01 |
|  |  |  | A_11_P200633 | LEM domain containing 2 | -2.01 |
|  |  |  | A_11_P0000012080 |  | -2.01 |
|  |  |  | A_11_P126041 |  | -2.01 |
|  |  |  | A_11_P0000031268 | keratin associated protein 11-1 | -2.01 |
|  |  |  | A_11_P0000018109 |  | -2.01 |
|  |  |  | A_11_P0000039822 |  | -2.01 |
|  |  |  | A_11_P0000029011 | patatin-like phospholipase domain containing 6 | -2.00 |
|  |  |  | A_11_P163883 |  | -2.00 |
|  |  |  | A_11_P0000032364 | HMP19 protein | -2.00 |
|  |  |  | A_11_P065796 | anoctamin 6 | -2.00 |
|  |  |  | A_11_P138606 |  | -2.00 |
|  |  |  | A_11_P0000018688 |  | -2.00 |
|  |  |  | A_11_P130676 |  | -2.00 |
|  |  |  | A_11_P079186 | protein phosphatase 1, regulatory subunit 9A | -2.00 |
|  |  |  | A_11_P000005386 | hook microtubule-tethering protein 3 | -2.00 |
|  |  |  | A_11_P092766 | nuclear factor of kappa light polypeptide gene enhancer in B-cells inhibitor, alpha | -2.00 |
|  |  |  | A_11_P075881 | 6-phosphofructo-2-kinase/fructose-2,6-biphosphatase 4 | -2.00 |
|  |  |  | A_11_P0000014989 | chemokine (C-C motif) ligand 24 | -2.00 |
|  |  |  | A_11_P000008703 |  | -2.00 |
|  |  |  | A_11_P057186 | G protein-coupled receptor 155 | -2.00 |
|  |  |  | A_11_P101856 |  | -2.00 |
|  |  |  | A_11_P00000291 | K(lysine) acetyltransferase 6A | -2.00 |
|  |  |  | A_11_P0000017179 |  | -2.00 |
|  |  |  | A_11_P052946 | NPC1 (Niemann-Pick disease, type C1, gene)-like 1 | -2.00 |
|  |  |  | A_11_P0000032505 | Fli-1 proto-oncogene, ETS transcription factor | -2.00 |
|  |  |  | A_11_P0000027667 |  | -2.00 |
|  |  |  | A_11_P121271 | TSC22 domain family, member 3 | -2.00 |
|  |  |  | A_11_P0000029871 | SH3 domain containing ring finger 1 | -2.00 |
|  |  |  | A_11_P000006365 |  | -2.00 |
|  |  |  | A_11_P063712 | pancreatic lipase | -2.00 |
